# Single-Tube Mass Spectrometry-Based Workflow for Multi-Omics Profiling of Diverse Biomolecules

**DOI:** 10.1021/acs.analchem.6c03036

**Published:** 2026-09-14

**Authors:** Maxim Itkin, Bareket Dassa, Yishai Levin, Andrii Vainer, Asaph Aharoni, Sergey Malitsky

**Affiliations:** † Life Sciences Core Facilities, 34976 Weizmann Institute of Science, Rehovot 7610001, Israel; ‡ Department of Plant and Environmental Sciences, Weizmann Institute of Science, Rehovot 7610001, Israel; § de Botton Institute for Protein Profiling, Israel National Center for Personalized Medicine, Weizmann Institute of Science, Rehovot 7610001, Israel

## Abstract

Advances in mass spectrometry-based omics technologies enable detailed exploration of biological processes. However, robust detection of multiple biomolecule types from the same biological sample remains challenging. We developed a multi-omics platform for the simultaneous detection of metabolites (polar and semi-polar), lipids, proteins, and native peptides, all detected in a high-throughput manner from a single tube. As a proof-of-concept for the platform, we profiled the profound and dynamic molecular changes occurring in tomato during fruit development. This included optimizing sample collection, standardizing data acquisition from the chromatographic systems, and developing a bioinformatic pipeline to integrate data from the diverse omics technologies. Across omics layers, we detected thousands of biomolecules whose abundances shifted significantly during fruit development in peel and flesh tissues, providing temporal signatures that differentiate fruit developmental stages. This includes the characterization of numerous naturally produced endogenous peptides that exhibit dynamic changes in composition and cleavage preferences. Finally, we demonstrate the use of a machine learning framework for unsupervised integration of this type of high-throughput data. Collectively, our multi-omics platform enables a comprehensive exploration of diverse biological tissues and biofluids with broad applicability across research fields.

## Graphical abstract

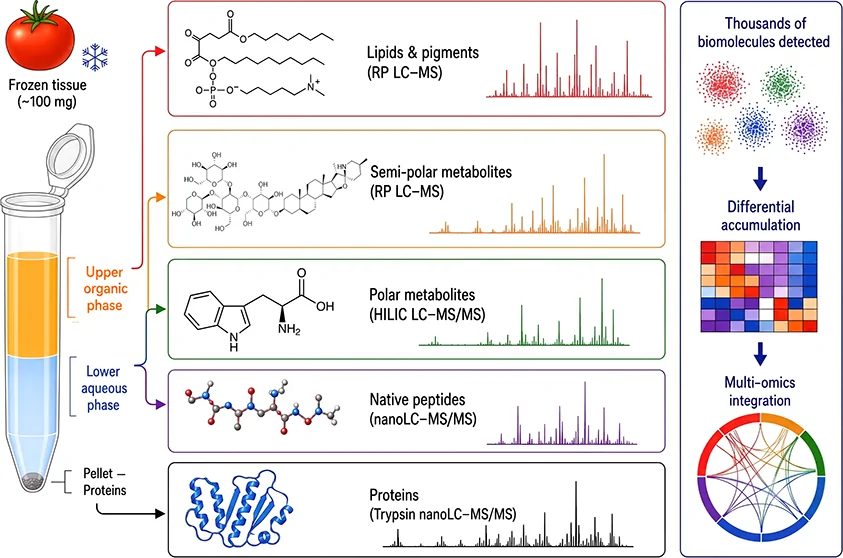

## Introduction

Considerable effort is being devoted to deciphering the complexity of biological systems by profiling multiple layers of information, such as gene, protein, metabolite, and lipid abundances, to obtain a comprehensive overview of cellular functions. While standard protocols exist for the separate extraction of each biomolecule class, there are clear advantages to workflows that enable simultaneous extraction of multiple molecular species from a single sample aliquot. This approach not only reduces sample handling time and variability but also facilitates cross-omics integration, potentially uncovering distinct molecular signatures. In recent years, there has been growing interest in integrative, system-level approaches to developmental and stress biology. Foundational guidance for mass spectrometry (MS)-based metabolomics, including annotation, quantification, and reporting standards, has been articulated.^1^ In parallel, MS-based workflows enabling multi-omics analysis from limited material have been demonstrated, including single-sample fractionated extraction of metabolites, lipids, and proteins in plants,^2,3^ and integrated metabolomics-peptidomics studies in human samples.^4^ Additional multi-omics workflows from the Coon laboratory enabled the joint analysis of proteins, lipids, and/or metabolites in diverse biological systems.^5–7^ However, the reported molecular coverage, ranging from dozens to a few thousand molecules, or restriction to specific omics types, still falls short of capturing the full cellular complexity, limiting the potential for comprehensive multi-omics data integration. These challenges arise, in part, from differences in extraction requirements and analytical coverage across metabolomics and lipidomics workflows.^8^

The multiple stages of tomato fruit (*Solanum lycopersicum*) development and ripening constitute a complex biological process that involves highly coordinated biochemical reprogramming, including dynamic shifts in primary and secondary metabolism, lipid remodeling, and changes in protein expression.^9–12^ Yet truly multilayered analyses capturing a broad spectrum of molecular species, ranging from small metabolites to larger peptides and proteins involved in fruit development, have been reported only to a limited extent. Moreover, small native peptides are increasingly recognized as important regulators of plant development and stress responses;^13–16^ however, they have rarely been systematically studied during fruit ripening. Such peptides typically range from 2 to 65 amino acids, often result from proteolytic cleavage of precursor proteins, and may undergo post-translational modifications such as hydroxylation or glycosylation.^17^ Yet their low abundance and diverse physicochemical properties have restricted comprehensive profiling in complex tissues such as fruit.

In this work, we conducted an integrated multi-omics investigation of tomato fruit development, focusing on peel and flesh tissues across eight defined fruit developmental stages. Using a modified biphasic extraction protocol (methyl *tert*-butyl ether (MTBE)/methanol/water system),^2,18^ we partitioned polar, semi-polar metabolites and native peptides into the aqueous phase, lipids into the organic phase, and extracted proteins from the remaining pellet. This strategy, adapted from protocols originally optimized for high-throughput lipidomics,^18^ enables simultaneous recovery of polar and semi-polar metabolites, lipids (including pigments, a subclass of lipophilic molecules), and thousands of proteins and native peptides extracted from a single biological sample. By generating all omics layers from the same biological specimen and extraction event, the workflow may reduce subsampling and extraction-related variability and facilitate direct cross-omics integration. Our workflow also enables extensive profiling of native peptides and the integration of data across all molecular levels.

## Experimental Section

### Experimental Design

Tomato plants (*Solanum lycopersicum*; cultivar Micro-Tom) were cultivated under natural light in a greenhouse at 24 °C during the day and 18 °C at night. At anthesis, flowers were labeled, and fruit were subsequently collected according to their developmental stage, as characterized by size and color. Fruit samples were collected at the following developmental stages: 3 weeks post-anthesis (3 w) (20–21 days post-anthesis (DPA)), immature green (IG) (ca. 25 DPA), mature green (MG) (36–40 DPA), early/late breaker (EBR/LBR) (40–44 DPA), early/late orange (EOR/LOR) (44–48 DPA), and red ripe (RR, 50–52 DPA). Since individual fruits developed at different rates, the specific DPA for each sample varied. Upon harvest, the fruits were immediately dissected with a scalpel to separate the peel and flesh tissues. The tissues were flash-frozen, stored at −80 °C, and ground to a fine powder before extraction. Each biological replicate consisted of pooled fruit from three independent plants (a minimum of three fruits per plant). Seeds, including the associated mucilaginous tissue (jelly), and sepals were not collected.

### Overview of the Multi-Omics Workflow

An overview of the integrated sample preparation and analytical workflow is shown in Figure 1A. Briefly, approximately 100 mg of tomato tissue was subjected to a single biphasic MTBE/methanol extraction, generating organic, polar, and residual protein fractions. These fractions were subsequently processed for lipidomics and pigment analysis; polar and semi-polar metabolomics, native peptidomics; and bottom-up proteomics, respectively, as described below.

**Figure 1:**
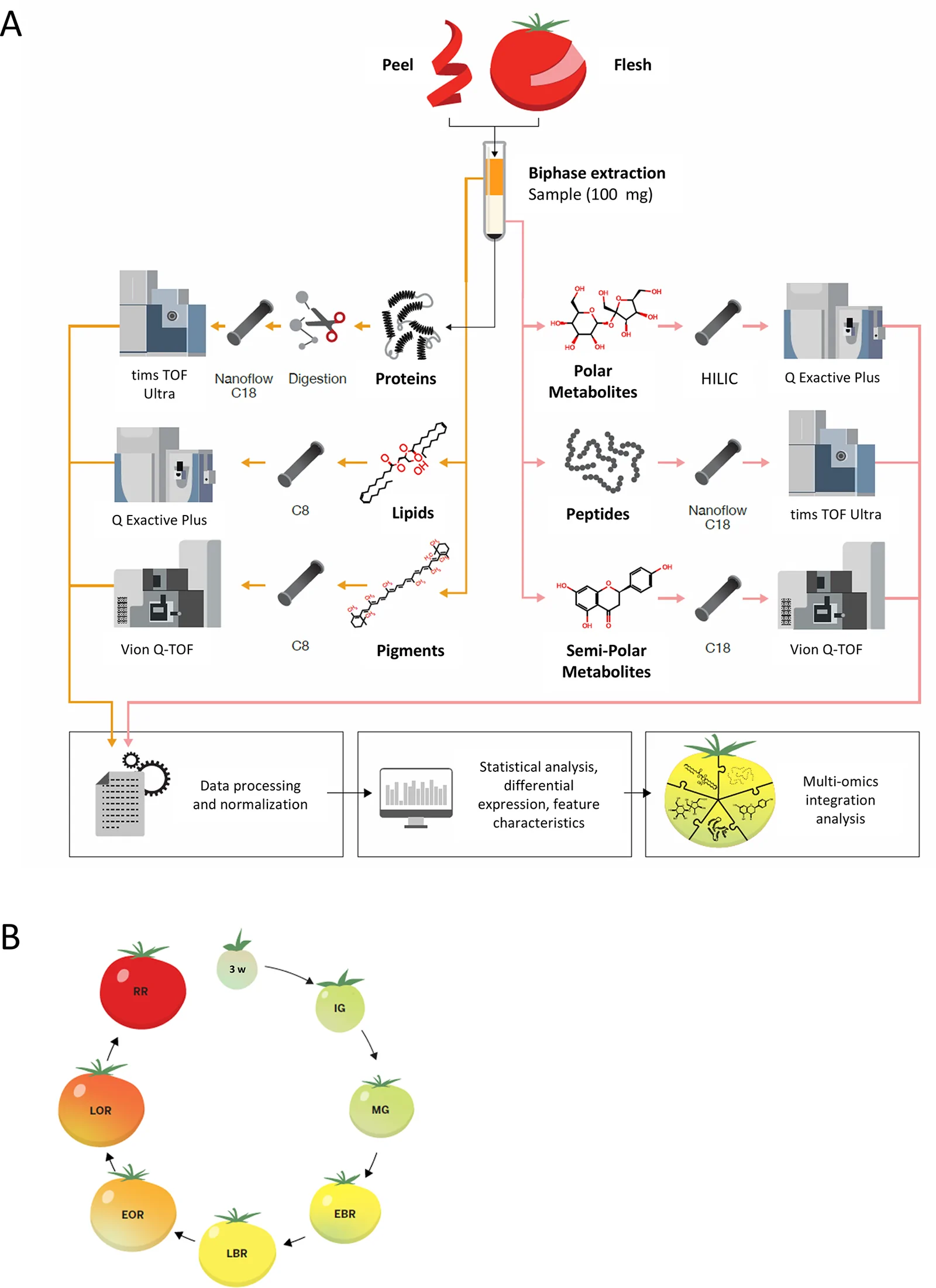
Experimental methodology and analysis workflow. (A) A multi-omics platform for the simultaneous detection of both metabolites (polar and semi-polar), lipids, proteins, and peptides from the same biological sample. Biomolecules were extracted from either aqueous or organic phases, and the data were processed and normalized using dedicated pipelines for statistical analysis and differential accumulation testing, yielding an integrated, multi-layer interpretation. (B) The experimental setup included the extraction of biomolecules from tomato fruit at eight developmental stages (green to red), from either peel or flesh tissues in three or more replicates. 3 w, 3 weeks post-anthesis; IG, immature green; MG, mature green; EBR, early breaker; LBR, late breaker; EOR, early orange; LOR, late orange; RR, red ripe.

### Metabolite Extraction

Extraction and analysis of polar metabolites and lipids were conducted following previously established methods by Malitsky et al.^19^ and Zheng et al.,^20^ with minor modifications. Approximately 100 mg of frozen (−80 °C) ground tomato flesh or peel was extracted under red light using 1 mL of a pre-chilled (−20 °C) methanol/Methyl *tert*-butyl ether (MTBE) mixture at a 1:3 (v/v) ratio. Internal standards included phosphatidylcholine (17:0/17:0, Avanti; 0.1 μg/mL), phosphatidylethanolamine (17:0/17:0, Avanti; 0.4 μg/mL), Ceramide/Sphingoid Internal Standard Mixture II (Avanti, LM6005; 0.15 nmol/mL), d5-triacylglycerol Internal Standard Mixture I (Avanti, LM6000; 0.0267 μg/mL), and palmitic acid-13C (Sigma, 605573; 0.1 μg/mL). Samples were briefly vortexed, sonicated for 30 min in an ice-cold bath (with intermittent vortexing every 10 min), and then mixed with 0.5 mL of DDW/methanol (3:1, v/v), containing labeled amino acid standards (^13^C and ^15^N; Sigma, 767964; 1:1500 dilution). After thorough vortexing and centrifugation, the upper organic phase was collected. The remaining polar phase was extracted using an additional 0.5 mL MTBE. The combined organic extract and the polar phase were collected separately, dried under a gentle stream of nitrogen, and stored at −80 °C. The residual protein-containing pellet was dried in a vacuum concentrator and retained for bottom-up proteomic analysis. The dried polar fraction was subsequently used for semi-polar metabolite profiling and endogenous peptidomics.

### LC-MS Analysis of Polar Metabolites

Dried polar extracts were reconstituted in 150 μL methanol:DDW (50:50, v/v), centrifuged twice, and 80 μL was transferred to UPLC vials. Analysis utilized an Acquity I-class UPLC system (Waters) coupled with a Q Exactive Plus Orbitrap mass spectrometer (Thermo Fisher Scientific) operating in negative ionization mode. Metabolites were separated using a SeQuant Zic-pHilic column (150 × 2.1 mm) with a corresponding guard column (Merck). Mobile phases comprised ACN (phase B) and 20 mM ammonium carbonate with 0.1% ammonium hydroxide in DDW/ACN (80:20, v/v; phase A). The flow rate was 200 μL/min at 45 °C. The gradient was: 75% B (0–2 min), decreasing to 25% B by 14 min, holding until 18 min, returning to 75% B at 19 min, and equilibration until 23 min. Injection volume was 2 μL. Mass spectra were acquired in negative heated electrospray ionization (HESI) mode over the mass range 70–1050 *m*/*z*. Source parameters: capillary temperature 325 °C, spray voltage 3.25 kV, sheath gas 40 units, auxiliary gas 10 units, auxiliary gas temperature 50 °C. MS1 spectra were acquired at a 35,000 FWHM resolution, and MS/MS data collection was performed at a 17,500 FWHM resolution with a 3 *m*/*z* isolation window.

### LC-MS for Lipidomics and Pigment Analysis

Dried lipid extracts were resuspended in 150 μL ACN/isopropanol (ACN/IPA, 75:25, v/v), vortexed, and centrifuged. Supernatants (130 μL) were analyzed using an Acquity I-class UPLC coupled with either a Q Exactive Plus Orbitrap mass spectrometer (lipids) or a Vion IMS QTof mass spectrometer (pigments). Chromatography employed an ACQUITY UPLC BEH C8 column (100 × 2.1 mm, 1.7 μm). Mobile phase A was DDW/ACN/IPA (46:38:16, v/v/v) containing 1% 1 M NH_4_Ac and 0.1% acetic acid; phase B was DDW/ACN/IPA (1:69:30, v/v/v) with identical additives. The flow rate was 0.4 mL/min at 40 °C. Gradient conditions: 100% A (0–1 min), decreasing to 25% A at 12 min, then to 0% A at 16 min, holding 100% B until 21 min, returning to 100% A at 21.5 min, with equilibration until 25 min. Injection volume was 2 μL. Lipid data were collected in positive and negative switching HESI mode (*m*/*z* 100–1500) at a capillary temperature of 275 °C, a spray voltage of 3.1 kV, a sheath gas flow of 60 units, an auxiliary gas flow of 20 units, and an auxiliary gas temperature of 300 °C. MS1 resolution was 35,000 FWHM, MS/MS resolution 17,500 FWHM (1 *m*/*z* isolation). Pigment analysis was performed using the Vion IMS QTof in positive ionization mode. Source parameters: source temperature 120 °C, desolvation temperature 450 °C, capillary voltage 3.0 kV, cone voltage 40 V, nitrogen flow (desolvation: 800 L/h; cone gas: 30 L/h). Full-scan HDMSE mode ranged from 50–2000 Da with collision energy ramps of 20–80 eV (high-energy) and 4 eV (low-energy).

### LC-MS Analysis of Semi-Polar Metabolites

Semi-polar metabolite profiling was performed using a Waters ACQUITY Premier UPLC system with a Vion IMS QTof mass spectrometer. LC conditions adapted from Itkin et al.^21^ included an ACQUITY UPLC BEH C18 column (100 × 2.1 mm, 1.7 μm). Initial mobile phase A (DDW/ACN, 95:5 v/v containing 0.1% FA) decreased from 100% to 72% by 22 min, 60% by 22.5 min, and 0% by 23 min; phase B (ACN containing 0.1% FA) ran at 100% until 26.5 min. Equilibration returned phase A to 100% by 28 min. For semi-polar analysis, the resuspended polar phase was used. Injection volume was 1 μL. Mass spectral analysis was performed in both positive and negative ionization modes. Source parameters included source/desolvation temperatures (120:350 °C), capillary voltages (positive mode: 1 kV; negative mode: 2 kV), cone voltage (40 V), and nitrogen gas flow rates (desolvation: 700 L/h, cone: 50 L/h positive mode, 100 L/h negative mode). HDMSE scan range was 50–2000 Da; collision energy ramps were 20–80 eV (high-energy), and 4 eV (low-energy).

### Metabolomics Data Processing

Polar metabolite data were analyzed using TraceFinder v.5.1 software (Thermo Fisher Scientific), identifying compounds based on accurate mass (≤5 ppm), retention time, and spectral libraries generated from injected standards. Lipid annotations were generated with LipidSearch v.4.2 software, using mass accuracy ≤5 ppm, and validated against a custom lipid library (excluding putative PEt (phosphatidylethanol)) and expected lipid elution order. Pigments and semi-polar compounds were analyzed with UNIFI software (v1.9.4, Waters Corp., MA, USA) and identified by accurate mass (≤5 ppm), fragmentation patterns, retention time, and ion mobility (CCS) (≤2%) values, with reference to in-house-generated semi-polar and pigment databases, based on injected standards and previously published data.^21^ Relative metabolite levels were normalized to internal standards and sample weight.

### Peptidomics Liquid Chromatography

UPLC/MS grade solvents were used for all chromatographic steps. For endogenous peptidomics analysis, the dried polar fraction obtained from the cold biphasic extraction was reconstituted in 200 μL of 0.1% trifluoroacetic acid (TFA). Although flash-freezing and extraction under cold organic-solvent conditions were used to suppress proteolytic activity, limited ex vivo peptide cleavage during sample preparation cannot be completely excluded. Each sample was loaded using splitless nano-ultra-performance liquid chromatography (nanoElute2, Bruker Daltonics, Germany). The mobile phases were A: double-distilled water (DDW) containing 0.1% formic acid (FA), and B: acetonitrile (ACN) containing 0.1% FA. Desalting of the samples was performed online using a reversed-phase PepMap trapping column (0.3 mm × 5 mm, Thermo Fisher Scientific, USA). The peptides were then separated using a C18 nano-column (75 μm internal diameter, 250 mm length, IonOpticks, Australia) at 0.3 μL/min. Peptides were eluted from the column into the mass spectrometer using the following gradient: 2% to 35% B in 30 min, 35% to 95% B in 4.65 min.

### Peptidomics Mass Spectrometry

The column was connected to a timsTOF Ultra mass spectrometer (Bruker Daltonics) via the Captive Spray nanoESI source. The MS was operated in data-dependent acquisition Parallel Accumulation Serial Fragmentation (DDA-PASEF) mode with the following settings: MS1 mass range 100–1700 Th. Resolution was set to 50,000. TIMS ramp 100 ms, ion mobility range of 0.64–1.7 1/K_0_. MS/MS was triggered from *z* = 1 to *z* = 5. Number of PASEF ramps = 5, target intensity of 20,000, threshold 500, collision energy 15 to 55 for ion mobility range of 0.6–1.6 1/K_0_, respectively. Dynamic exclusion was set to 0.4 min. Isolation width was set to 2 *m*/*z*.

### Peptidomics Data Processing

Raw mass spectrometry data were processed with FragPipe version 22 (https://github.com/Nesvilab/FragPipe) using the default ‘non-specific peptidomics’ workflow. Data were searched with MSFragger v4.1 against the tomato sequence database version 07, 2024 from UniProtKB, allowing identification of non-specific peptides of lengths 7 to 65 amino acids. The false discovery rate (FDR) was assessed using a target-decoy approach with the embedded ProteinProphet algorithm^22^ set to a maximum of 1%. Quantification was performed using IonQuant version 1.10.27, with normalization and inter-run matching. The mass spectrometry peptidomics data have been deposited to the ProteomeXchange Consortium via the PRIDE (https://www.ebi.ac.uk/pride/) partner repository under the dataset identifier PXD075865.

### Proteomics Sample Preparation

Residual protein-containing debris from the same biphasic extraction tube was lysed with 5% SDS in 50 mM Tris-HCl. Lysates were incubated at 96 °C for 5 min, followed by six cycles of 30 s sonication (Bioruptor Pico, Diagenode, USA). Protein concentration was measured using the BCA assay (Thermo Scientific, USA), and a total of 100 μg protein was reduced with 5 mM dithiothreitol and alkylated with 10 mM iodoacetamide in the dark. Phosphoric acid was added to the lysates to a final concentration of 1.2%. A mixture of methanol/50 mM ammonium bicarbonate (9:1, v/v) was then added to the samples. Each sample was then loaded onto an S-Trap 96-well mini-plate (Protifi, USA). Samples were then digested with trypsin (1:50 trypsin/protein) for 1.5 h at 47 °C. The digested peptides were eluted using 50 mM ammonium bicarbonate; trypsin was added to this fraction and incubated overnight at 37 °C. Two more elutions were made using 0.2% FA and 0.2% FA in 50% ACN. The three elutions were pooled and vacuum-centrifuged to dry. Samples were kept at −80 °C until analysis.

### Proteomics Liquid Chromatography

UPLC/MS grade solvents were used for all chromatographic steps. Digested samples were solubilized in 250 μL 0.1% trifluoroacetic acid (TFA). Each sample was loaded using splitless nano-ultra-performance liquid chromatography (nanoElute2, Bruker Daltonics, Germany). The mobile phase was A: DDW containing 0.1% FA, and B:ACN containing 0.1% FA. Desalting of the samples was performed online using a reversed-phase PepMap trapping column (0.3 mm × 5 mm, Thermo Fisher Scientific, USA). The peptides were then separated using a C18 nano-column (75 μm internal diameter, 250 mm length, IonOpticks, Australia) at 0.3 μL/min. Peptides were eluted from the column into the mass spectrometer using the following gradient: 2% to 35% B in 60 min, 35% to 95% B in 4.65 min.

### Proteomics Mass Spectrometry

The column was connected to a timsTOF Pro mass spectrometer (Bruker Daltonics) via the Captive Spray nanoESI source. The MS was operated in data-dependent acquisition Parallel Accumulation Serial Fragmentation (DDA-PASEF) mode with the following settings: MS1 mass range 100–1700 Th. Resolution was set to 50,000. TIMS ramp 100 ms, ion mobility range of 0.6–1.6 1/K_0_. MS/MS was triggered from *z* = 1 to *z* = 5. Number of PASEF ramps = 5, target intensity of 20,000, threshold 500, collision energy 15 to 55 for ion mobility range of 0.6–1.6 1/K_0_, respectively. Dynamic exclusion was set to 0.4 min. Isolation width was 2 *m*/*z*.

### Proteomics Data Processing

Raw mass spectrometry data were processed with FragPipe version 22 (https://github.com/Nesvilab/FragPipe) using the default LFQ-MBR workflow. Data were searched with MSFragger v4.1 against the tomato sequence database version 07, 2024 from UniProtKB, allowing identification of tryptic peptides; up to two miscleavages were permitted. FDR was set to a maximum of 1%. Quantification was performed using IonQuant version 1.10.27, with normalization and inter-run matching. Sample EBR_2 was excluded from the downstream analysis due to a technical problem. The mass spectrometry proteomics data have been deposited to the ProteomeXchange Consortium via the PRIDE (https://www.ebi.ac.uk/pride/) partner repository under the dataset identifier PXD075890.

### Bioinformatics Data Analysis

Raw intensities were preprocessed to exclude known contaminants and retain only identified biomolecules with non-zero values. Intensities were log 2-transformed and were analyzed without imputation of missing values. Statistical analysis of differentially accumulated features was applied to each pair of consecutive time points, using a combined ANOVA and G-test for independence of missing data (IMD-ANOVA) implemented in the pmartR workflow.^23^ We required at least 2 observations per group, or, for the IMD test (*G*-test), 3 missing values per group. *P*-values were adjusted to correct for multiple tests within a biomolecule, to perform post hoc pairwise comparisons (Tukey test for ANOVA or Holm test for *G*-test), and to perform FDR correction to account for testing the same hypothesis across multiple biomolecules. Differential biomolecules passed the FDR cutoff of ≤0.05. For visualization in heatmaps, missing values were replaced with a constant value of 1, rows were scaled, and the data were hierarchically clustered (Euclidean distance with either the complete or average method). Principal component analysis (PCA) was applied to non-imputed abundances (except for the peptidomics, where missing values were imputed). Integration of omics was performed using a probabilistic Bayesian framework for multi-omics integration, implemented in MOFA+.^24^ This algorithm attempts to identify latent factors in the data, and was applied to the entire dataset, namely 48 samples from flesh and peel, selecting the top variable features from each modality (3000 proteins, 5000 peptides, all polar metabolites, all semi-polar metabolites, and all lipids). Intensities were log 2­(*x* + 1)-transformed and analyzed using a Gaussian MOFA model with 10 factors. Flesh and peel were defined as ‘groups’ in the model. The 10 captured factors were not correlated with one another. Pathway enrichment testing for tomato proteins was applied using the ShinyGO functional enrichment analysis tool (https://bioinformatics.sdstate.edu/go/), using all MOFA input proteins as background, inspecting pathways with FDR ≤0.05.^25^

## Results and Discussion

### Development of a Multi-Omics Platform for Profiling Tomato Fruit Ripening

The overall multi-omics workflow is illustrated in Figure 1A. Using a single biphasic extraction from approximately 100 mg of tissue, we simultaneously recovered polar and semi-polar metabolites, lipids, pigments, proteins, and native peptides for downstream analyses.

This approach utilized a biphasic extraction protocol, partitioning the samples into aqueous and organic phases, thereby enabling comprehensive coverage of diverse molecular classes from a single 100 mg tissue sample.

Proteins were measured by ‘bottom-up’ mass spectrometry-based proteomics. Polar metabolites were separated using hydrophilic interaction chromatography (HILIC) and subjected to high-resolution MS analysis. Semi-polar metabolites and peptides were both analyzed using reversed-phase high-resolution MS. Lipids were extracted into the organic phase and profiled using reversed-phase high-resolution MS. Each omics dataset underwent dedicated data preprocessing and normalization pipelines, followed by differential accumulation testing, characterization of its biomolecules, and, eventually, an integrative statistical analysis across all omics layers.

### Experimental Design and Ripening Stages

The experimental setup aimed to map temporal molecular changes during tomato fruit development and maturation in two tissues (peel and flesh) with sampling at eight distinct stages spanning 3 weeks post-anthesis (3 w) to red ripe (RR) fruit (Figure 1B). Each stage was represented by at least three biological replicates, ensuring statistical rigor and reproducibility. The stages included: 3 weeks post-anthesis (3 w), immature green (IG), mature green (MG), early breaker (EBR), late breaker (LBR), early orange (EOR), late orange (LOR), and red ripe (RR). Visual phenotyping confirmed the progression of fruit color and morphology across developmental stages, consistent with established developmental benchmarks. This design enabled parallel extraction of different biomolecule types, supporting direct comparison across omics layers and tissue types.

### Comprehensive Detection of Biomolecules during Tomato Fruit Development

To evaluate the breadth of molecular coverage achieved by our workflow, we quantified the number of detected peptides, proteins, and annotated metabolites in both flesh and peel tissues of tomato fruit (Figure 2). The results demonstrate substantial molecular diversity captured across omics layers and tissue types. Native peptides represented the largest detected biomolecule class, with 35,533 and 20,902 unique peptides identified in the flesh and peel, respectively. This highlights the remarkable complexity of the native peptidome, particularly in fruit flesh, and demonstrates the effectiveness of the protocol for native peptide recovery, a molecular class that is often underrepresented in conventional multi-omics workflows. The number of proteins identified across tissues was comparable, with 6851 in the flesh and 6910 in the peel. Over a thousand metabolites were identified in the flesh and peel, including 319 polar metabolites, 209 semi-polar metabolites, 712 lipids, and 23 pigments, a subclass of lipophilic molecules, in the flesh. Together, these molecules reflect the diverse polar, semi-polar, and lipid metabolic landscapes present in both tissue types. A detailed list of all detected and annotated molecules before preprocessing of the dataset is provided in Supporting File S1.

**Figure 2:**
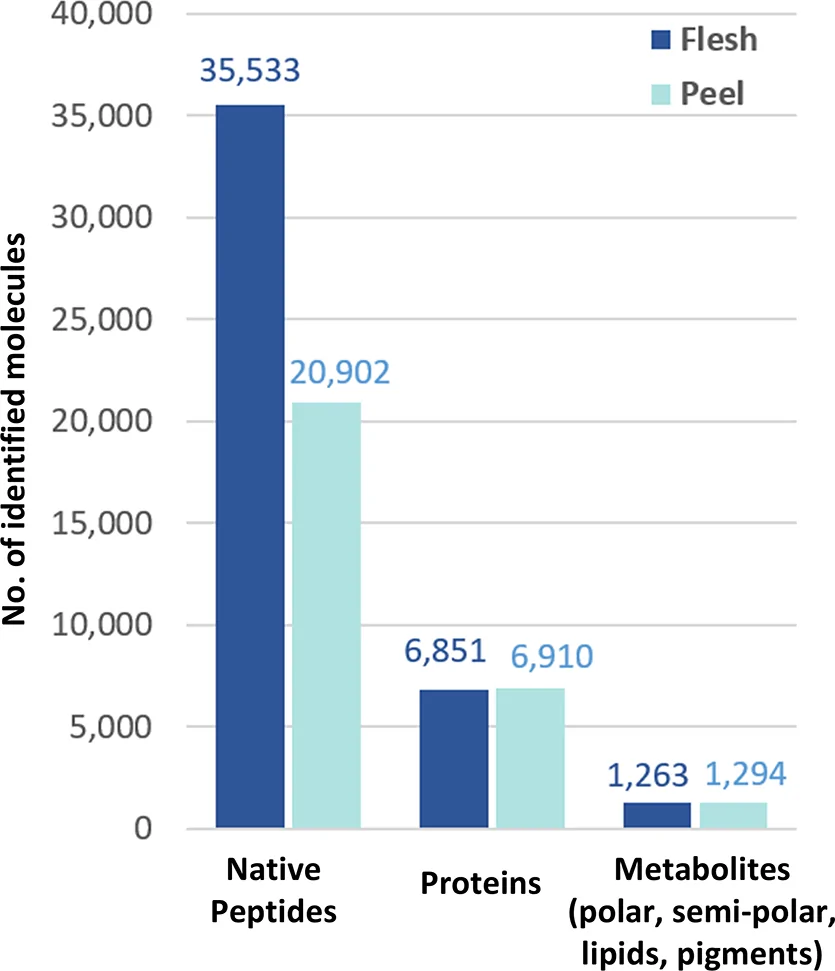
Total number of annotated biomolecules detected in each omics layer. Numbers denote unique biomolecules (after preprocessing), detected across all replicates of the eight tomato fruit stages in either flesh or peel tissues, for native peptide, protein, or metabolite datasets.

Collectively, these results underscore the capacity of our integrated extraction protocol to enable simultaneous, high-throughput profiling of thousands of peptides and proteins, as well as hundreds of polar metabolites, lipids, and pigments, from a single biological sample. These data establish a foundation for subsequent multi-omics integration and comparative analyses of metabolic and proteomic dynamics during tomato fruit development and ripening.

### Differential Analysis Reveals Distinct Fruit Developmental Trajectories across Omics Layers

To investigate the global patterns and temporal dynamics of molecular changes during tomato fruit ripening, we performed principal component analysis (PCA) of proteomics, peptidomics, and metabolomics datasets for flesh and peel tissues (Figure 3). The PCA of protein abundance in flesh samples revealed a clear progression along the first principal component (PC1), corresponding to the ripening stage. Early stages (3 w, IG) clustered distinctly from later stages (EOR, LOR, RR), with intermediate stages (MG, EBR) positioned accordingly along the trajectory. This pattern was highly reproducible across biological replicates, demonstrating robust protein-level shifts during fruit development and ripening.

**Figure 3:**
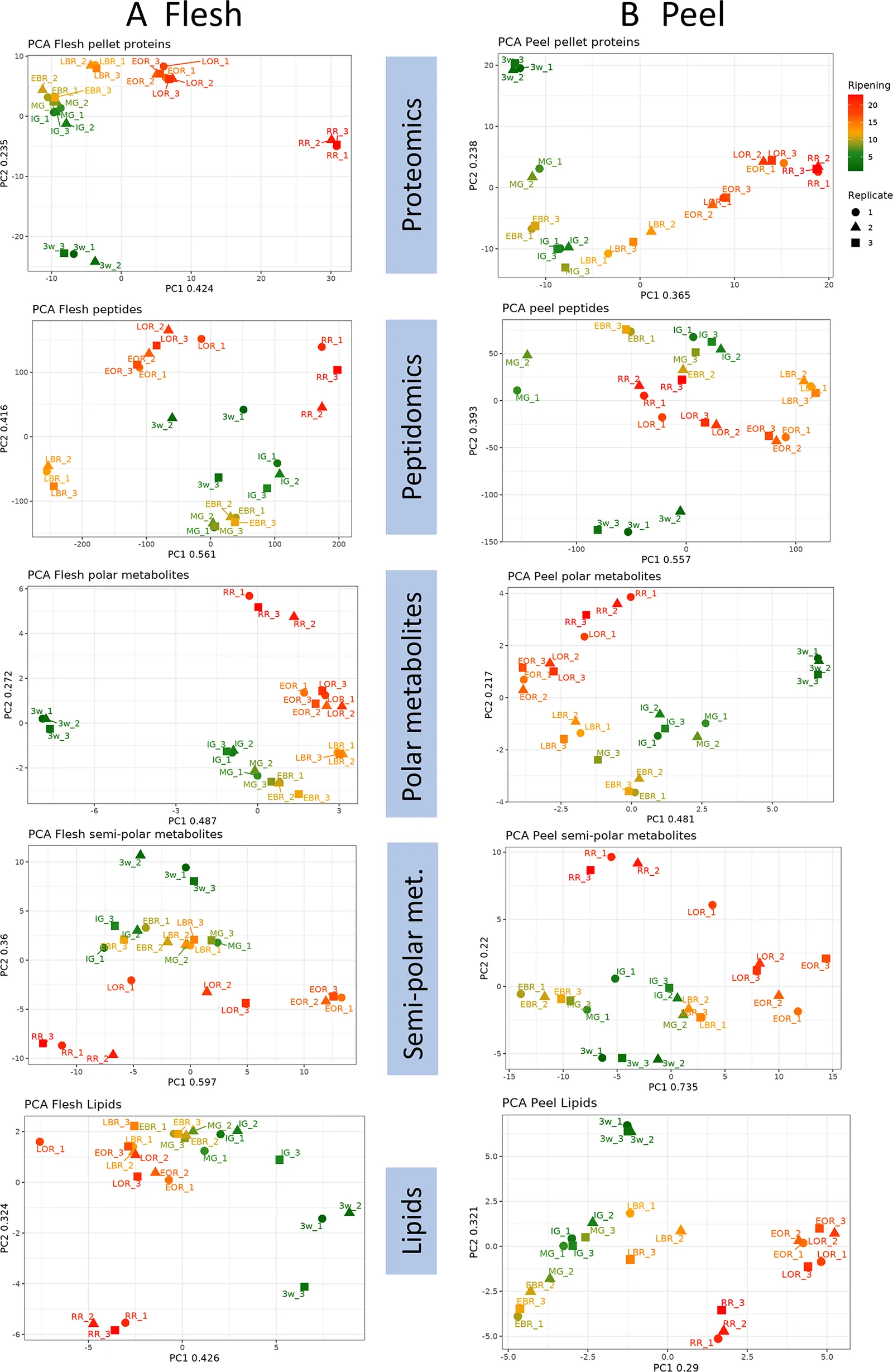
Principal component analysis (PCA) of all detected biomolecule accumulation profiles in five omics layers: protein expression, native peptides, polar metabolites, semi-polar metabolites, and lipids. Each point represents one biological replicate; samples are colored by developmental stage (green to red), and shapes indicate replicate number. Panels (A) and (B) show flesh and peel tissue, respectively. The proportion of variance captured by PC1 and PC2 is shown on the axes. Met., metabolites; 3 w, 3 weeks post-anthesis; IG, immature green; MG, mature green; EBR, early breaker; LBR, late breaker; EOR, early orange; LOR, late orange; RR, red ripe.

Interestingly, PCA of the native peptide data showed even larger separation of developmental stages along PC1 and PC2. It revealed subtle differences between intermediate stages (i.e., MG, EBR, LBR), suggesting that native peptide profiles provide higher temporal resolution than proteomics or metabolomics alone. The metabolites and lipids also captured the progression from immature to ripe stages, with clear separation along PC1. However, the clustering of metabolites at intermediate fruit stages was less distinct than that in the native peptide data, suggesting that metabolic changes may be more gradual or less stage-specific than peptidomics shifts.

The peel proteome displayed a similar developmental trajectory to that of the flesh tissue, with samples transitioning smoothly from early to late stages (in PC1). Replicate consistency was high, and the overall pattern mirrored that observed in the flesh. As in the flesh, native peptide PCA in the peel resolved ripening stages with high resolution. Notably, several intermediate stages (e.g., MG, EBR) were more distinctly separated than in the proteome or metabolome, again highlighting the peptidome’s sensitivity to developmental state. The peel polar metabolite samples showed clear separation in PCA between early and late stages, but greater overlap among intermediate stages, consistent with the flesh results.

The similarity in developmental trajectories between proteomics and peptidomics is consistent with coordinated regulation of proteins and peptides, but does not establish the origin of individual peptide species. However, the higher resolution of native peptide PCA, particularly in resolving intermediate stages, indicates that native peptides may serve as sensitive markers of developmental transitions.

### Differential Analysis Reveals Distinct Molecular Profiles during Ripening

We next analyzed the dataset and identified significant molecular changes associated with tomato fruit development. We performed a differential analysis of the accumulation of proteins, native peptides, polar and semi-polar metabolites and lipids in either flesh (Figure 4) or peel (Figure S1) tissues. Our comparison included consecutive developmental stage pairs and profiling of biomolecules showing significant abundance changes across stages (e.g., RR vs. LOR, EOR vs. LBR, LBR vs. EBR, MG vs. IG, IG vs. 3 w; see Figure S2). Figure 4A illustrates the proportion of differential molecules across comparisons of fruit stages, showing a higher proportion in transitions such as MG vs. IG or LOR vs. EOR, indicating major shifts in the molecular landscape at those specific points in fruit development and ripening. The heatmap generated from flesh proteomics data (Figure 4B) displays 5858 differentially expressed proteins. We observed a clear pattern of up- and down-regulation across the developmental time course. Proteins generally clustered into groups that are either highly abundant in early stages (3 w, IG) and decrease towards later stages, or conversely, are low in early stages and increase significantly as the fruit ripens (EOR, LOR, RR). Intermediate stages (MG, EBR, LBR) show transitional expression patterns. This broad shift in the proteome reflects the dramatic physiological and biochemical changes that occur during fruit maturation, including the production of ripening-related enzymes and the degradation of components from earlier developmental stages.

**Figure 4:**
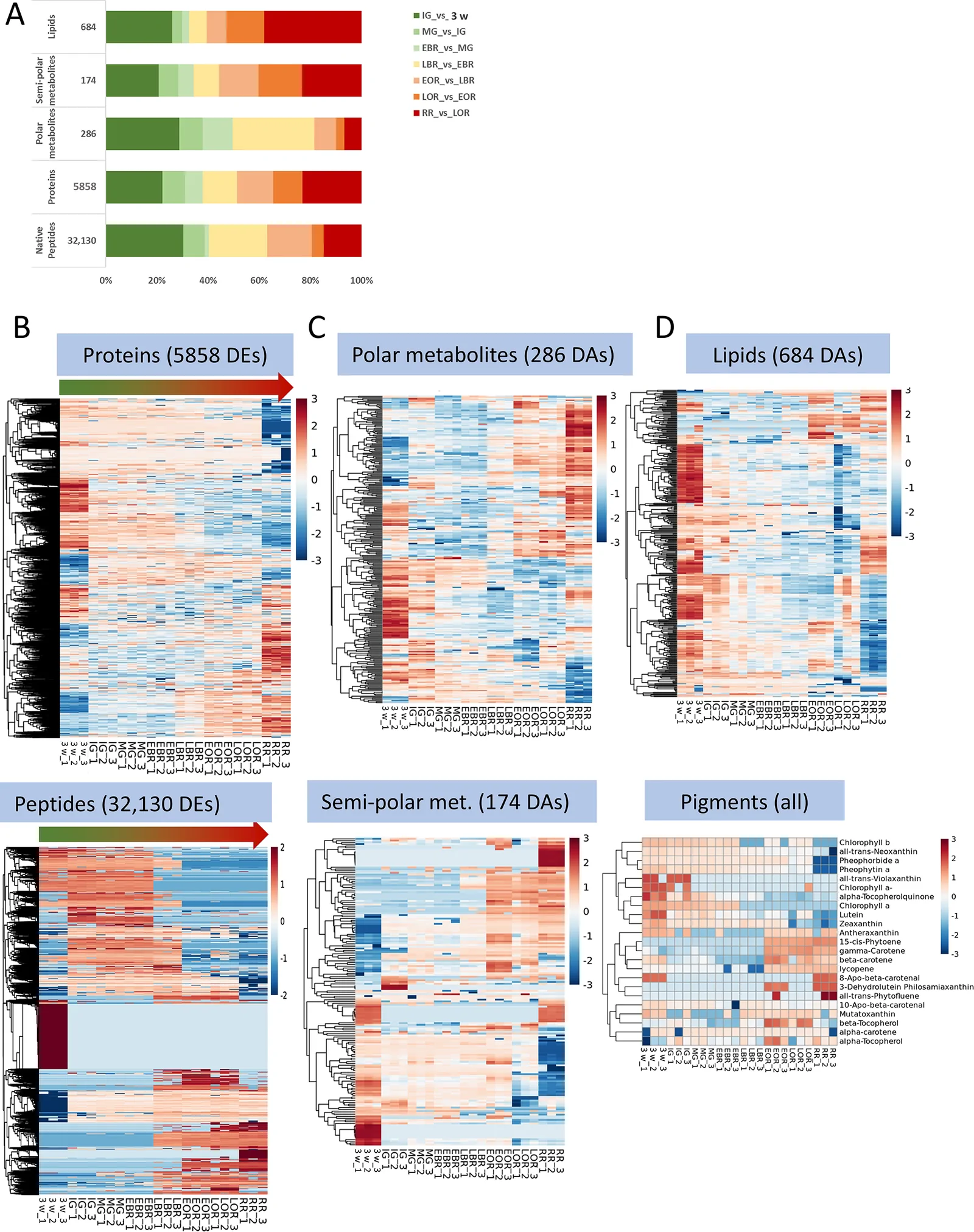
Differential accumulation of flesh biomolecules across ripening stages. Differentially accumulated biomolecules were detected between consecutive fruit developmental stages in each omics dataset (FDR cutoff of ≤0.05). (A) Proportions of differentially abundant molecules detected in each comparison, in each omics. The total numbers of differential molecules are noted on the left. (B) Heatmaps showing the relative abundance of significantly differentially abundant proteins and peptides across samples (C) Heatmaps of differentially abundant polar or semi-polar metabolites. (D) Heatmaps of differentially abundant lipids and pigments. Row measured values were scaled and displayed as colors ranging from red (highest) to blue (lowest), as shown in the key; missing values were imputed, and rows were hierarchically clustered. A similar analysis of the peel tissue is shown in Figure S1. Corresponding volcano plots of differentially accumulated molecules are shown in Figure S2. Met., metabolites; DE, differentially expressed; DA, differentially accumulated; 3 w, 3 weeks post-anthesis; IG, immature green; MG, mature green; EBR, early breaker; LBR, late breaker; EOR, early orange; LOR, late orange; RR, red ripe.

The native peptide heatmap (Figure 4B) revealed a substantially larger number of differentially abundant peptides, totaling 32,130. This large number underscores the dynamic nature of the peptidome during ripening and suggests that native peptides play a significant yet often overlooked role in this process. Similar to proteins, distinct clusters of peptide expression are evident, with some peptides showing high abundance in early stages and decreasing, while others increase towards full ripeness. A notable observation was a group of ∼460 peptides unique to the 3 w stage. This set of early-stage, unique peptides highlights specific proteolytic events or peptide-synthesis pathways active during very early development. The greater distinction between early and late stages in the peptide data, compared to proteins, further supports the PCA findings that peptides offer higher temporal resolution.

Metabolite heatmaps show 286 polar, 174 semi-polar metabolites (Figure 4C), and 684 lipids (Figure 4D) that were differentially accumulated. While fewer in number compared to proteins and peptides, these metabolites exhibit patterns of accumulation or depletion across stages, reflecting shifts in primary metabolism, secondary metabolite synthesis, and flavor compound development. Clustering generally separates early and late stages, similar to the broader trends seen in proteins and peptides, but perhaps with less fine-grained resolution of intermediate stages than in the peptidome.

During tomato fruit ripening, pronounced and well-characterized changes occur in pigment composition, particularly within the fruit flesh (Figure 4D). In immature green and mature green stages, chlorophyll a and b predominate, reflecting active chloroplast function. As ripening initiates at the breaker stage, chlorophylls and their derivatives (including pheophytin and pheophorbide) decline sharply, consistent with chloroplast-to-chromoplast conversion and dismantling of the photosynthetic apparatus.^11,26^ Concomitantly, carotenoid biosynthesis is strongly induced. Early carotenoid intermediates such as phytoene and phytofluene accumulate at breaker and orange stages, followed by a pronounced increase in lycopene, which becomes the dominant pigment in red ripe fruit and is responsible for the characteristic red coloration.^27,28^ β-Carotene and minor carotenoid species, γ-carotene, also increase during ripening, though to a lesser extent than lycopene. Xanthophylls associated with photosynthetic tissues, including lutein, violaxanthin, neoxanthin, and antheraxanthin, generally decrease as ripening progresses, reflecting the loss of chloroplast-localized pigments. Tocopherols remain abundant throughout development, consistent with their role in antioxidant protection during membrane remodeling. Similar qualitative trends are observed in the peel, although pigment abundances and timing may differ due to tissue-specific metabolic activity.

The differential accumulation analysis across proteins, peptides, lipids, and metabolites in tomato fruit flesh provides a comprehensive view of the molecular events underlying ripening. The large number of differentially abundant native peptides and their ability to offer finer temporal resolution suggest that the peptidome is a highly dynamic and informative layer in understanding fruit development.

### The Fruit Peptidome Reveals Distinct Features and Dynamic Cleavage Preferences during Development

We detected and quantified thousands of endogenous peptides, which are not the result of controlled proteolytic digestion of proteins (as in ‘bottom-up’ proteomics), and were therefore analyzed as endogenous peptide candidates. The differential peptidome analysis described above revealed stage-specific patterns and significant changes between adjacent developmental stages of fruit, evident in both flesh and peel (Figures 4 and S1). A comparative analysis further revealed that specific peptide clusters co-varied with changes in metabolites or proteins, suggesting potential functional coordination during ripening (see the next section). In addition to the soluble fractions, we leveraged the protein-rich pellet remaining after biphasic extraction for proteomics analysis, providing complementary information on the expression dynamics of larger proteins. Analyzing proteomics and peptidomics from the same sample allows comparison of putative source-protein abundance with the accumulation patterns of mapped peptide sequences. To further understand the nature of the native peptides detected (including all detected peptides, prior to preprocessing), we characterized their length distribution, protein origins, and amino acid cleavage site preferences within the tomato fruit (primarily flesh) peptidome.

### Length and Protein Origin of Native Peptides

Analysis of native peptides’ length distribution (Figure 5A) revealed that the majority of detected peptides from both flesh and peel tissues were relatively short. The distribution shows a peak around 10–12 amino acids (aa), with a rapid decrease in abundance for longer peptides. For flesh, the minimum peptide length was 7 aa, the median was 13 aa, and the average was 13.2 aa. In the peel, the median was 12 aa, and the average was 14.2 aa. This characteristic short length suggests either an inherent bias of the mass spectrometer toward the detection of short peptides, specific proteolytic cleavages rather than random protein degradation, or a combination of both.

**Figure 5:**
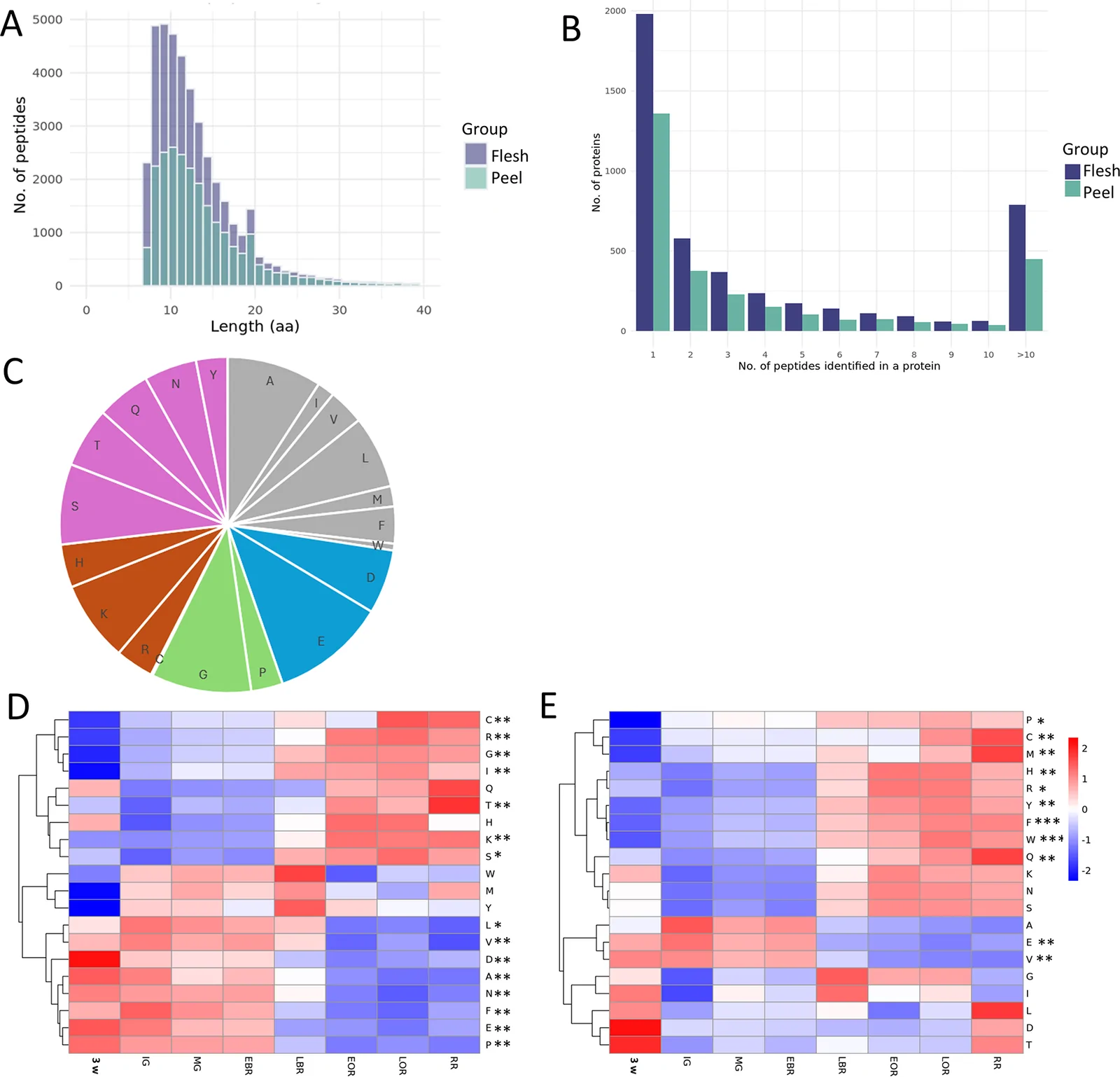
Characteristics of detected native peptides. (A) Distribution of peptide lengths, based on all 41,331 flesh and 23,560 peel detected peptides. (B) Number of endogenous peptides mapped to each putative source protein. (C) Proportion of the C-terminal amino acid cleavage-site of 41,331 native peptides detected in flesh samples, colored by the amino acid property (Non-polar, gray; Acidic, blue; Basic, red; Polar, pink; Other, green). (D) Changes in the frequency of the C-terminal amino acid in flesh peptides (from C) across ripening stages. (E) Changes in the proportions of amino acid composition of flesh peptides (from A) across fruit developmental stages. Heatmap rows were scaled to reflect high (red) or low (blue) frequencies, and hierarchically clustered using Euclidean distance and complete linkage. A significant linear trend is noted next to each row, where (*) means Padj <0.05, (**) Padj <0.01 and (***) Padj <0.001. 3 w, 3 weeks post-anthesis; IG, immature green; MG, mature green; EBR, early breaker; LBR, late breaker; EOR, early orange; LOR, late orange; RR, red ripe.

When examining the frequency of native peptides that originate from the same parent protein (Figure 5B), we observed that for both flesh and peel, 44% of mapped putative source proteins were represented by a single detected peptide (3338 parent proteins out of 7533). Because the analysis is peptide-centric, representation by a single peptide should not be interpreted as independent protein-level identification, particularly when a sequence may be shared among homologous proteins or isoforms. The data are based on 41,331 flesh and 23,560 peel peptides. The minimum number of peptides per protein was 1, while the median was 2 for both flesh and peel, and the average was 9 for flesh and 8 for peel. Surprisingly, the maximum number of peptides per protein was 2232 for flesh and 1863 for peel, indicating that a few proteins were extensively processed into numerous peptides. These findings suggest that while many proteins contribute a single unique peptide, a subset of proteins undergoes extensive or complex proteolytic processing.

### Amino Acid Composition and Cleavage Site Preferences of Peptides

Unlike peptides that are generated during the workflow of shotgun proteomics, where an enzymatic proteolytic digestion is applied (i.e., by trypsin) to parent proteins, native peptides are naturally produced in cells, and often their cleavage sites are not restricted to specific amino acids. We analyzed the proportion of amino acids at the cleavage site (C-terminus) of 41,331 flesh peptides (Figure 5C), revealing a diverse distribution of the last amino acid types favored at the C-terminal cleavage site. Figure S3 provides a comparison of the last amino acid proportions with the overall frequency of amino acids in the entire tomato proteome. Of note is the overrepresentation of amino acids such as A, E and G at the peptides’ C-terminus, and the underrepresentation of I and L amino acids. This potential preference in the cleavage site was further inspected below.

Notably, we observed dynamic changes in the composition and cleavage preferences across the eight stages. Figure 5D illustrates how the relative abundance of specific C-terminal amino acids is dynamic. For example, amino acids C, R, G, and I increase in frequency during later ripening, while P and D are more prevalent at the cleavage site in early stages. Similarly, Figure 5E reflects changes in the overall amino acid composition of the entire peptide pool during development. To determine the significance of these changes, the proportions of each amino acid were modeled independently using linear regression against ripening stages, yielding an associated *p*-value (Wald test on the slope coefficient) per amino acid, adjusted for multiple testing using the Benjamini-Hochberg procedure to control the false discovery rate (FDR). These patterns may suggest shifts in the proteolytic landscape throughout fruit development, with specific proteases activated or deactivated at different stages, leading to a dynamic peptidome with evolving cleavage specificities and overall amino acid profiles. To our knowledge, this represents the first system-level characterization of endogenous protease products during tomato fruit ripening.

### Peptidomics of ABSCISIC STRESS-RIPENING PROTEIN 1 and PECTIN METHYLESTERASE during Tomato Development and Maturation

To exemplify the breadth of molecular information captured by the peptidomics layer, we focused on two proteins whose peptide signatures exhibit clear developmental dynamics (Figure 6). Peptidomics profiling of tomato fruit flesh revealed native peptide series derived from the parent proteins ABSCISIC STRESS-RIPENING PROTEIN 1 (ASR1, UniProt ID O82575) and PECTIN METHYLESTERASE (PME, UniProt ID A0A3Q7HI50) that change across the 3 w-RR timeline. ASR1 is an abscisic acid-, stress-, and ripening-associated transcription factor. Cell-wall-related genes have been identified as ASR1 targets,^29^ and multi-omics analysis has implicated ASR1 in amino-acid metabolism and tomato fruit ripening.^30^ PME was shown to remove methyl esters from homogalacturonan in the primary cell wall of tomato fruit, which expresses several PME isoforms with distinct patterns of expression during ripening.^31,32^ Reduced PME activity was shown by Tieman et al.^33^ to alter tissue integrity in tomato, underscoring PME’s role in wall modification during maturation and senescence.

**Figure 6:**
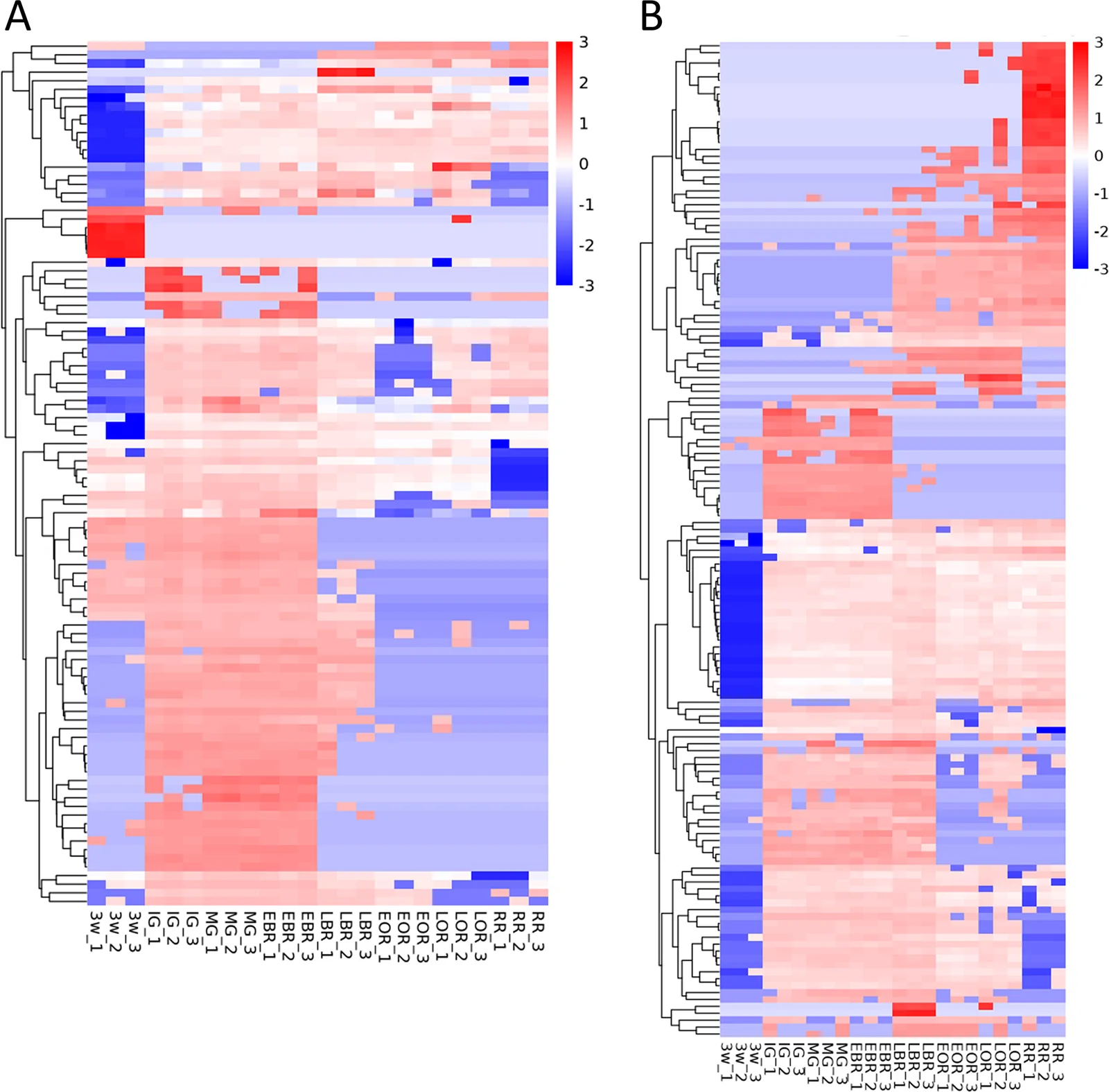
Abundance of detected native peptides in selected parent proteins. (A) Abscisic Stress-Ripening Protein 1 (ASR1) and (B) Pectin Methylesterase (PME) proteins. Heatmaps show the relative intensities of differentially abundant native peptides across developmental stages in flesh (values are log 2-transformed; missing values were imputed; rows were scaled and hierarchically clustered). 3 w, 3 weeks post-anthesis; IG, immature green; MG, mature green; EBR, early breaker; LBR, late breaker; EOR, early orange; LOR, late orange; RR, red ripe.

In our data, the abundance of native peptides assigned to ASR1 or PME did not always co-vary; peptides from different regions of the same protein showed different trajectories within a given ripening stage. This behavior is expected in native peptidomics, which measures *in vivo* protease products rather than uniform tryptic surrogates. Protein region-specific cleavage and fragment stability may result in peptides with different accumulation profiles. As native peptide signals reflect the steady-state interplay of fragment production and degradation, simply summing all peptides to infer a single protein-level quantification, as done in typical shotgun proteomics experiments, can consequently be misleading.

An apparent increase in precursor synthesis can coincide with faster processing, producing opposing trends among fragments and partial cancellation when aggregated. Accordingly, peptide-level results are interpreted here as indicators of proteolysis and turnover dynamics rather than as direct quantitative substitutes for intact-protein abundance. Within this context, stage-dependent ASR1 peptide changes are consistent with its reported role in ripening control,^30^ and PME-derived peptide diversity around breaker stages aligns with documented changes in PME expression and activity during fruit development and ripening.^31–33^

### Multi-Omics Unbiased Data Integration

We next summarized the profiles of all detected biomolecules across five omics layers, across eight tomato ripening stages, and two tissue types (flesh and peel). To integrate this dataset, we applied a machine learning method named MOFA (multi-omics factor analysis).^24^ MOFA is an unsupervised analysis that models both shared and omics-specific variation across the different data types (proteomics, peptidomics, polar and semi-polar metabolites, and lipids) via dimensionality reduction. This information differs from the differential analysis tests we presented above because it is not dependent on a specific, supervised comparison (e.g., comparing only consecutive developmental stages) and allows the identification of previously hidden latent sources of variation.

Overall, the variance captured by MOFA was 60–90% across most omics layers (Figure 7A), whereas polar metabolites and flesh lipids exhibited a lower proportion of variance explained by the model (<20%), likely reflecting greater biological heterogeneity and/or broader chemical diversity. These high-dimensional data were then reduced to ten latent factors that capture the most meaningful information across omics, fruit developmental stages, and tissue (Figure 7B). To interpret the biological relevance of each MOFA factor, we inspected the factor scores for the first ten MOFA factors (Figure 7C,D), and performed pathway enrichment analysis on the biomolecules contributing most strongly to each MOFA factor. Factors 1 and 2 captured the variation between the early (3 w to EBR) and late (LBR to RR) stages within the peel and flesh tissues, respectively. An example of the biomolecules that contributed most to MOFA Factor 2, which denotes differences in their abundance profile in flesh samples between early and late stages, is presented in Figure 8. While inference of biological pathways from tomato proteins is limited to known, annotated tomato genes in the databases, our pathway enrichment analysis of the top 100 proteins detected in factor 2 suggested their involvement in photosynthesis-related processes. The next factors (3 to 10) captured different types of variations, for example, the segregation of information in 3 w and LOR from all other stages (factors 4 and 6), or the distinction of the last stage (RR) in factor 5 (Figure 7D). Thus, the top 100 proteins associated with Factor 3 revealed a significant enrichment in oxylipin, sterol, and steroid metabolism. The integration of such proteins with the top lipids associated with the same Factor 3 provides an opportunity to identify potential associations between these pathways and these lipids. Interestingly, the top proteins associated with Factor 4 were enriched in amino acid metabolism pathways, suggesting potential coordination with the top polar metabolites associated with the same factor (including 13 identified amino acids). These cross-omics associations were not apparent from the pairwise differential analyses presented above, because the new latent factors do not overlap with the specific ripening-stage comparison chosen in a supervised manner. In addition, the multi-omics framework allows molecules from multiple omics layers to be associated with the same latent factor. We provide the heatmaps of the top 30 features ranked by absolute MOFA weights across all factors and omics in Supporting File S2.

**Figure 7:**
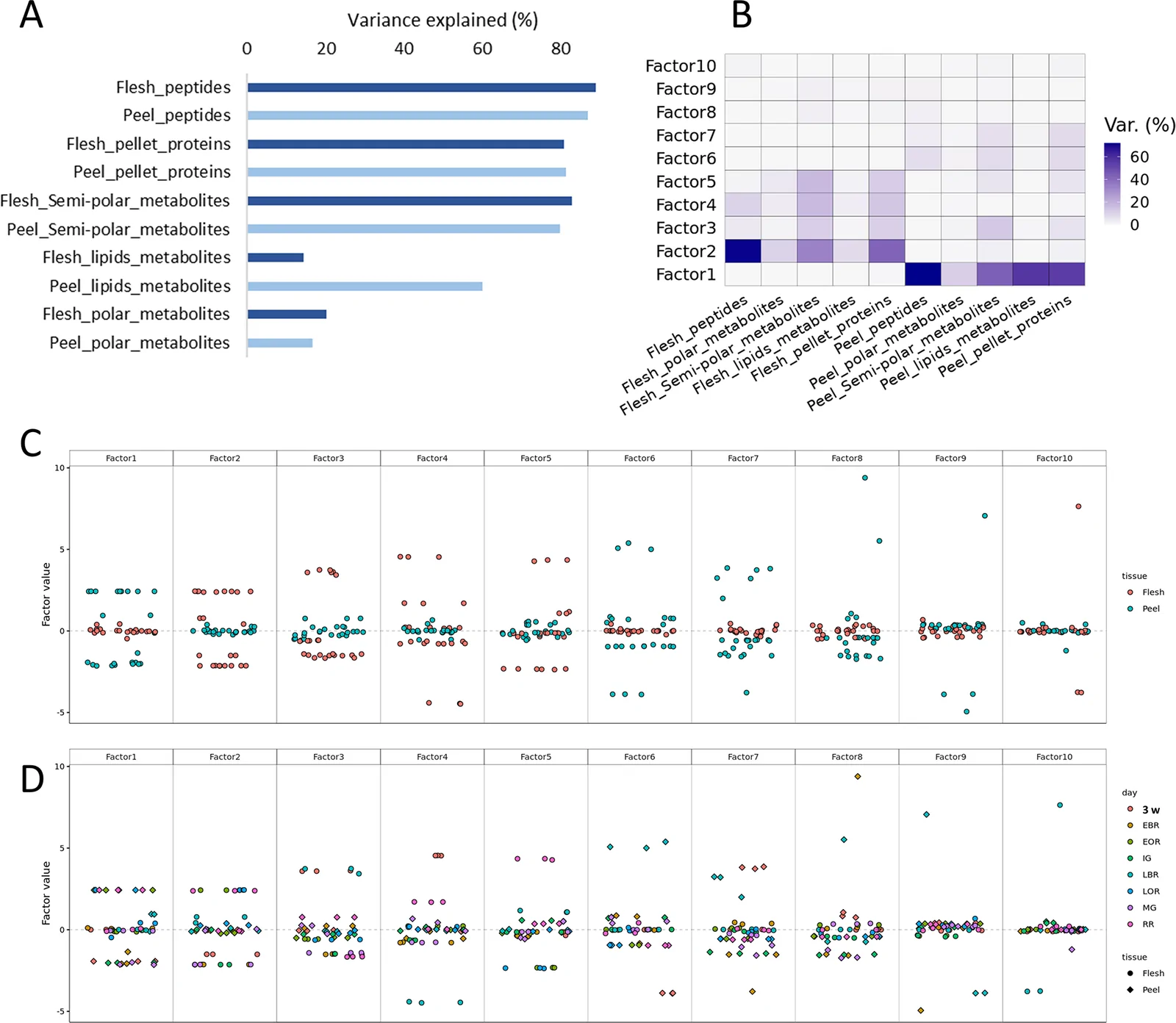
Multi-omics integration of all modalities across developmental stages in flesh and peel, using Multi-Omics Factor Analysis (MOFA). (A) Total variance explained by MOFA across omics. (B) Variance decomposition of the top 10 factors per omics. (C) Values of the top 10 factors; each dot corresponds to a sample, colored by the tissue type or by (D) developmental stage, and shapes denote tissue. 3 w, 3 weeks post-anthesis; IG, immature green; MG, mature green; EBR, early breaker; LBR, late breaker; EOR, early orange; LOR, late orange; RR, red ripe.

**Figure 8:**
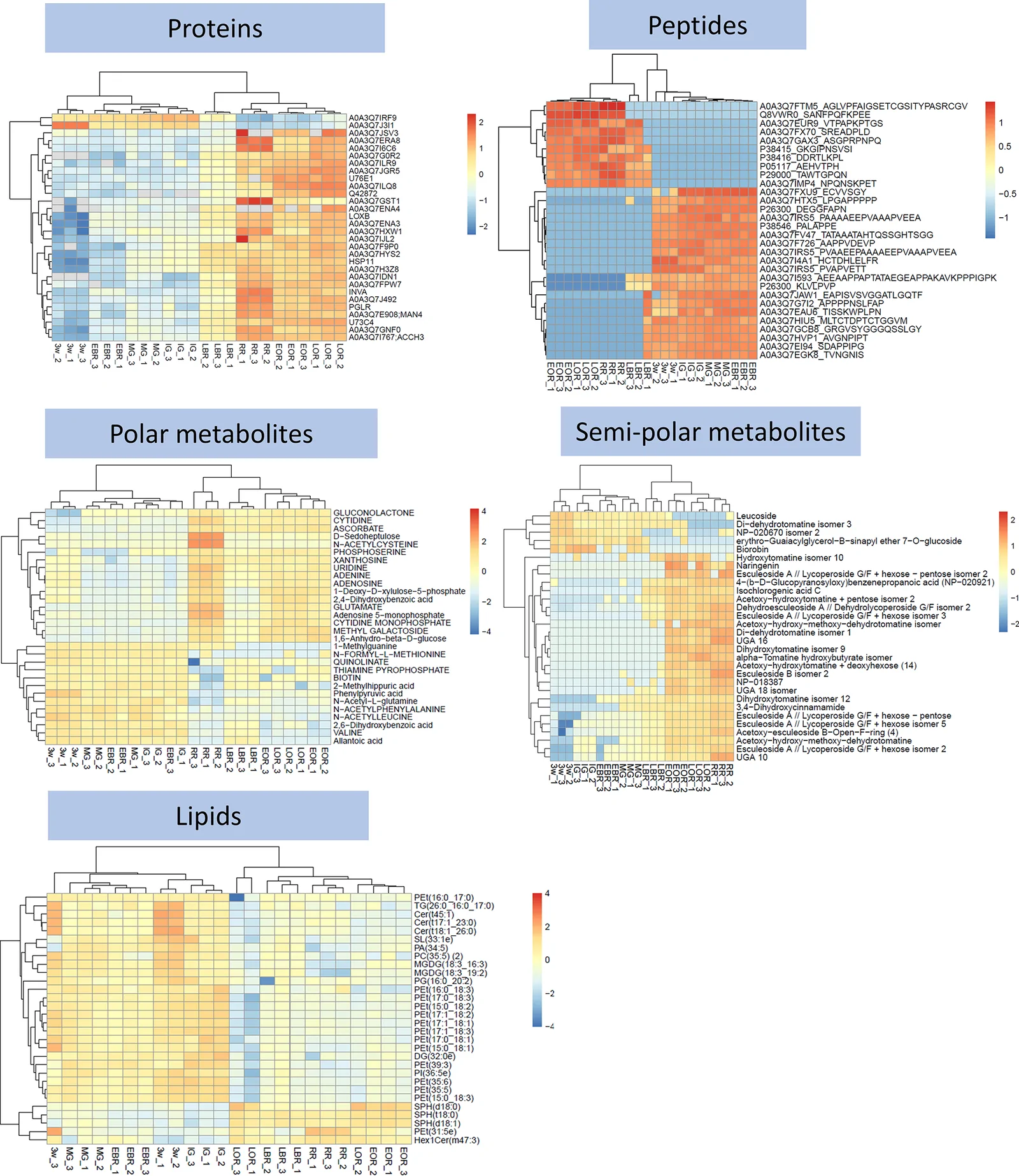
Heatmaps of the abundance of the top 30 biomolecules associated with MOFA factor 2 in the different omics across all flesh samples (from MOFA factor 2 shown in Figure 7B). Heatmap rows were scaled to reflect high (red) or low (blue) MOFA weights, and hierarchically clustered using Euclidean distance and complete linkage. Protein names or UniProt IDs are noted, together with their detected peptide sequences. 3 w, 3 weeks post-anthesis; IG, immature green; MG, mature green; EBR, early breaker; LBR, late breaker; EOR, early orange; LOR, late orange; RR, red ripe.

## Conclusions

The integrative approach presented here, spanning metabolomics, lipidomics, semi-polar metabolite profiling, peptidomics, and proteomics within a single extraction workflow, provides unique insight into the complex molecular reprogramming occurring during tomato fruit development and maturation. Furthermore, our unified dataset enables cross-layer correlative analyses, for example, between lipid class accumulation and the expression of associated biosynthetic enzymes, or between peptide abundance and metabolic shifts, although these relationships require future functional validation. In addition, this work provides a comprehensive resource for the community, including stage-resolved accumulation patterns and integrated multi-omics interpretation.

A key novelty of this work was the native peptide analysis, derived from the polar phase, which revealed a highly diverse set of peptides in both flesh and peel tissues. Peptide patterns separated intermediate developmental stages with high resolution and showed coordinated shifts across the time course. Analysis of peptide lengths and terminal amino acids indicated structured cleavage preferences that varied with development. Proteomics analysis of the pellet fraction revealed large-scale remodeling of protein abundance along the developmental trajectory. Joint consideration of proteins and peptides from the same samples allowed for a direct comparison of precursor protein levels with processed peptide products at scale. Although the reproducible developmental patterns, structured cleavage-site preferences, and the high quality of the corresponding bottom-up proteomic data argue against extensive nonspecific degradation during sample preparation, a limited contribution from residual ex vivo proteolysis cannot be completely excluded. Accordingly, the detected peptides should be considered predominantly endogenous, while acknowledging the possibility of minor preparation-induced cleavage. It should be noted that while we presented high coverage of the peptidome, not all endogenous peptides are detectable by mass spectrometry. The confounding factor is whether a peptide can be charged by ESI to a level that is above the limit of detection.

The observed peptide-length range of 7–65 amino acids partly reflects the peptide-length limits specified in the database search and should therefore not be interpreted as representing the complete endogenous peptidome. The observed distribution may also reflect extraction selectivity, because shorter and more hydrophilic peptides may preferentially remain in the polar fraction, whereas longer or more hydrophobic species may partition with the protein-containing pellet. The inclusion of non-peptide fractions broadened the chemical scope. Polar metabolite profiling encompassed central carbon and nitrogen pathways, tracking shifts relevant to energy balance and flavor formation. Semi-polar and pigment analyses quantified flavonoids, carotenoids, and chlorophyll-derived compounds that distinguish between early and late stages and align with fruit color development. Lipidomics identified pronounced remodeling of membrane and storage lipid classes, including phosphatidylcholines, galactolipids, and triacylglycerols, consistent with the transition from chloroplast to chromoplast and with peel-associated structural changes.

Across all layers, PCA separated samples by stage and tissue and showed tight clustering of biological replicates, supporting high reproducibility. Differential analysis yielded numerous significant features per omics layer between adjacent stages, with the peptidome yielding the most extensive set and providing high temporal resolution. Data integration was performed using MOFA, including all omics layers from peel and flesh. The model captured a high proportion of variance and resolved early versus late stages without relying on predefined contrasts. Loadings identified the features driving each latent factor and provided a compact representation of shared and layer-specific variation.

It should be noted that while the analytical platforms used in this study represent state-of-the-art instrumentation, and given that the starting material is not limiting, the same approach could be implemented using earlier-generation instrumentation. In fact, we performed the initial method optimization using instruments like the Q Exactive HF (Thermo) and still achieved substantial molecular coverage (data not shown). Because reduced configurations were not experimentally benchmarked, we cannot define a universally essential subset of analytical modules. The workflow is modular, and laboratories using one high-resolution mass spectrometer may select the omics layers most relevant to their biological question and run the corresponding LC-MS methods sequentially. For example, pigment analysis may be omitted when pigmentation is not a primary objective, whereas dedicated peptidomics should be retained when endogenous peptide biology is central to the study. In summary, this workflow enables high-throughput, quantitative recovery of peptides, proteins, polar and semi-polar metabolites, lipids, and pigments from a single small tissue sample. The resulting dataset defines stage-resolved molecular programs in tomato fruit across tissues and chemistries and provides directly comparable measurements across omics. By generating all omics layers from the same extraction and biological specimen, the workflow may reduce extraction-related variability between independently processed aliquots and maximize the molecular information obtained from each sample. Although these potential advantages were not directly evaluated in the present study, the workflow may be adaptable to other plant tissues and may also prove valuable for sample-limited materials. Together, the data and workflow provide a practical template for multi-omics investigations of plant development, with standardized acquisition and integrative analysis. This platform may facilitate biomarker discovery relevant to the food industry and may also be applicable to diverse biological tissues and biofluids.

## Supplementary Material





## Data Availability

The mass spectrometry peptidomics and proteomics data have been deposited to the ProteomeXchange Consortium via the PRIDE (https://www.ebi.ac.uk/pride/) partner repository under the dataset identifiers PXD075865 and PXD075890, respectively.

## Notes

Raw detected measurements in all omics (Supporting File S1) (XLSX)
Differential accumulation of peel biomolecules across ripening stages (Figure S1); Volcano plots of differentially accumulated features detected between consecutive ripening stages (Figure S2); frequency of amino acids (Figure S3); enriched GO biological processes (Figure S4); and MOFA heatmaps (Supporting File S2) (PDF)

## Abbreviations

MTBE: methyl *tert*-butyl ether
HILIC: hydrophilic interaction liquid chromatography
FA: formic acid
HDMSE: high-definition data-independent acquisition mass spectrometry
DDW: double-distilled water
ACN: acetonitrile
IPA: isopropanol
UPLC: ultra-performance liquid chromatography
PCA: principal component analysis
MOFA: multi-omics factor analysis
ANOVA: analysis of variance
FDR: false discovery rate
DPA: days post-anthesis
3 w: 3 weeks post-anthesis
IG: immature green
MG: mature green
EBR: early breaker
LBR: late breaker
EOR: early orange
LOR: late orange
RR: red ripe
