# Supplementary material for: Single-Tube Mass Spectrometry-Based Workflow for Multi-Omics Profiling of Diverse Biomolecules
Source: Anal Chem. 2026 Sep 14;98(38):28106–21. doi: 10.1021/acs.analchem.6c03036 (PMC13629164; doi:10.1021/acs.analchem.6c03036)
Supplement: Supplementary file 1 [file ac6c03036_si_002.pdf]

Supporting Information

Single-Tube Mass Spectrometry-Based Workflow for Multi-Omics Profiling of Diverse Biomolecules

Maxim Itkin[1]‡, Bareket Dassa[1]‡, Yishai Levin[1,3], Andrii Vainer[2], Asaph Aharoni[2], Sergey Malitsky[1]*

[1] Life Sciences Core Facilities, Weizmann Institute of Science, Rehovot 7610001, Israel

[2] Department of Plant and Environmental Sciences, Weizmann Institute of Science, Rehovot 7610001, Israel

[3] de Botton Institute for Protein Profiling, Israel National Center for Personalized Medicine, Weizmann Institute of Science, Rehovot 7610001, Israel

‡ These authors contributed equally

- Corresponding author, sergey.malitsky@weizmann.ac.il

Table of Contents:

Supplementary Figure S1 ……………….. 2

Supplementary Figure S2 ……………….. 3

Supplementary Figure S3 ……………….. 6

Supplementary Figure S4 ……………….. 7

Supplementary File S1 ……………….. 8

Supplementary File S2 ……………….. 9

Figure S1: Differential accumulation of peel biomolecules across ripening stages. Differentially accumulated biomolecules were detected between consecutive fruit developmental stages in each omics dataset (FDR cutoff of ≤0.05). The heatmap shows the relative abundance of significant differential molecules across samples in each omics. Raw measured values were scaled and displayed as colors ranging from red to blue, as shown in the key; missing values were imputed, and rows were hierarchically clustered. A similar analysis of the flesh tissue is shown in Figure 4. Corresponding volcano plots of differential molecules are shown in Figure S2. Met., metabolites; DE, differentially expressed; DA, differentially accumulated; 3 w, 3 weeks post-anthesis; IG, immature green; MG, mature green; EBR, early breaker; LBR, late breaker; EOR, early orange; LOR, late orange; RR, red ripe.

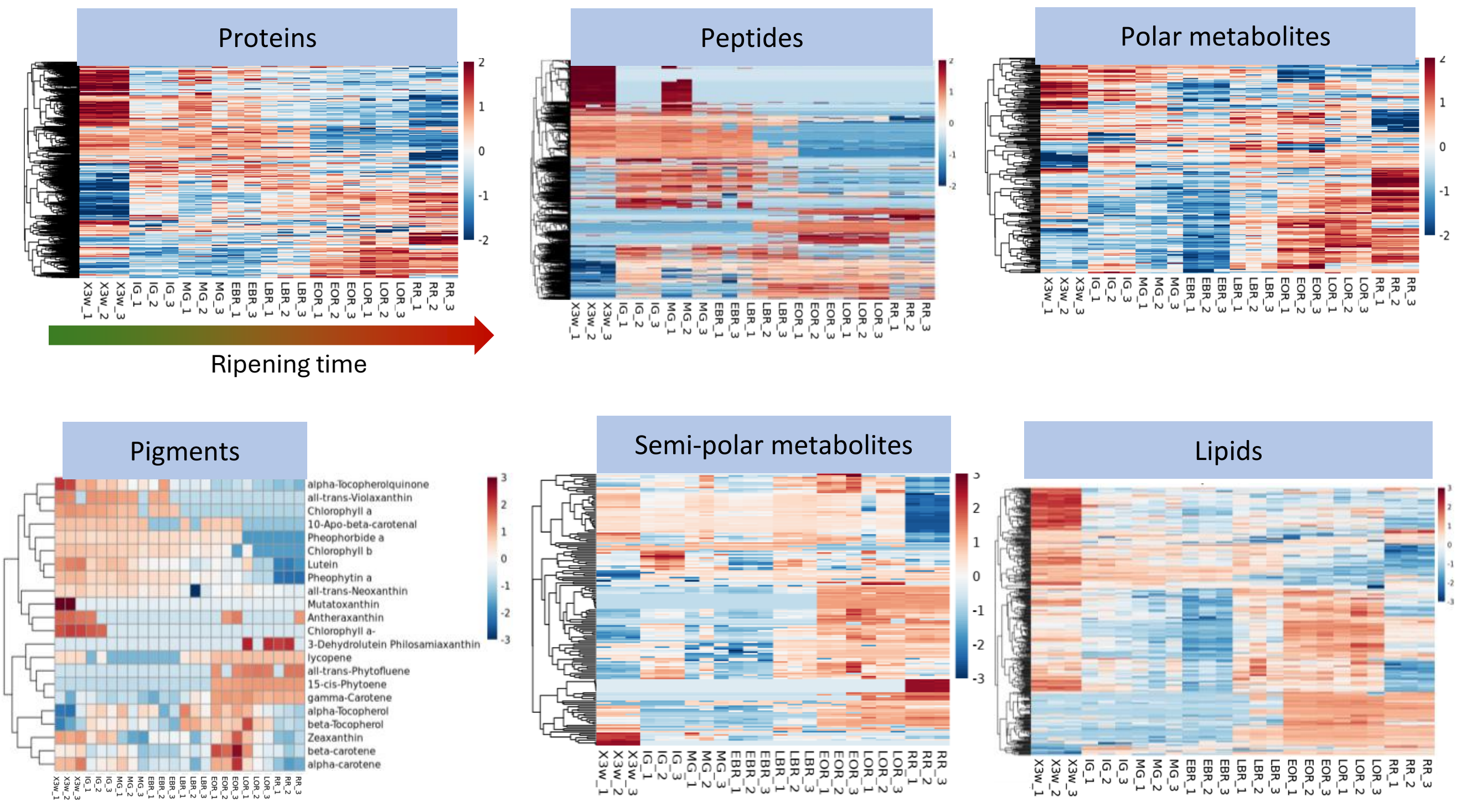

Figure S2: Volcano plots of differentially accumulated features detected between consecutive ripening stages for (**A**) native peptides, (**B**) proteins, (**C**) polar metabolites, in flesh or peel samples. 3 w, 3 weeks post-anthesis; IG, immature green; MG, mature green;EBR, early breaker; LBR, late breaker; EOR, early orange; LOR, late orange; RR, red ripe.

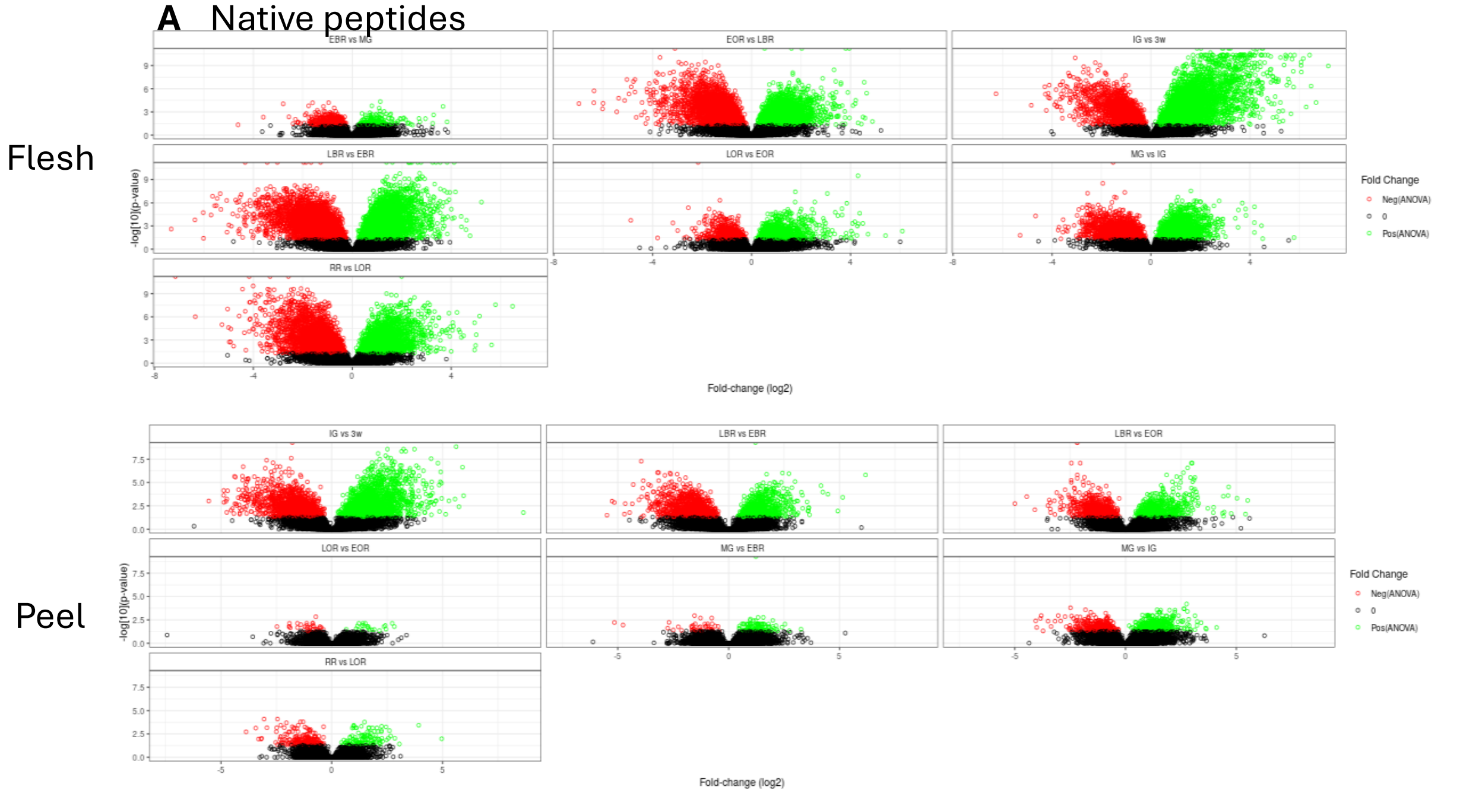

B Proteins

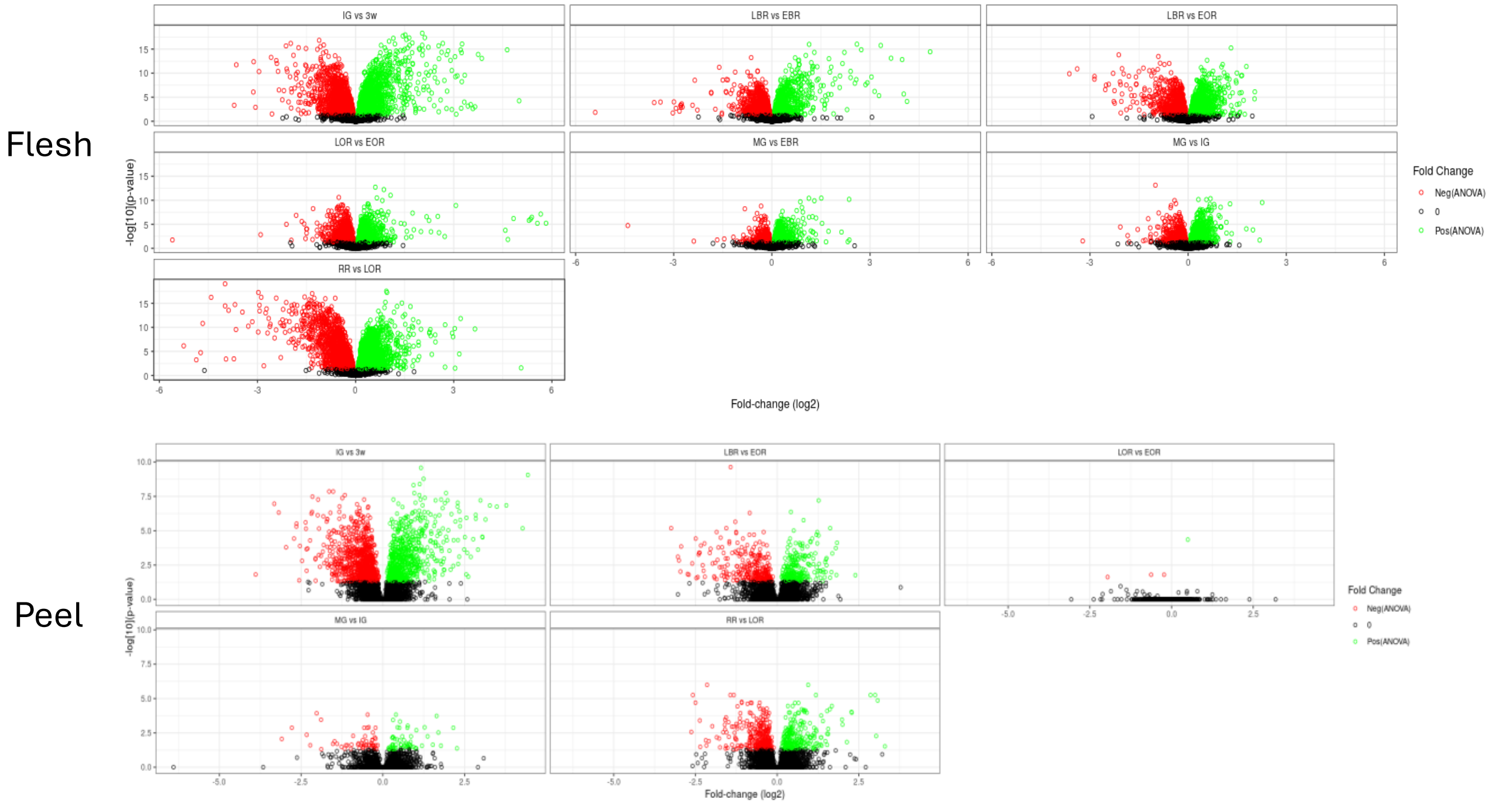

Flesh
IG vs 3w
LBR vs EBR
LBR vs EOR
LOR vs EOR
MG vs EBR
MG vs IG
RR vs LOR
-log[10](p-value)
Fold-change (log2)
Fold Change
Neg(ANOVA)
0
Pos(ANOVA)
Peel
IG vs 3w
LBR vs EOR
LOR vs EOR
MG vs IG
RR vs LOR
-log[10](p-value)
Fold-change (log2)
Fold Change
Neg(ANOVA)
0
Pos(ANOVA)

C Polar metabolites

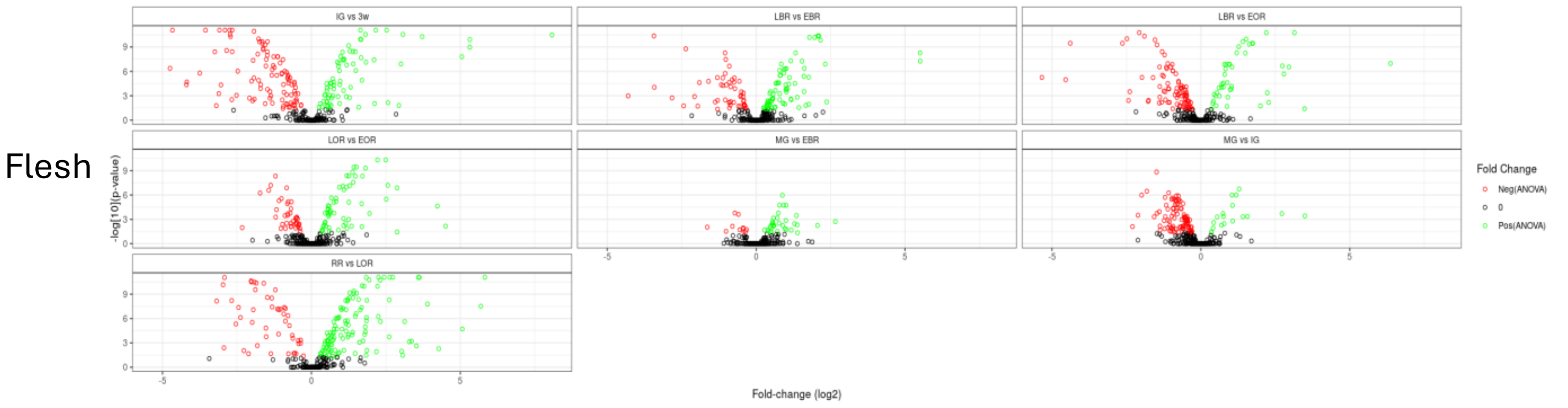

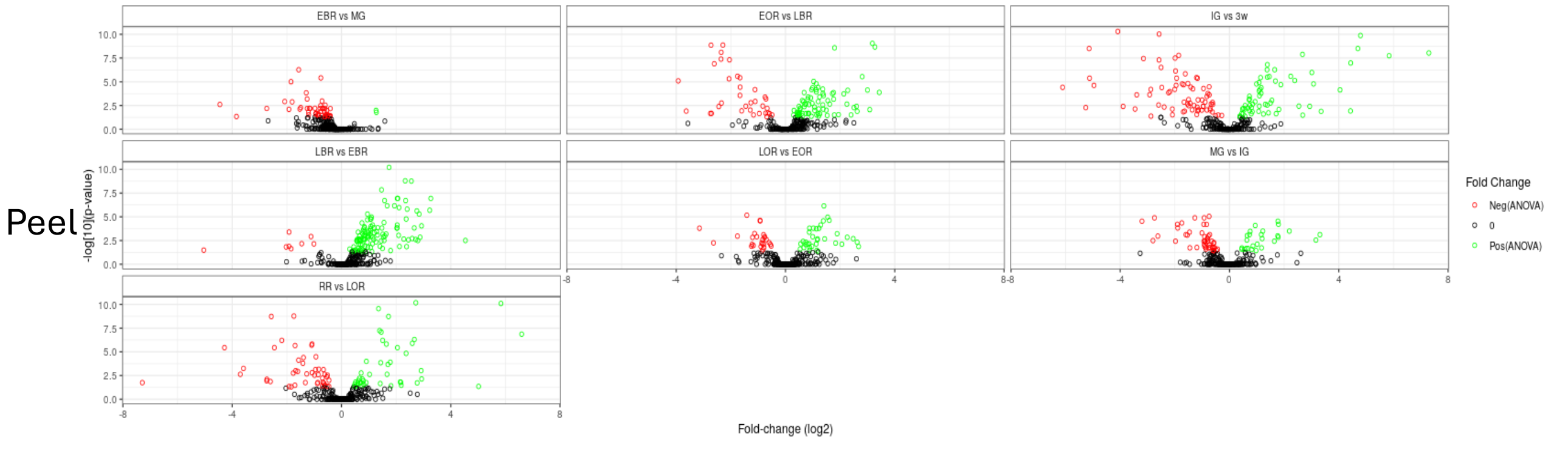

Figure S3: (A) Frequency of amino acids in the entire tomato proteome **(B)** Comparison of the proportions of last amino acid in flesh peptides with the whole proteome

A **B**

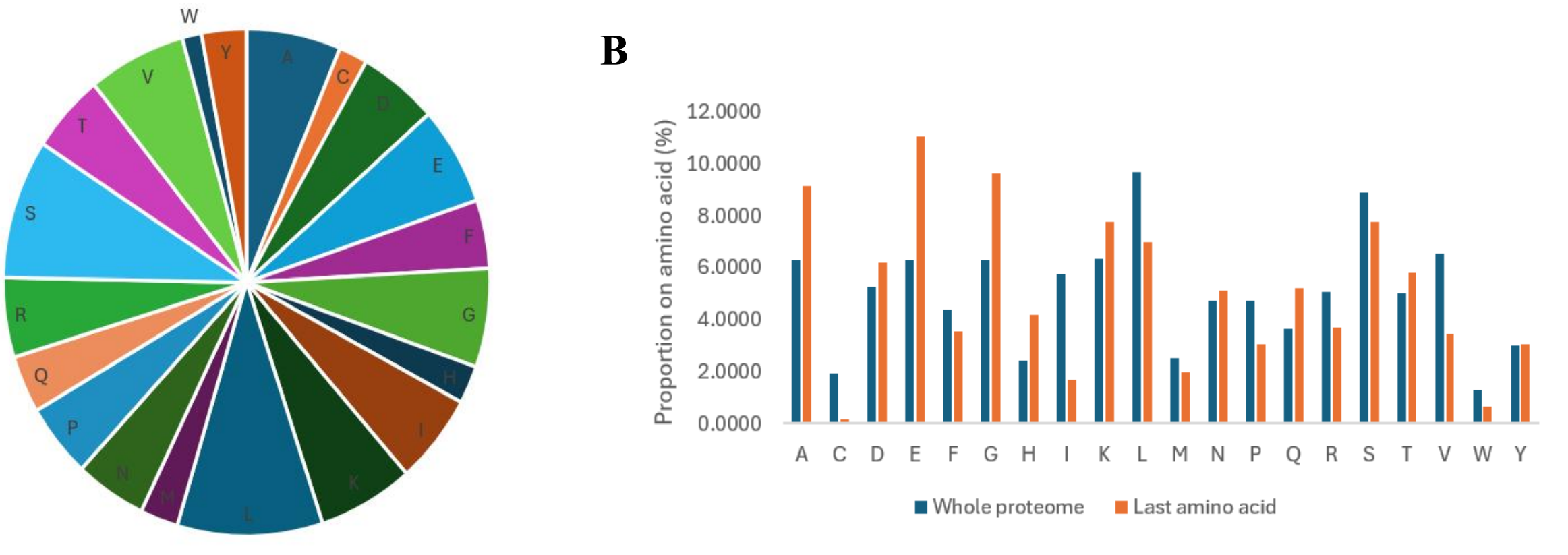

Figure S4: Enriched GO biological processes (with FDR ≤0.05) detected in the top 100 proteins associated with each MOFA latent factor that captured variance in the flesh samples. Functional enrichment analysis was applied with ShinyGO, using all MOFA input proteins as the background and the *Solanum lycopersicum* genes SL3.0 as the reference.

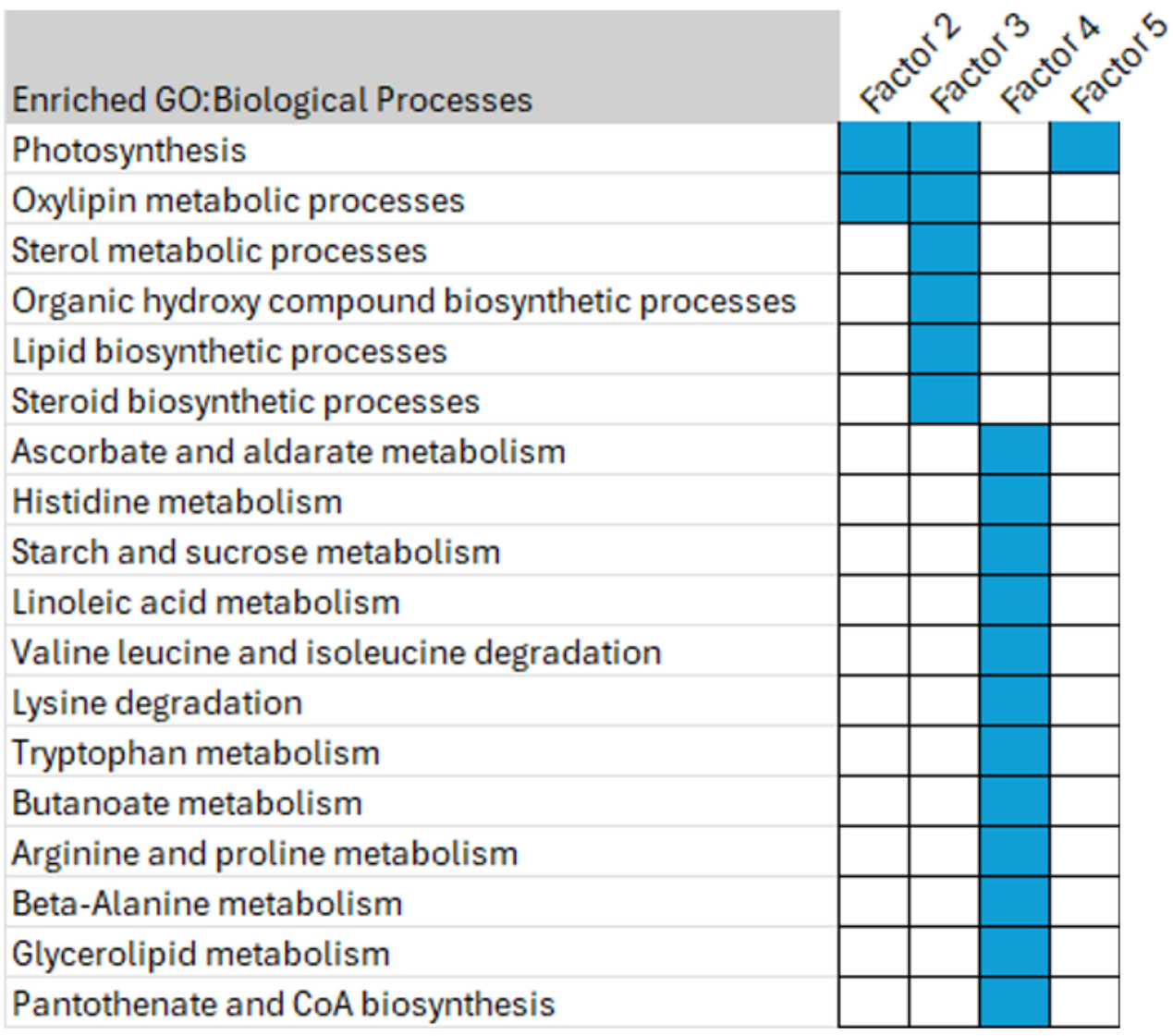

Supporting File S1: Raw detected measurements in all omics, including polar metabolites, semi-polar metabolites, lipids, pigments, proteins, and native peptides, for either flesh or peel samples. 3 w, 3 weeks post-anthesis; IG, immature green; MG, mature green; EBR, early breaker; LBR, late breaker; EOR, early orange; LOR, late orange; RR, red ripe; UGA,unidentified glycoalkaloid.

(Provided as an Excel file)

Supporting File S2: MOFA heatmaps of weights for the top 30 molecules detected in each MOFA factor in each omics type (presented without denoising). Heatmap rows were scaled to reflect high (red) or low (blue) weights, and hierarchically clustered. 3 w, 3 weeks post-anthesis; IG, immature green; MG, mature green; EBR, early breaker; LBR, late breaker; EOR,early orange; LOR, late orange; RR, red ripe.

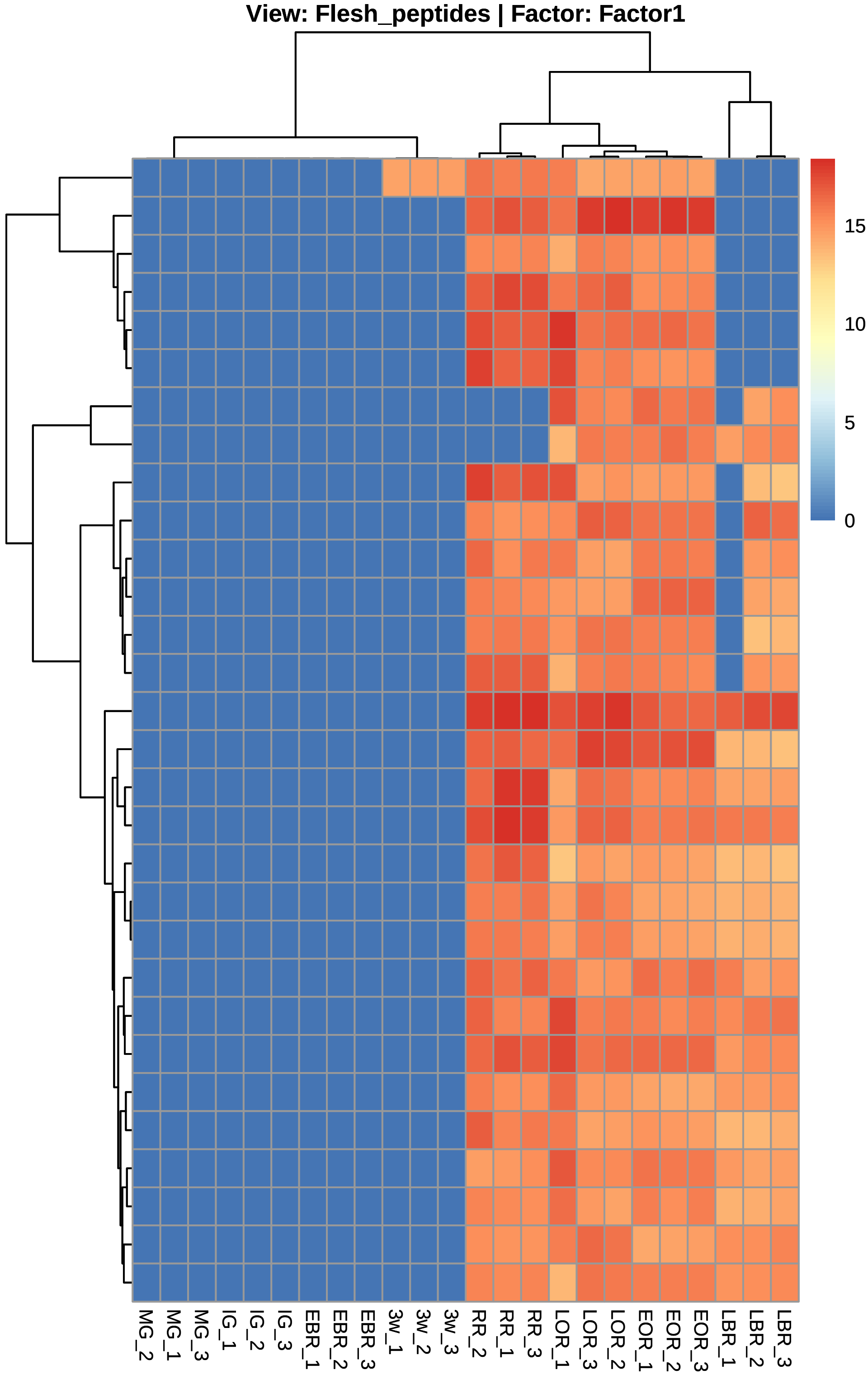
View: Flesh_peptides | Factor: Factor1
15
10
5
0
MG_2
MG_1
MG_3
IG_1
IG_2
IG_3
EBR_1
EBR_2
EBR_3
3w_1
3w_2
3w_3
RR_2
RR_1
RR_3
LOR_1
LOR_3
LOR_2
EOR_1
EOR_2
EOR_3
LBR_1
LBR_2
LBR_3

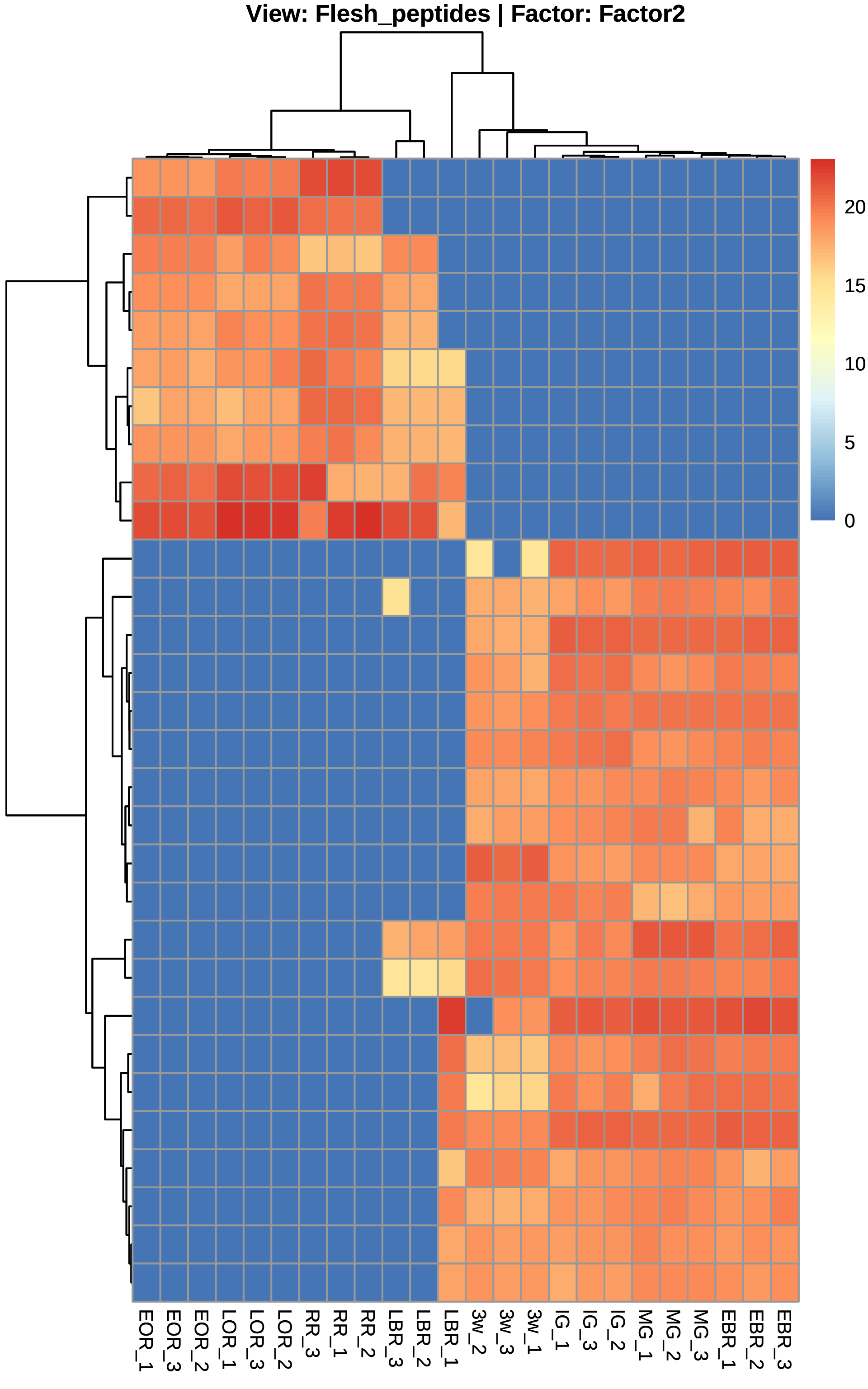
View: Flesh_peptides | Factor: Factor2
20
15
10
5
0
EOR_1
EOR_3
EOR_2
LOR_1
LOR_3
LOR_2
RR_3
RR_1
RR_2
LBR_3
LBR_2
LBR_1
3w_2
3w_3
3w_1
IG_1
IG_3
IG_2
MG_1
MG_2
MG_3
EBR_1
EBR_2
EBR_3

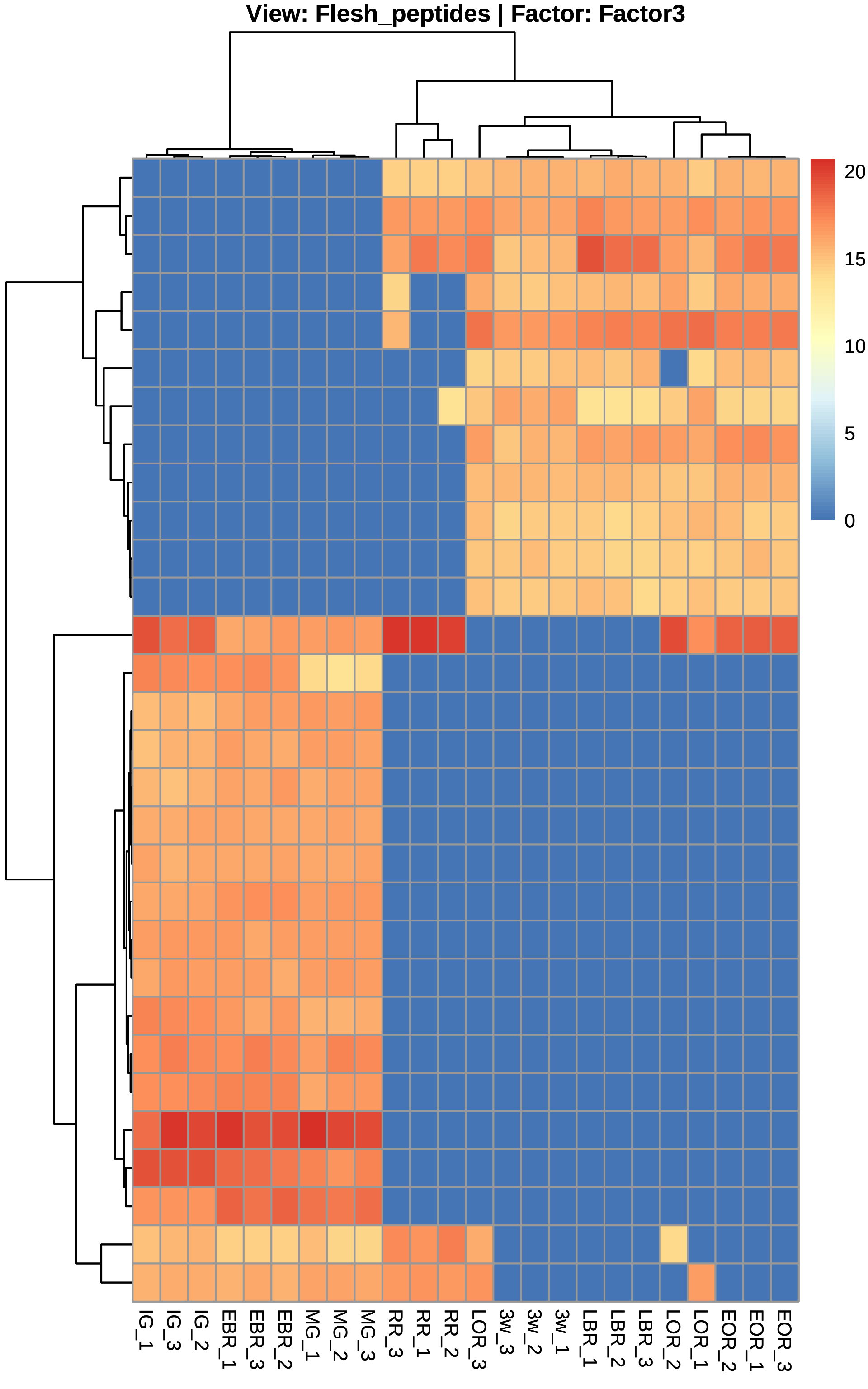
View: Flesh_peptides | Factor: Factor3
20
15
10
5
0
IG_1
IG_3
IG_2
EBR_1
EBR_3
EBR_2
MG_1
MG_2
MG_3
RR_3
RR_1
RR_2
LOR_3
3w_3
3w_2
3w_1
LBR_1
LBR_2
LBR_3
LOR_2
LOR_1
EOR_2
EOR_1
EOR_3

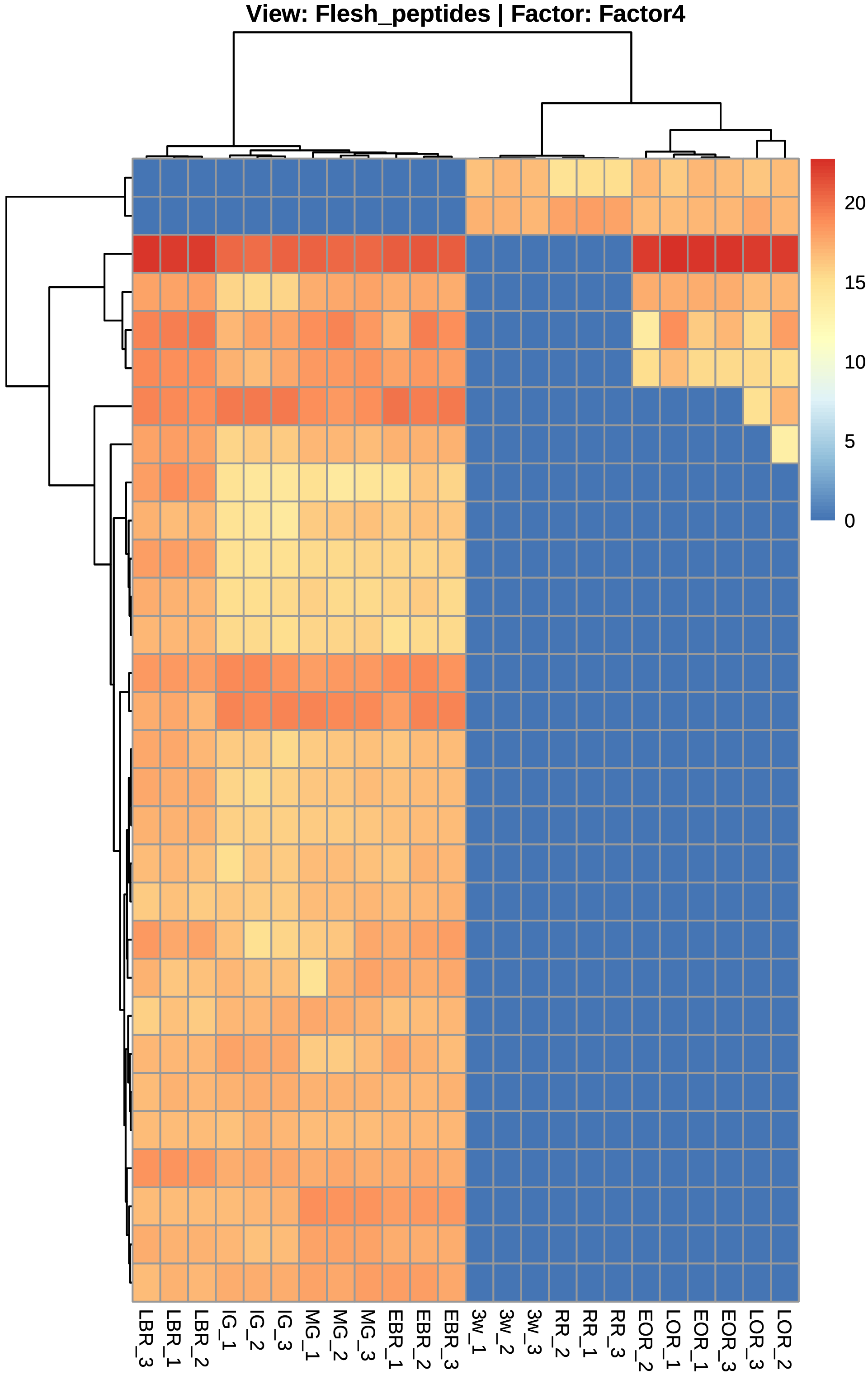

View: Flesh_peptides | Factor: Factor4
20
15
10
5
0
LBR_3
LBR_1
LBR_2
IG_1
IG_2
IG_3
MG_1
MG_2
MG_3
EBR_1
EBR_2
EBR_3
3w_1
3w_2
3w_3
RR_2
RR_1
RR_3
EOR_2
LOR_1
EOR_1
EOR_3
LOR_3
LOR_2

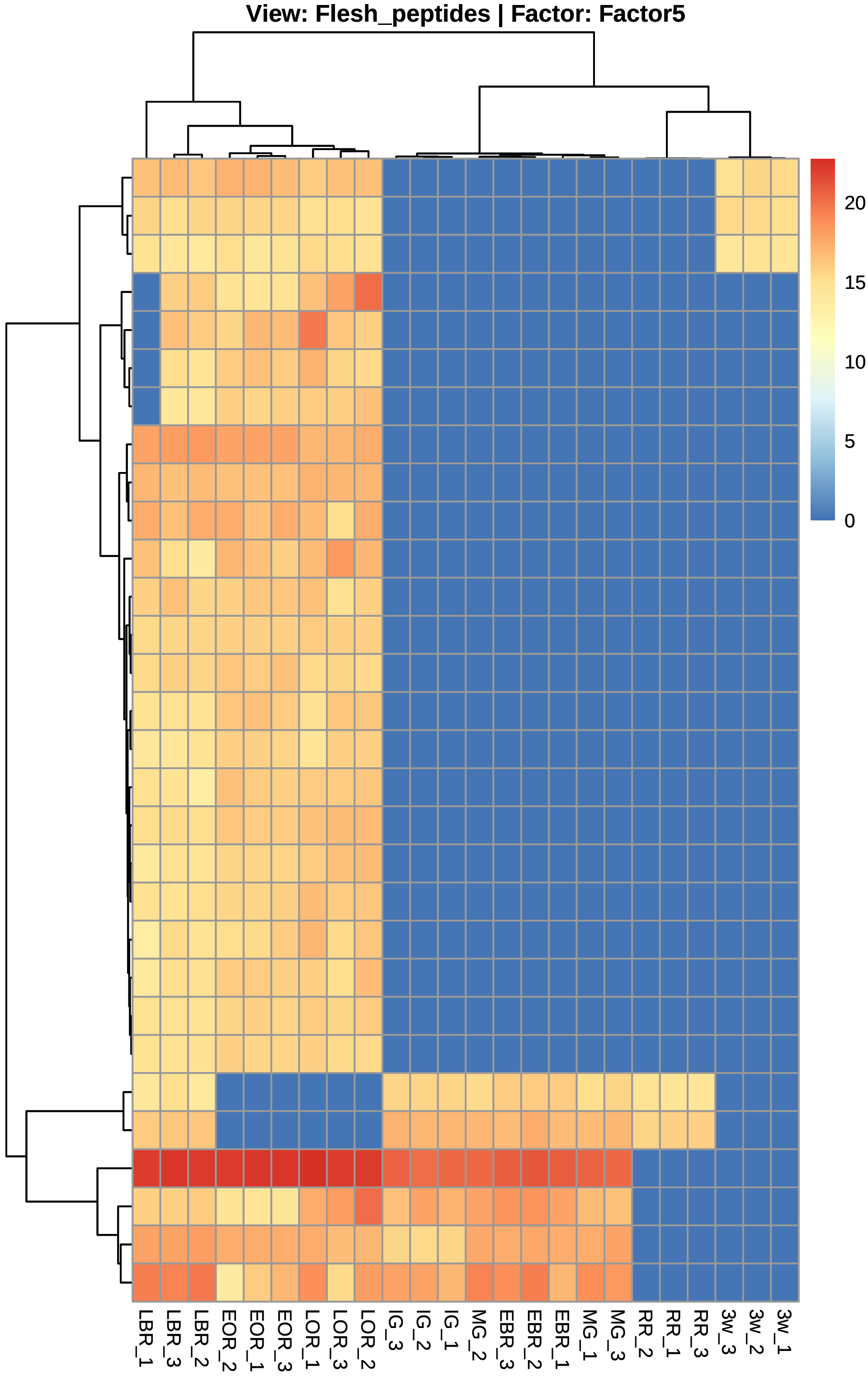
View: Flesh_peptides | Factor: Factor5
20
15
10
5
0
LBR_1
LBR_3
LBR_2
EOR_2
EOR_1
EOR_3
LOR_1
LOR_3
LOR_2
IG_3
IG_2
IG_1
MG_2
EBR_3
EBR_2
EBR_1
MG_1
MG_3
RR_2
RR_1
RR_3
3w_3
3w_2
3w_1

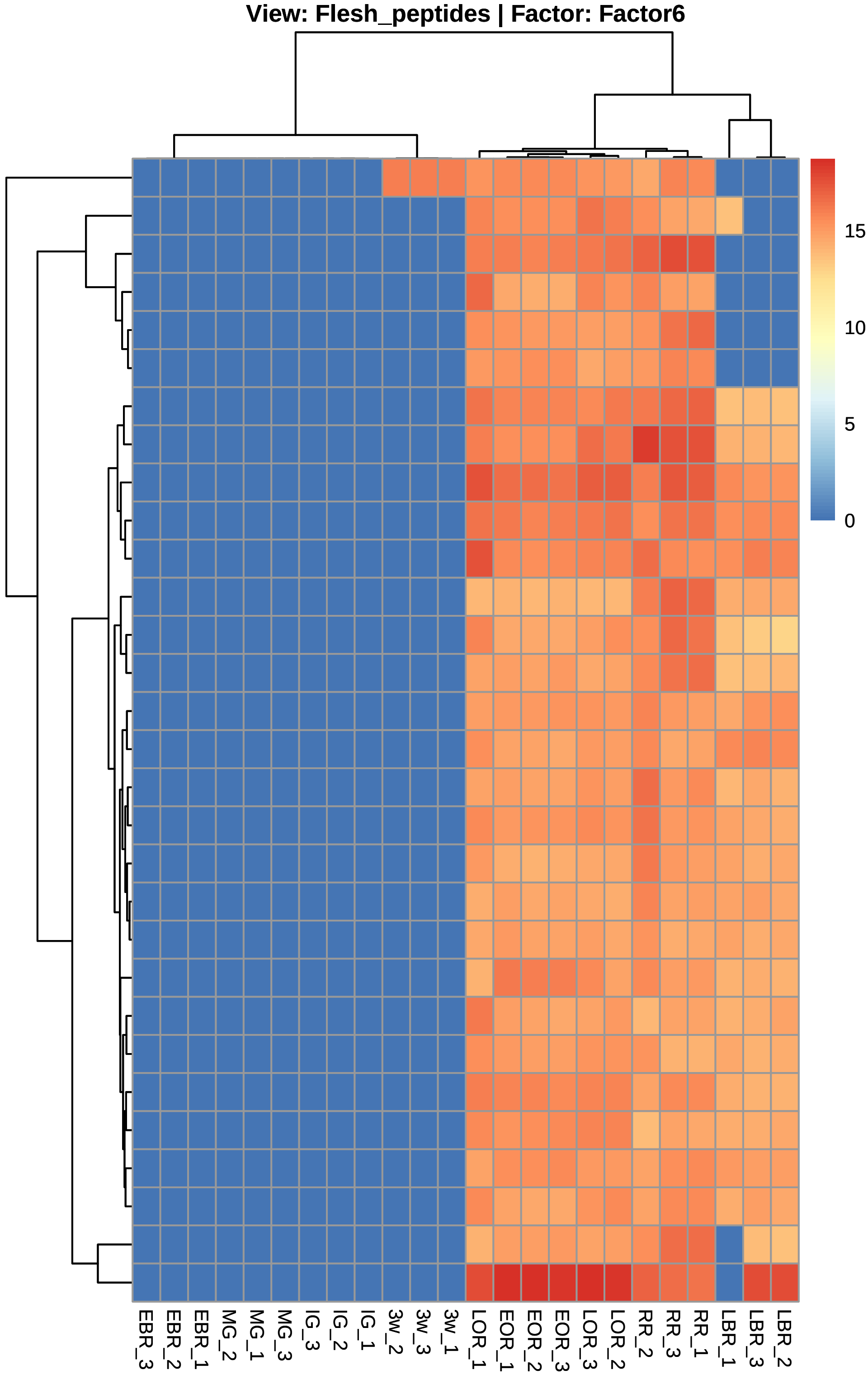
View: Flesh_peptides | Factor: Factor6
15
10
5
0
EBR_3
EBR_2
EBR_1
MG_2
MG_1
MG_3
IG_3
IG_2
IG_1
3w_2
3w_3
3w_1
LOR_1
EOR_1
EOR_2
EOR_3
LOR_3
LOR_2
RR_2
RR_3
RR_1
LBR_1
LBR_3
LBR_2

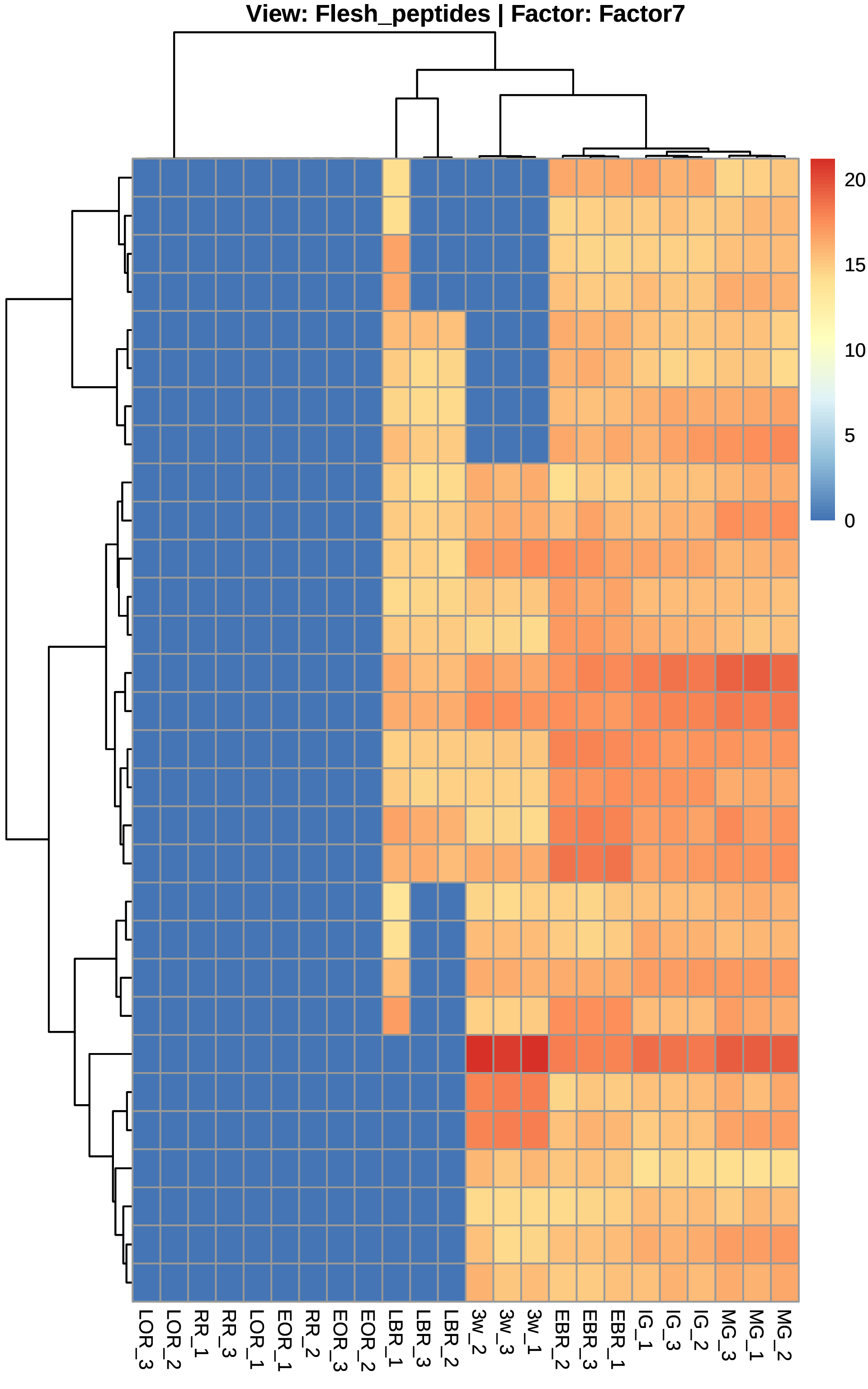

View: Flesh_peptides | Factor: Factor7
20
15
10
5
0
LOR_3
LOR_2
RR_1
RR_3
LOR_1
EOR_1
RR_2
EOR_3
EOR_2
LBR_1
LBR_3
LBR_2
3w_2
3w_3
3w_1
EBR_2
EBR_3
EBR_1
IG_1
IG_3
IG_2
MG_3
MG_1
MG_2

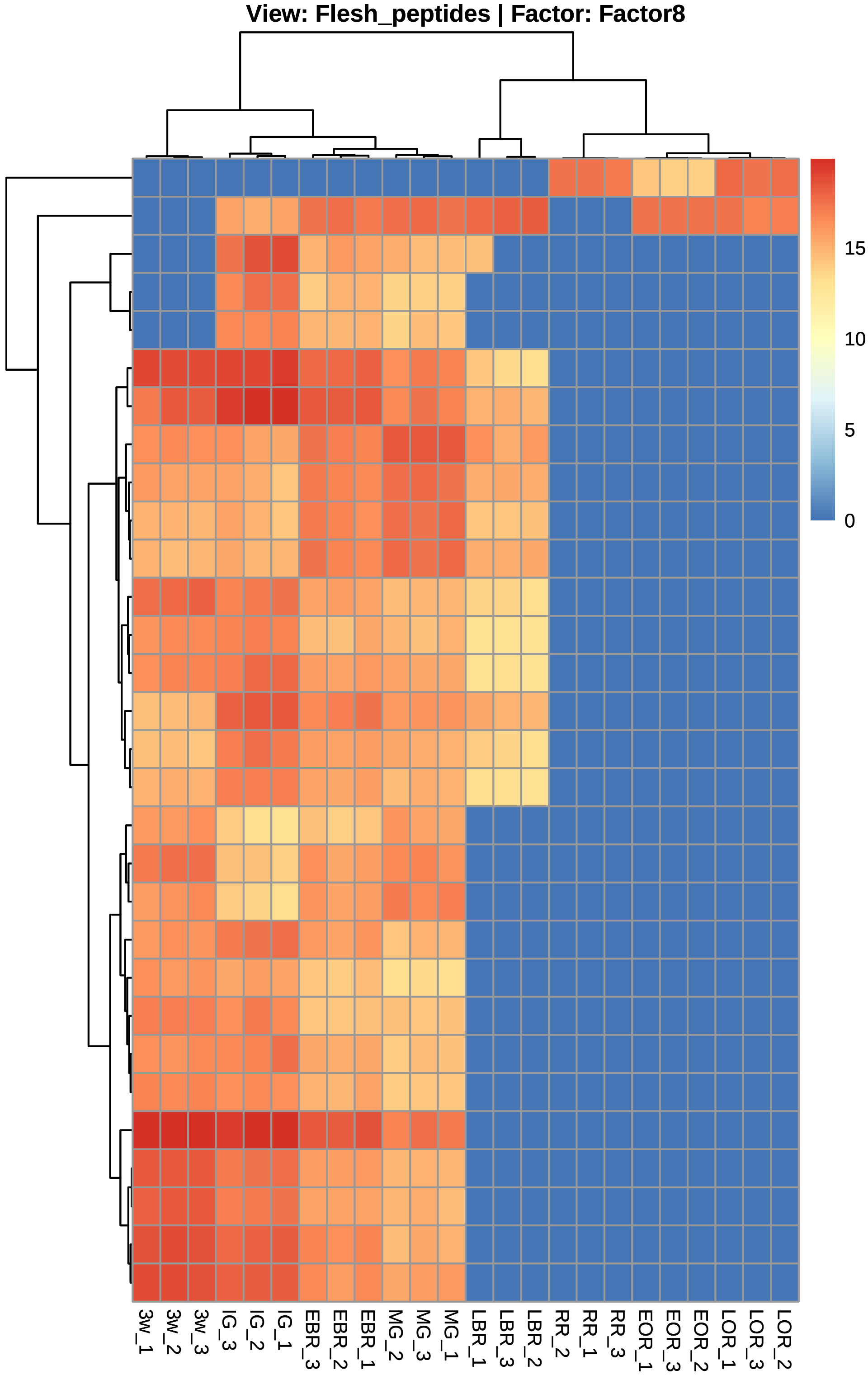
View: Flesh_peptides | Factor: Factor8
15
10
5
0
3w_1
3w_2
3w_3
IG_3
IG_2
IG_1
EBR_3
EBR_2
EBR_1
MG_2
MG_3
MG_1
LBR_1
LBR_3
LBR_2
RR_2
RR_1
RR_3
EOR_1
EOR_3
EOR_2
LOR_1
LOR_3
LOR_2

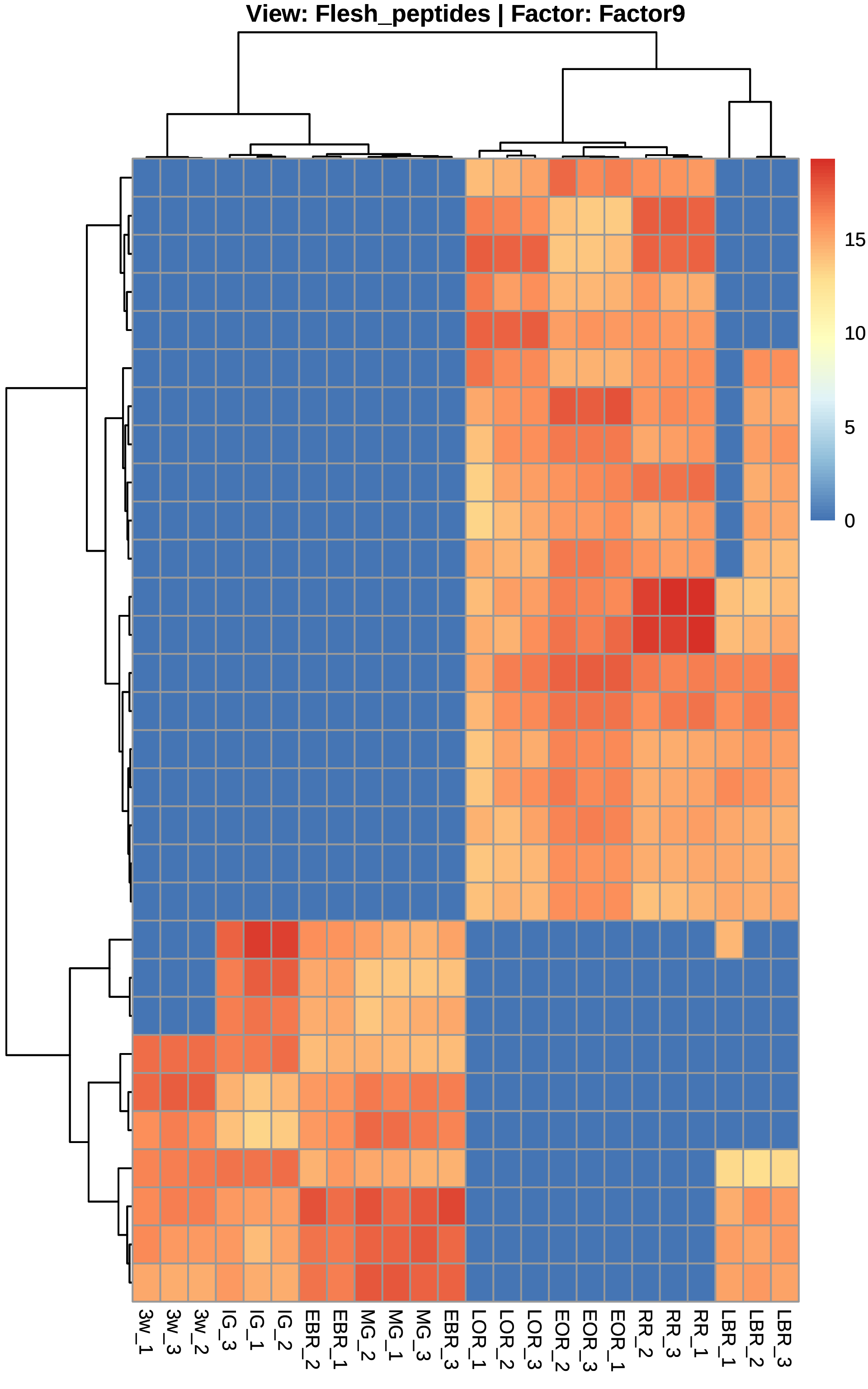
View: Flesh_peptides | Factor: Factor9
15
10
5
0
3w_1
3w_3
3w_2
IG_3
IG_1
IG_2
EBR_2
EBR_1
MG_2
MG_1
MG_3
EBR_3
LOR_1
LOR_2
LOR_3
EOR_2
EOR_3
EOR_1
RR_2
RR_3
RR_1
LBR_1
LBR_2
LBR_3

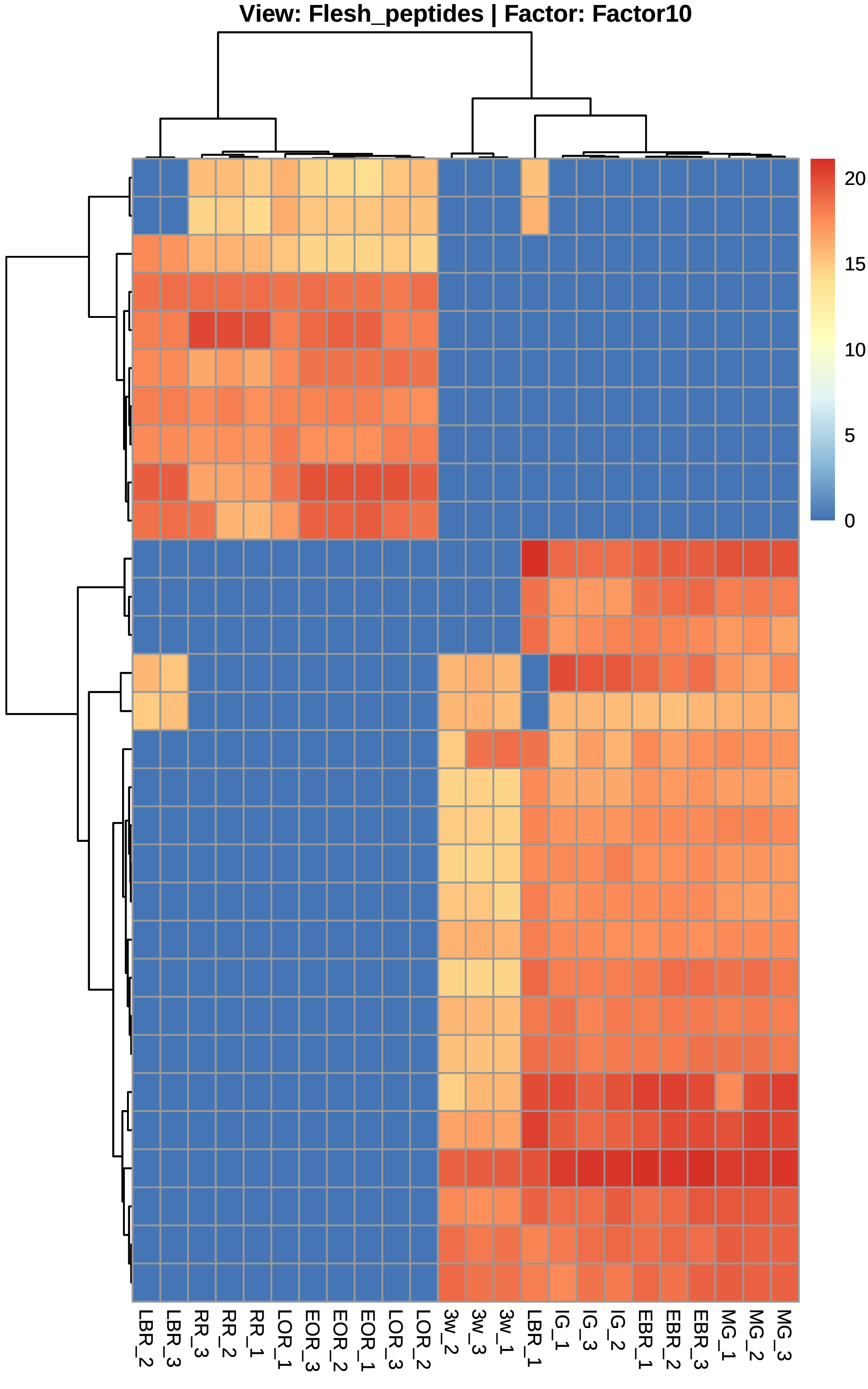

View: Flesh_peptides | Factor: Factor10
20
15
10
5
0
LBR_2
LBR_3
RR_3
RR_2
RR_1
LOR_1
EOR_3
EOR_2
EOR_1
LOR_3
LOR_2
3w_2
3w_3
3w_1
LBR_1
IG_1
IG_3
IG_2
EBR_1
EBR_2
EBR_3
MG_1
MG_2
MG_3

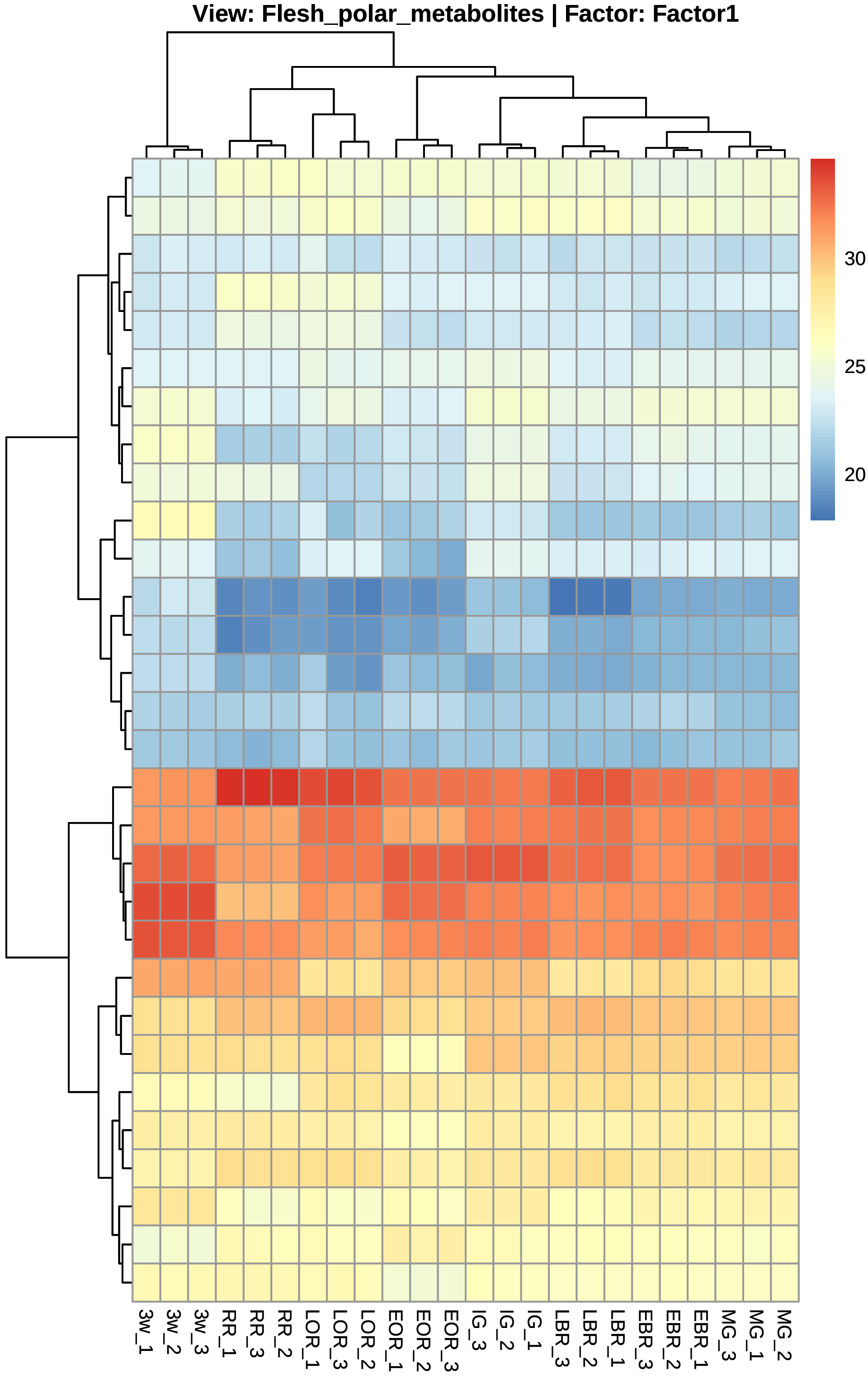

View: Flesh_polar_metabolites | Factor: Factor1
30
25
20
3w_1
3w_2
3w_3
RR_1
RR_3
RR_2
LOR_1
LOR_3
LOR_2
EOR_1
EOR_2
EOR_3
IG_3
IG_2
IG_1
LBR_3
LBR_2
LBR_1
EBR_3
EBR_2
EBR_1
MG_3
MG_1
MG_2

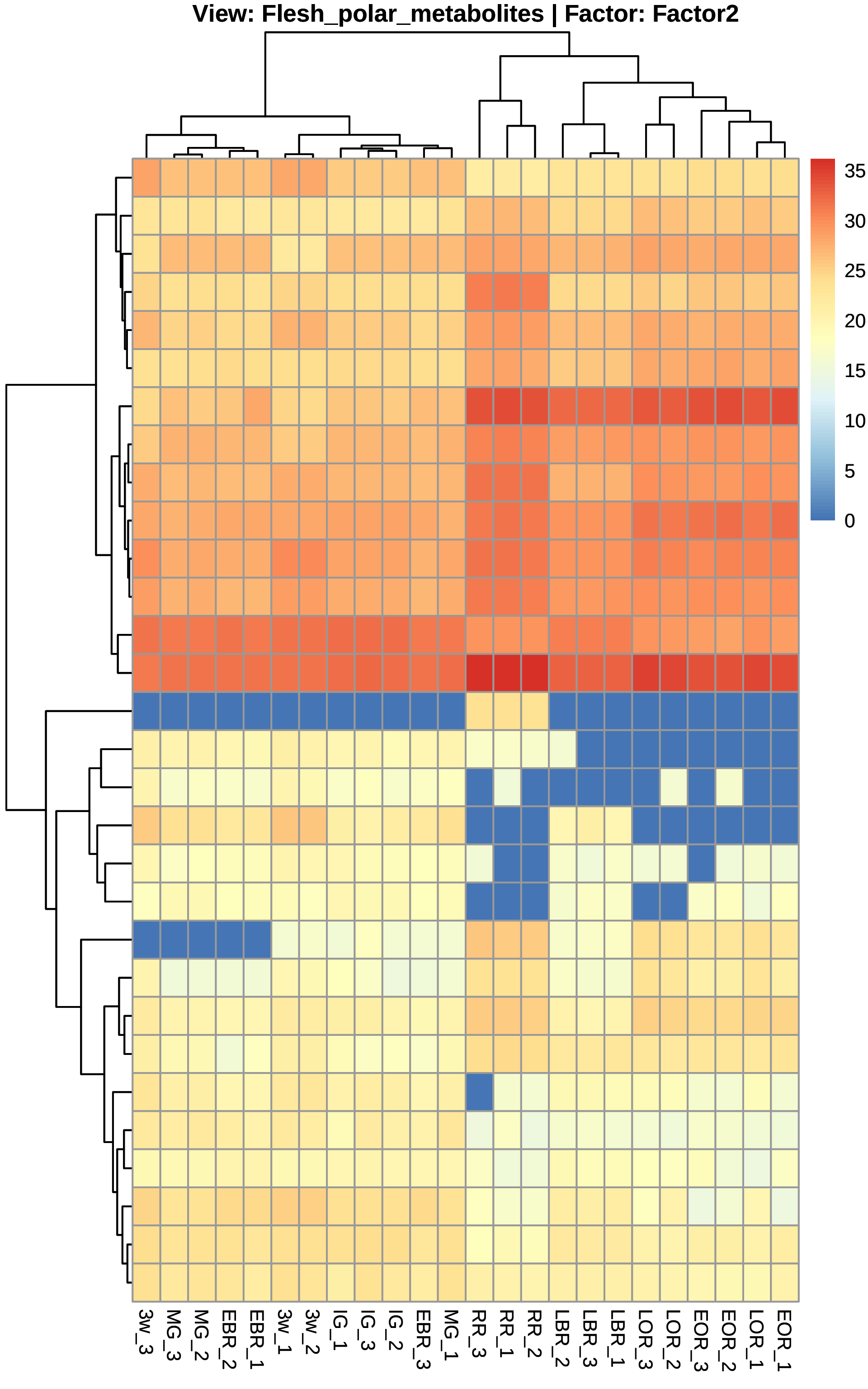
View: Flesh_polar_metabolites | Factor: Factor2
35
30
25
20
15
10
5
0
3w_3
MG_3
MG_2
EBR_2
EBR_1
3w_1
3w_2
IG_1
IG_3
IG_2
EBR_3
MG_1
RR_3
RR_1
RR_2
LBR_2
LBR_3
LBR_1
LOR_3
LOR_2
EOR_3
EOR_2
LOR_1
EOR_1

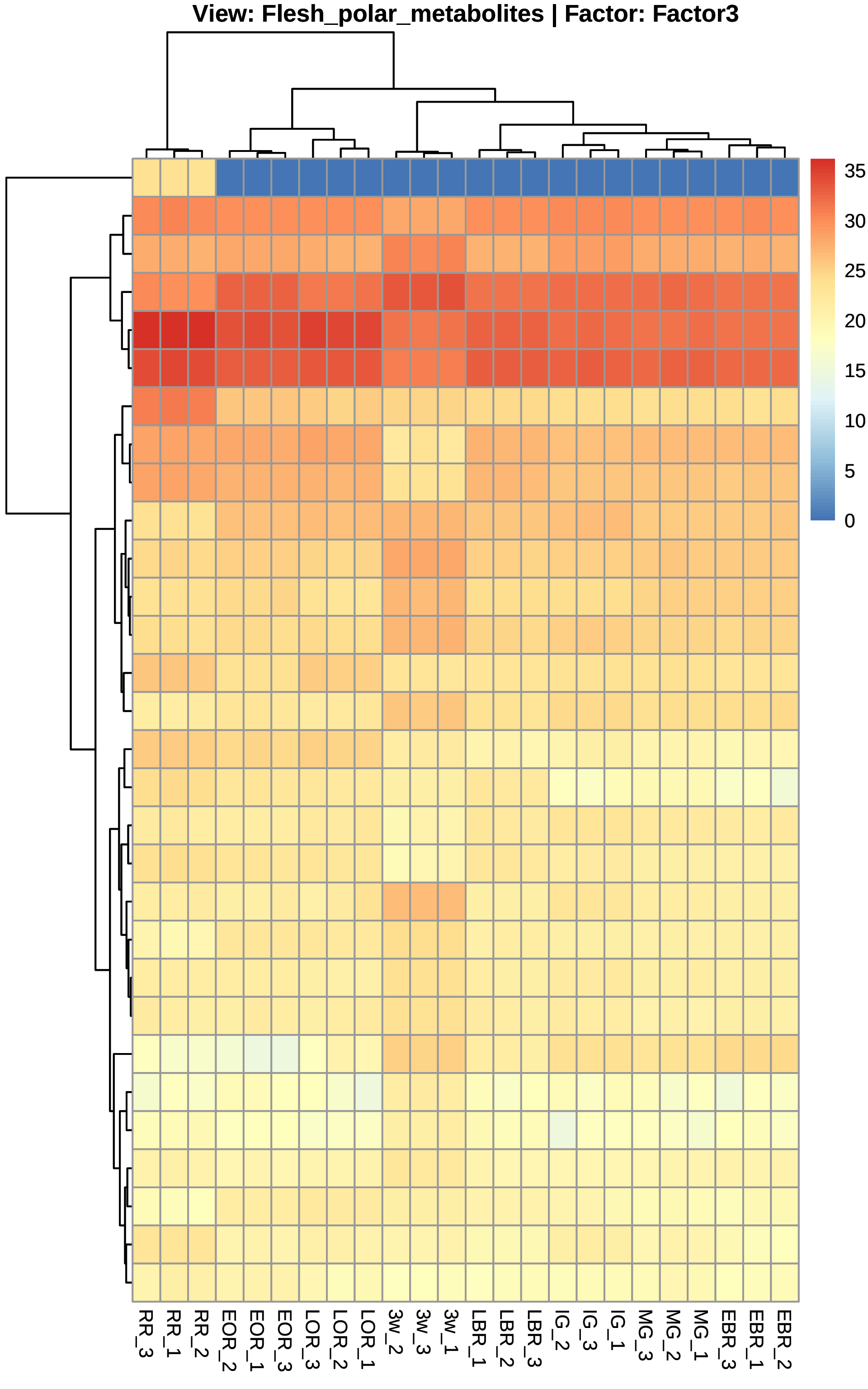
View: Flesh_polar_metabolites | Factor: Factor3
35
30
25
20
15
10
5
0
RR_3
RR_1
RR_2
EOR_2
EOR_1
EOR_3
LOR_3
LOR_2
LOR_1
3w_2
3w_3
3w_1
LBR_1
LBR_2
LBR_3
IG_2
IG_3
IG_1
MG_3
MG_2
MG_1
EBR_3
EBR_1
EBR_2

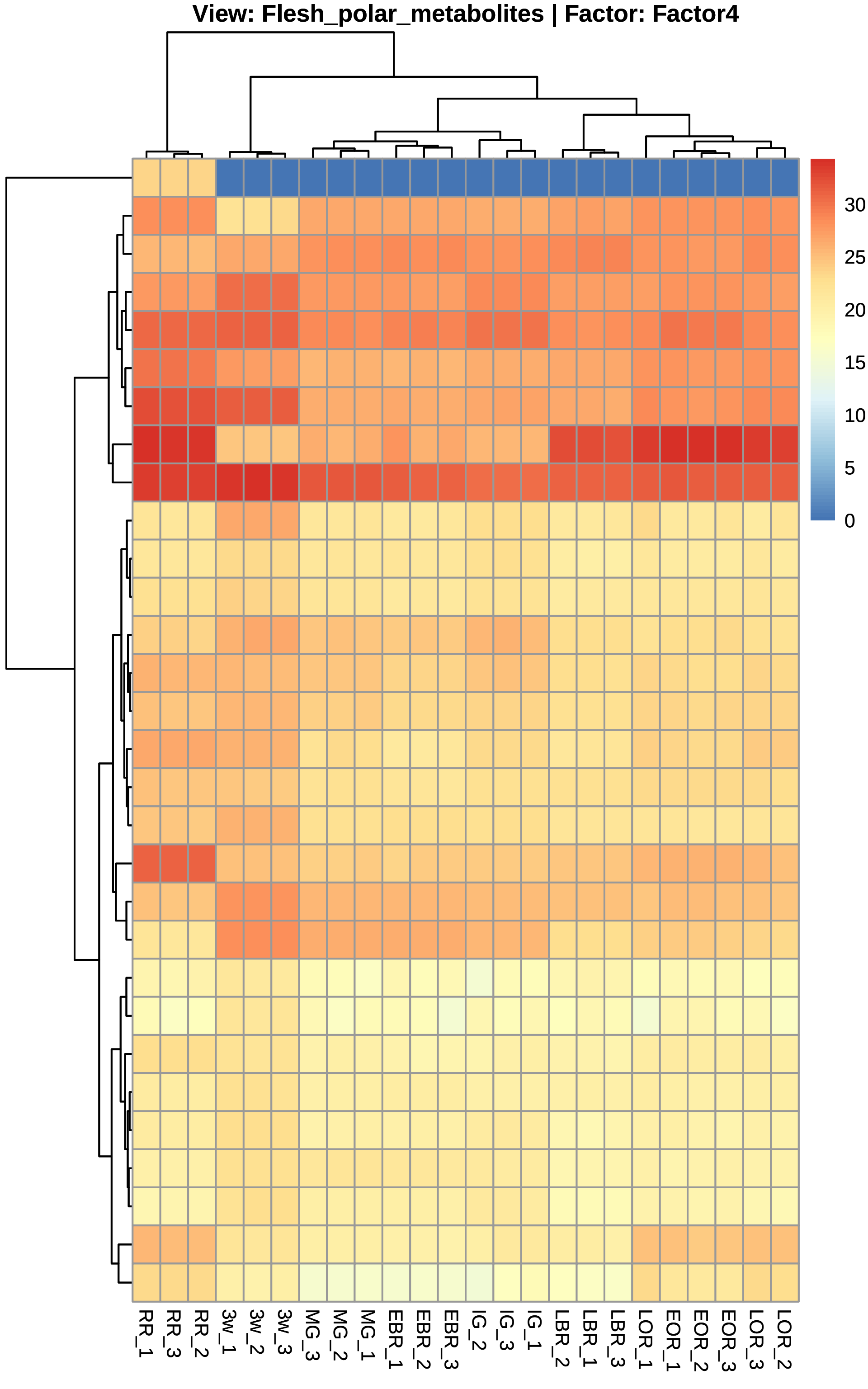
View: Flesh_polar_metabolites | Factor: Factor4
30
25
20
15
10
5
0
RR_1
RR_3
RR_2
3w_1
3w_2
3w_3
MG_3
MG_2
MG_1
EBR_1
EBR_2
EBR_3
IG_2
IG_3
IG_1
LBR_2
LBR_1
LBR_3
LOR_1
EOR_1
EOR_2
EOR_3
LOR_3
LOR_2

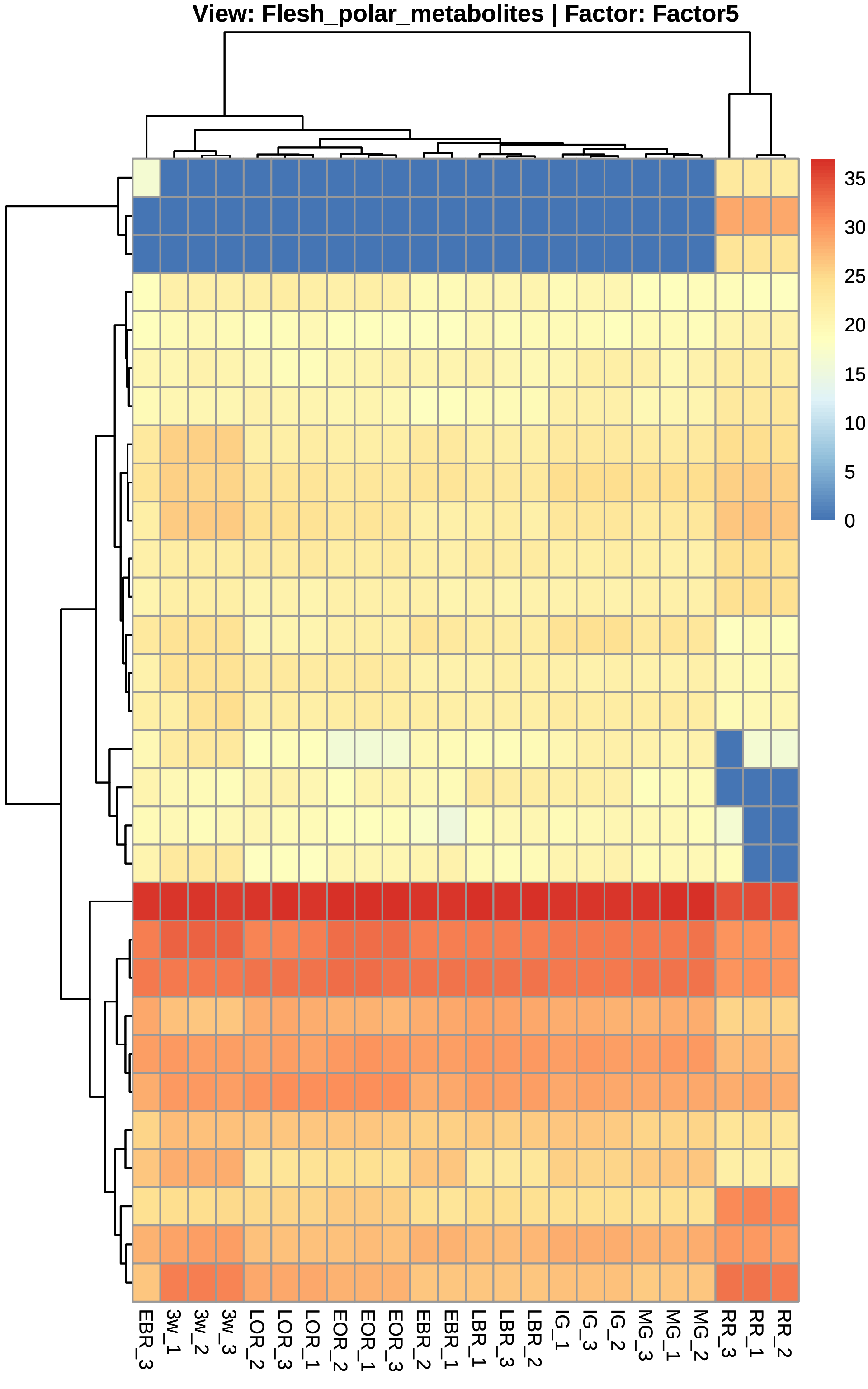
View: Flesh_polar_metabolites | Factor: Factor5
35
30
25
20
15
10
5
0
EBR_3
3w_1
3w_2
3w_3
LOR_2
LOR_3
LOR_1
EOR_2
EOR_1
EOR_3
EBR_2
EBR_1
LBR_1
LBR_3
LBR_2
IG_1
IG_3
IG_2
MG_3
MG_1
MG_2
RR_3
RR_1
RR_2

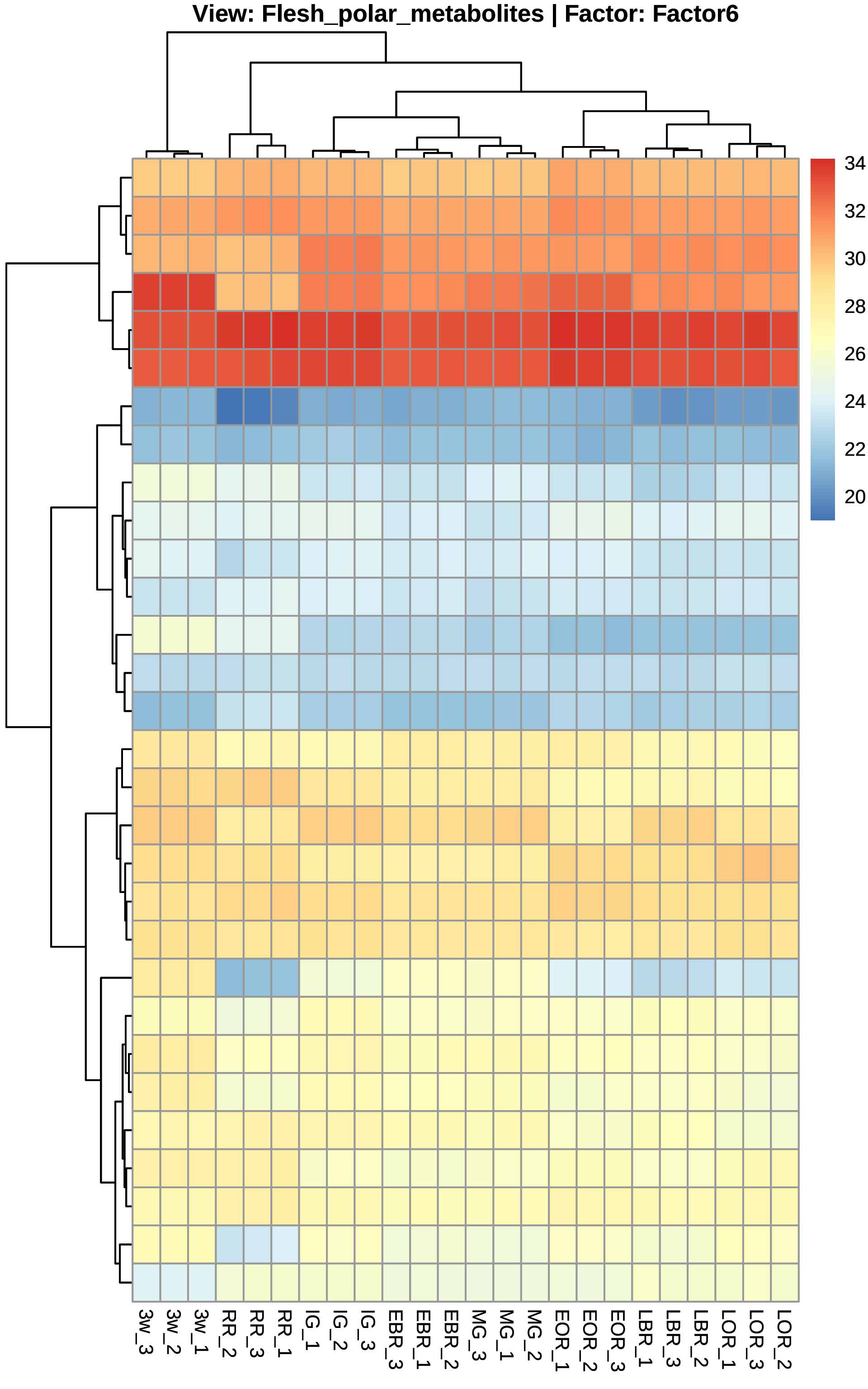
View: Flesh_polar_metabolites | Factor: Factor6
34
32
30
28
26
24
22
20
3w_3
3w_2
3w_1
RR_2
RR_3
RR_1
IG_1
IG_2
IG_3
EBR_3
EBR_1
EBR_2
MG_3
MG_1
MG_2
EOR_1
EOR_2
EOR_3
LBR_1
LBR_3
LBR_2
LOR_1
LOR_3
LOR_2

View: Flesh_polar_metabolites | Factor: Factor7

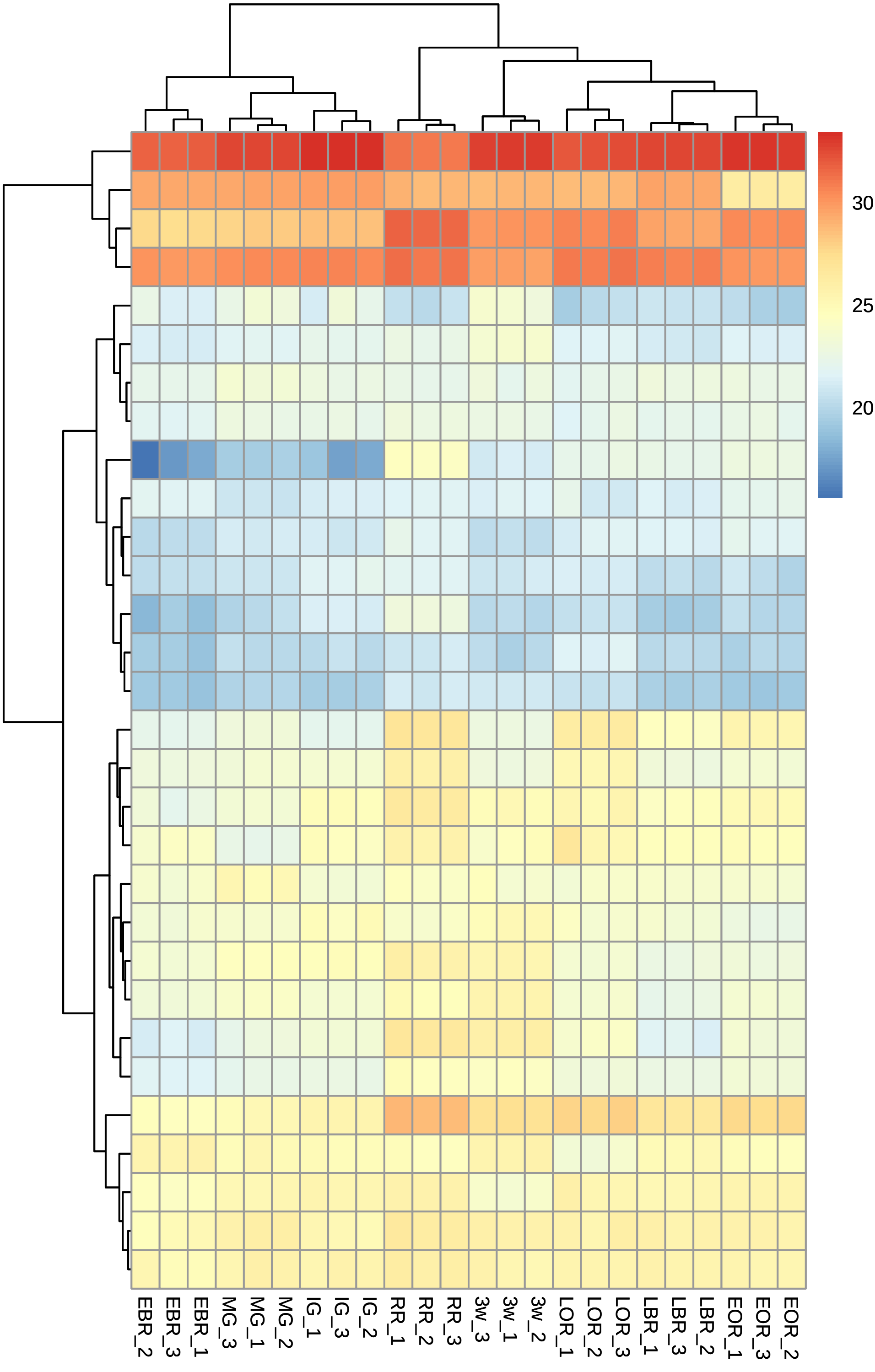

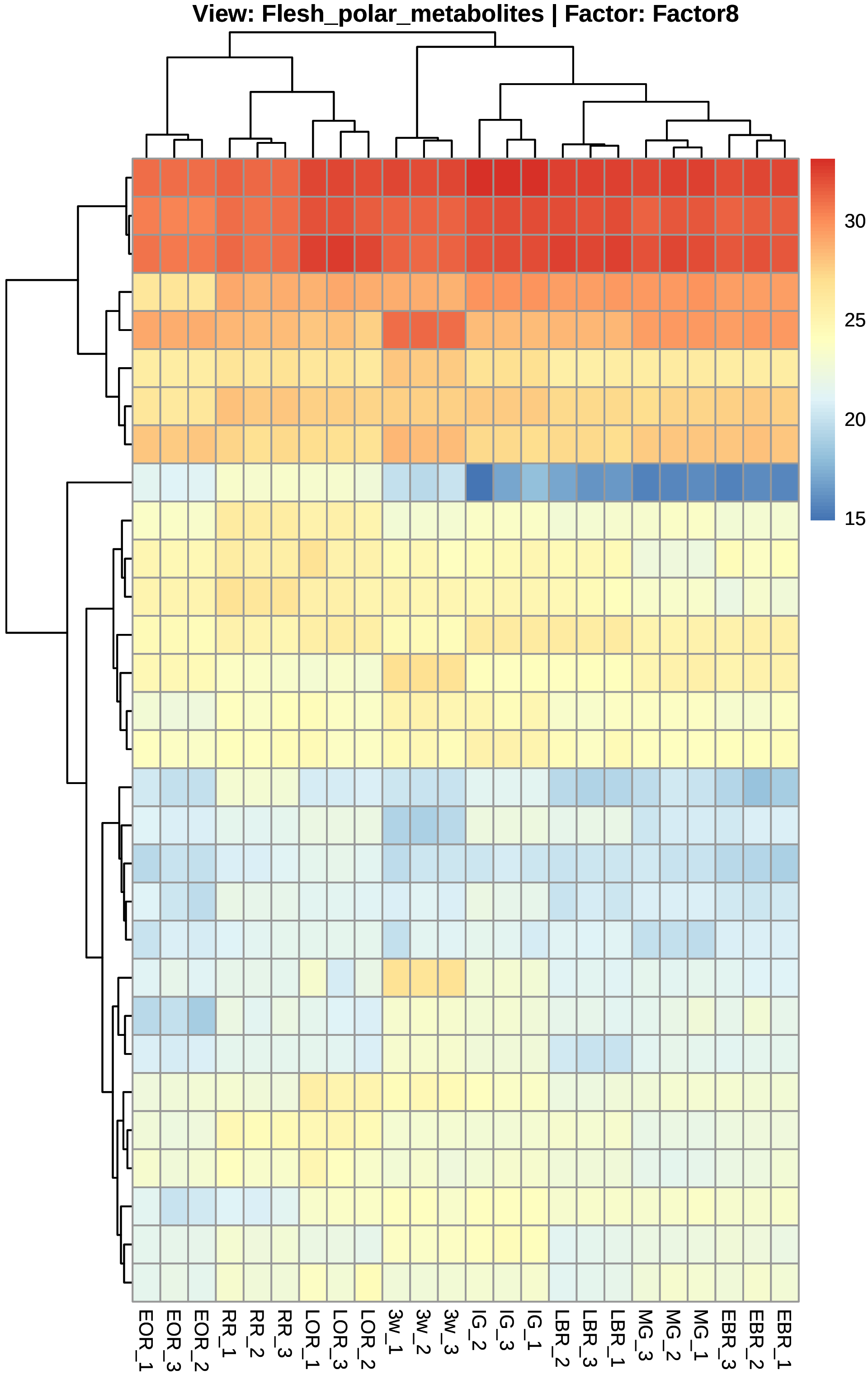
View: Flesh_polar_metabolites | Factor: Factor8
30
25
20
15
EOR_1
EOR_3
EOR_2
RR_1
RR_2
RR_3
LOR_1
LOR_3
LOR_2
3w_1
3w_2
3w_3
IG_2
IG_3
IG_1
LBR_2
LBR_3
LBR_1
MG_3
MG_2
MG_1
EBR_3
EBR_2
EBR_1

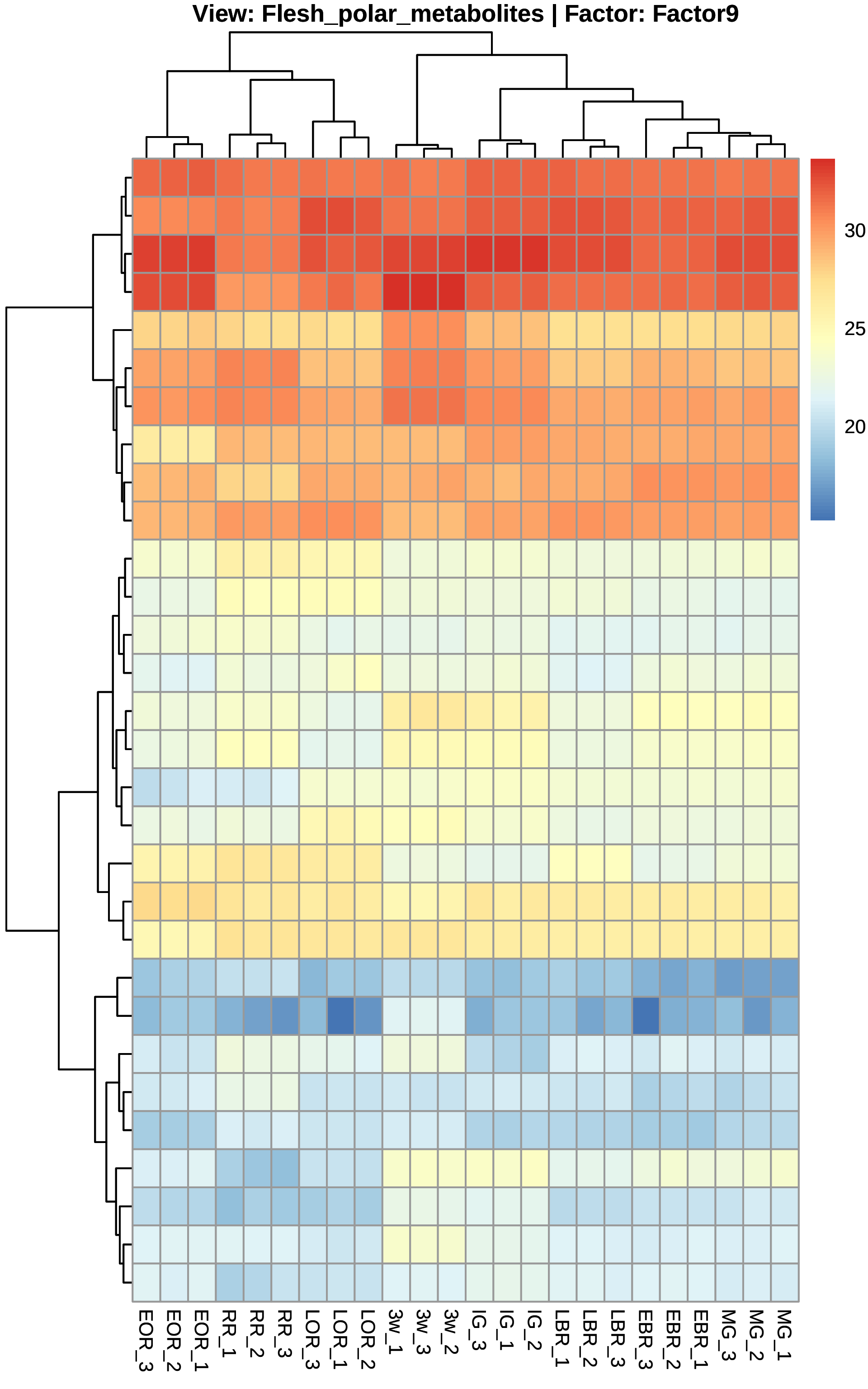
View: Flesh_polar_metabolites | Factor: Factor9
30
25
20
EOR_3
EOR_2
EOR_1
RR_1
RR_2
RR_3
LOR_3
LOR_1
LOR_2
3w_1
3w_3
3w_2
IG_3
IG_1
IG_2
LBR_1
LBR_2
LBR_3
EBR_3
EBR_2
EBR_1
MG_3
MG_2
MG_1

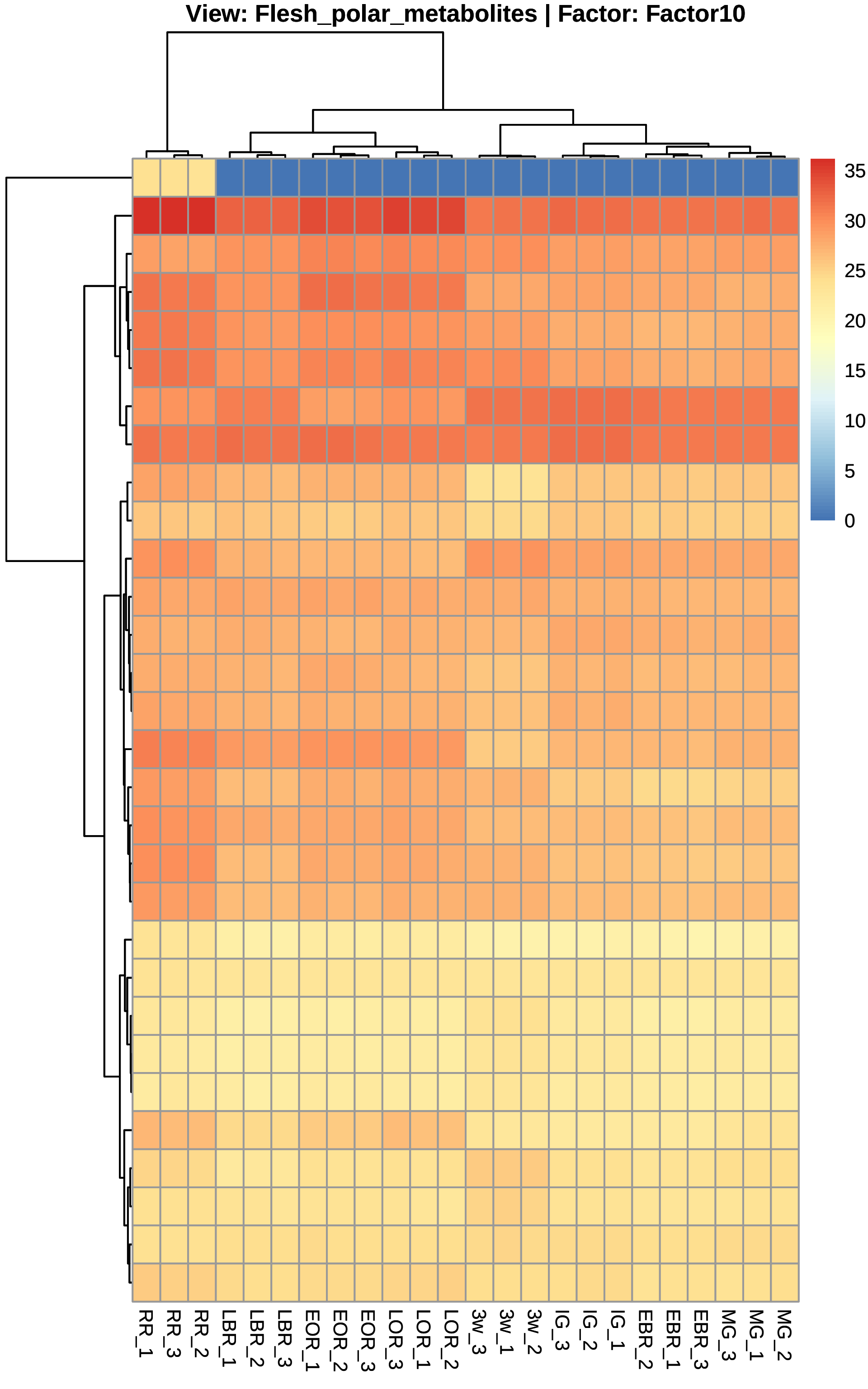
View: Flesh_polar_metabolites | Factor: Factor10
35
30
25
20
15
10
5
0
RR_1
RR_3
RR_2
LBR_1
LBR_2
LBR_3
EOR_1
EOR_2
EOR_3
LOR_3
LOR_1
LOR_2
3w_3
3w_1
3w_2
IG_3
IG_2
IG_1
EBR_2
EBR_1
EBR_3
MG_3
MG_1
MG_2

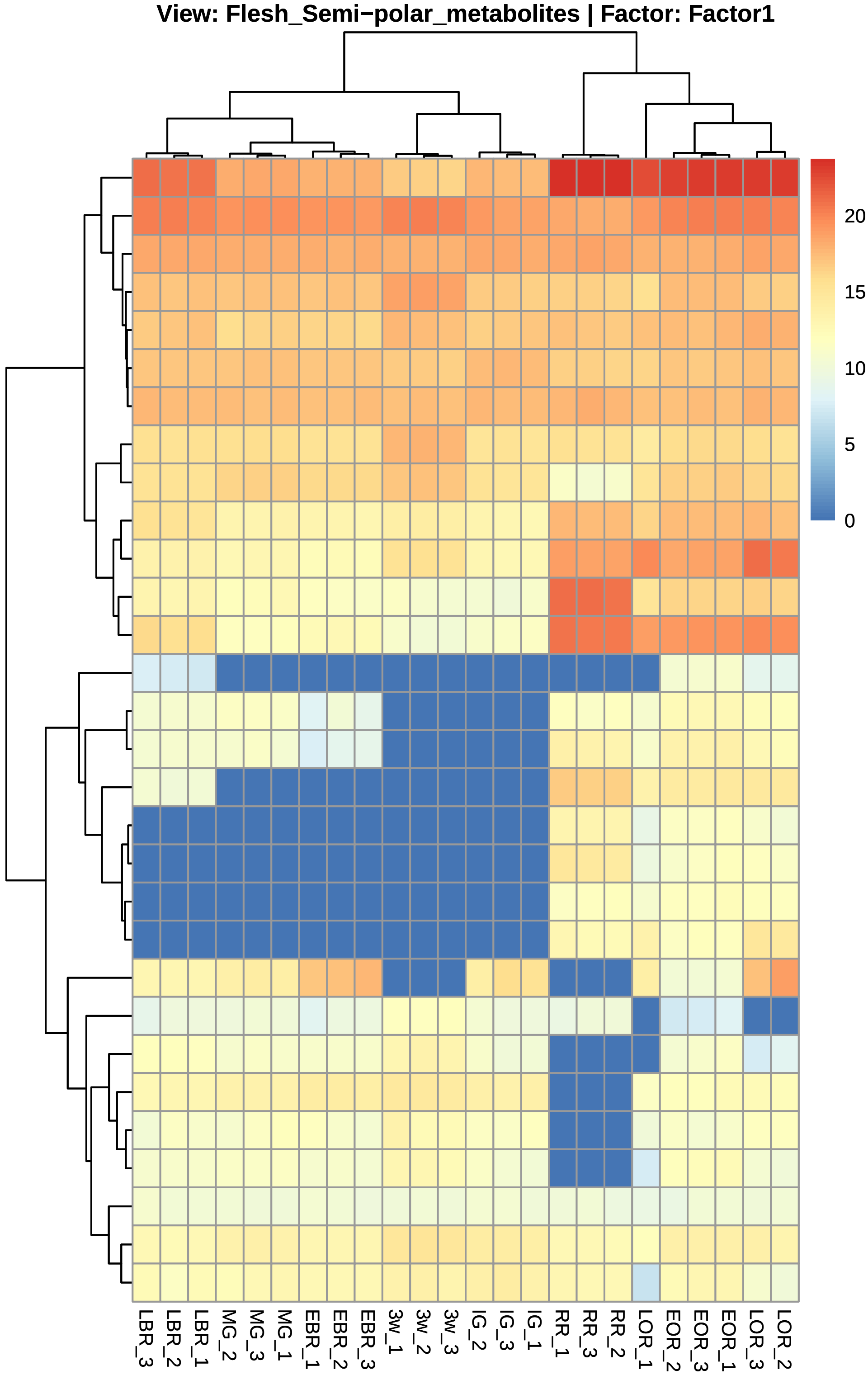
View: Flesh_Semi−polar_metabolites | Factor: Factor1
20
15
10
5
0
LBR_3
LBR_2
LBR_1
MG_2
MG_3
MG_1
EBR_1
EBR_2
EBR_3
3w_1
3w_2
3w_3
IG_2
IG_3
IG_1
RR_1
RR_3
RR_2
LOR_1
EOR_2
EOR_3
EOR_1
LOR_3
LOR_2

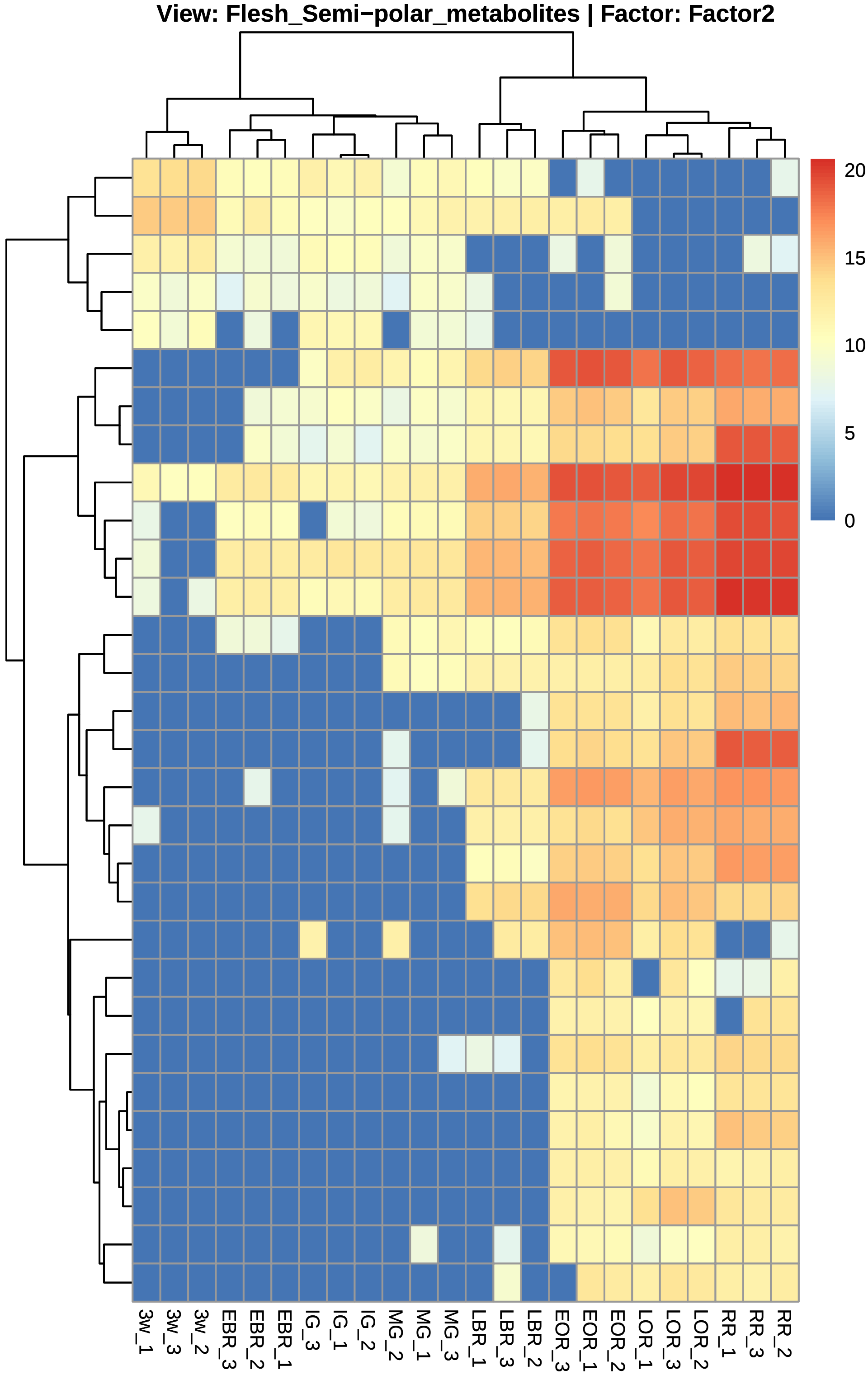
View: Flesh_Semi−polar_metabolites | Factor: Factor2
20
15
10
5
0
3w_1
3w_3
3w_2
EBR_3
EBR_2
EBR_1
IG_3
IG_1
IG_2
MG_2
MG_1
MG_3
LBR_1
LBR_3
LBR_2
EOR_3
EOR_1
EOR_2
LOR_1
LOR_3
LOR_2
RR_1
RR_3
RR_2

View: Flesh_Semi−polar_metabolites | Factor: Factor3

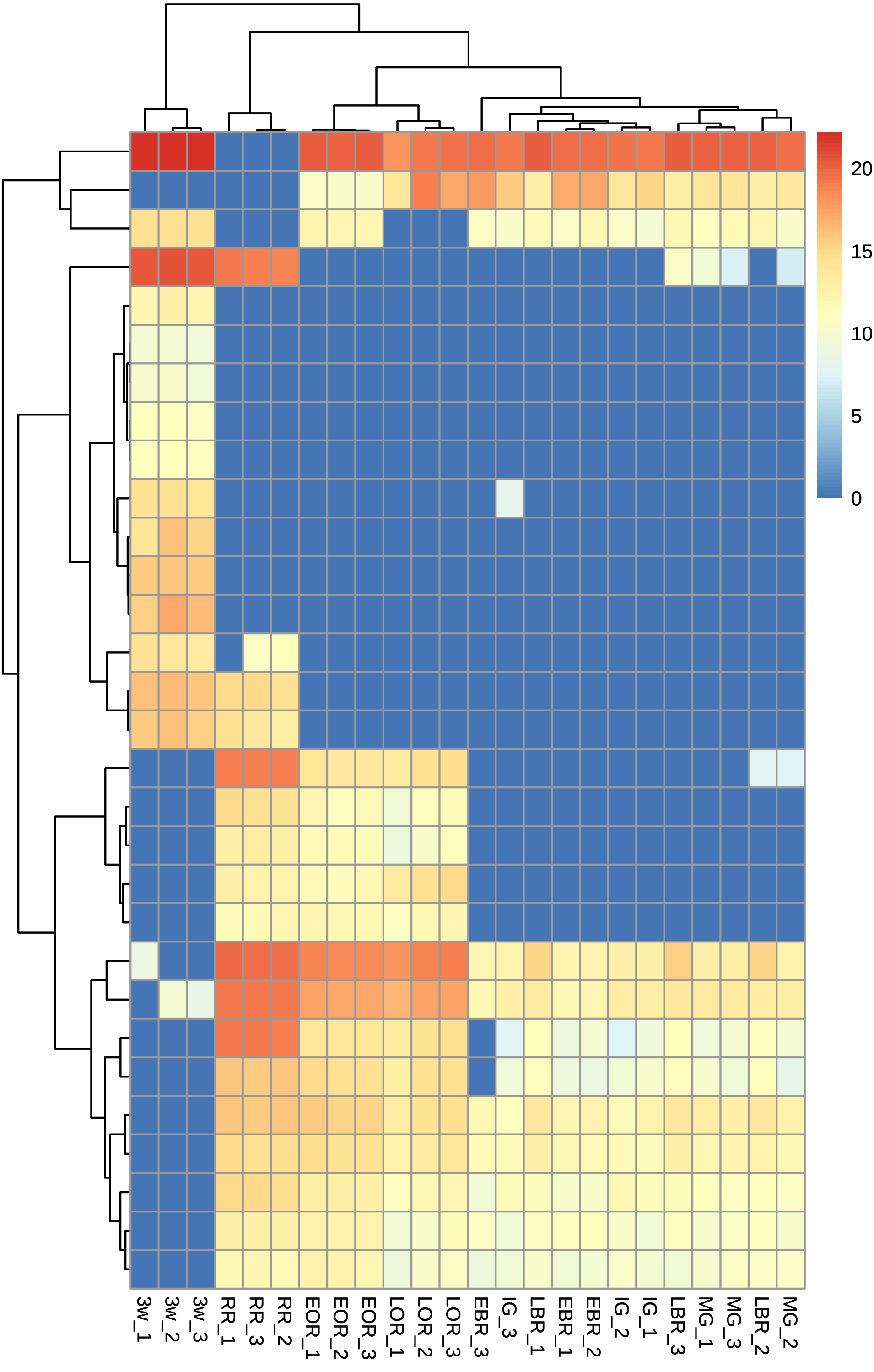

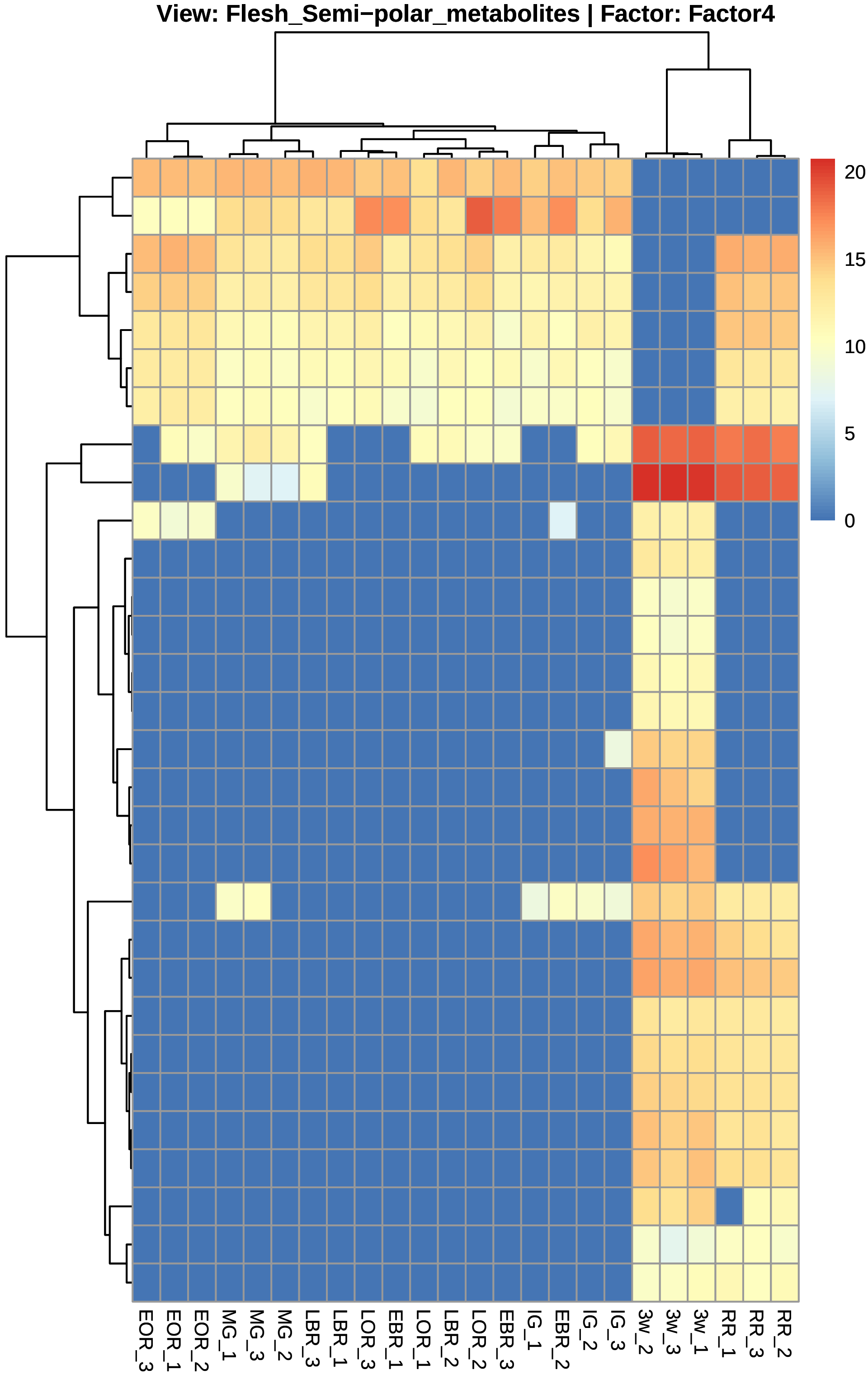

View: Flesh_Semi−polar_metabolites | Factor: Factor4
20
15
10
5
0
EOR_3
EOR_1
EOR_2
MG_1
MG_3
MG_2
LBR_3
LBR_1
LOR_3
EBR_1
LOR_1
LBR_2
LOR_2
EBR_3
IG_1
EBR_2
IG_2
IG_3
3w_2
3w_3
3w_1
RR_1
RR_3
RR_2

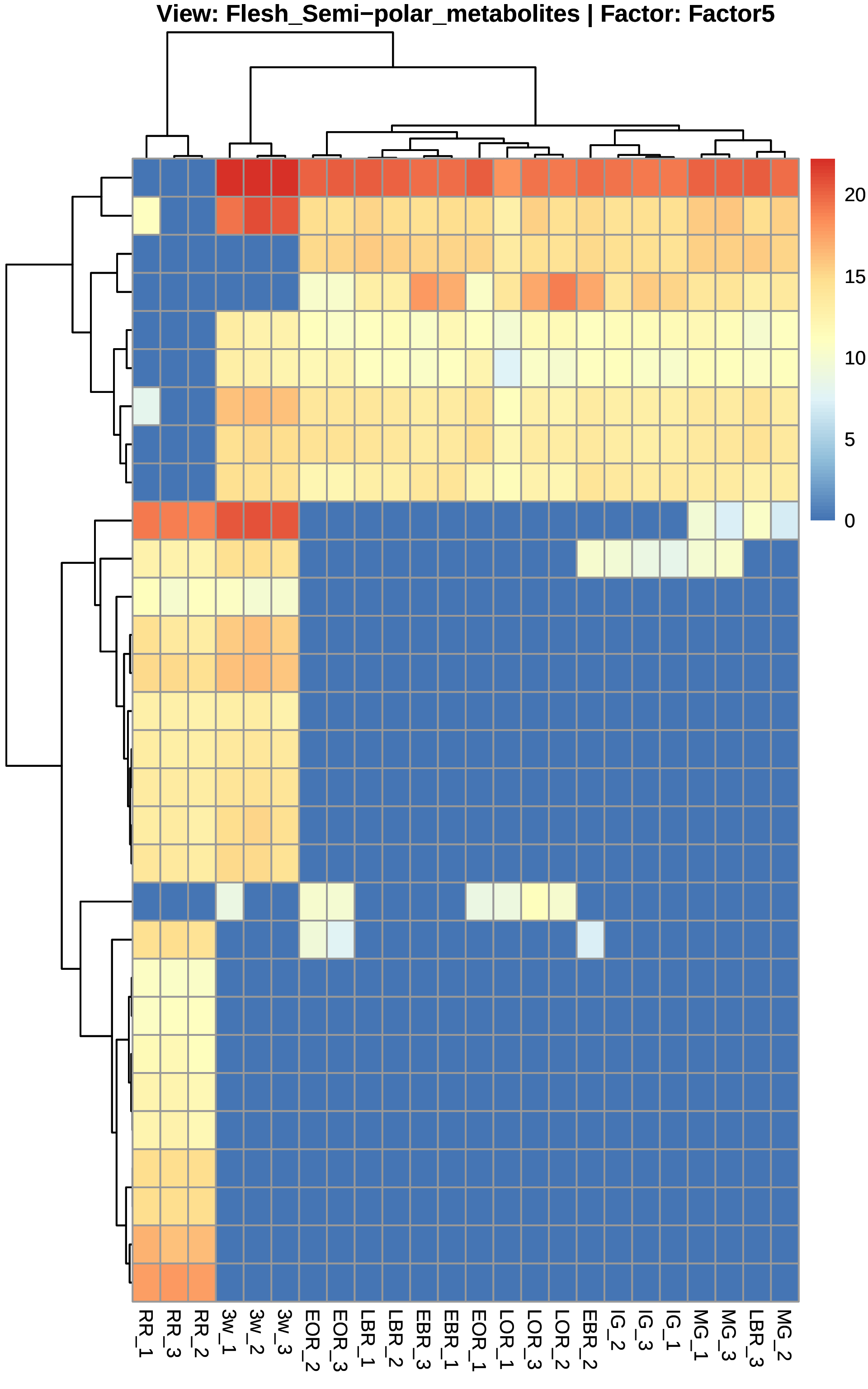
View: Flesh_Semi−polar_metabolites | Factor: Factor5
20
15
10
5
0
RR_1
RR_3
RR_2
3w_1
3w_2
3w_3
EOR_2
EOR_3
LBR_1
LBR_2
EBR_3
EBR_1
EOR_1
LOR_1
LOR_3
LOR_2
EBR_2
IG_2
IG_3
IG_1
MG_1
MG_3
LBR_3
MG_2

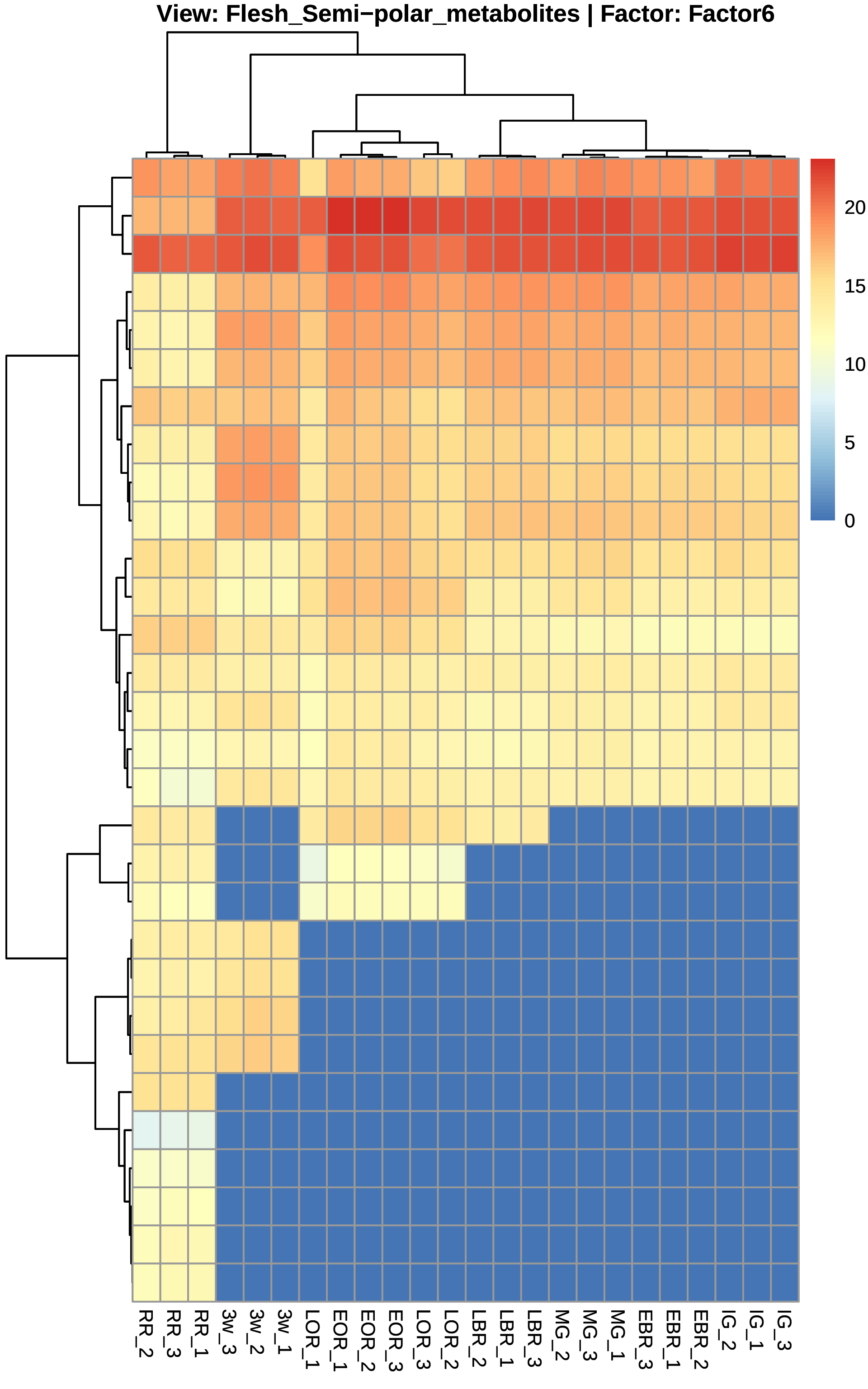
View: Flesh_Semi−polar_metabolites | Factor: Factor6
20
15
10
5
0
RR_2
RR_3
RR_1
3w_3
3w_2
3w_1
LOR_1
EOR_1
EOR_2
EOR_3
LOR_3
LOR_2
LBR_2
LBR_1
LBR_3
MG_2
MG_3
MG_1
EBR_3
EBR_1
EBR_2
IG_2
IG_1
IG_3

View: Flesh_Semi−polar_metabolites | Factor: Factor7

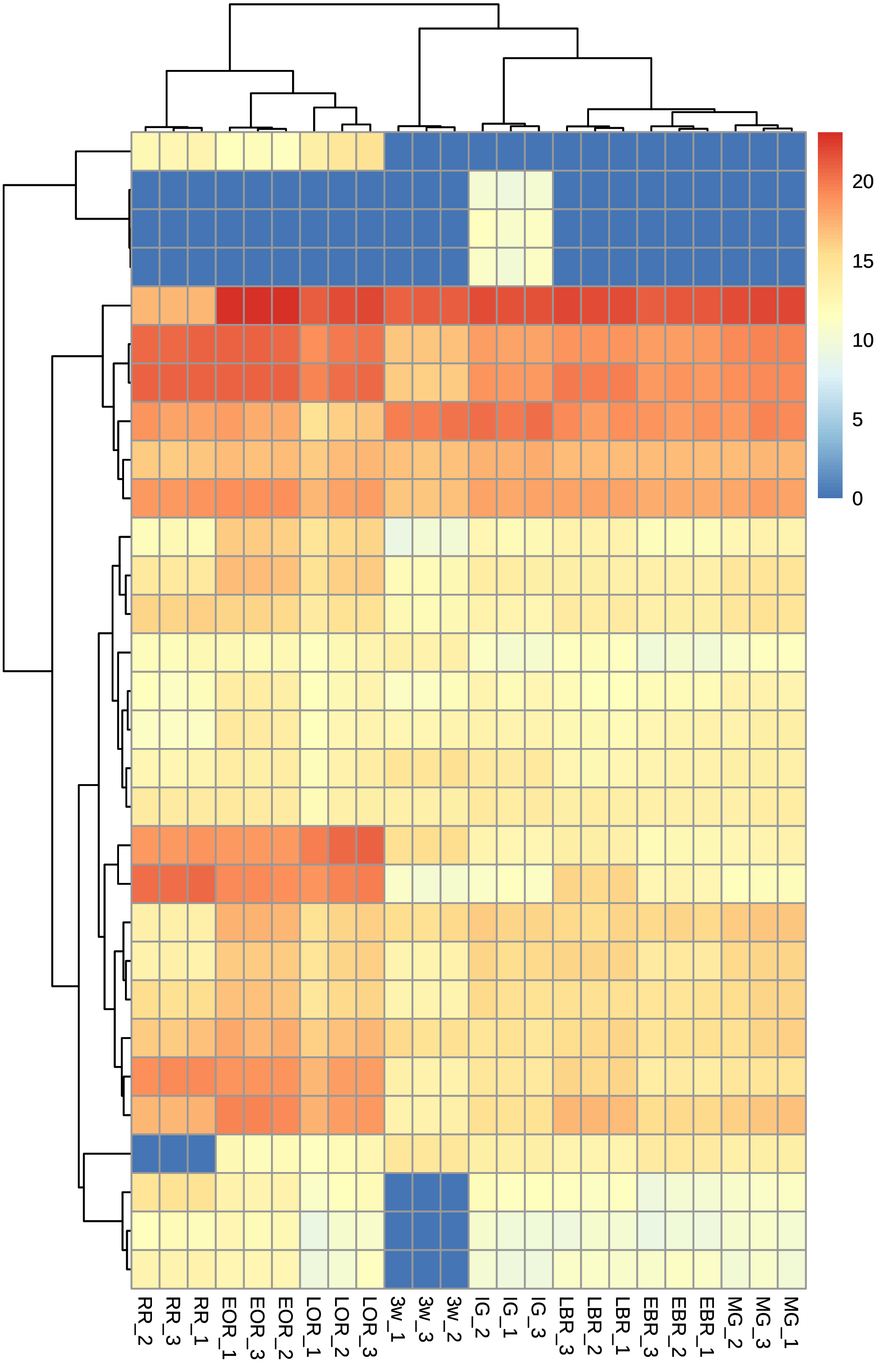

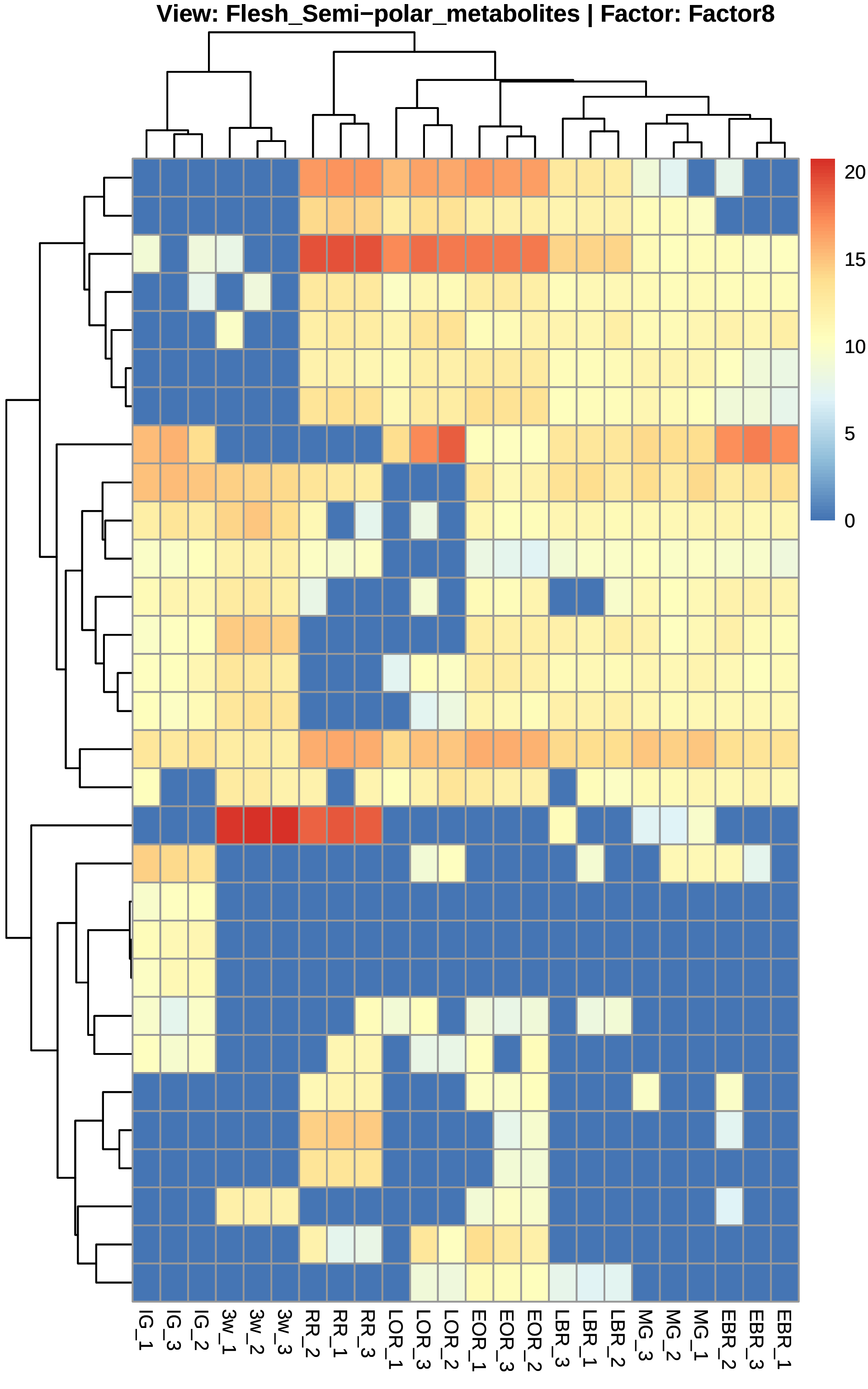
View: Flesh_Semi−polar_metabolites | Factor: Factor8
IG_1
IG_3
IG_2
3w_1
3w_2
3w_3
RR_2
RR_1
RR_3
LOR_1
LOR_3
LOR_2
EOR_1
EOR_3
EOR_2
LBR_3
LBR_1
LBR_2
MG_3
MG_2
MG_1
EBR_2
EBR_3
EBR_1
20
15
10
5
0

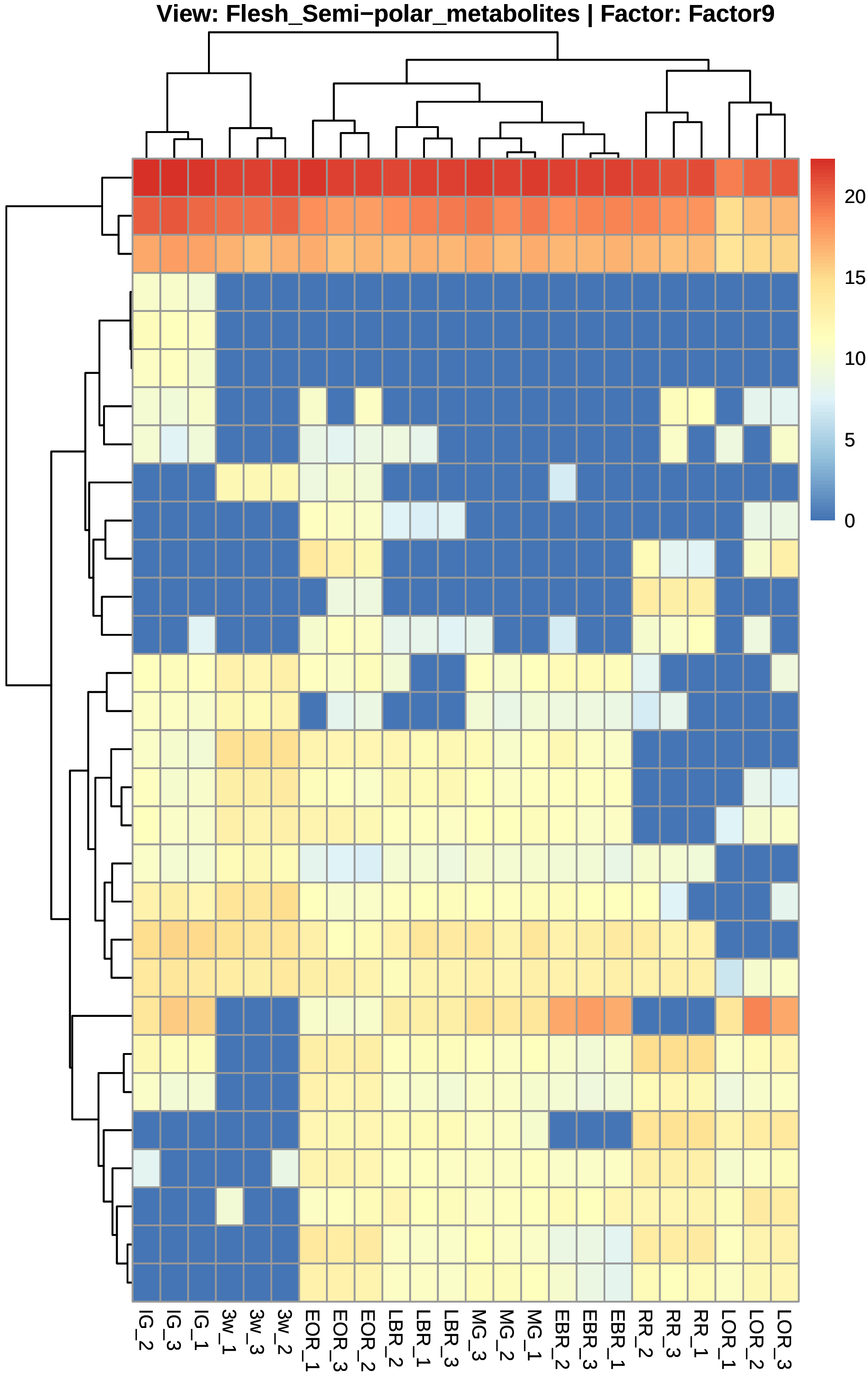

View: Flesh_Semi−polar_metabolites | Factor: Factor9
20
15
10
5
0
IG_2
IG_3
IG_1
3w_1
3w_3
3w_2
EOR_1
EOR_3
EOR_2
LBR_2
LBR_1
LBR_3
MG_3
MG_2
MG_1
EBR_2
EBR_3
EBR_1
RR_2
RR_3
RR_1
LOR_1
LOR_2
LOR_3

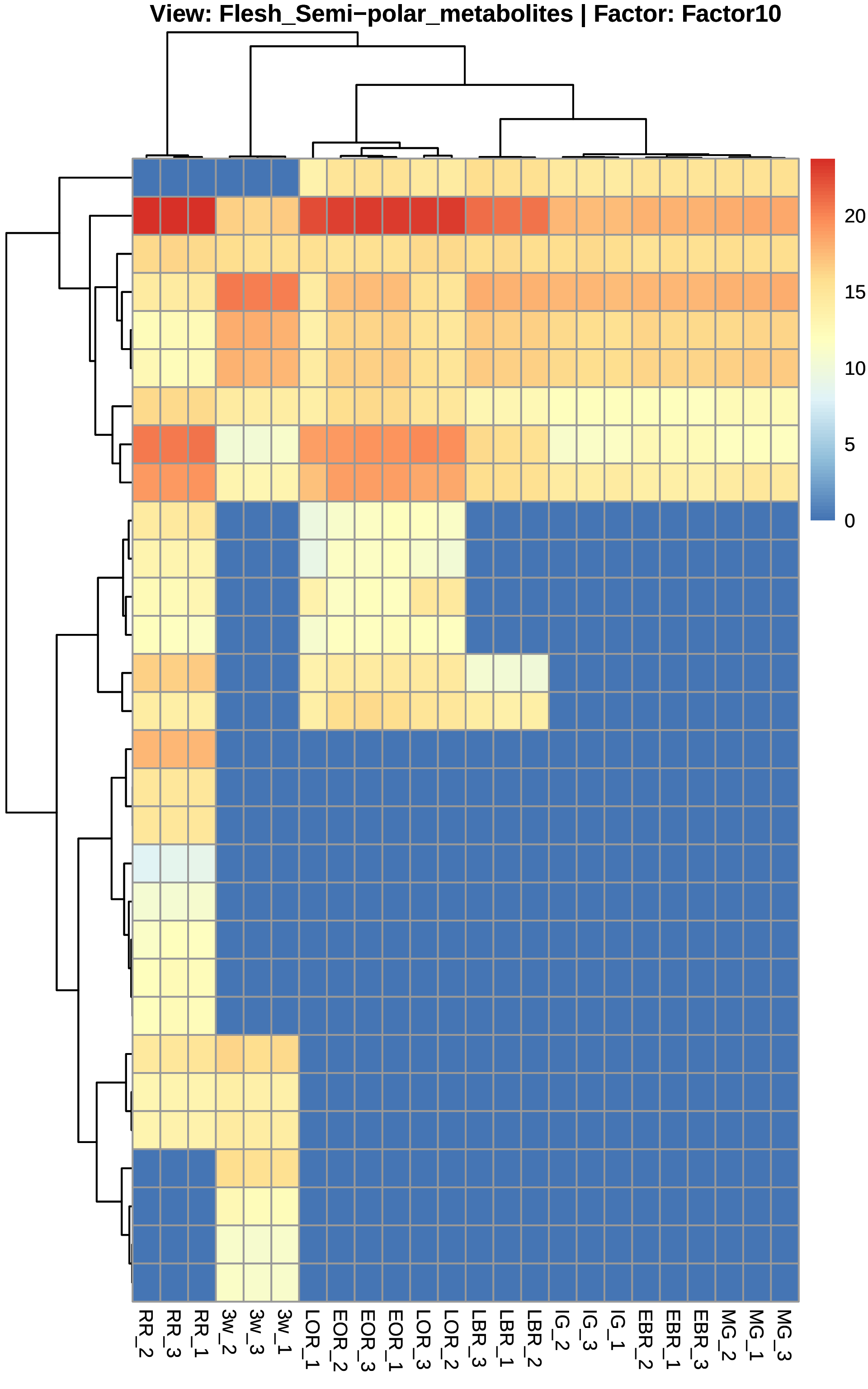
View: Flesh_Semi−polar_metabolites | Factor: Factor10
20
15
10
5
0
RR_2
RR_3
RR_1
3w_2
3w_3
3w_1
LOR_1
EOR_2
EOR_3
EOR_1
LOR_3
LOR_2
LBR_3
LBR_1
LBR_2
IG_2
IG_3
IG_1
EBR_2
EBR_1
EBR_3
MG_2
MG_1
MG_3

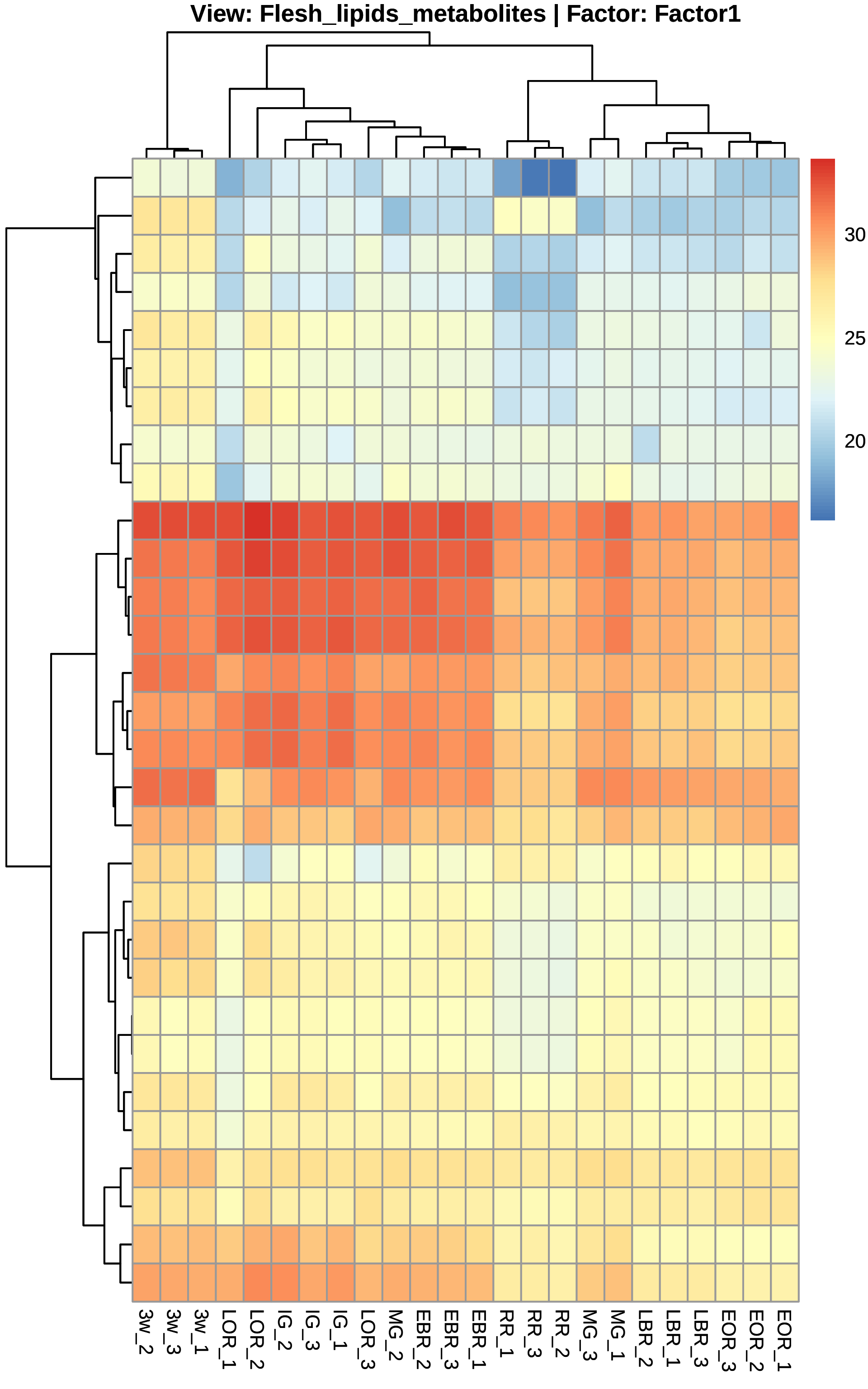
View: Flesh_lipids_metabolites | Factor: Factor1
30
25
20
3w_2
3w_3
3w_1
LOR_1
LOR_2
IG_2
IG_3
IG_1
LOR_3
MG_2
EBR_2
EBR_3
EBR_1
RR_1
RR_3
RR_2
MG_3
MG_1
LBR_2
LBR_1
LBR_3
EOR_3
EOR_2
EOR_1

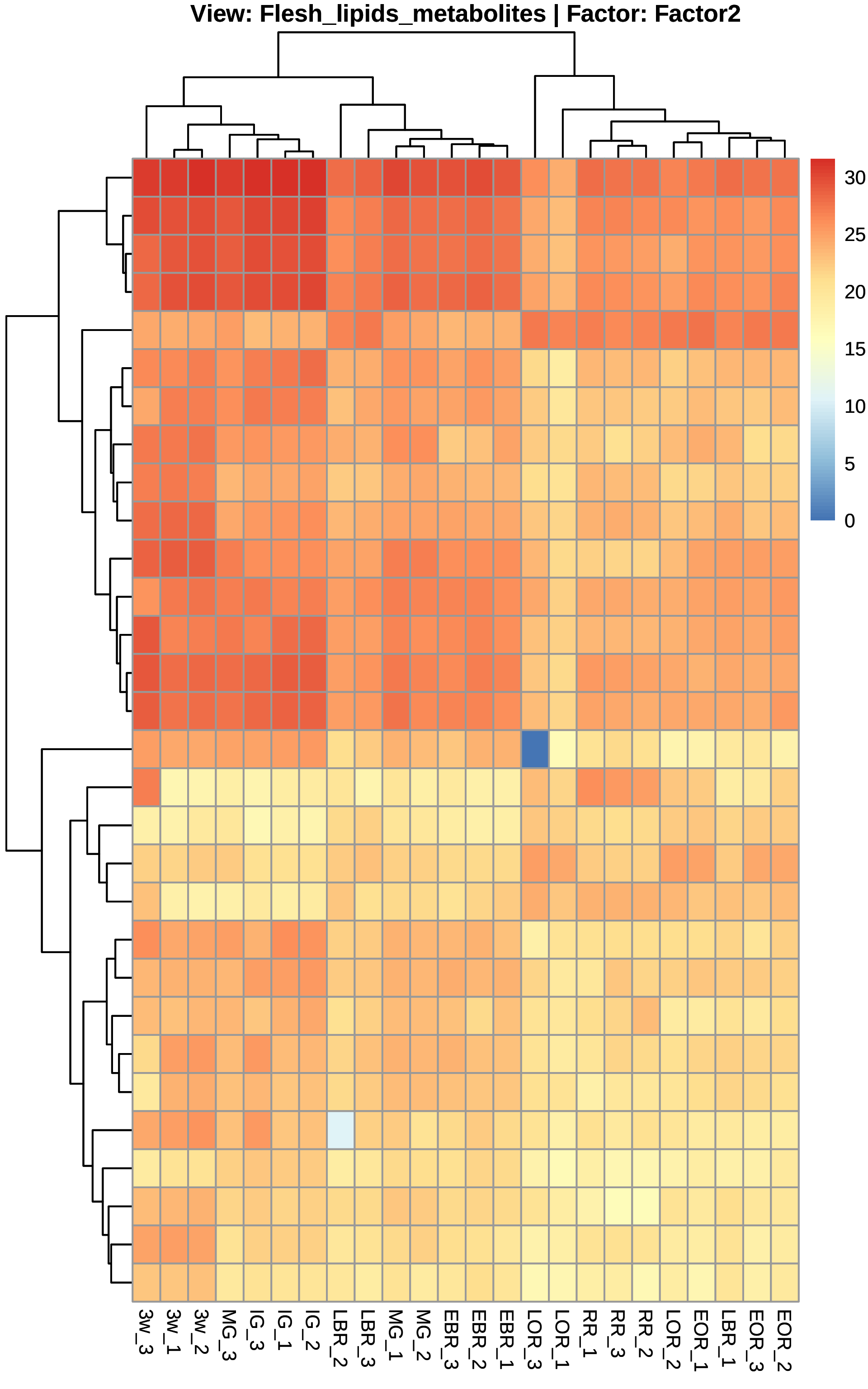
View: Flesh_lipids_metabolites | Factor: Factor2
30
25
20
15
10
5
0
3W_3
3W_1
3W_2
MG_3
IG_3
IG_1
IG_2
LBR_2
LBR_3
MG_1
MG_2
EBR_3
EBR_2
EBR_1
LOR_3
LOR_1
RR_1
RR_3
RR_2
LOR_2
EOR_1
LBR_1
EOR_3
EOR_2

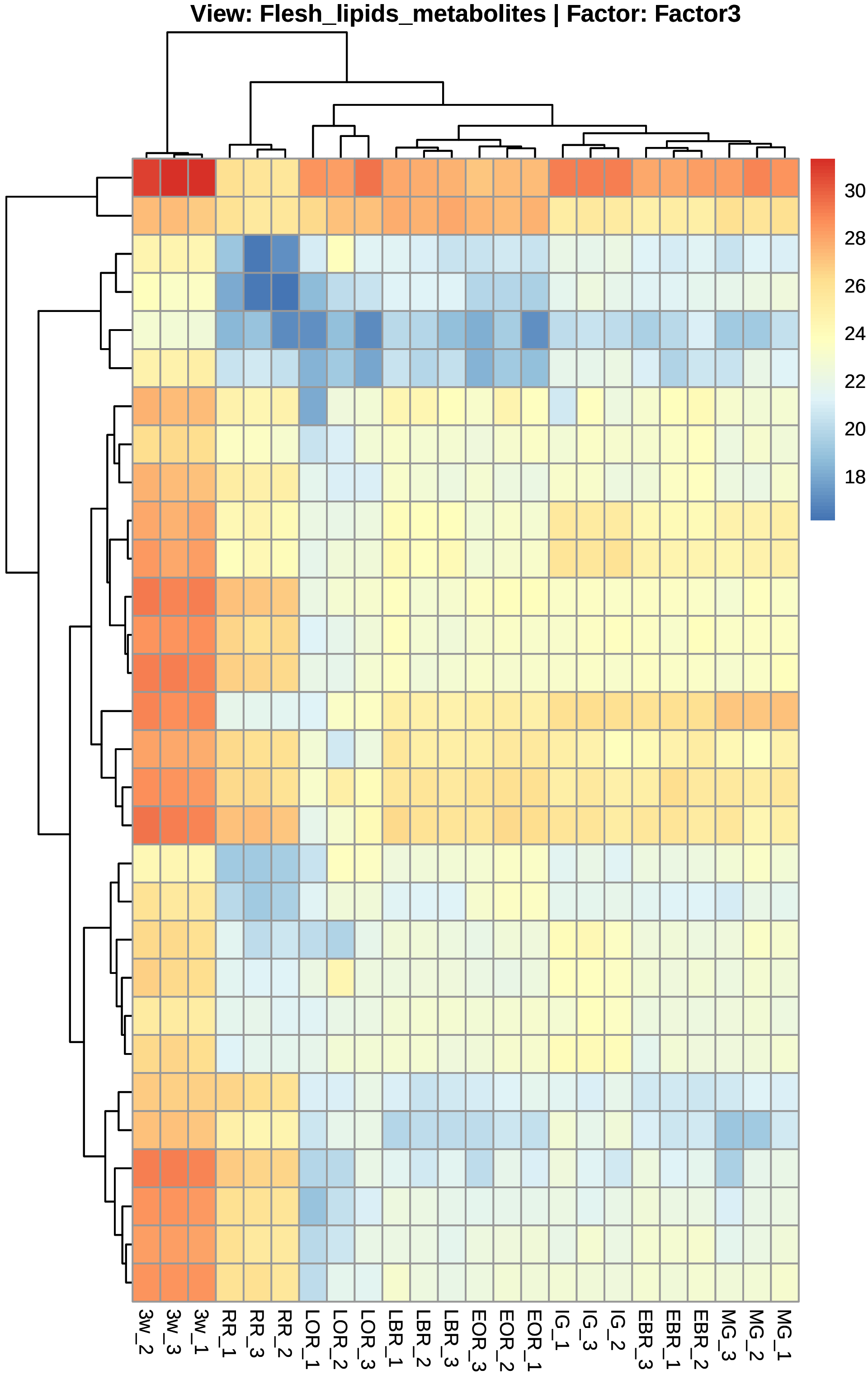
View: Flesh_lipids_metabolites | Factor: Factor3
30
28
26
24
22
20
18
3w_2
3w_3
3w_1
RR_1
RR_3
RR_2
LOR_1
LOR_2
LOR_3
LBR_1
LBR_2
LBR_3
EOR_3
EOR_2
EOR_1
IG_1
IG_3
IG_2
EBR_3
EBR_1
EBR_2
MG_3
MG_2
MG_1

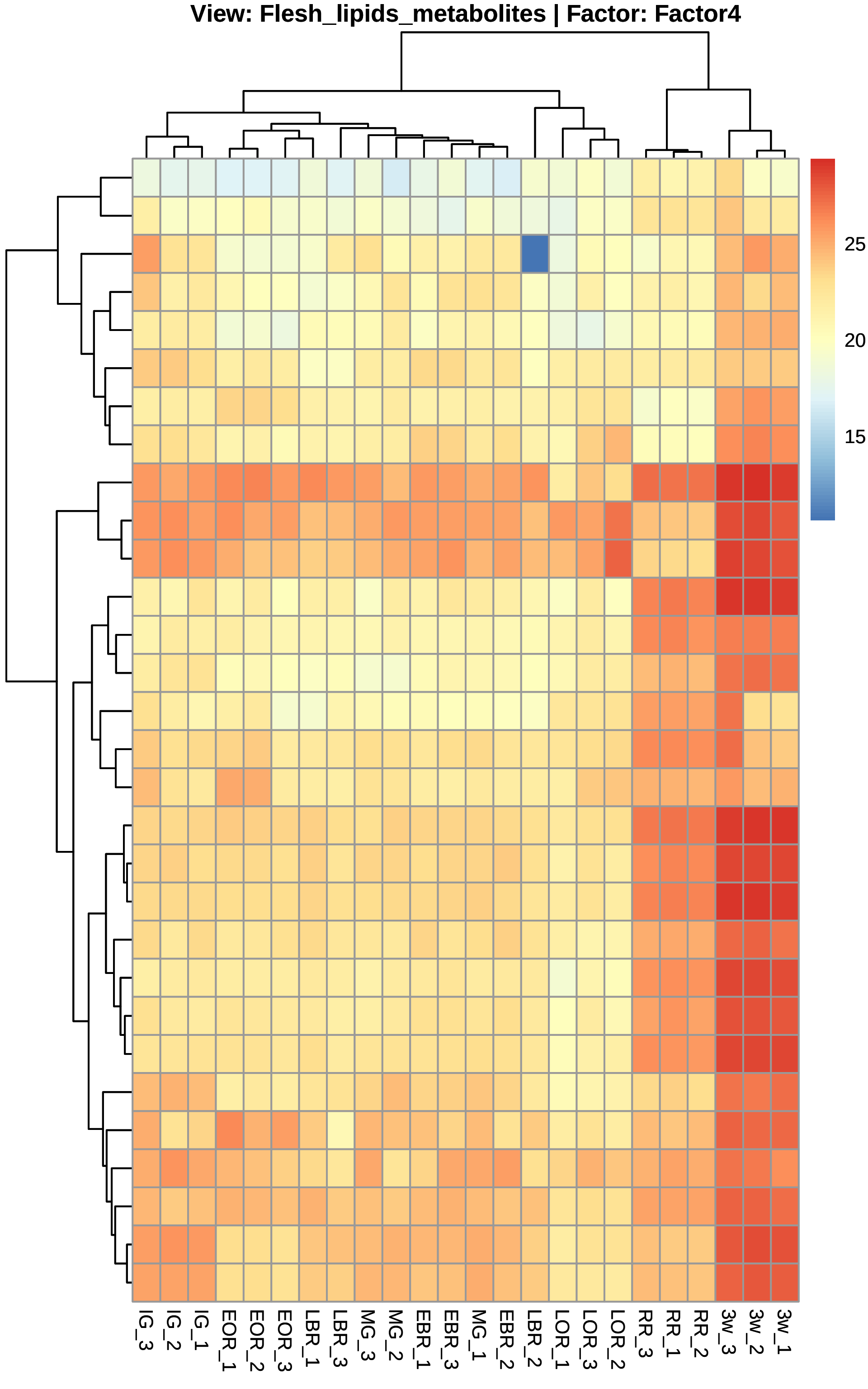

View: Flesh_lipids_metabolites | Factor: Factor4
25
20
15
IG_3
IG_2
IG_1
EOR_1
EOR_2
EOR_3
LBR_1
LBR_3
MG_3
MG_2
EBR_1
EBR_3
MG_1
EBR_2
LBR_2
LOR_1
LOR_3
LOR_2
RR_3
RR_1
RR_2
3w_3
3w_2
3w_1

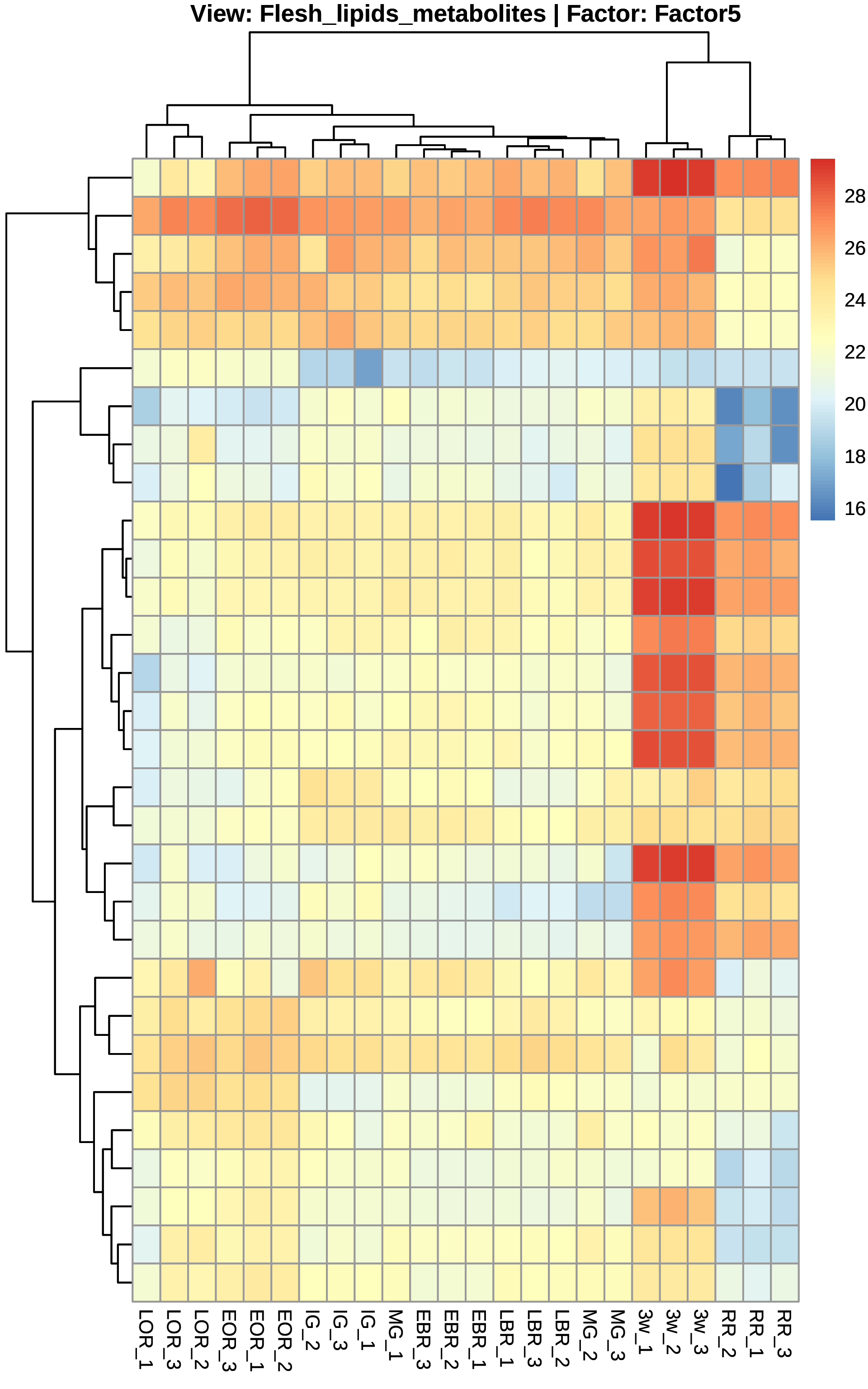
View: Flesh_lipids_metabolites | Factor: Factor5
28
26
24
22
20
18
16
LOR_1
LOR_3
LOR_2
EOR_3
EOR_1
EOR_2
IG_2
IG_3
IG_1
MG_1
EBR_3
EBR_2
EBR_1
LBR_1
LBR_3
LBR_2
MG_2
MG_3
3w_1
3w_2
3w_3
RR_2
RR_1
RR_3

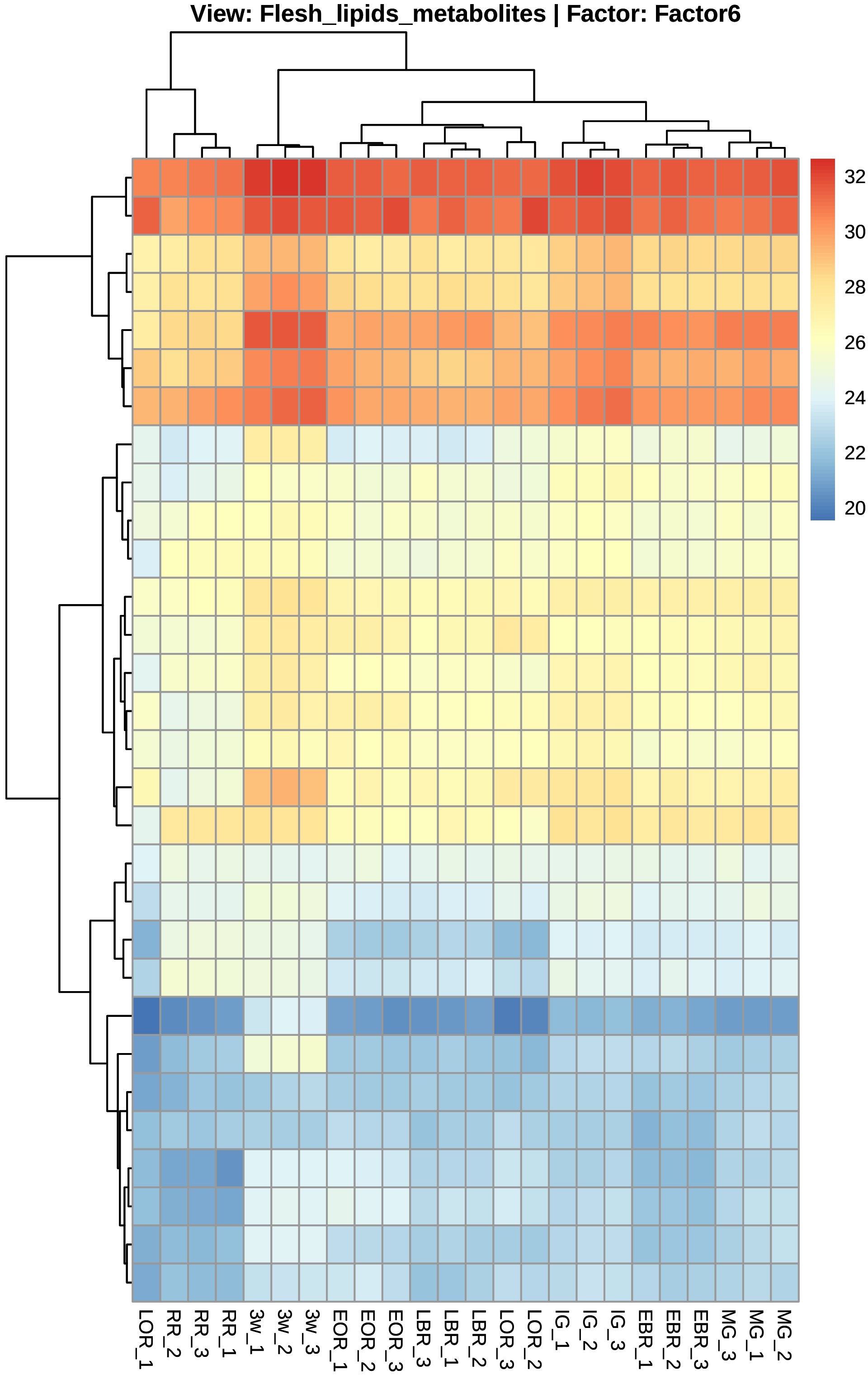
View: Flesh_lipids_metabolites | Factor: Factor6
32
30
28
26
24
22
20
LOR_1
RR_2
RR_3
RR_1
3w_1
3w_2
3w_3
EOR_1
EOR_2
EOR_3
LBR_3
LBR_1
LBR_2
LOR_3
LOR_2
IG_1
IG_2
IG_3
EBR_1
EBR_2
EBR_3
MG_3
MG_1
MG_2

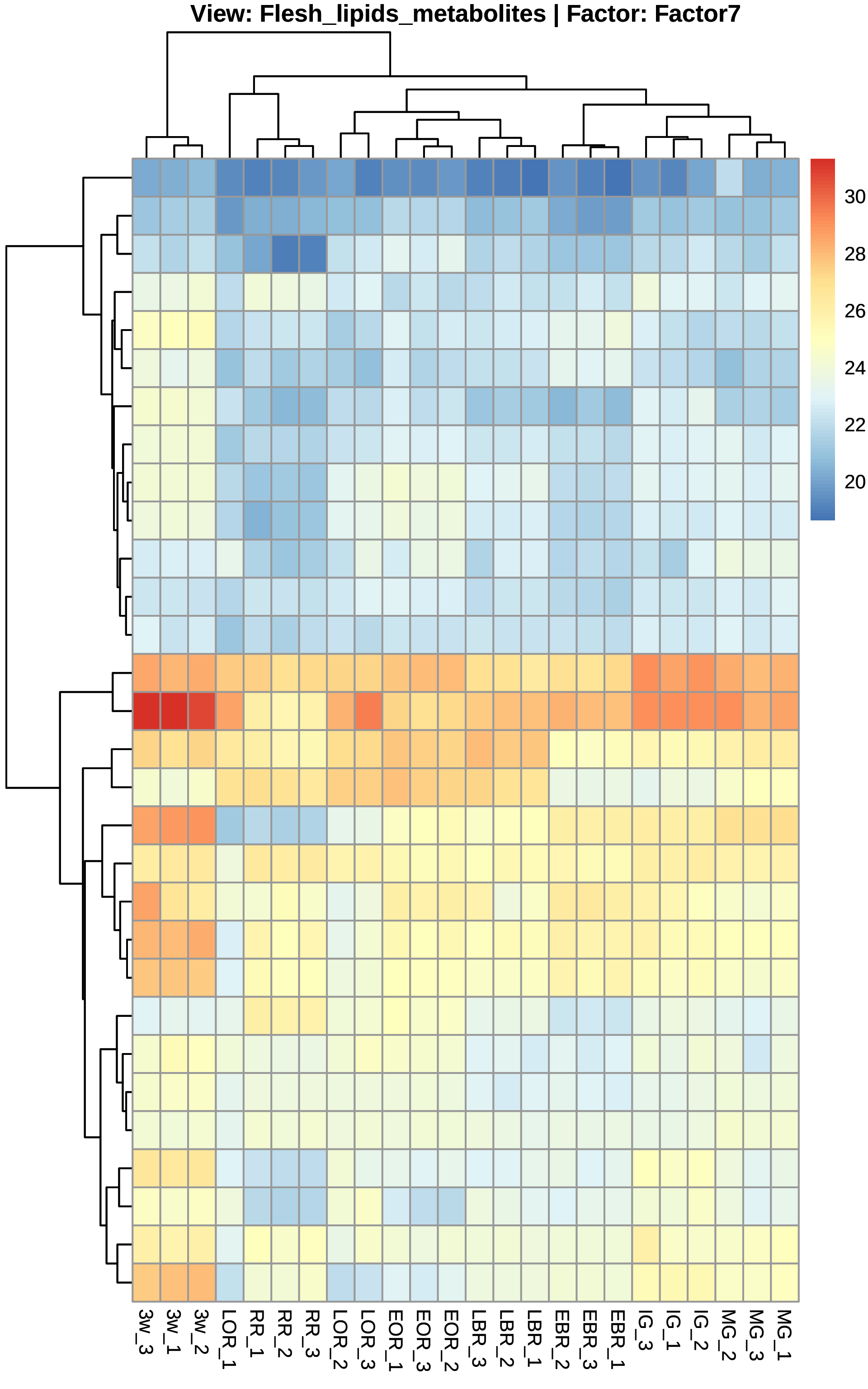
View: Flesh_lipids_metabolites | Factor: Factor7
3w_3
3w_1
3w_2
LOR_1
RR_1
RR_2
RR_3
LOR_2
LOR_3
EOR_1
EOR_3
EOR_2
LBR_3
LBR_2
LBR_1
EBR_2
EBR_3
EBR_1
IG_3
IG_1
IG_2
MG_2
MG_3
MG_1
30
28
26
24
22
20

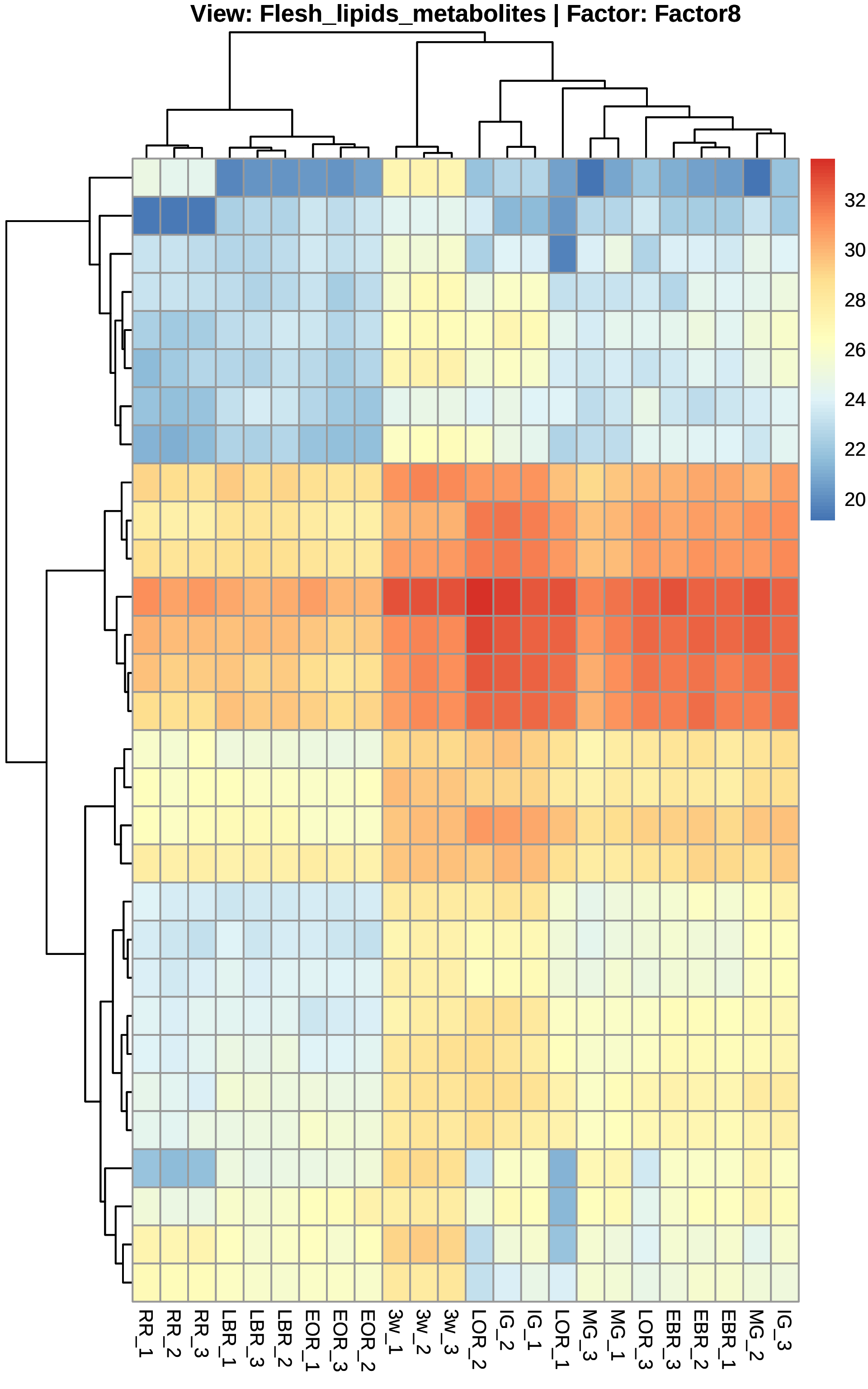
View: Flesh_lipids_metabolites | Factor: Factor8
RR_1
RR_2
RR_3
LBR_1
LBR_3
LBR_2
EOR_1
EOR_3
EOR_2
3w_1
3w_2
3w_3
LOR_2
IG_2
IG_1
LOR_1
MG_3
MG_1
LOR_3
EBR_3
EBR_2
EBR_1
MG_2
IG_3
32
30
28
26
24
22
20

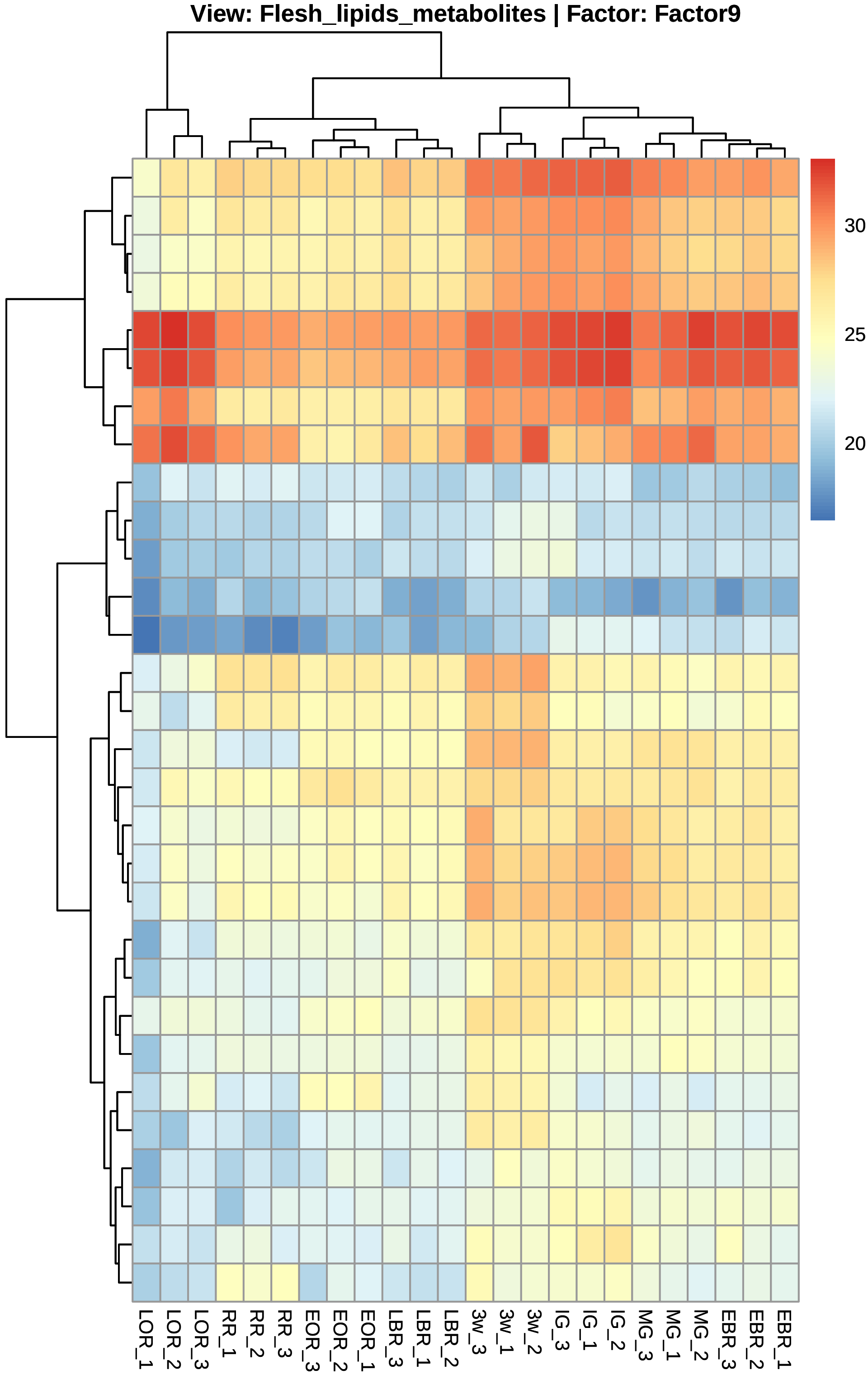

View: Flesh_lipids_metabolites | Factor: Factor9
30
25
20
LOR_1
LOR_2
LOR_3
RR_1
RR_2
RR_3
EOR_3
EOR_2
EOR_1
LBR_3
LBR_1
LBR_2
3w_3
3w_1
3w_2
IG_3
IG_1
IG_2
MG_3
MG_1
MG_2
EBR_3
EBR_2
EBR_1

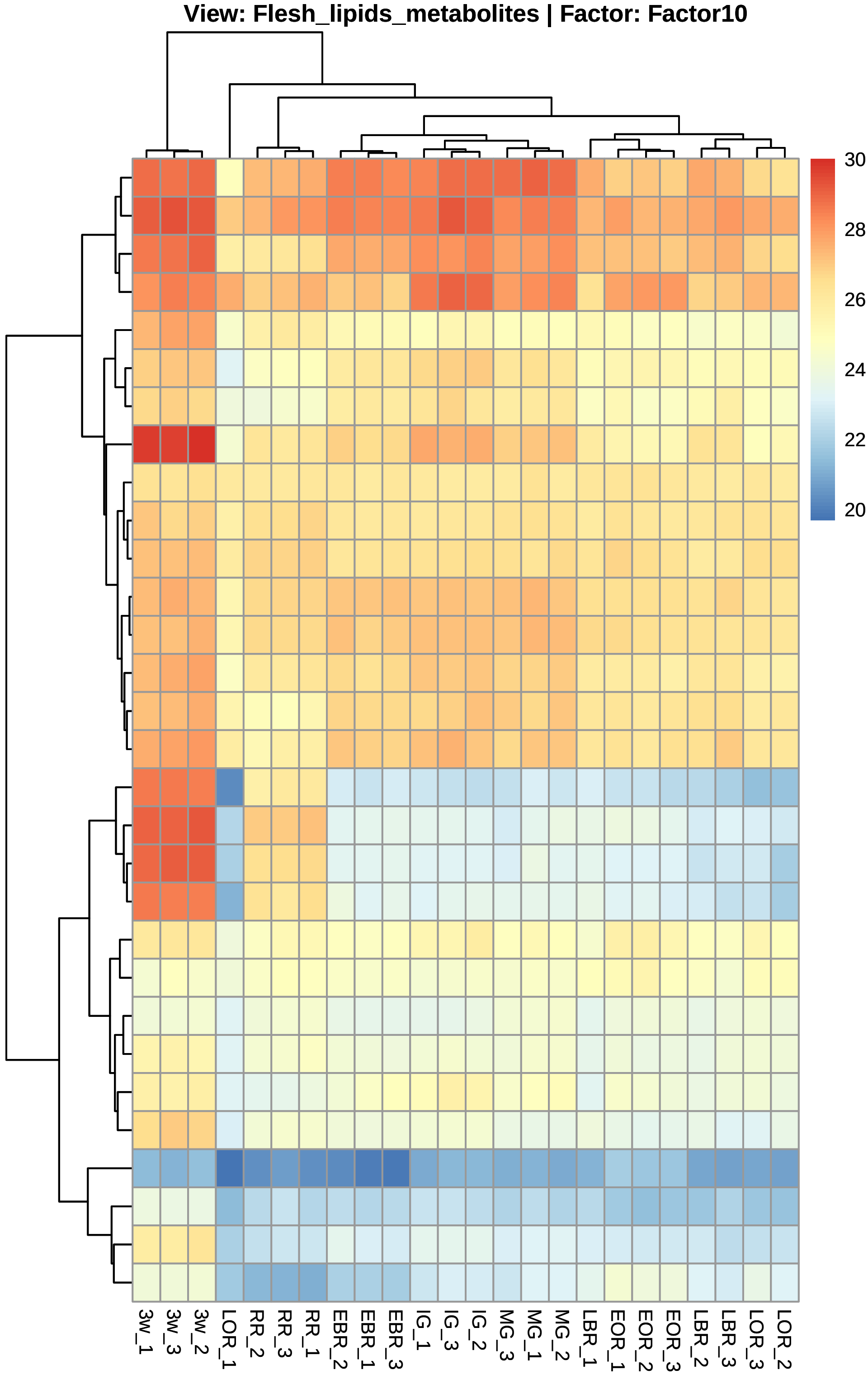

View: Flesh_lipids_metabolites | Factor: Factor10
30
28
26
24
22
20
3w_1
3w_3
3w_2
LOR_1
RR_2
RR_3
RR_1
EBR_2
EBR_1
EBR_3
IG_1
IG_3
IG_2
MG_3
MG_1
MG_2
LBR_1
EOR_1
EOR_2
EOR_3
LBR_2
LBR_3
LOR_3
LOR_2

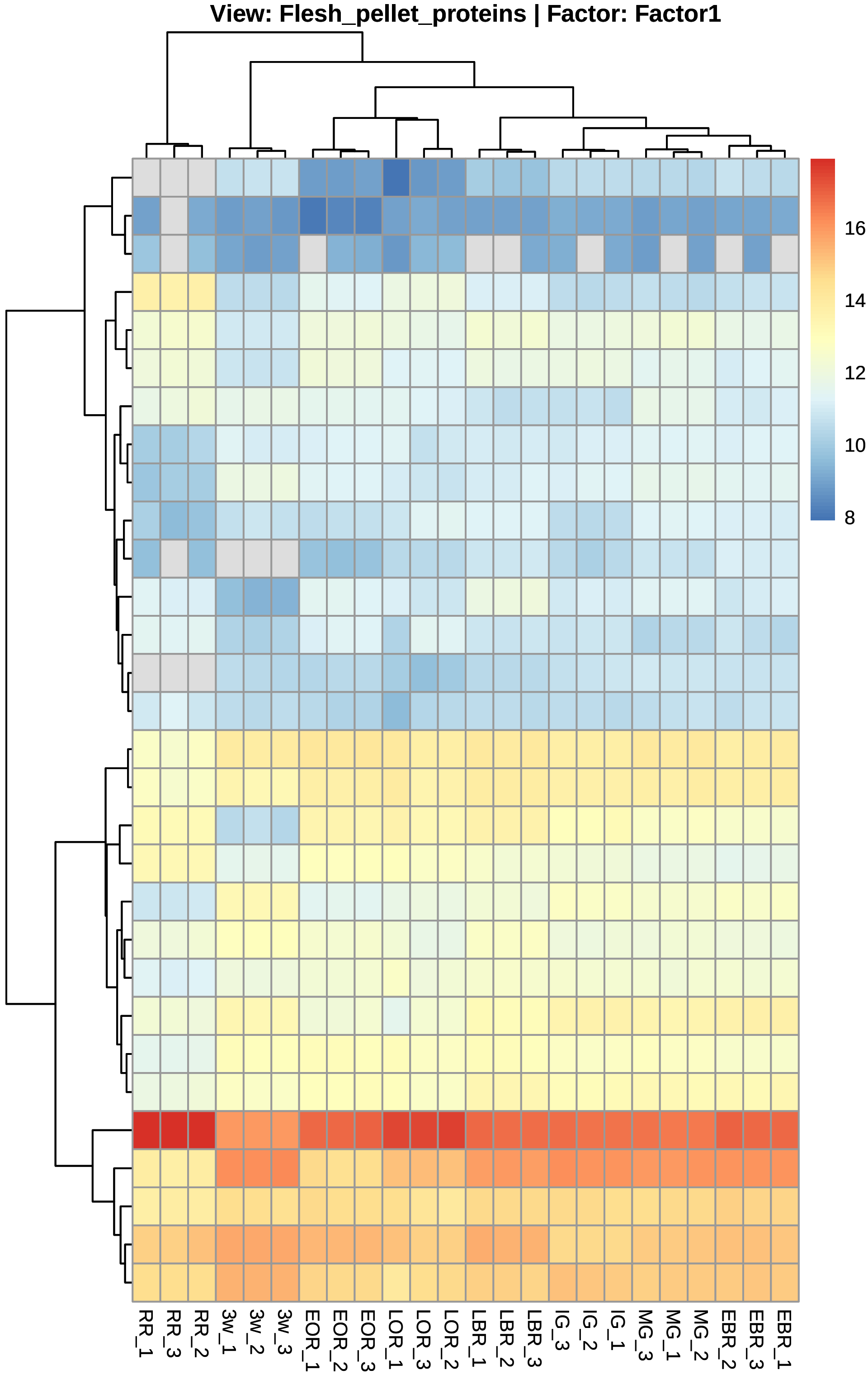

View: Flesh_pellet_proteins | Factor: Factor1
16
14
12
10
8
RR_1
RR_3
RR_2
3w_1
3w_2
3w_3
EOR_1
EOR_2
EOR_3
LOR_1
LOR_3
LOR_2
LBR_1
LBR_2
LBR_3
IG_3
IG_2
IG_1
MG_3
MG_1
MG_2
EBR_2
EBR_3
EBR_1

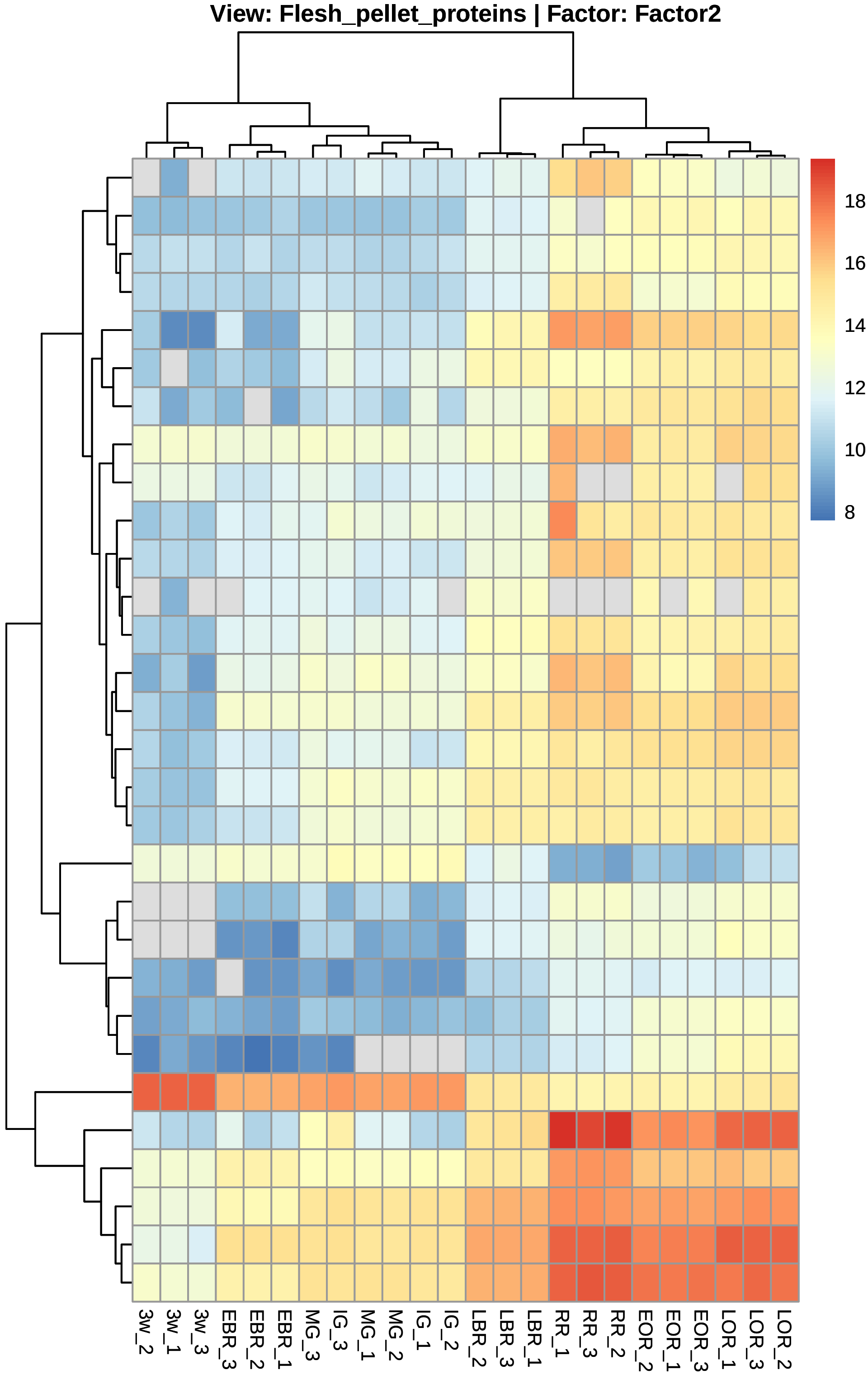

View: Flesh_pellet_proteins | Factor: Factor2
18
16
14
12
10
8
3w_2
3w_1
3w_3
EBR_3
EBR_2
EBR_1
MG_3
IG_3
MG_1
MG_2
IG_1
IG_2
LBR_2
LBR_3
LBR_1
RR_1
RR_3
RR_2
EOR_2
EOR_1
EOR_3
LOR_1
LOR_3
LOR_2

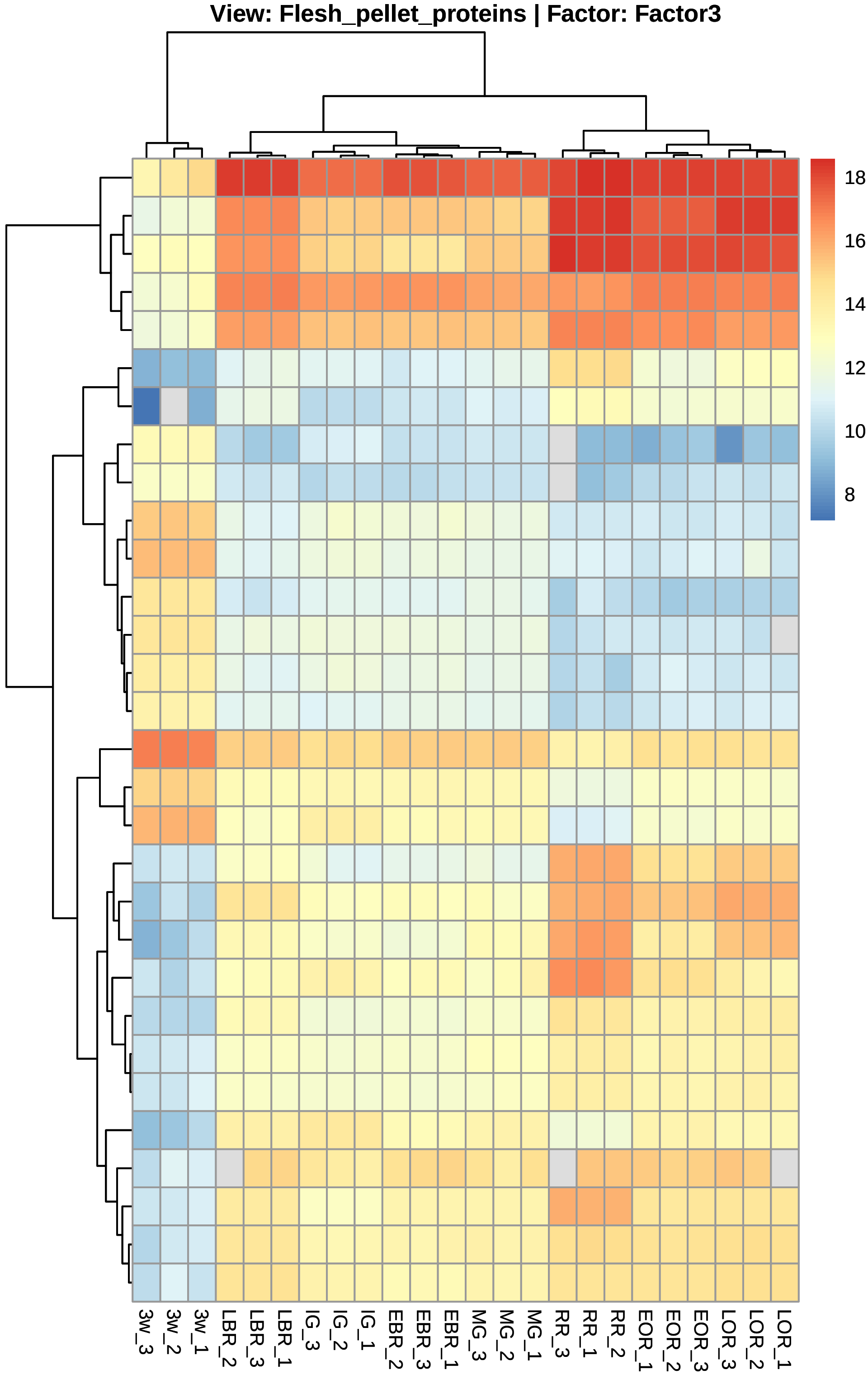
View: Flesh_pellet_proteins | Factor: Factor3
18
16
14
12
10
8
3w_3
3w_2
3w_1
LBR_2
LBR_3
LBR_1
IG_3
IG_2
IG_1
EBR_2
EBR_3
EBR_1
MG_3
MG_2
MG_1
RR_3
RR_1
RR_2
EOR_1
EOR_2
EOR_3
LOR_3
LOR_2
LOR_1

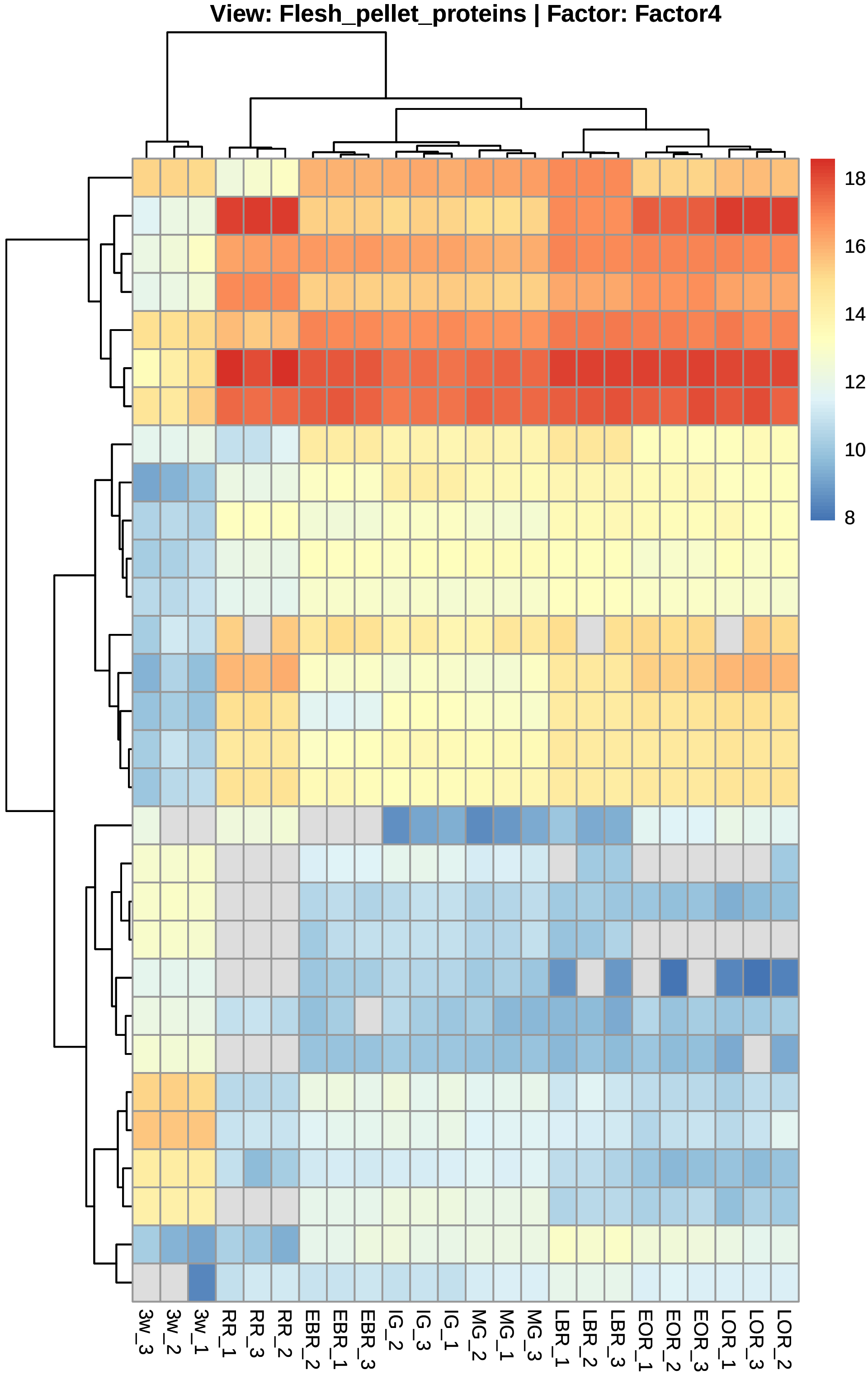
View: Flesh_pellet_proteins | Factor: Factor4
18
16
14
12
10
8
3w_3
3w_2
3w_1
RR_1
RR_3
RR_2
EBR_2
EBR_1
EBR_3
IG_2
IG_3
IG_1
MG_2
MG_1
MG_3
LBR_1
LBR_2
LBR_3
EOR_1
EOR_2
EOR_3
LOR_1
LOR_3
LOR_2

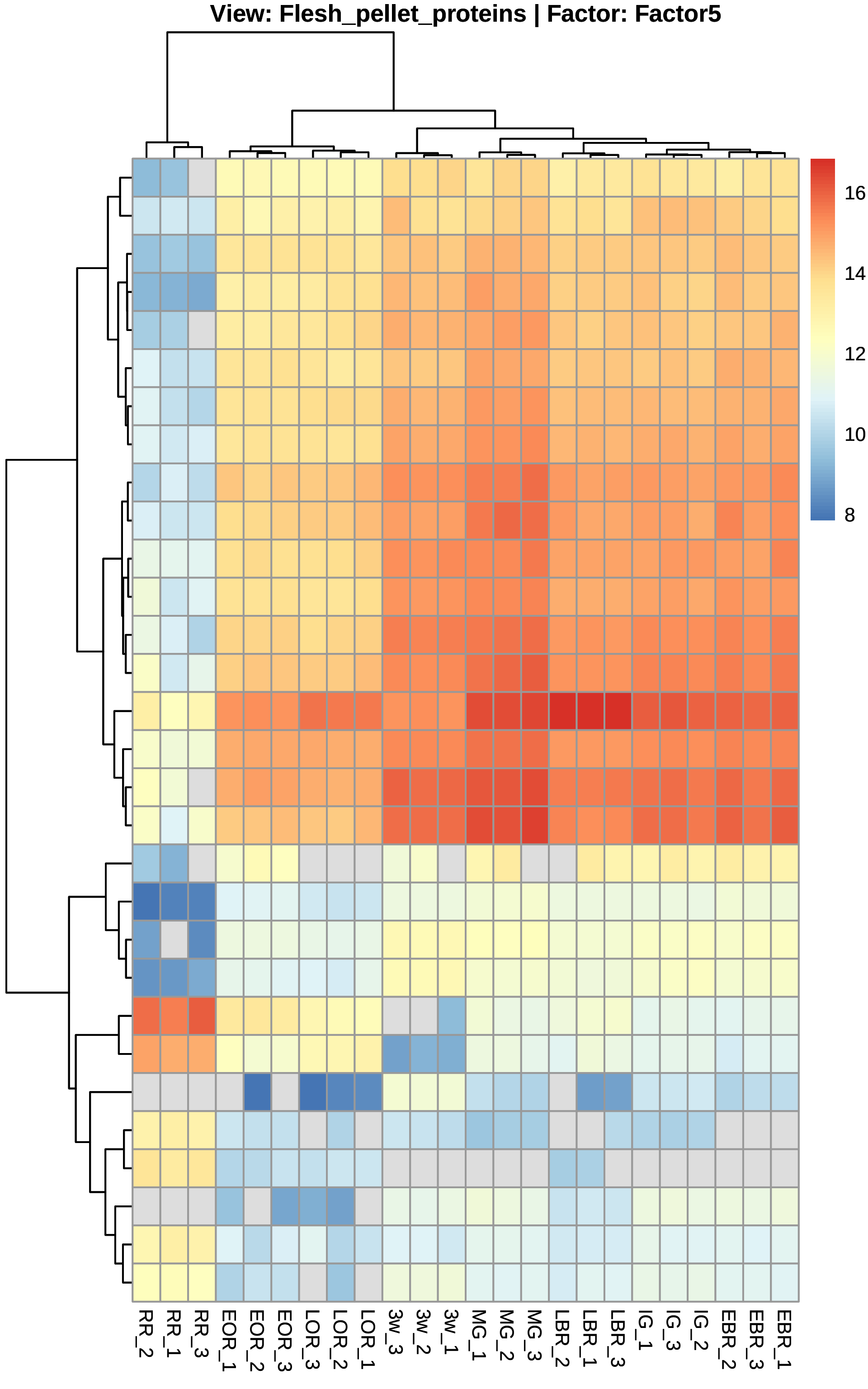
View: Flesh_pellet_proteins | Factor: Factor5
16
14
12
10
8
RR_2
RR_1
RR_3
EOR_1
EOR_2
EOR_3
LOR_3
LOR_2
LOR_1
3w_3
3w_2
3w_1
MG_1
MG_2
MG_3
LBR_2
LBR_1
LBR_3
IG_1
IG_3
IG_2
EBR_2
EBR_3
EBR_1

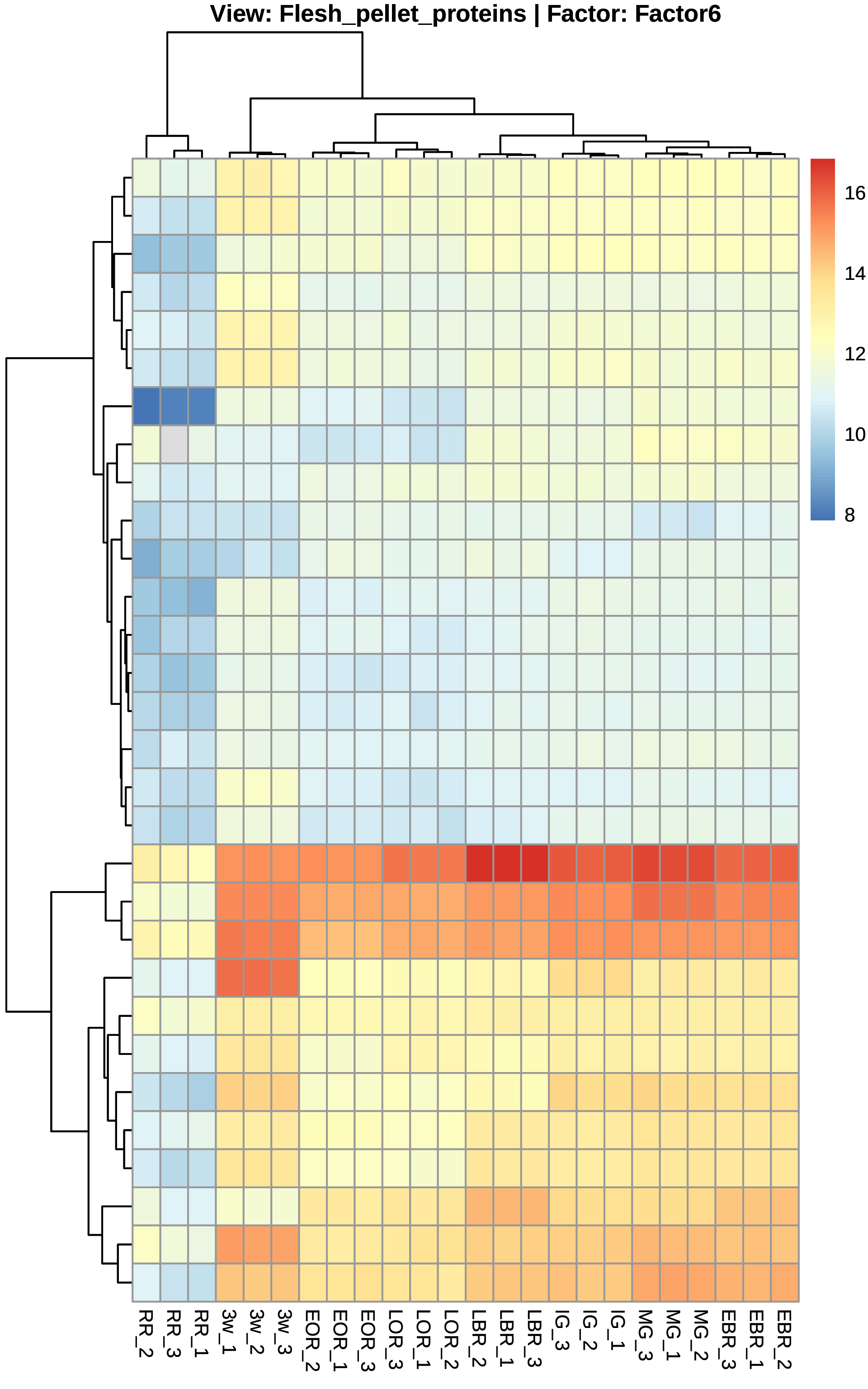
View: Flesh_pellet_proteins | Factor: Factor6
16
14
12
10
8
RR_2
RR_3
RR_1
3w_1
3w_2
3w_3
EOR_2
EOR_1
EOR_3
LOR_3
LOR_1
LOR_2
LBR_2
LBR_1
LBR_3
IG_3
IG_2
IG_1
MG_3
MG_1
MG_2
EBR_3
EBR_1
EBR_2

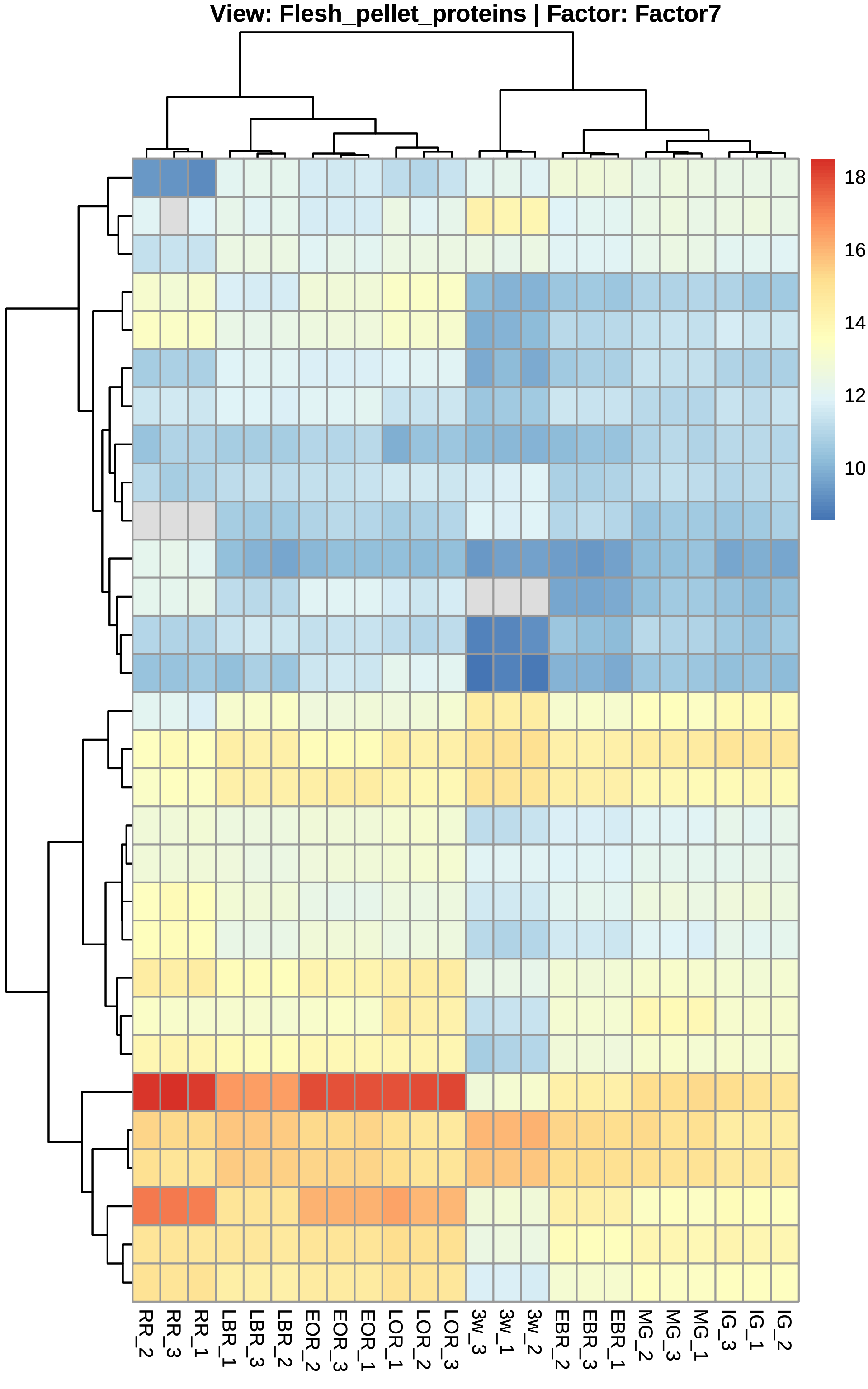

View: Flesh_pellet_proteins | Factor: Factor7
18
16
14
12
10
RR_2
RR_3
RR_1
LBR_1
LBR_3
LBR_2
EOR_2
EOR_3
EOR_1
LOR_1
LOR_2
LOR_3
3w_3
3w_1
3w_2
EBR_2
EBR_3
EBR_1
MG_2
MG_3
MG_1
IG_3
IG_1
IG_2

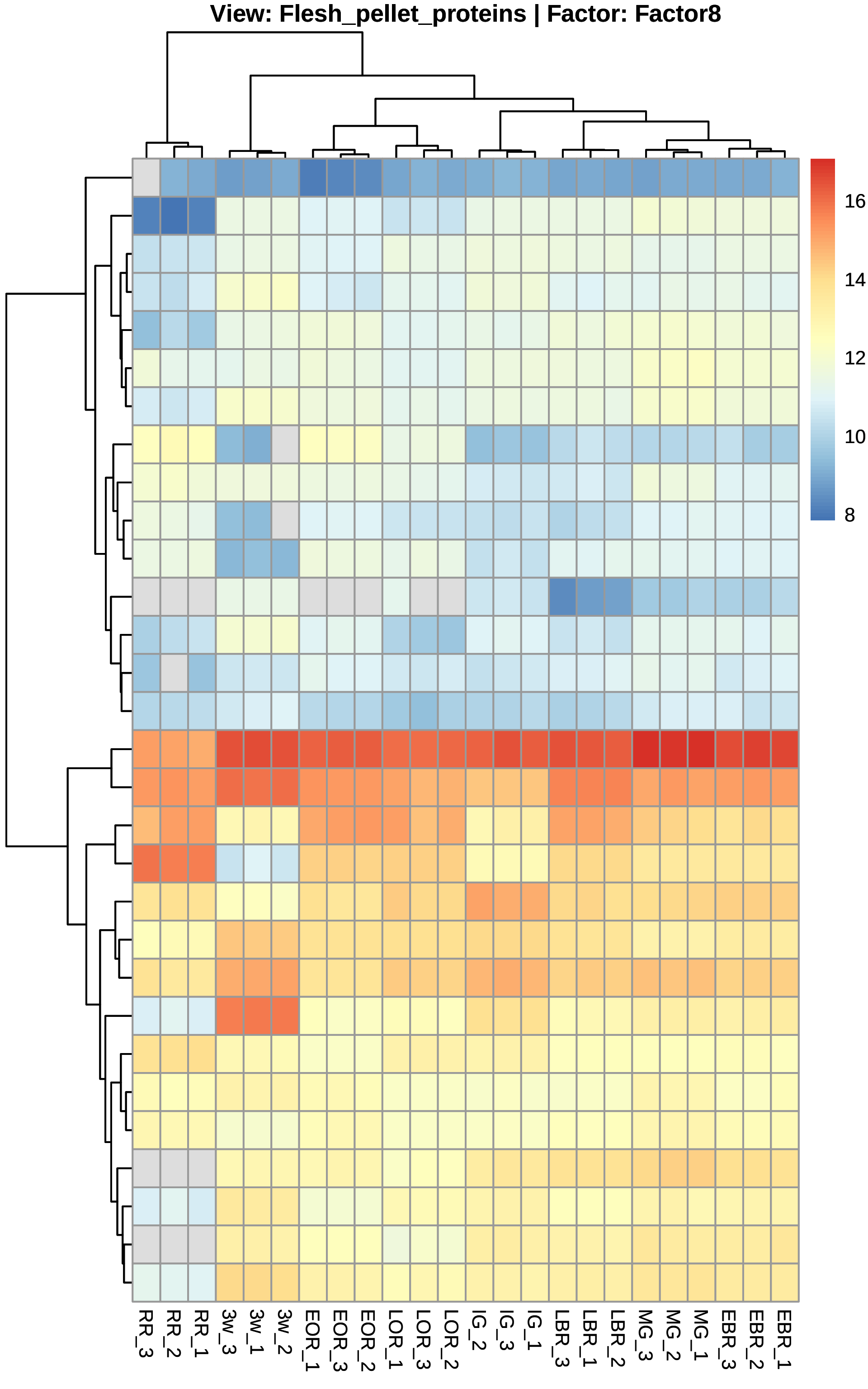
View: Flesh_pellet_proteins | Factor: Factor8
16
14
12
10
8
RR_3
RR_2
RR_1
3w_3
3w_1
3w_2
EOR_1
EOR_3
EOR_2
LOR_1
LOR_3
LOR_2
IG_2
IG_3
IG_1
LBR_3
LBR_1
LBR_2
MG_3
MG_2
MG_1
EBR_3
EBR_2
EBR_1

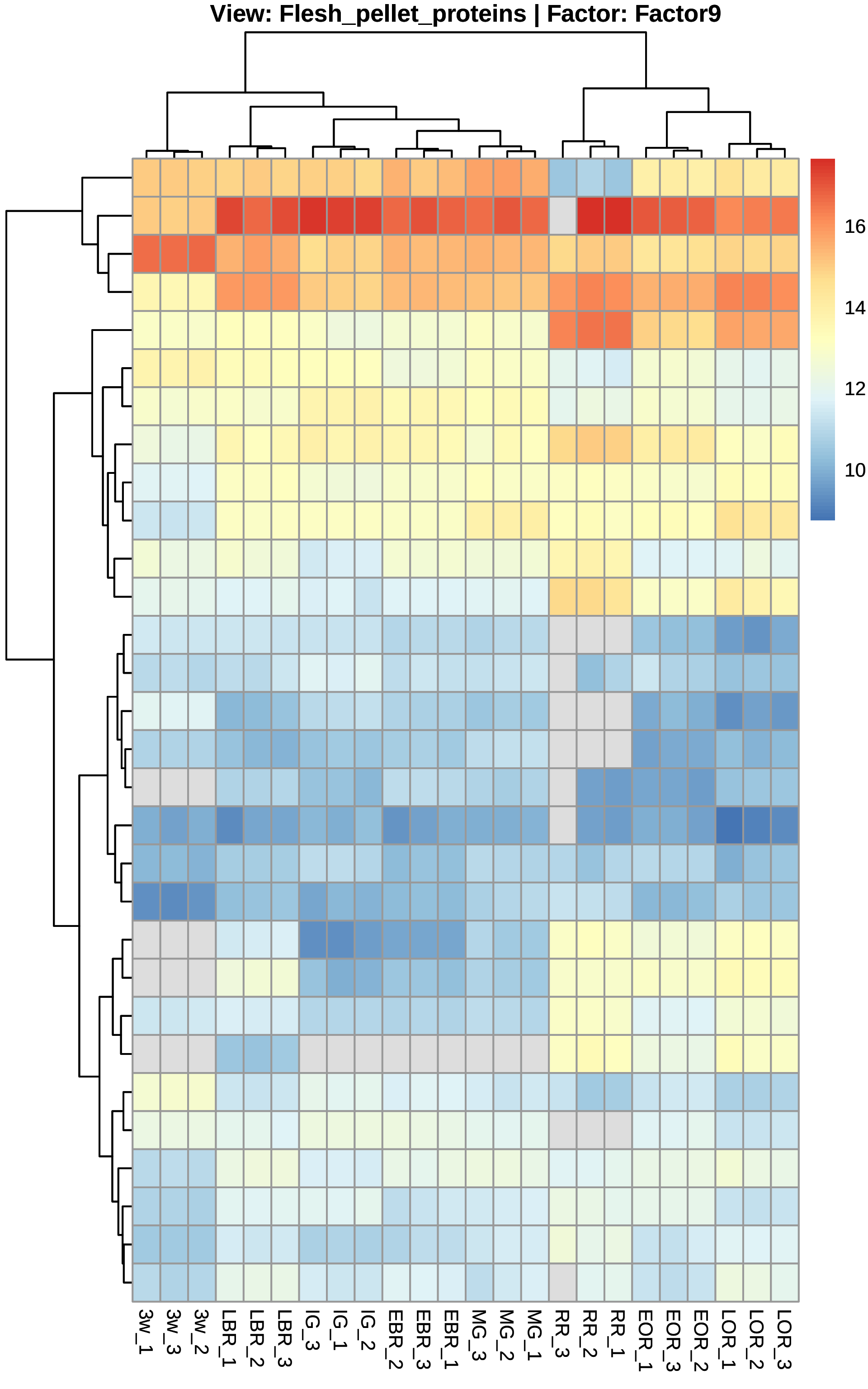

View: Flesh_pellet_proteins | Factor: Factor9
16
14
12
10
3w_1
3w_3
3w_2
LBR_1
LBR_2
LBR_3
IG_3
IG_1
IG_2
EBR_2
EBR_3
EBR_1
MG_3
MG_2
MG_1
RR_3
RR_2
RR_1
EOR_1
EOR_3
EOR_2
LOR_1
LOR_2
LOR_3

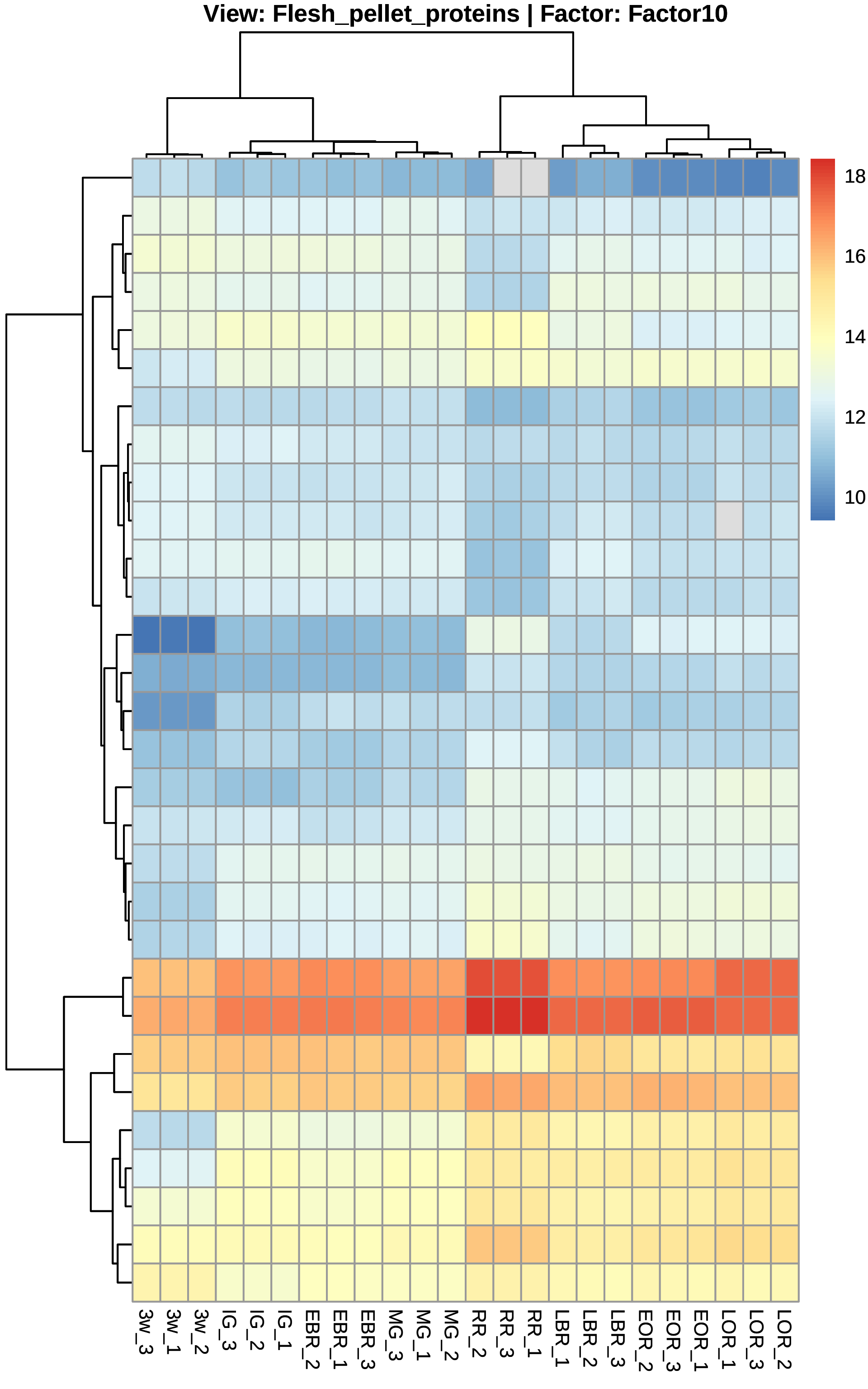
View: Flesh_pellet_proteins | Factor: Factor10
18
16
14
12
10
3w_3
3w_1
3w_2
IG_3
IG_2
IG_1
EBR_2
EBR_1
EBR_3
MG_3
MG_1
MG_2
RR_2
RR_3
RR_1
LBR_1
LBR_2
LBR_3
EOR_2
EOR_3
EOR_1
LOR_1
LOR_3
LOR_2

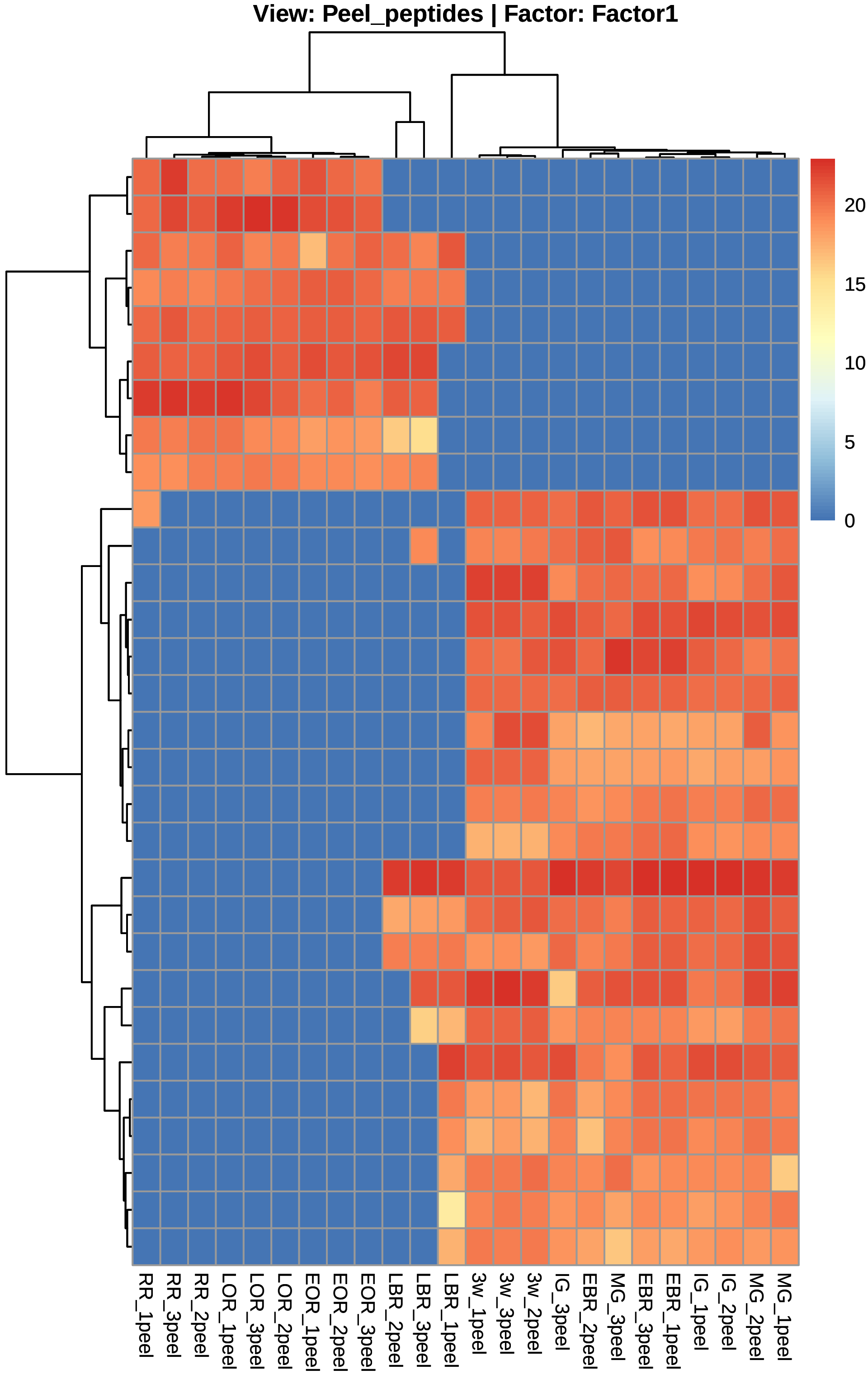
View: Peel_peptides | Factor: Factor1
20
15
10
5
0
RR_1peel
RR_3peel
RR_2peel
LOR_1peel
LOR_3peel
LOR_2peel
EOR_1peel
EOR_2peel
EOR_3peel
LBR_2peel
LBR_3peel
LBR_1peel
3w_1peel
3w_3peel
3w_2peel
IG_3peel
EBR_2peel
MG_3peel
EBR_3peel
EBR_1peel
IG_1peel
IG_2peel
MG_2peel
MG_1peel

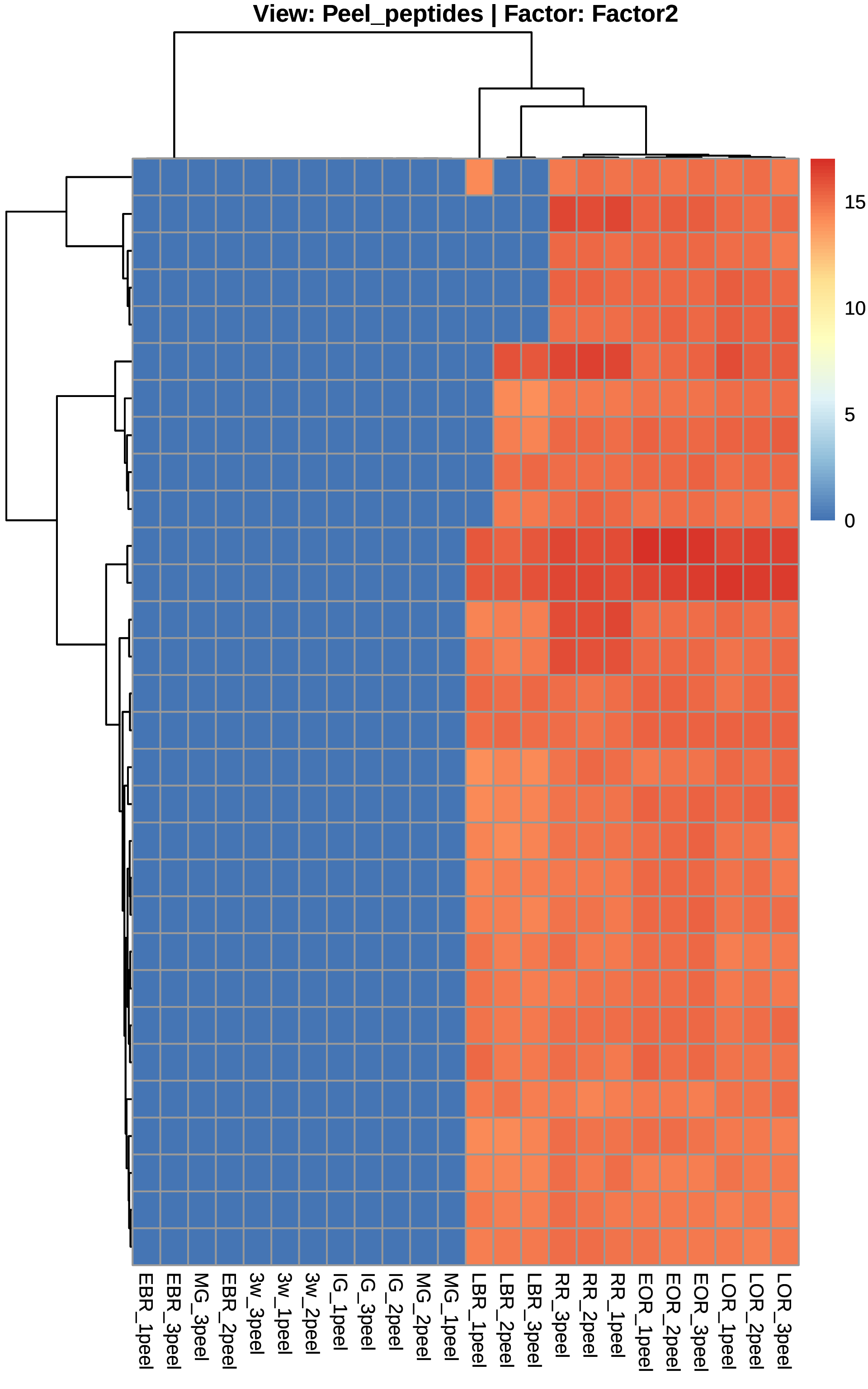
View: Peel_peptides | Factor: Factor2
EBR_1peel
EBR_3peel
MG_3peel
EBR_2peel
3w_3peel
3w_1peel
3w_2peel
IG_1peel
IG_3peel
IG_2peel
MG_2peel
MG_1peel
LBR_1peel
LBR_2peel
LBR_3peel
RR_3peel
RR_2peel
RR_1peel
EOR_1peel
EOR_2peel
EOR_3peel
LOR_1peel
LOR_2peel
LOR_3peel
15
10
5
0

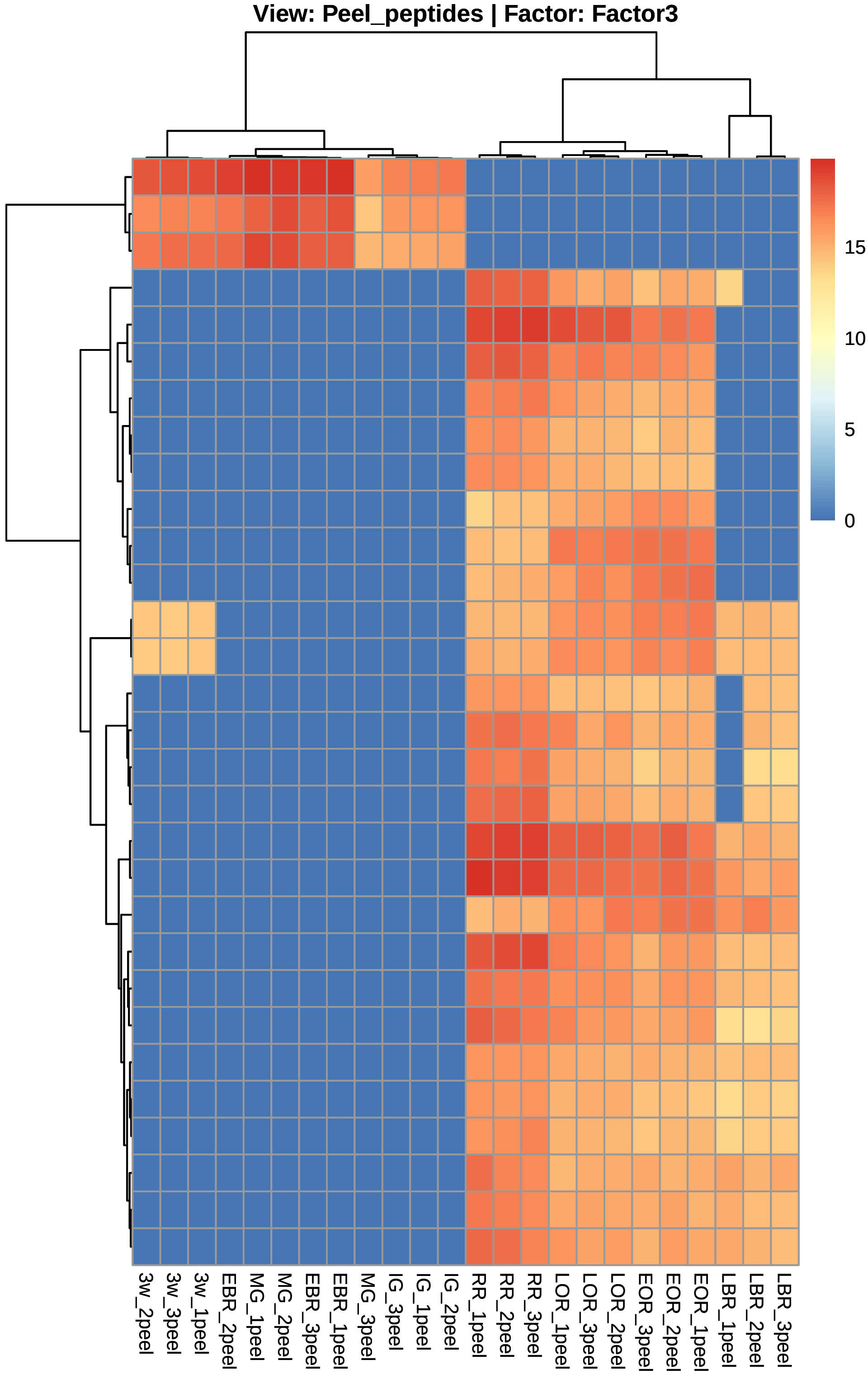

View: Peel_peptides | Factor: Factor3
15
10
5
0
3w_2peel
3w_3peel
3w_1peel
EBR_2peel
MG_1peel
MG_2peel
EBR_3peel
EBR_1peel
MG_3peel
IG_3peel
IG_1peel
IG_2peel
RR_1peel
RR_2peel
RR_3peel
LOR_1peel
LOR_3peel
LOR_2peel
EOR_3peel
EOR_2peel
EOR_1peel
LBR_1peel
LBR_2peel
LBR_3peel

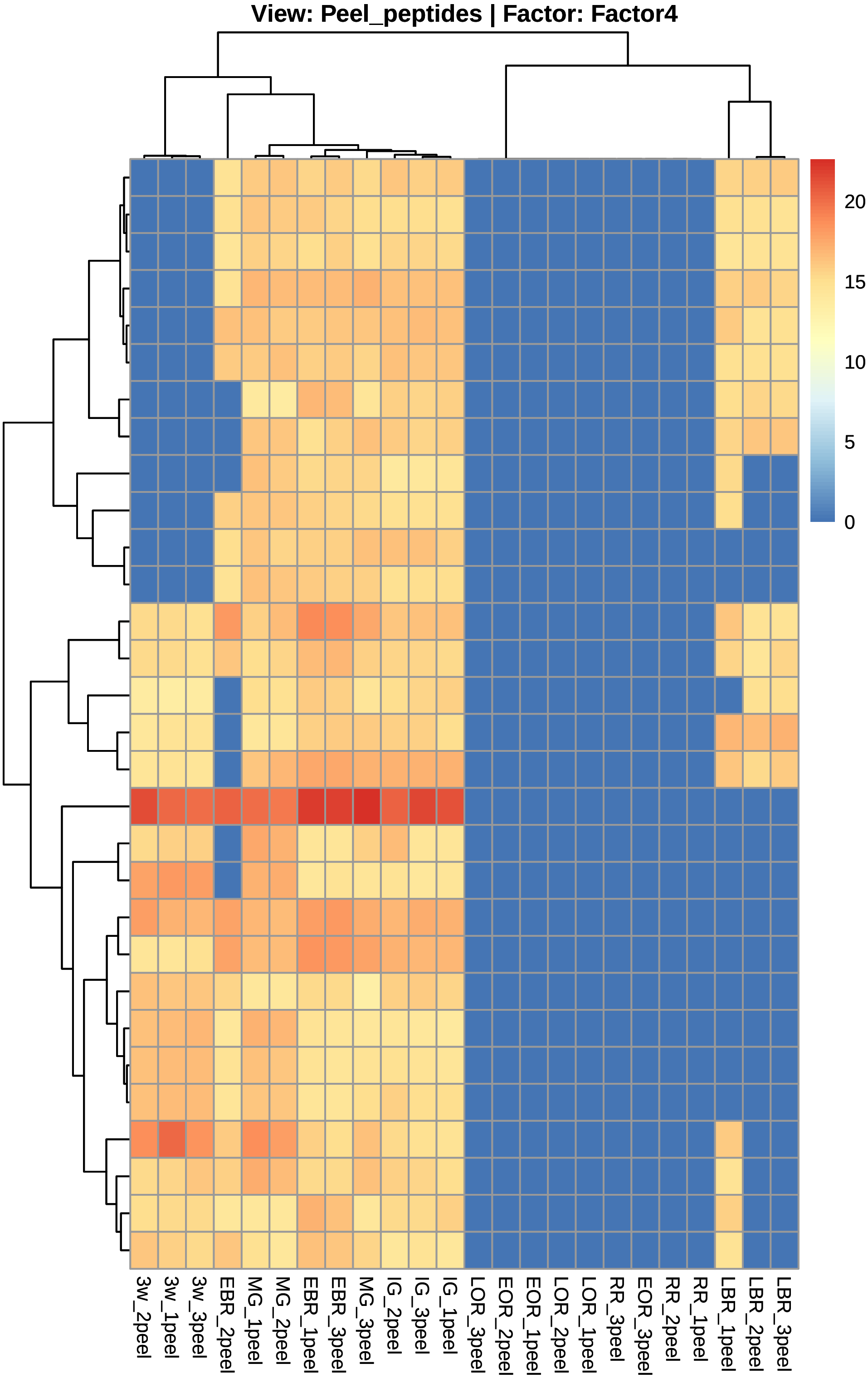
View: Peel_peptides | Factor: Factor4
20
15
10
5
0
3w_2peel
3w_1peel
3w_3peel
EBR_2peel
MG_1peel
MG_2peel
EBR_1peel
EBR_3peel
MG_3peel
IG_2peel
IG_3peel
IG_1peel
LOR_3peel
EOR_2peel
EOR_1peel
LOR_2peel
LOR_1peel
RR_3peel
EOR_3peel
RR_2peel
RR_1peel
LBR_1peel
LBR_2peel
LBR_3peel

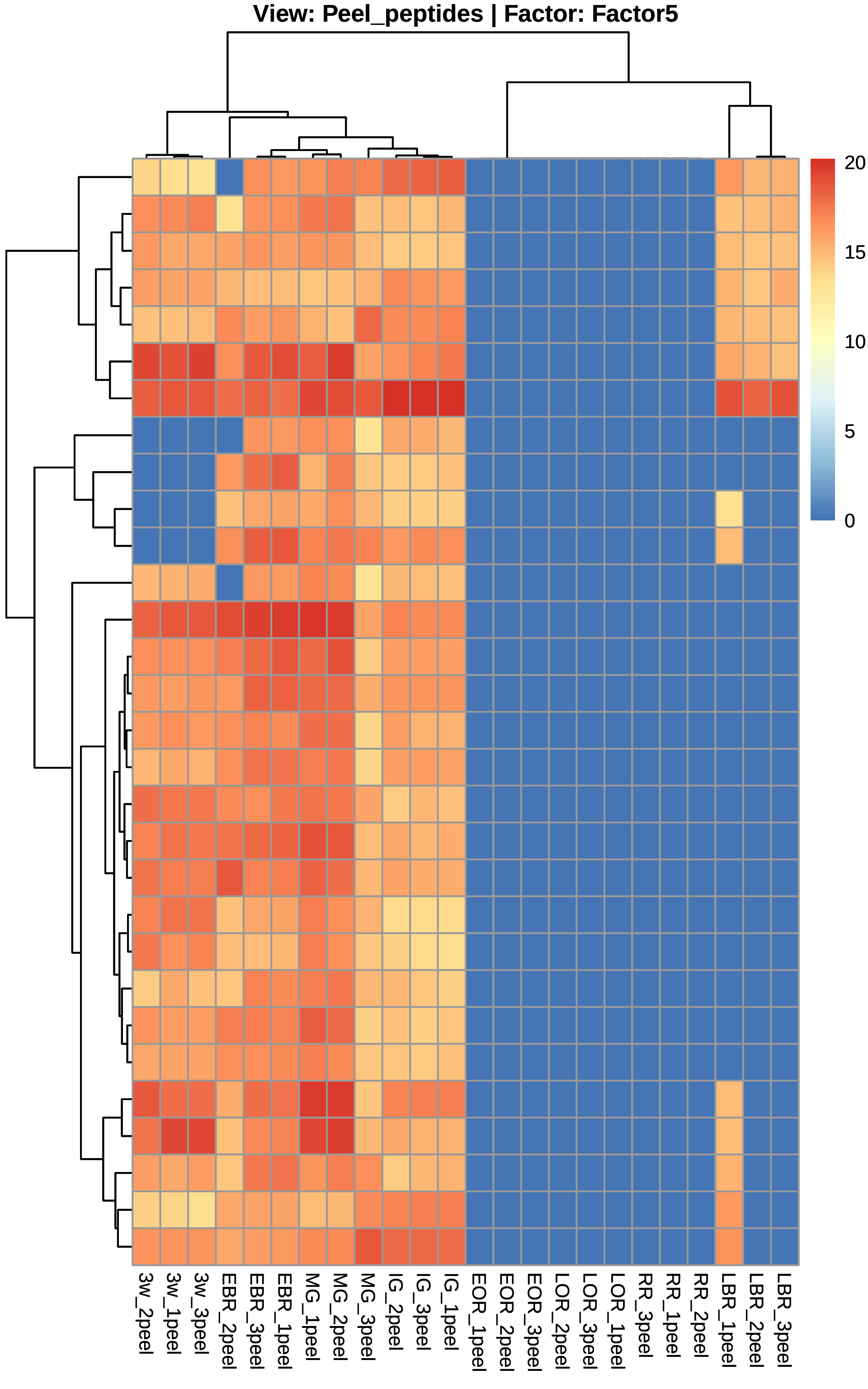
View: Peel_peptides | Factor: Factor5
20
15
10
5
0
3w_2peel
3w_1peel
3w_3peel
EBR_2peel
EBR_3peel
EBR_1peel
MG_1peel
MG_2peel
MG_3peel
IG_2peel
IG_3peel
IG_1peel
EOR_1peel
EOR_2peel
EOR_3peel
LOR_2peel
LOR_3peel
LOR_1peel
RR_3peel
RR_1peel
RR_2peel
LBR_1peel
LBR_2peel
LBR_3peel

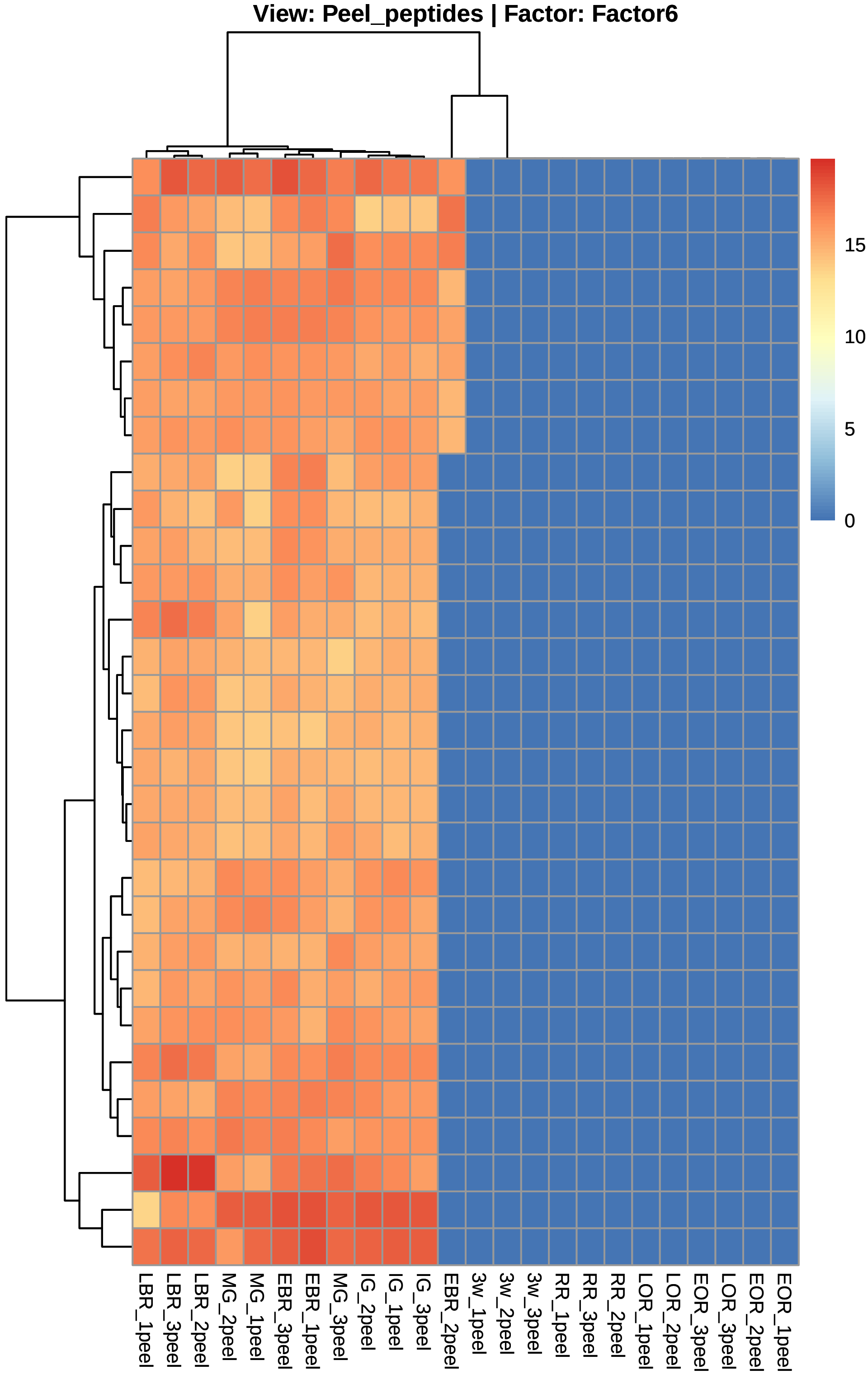
View: Peel_peptides | Factor: Factor6
15
10
5
0
LBR_1peel
LBR_3peel
LBR_2peel
MG_2peel
MG_1peel
EBR_3peel
EBR_1peel
MG_3peel
IG_2peel
IG_1peel
IG_3peel
EBR_2peel
3w_1peel
3w_2peel
3w_3peel
RR_1peel
RR_3peel
RR_2peel
LOR_1peel
LOR_2peel
EOR_3peel
LOR_3peel
EOR_2peel
EOR_1peel

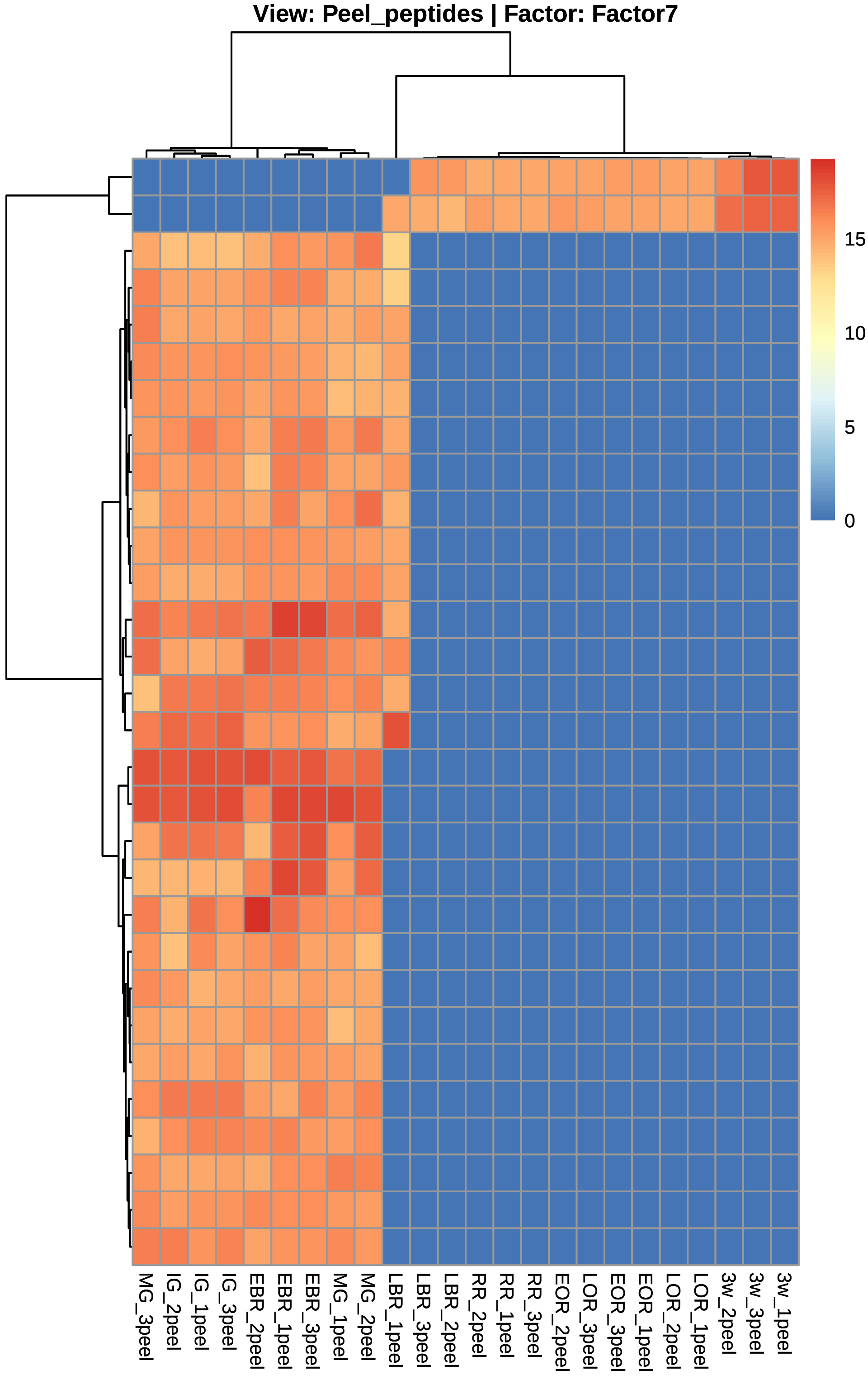
View: Peel_peptides | Factor: Factor7
15
10
5
0
MG_3peel
IG_2peel
IG_1peel
IG_3peel
EBR_2peel
EBR_1peel
EBR_3peel
MG_1peel
MG_2peel
LBR_1peel
LBR_3peel
LBR_2peel
RR_2peel
RR_1peel
RR_3peel
EOR_2peel
LOR_3peel
EOR_3peel
EOR_1peel
LOR_2peel
LOR_1peel
3w_2peel
3w_3peel
3w_1peel

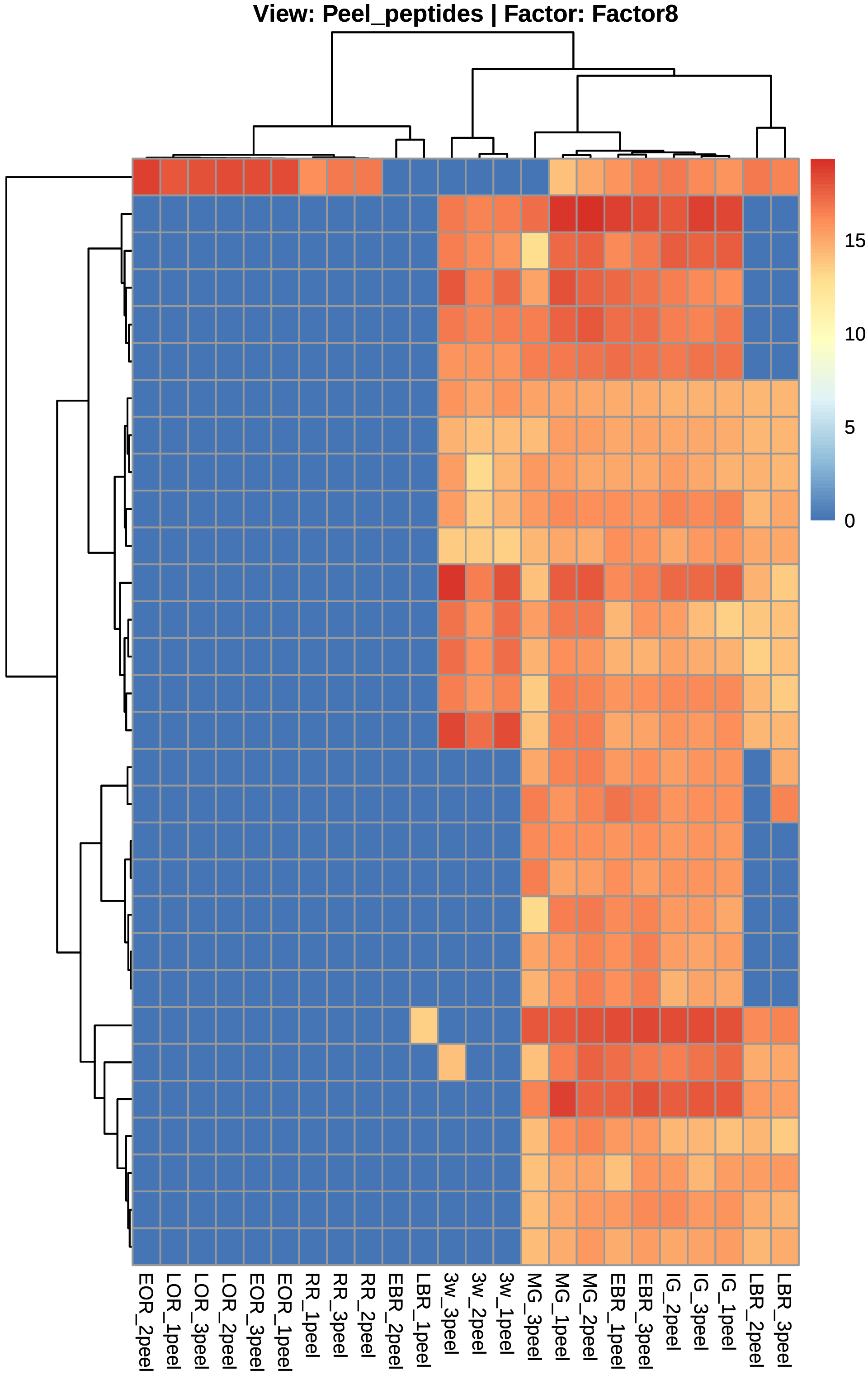
View: Peel_peptides | Factor: Factor8
15
10
5
0
EOR_2peel
LOR_1peel
LOR_3peel
LOR_2peel
EOR_3peel
EOR_1peel
RR_1peel
RR_3peel
RR_2peel
EBR_2peel
LBR_1peel
3w_3peel
3w_2peel
3w_1peel
MG_3peel
MG_1peel
MG_2peel
EBR_1peel
EBR_3peel
IG_2peel
IG_3peel
IG_1peel
LBR_2peel
LBR_3peel

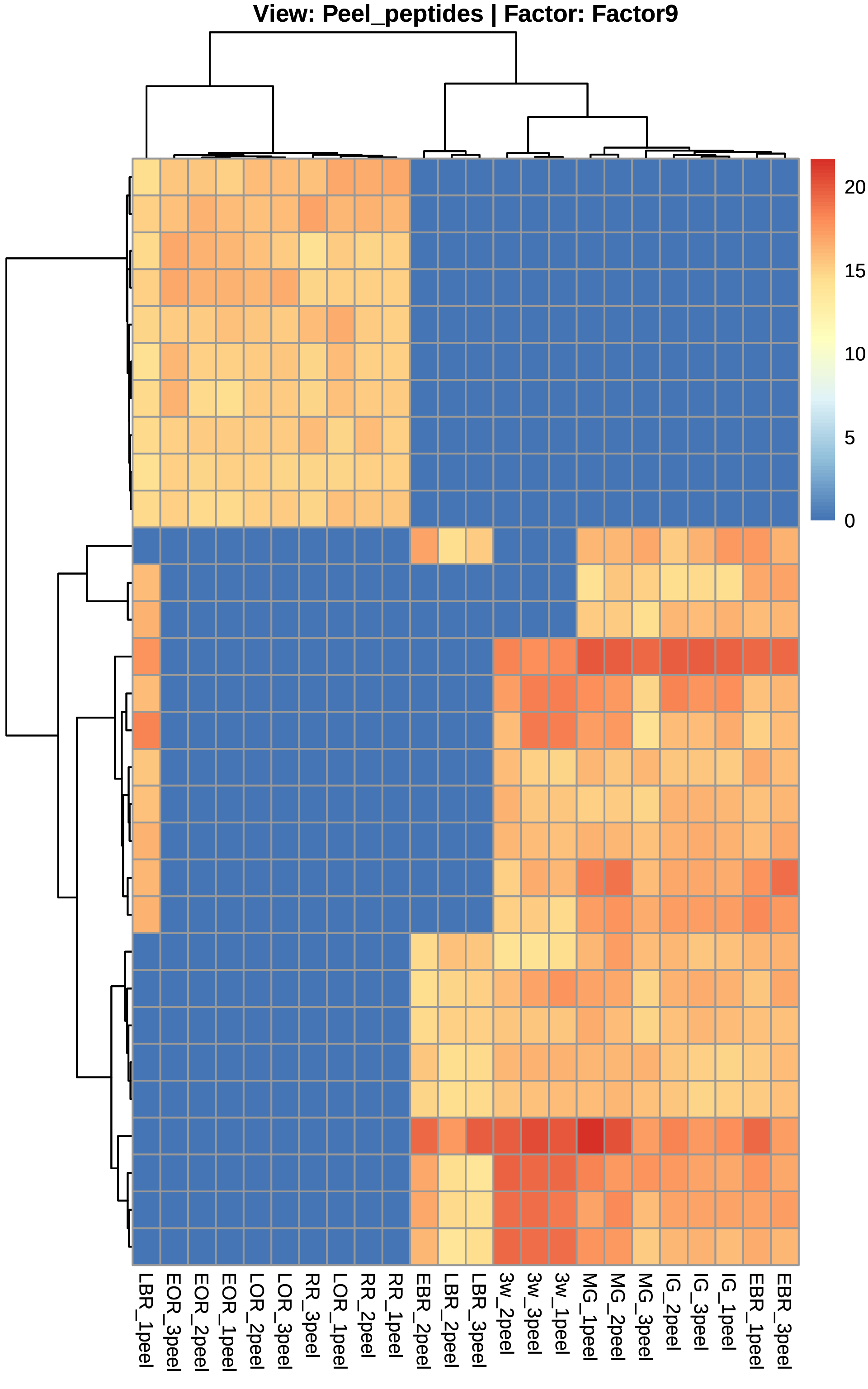

View: Peel_peptides | Factor: Factor9
20
15
10
5
0
LBR_1peel
EOR_3peel
EOR_2peel
EOR_1peel
LOR_2peel
LOR_3peel
RR_3peel
LOR_1peel
RR_2peel
RR_1peel
EBR_2peel
LBR_2peel
LBR_3peel
3w_2peel
3w_3peel
3w_1peel
MG_1peel
MG_2peel
MG_3peel
IG_2peel
IG_3peel
IG_1peel
EBR_1peel
EBR_3peel

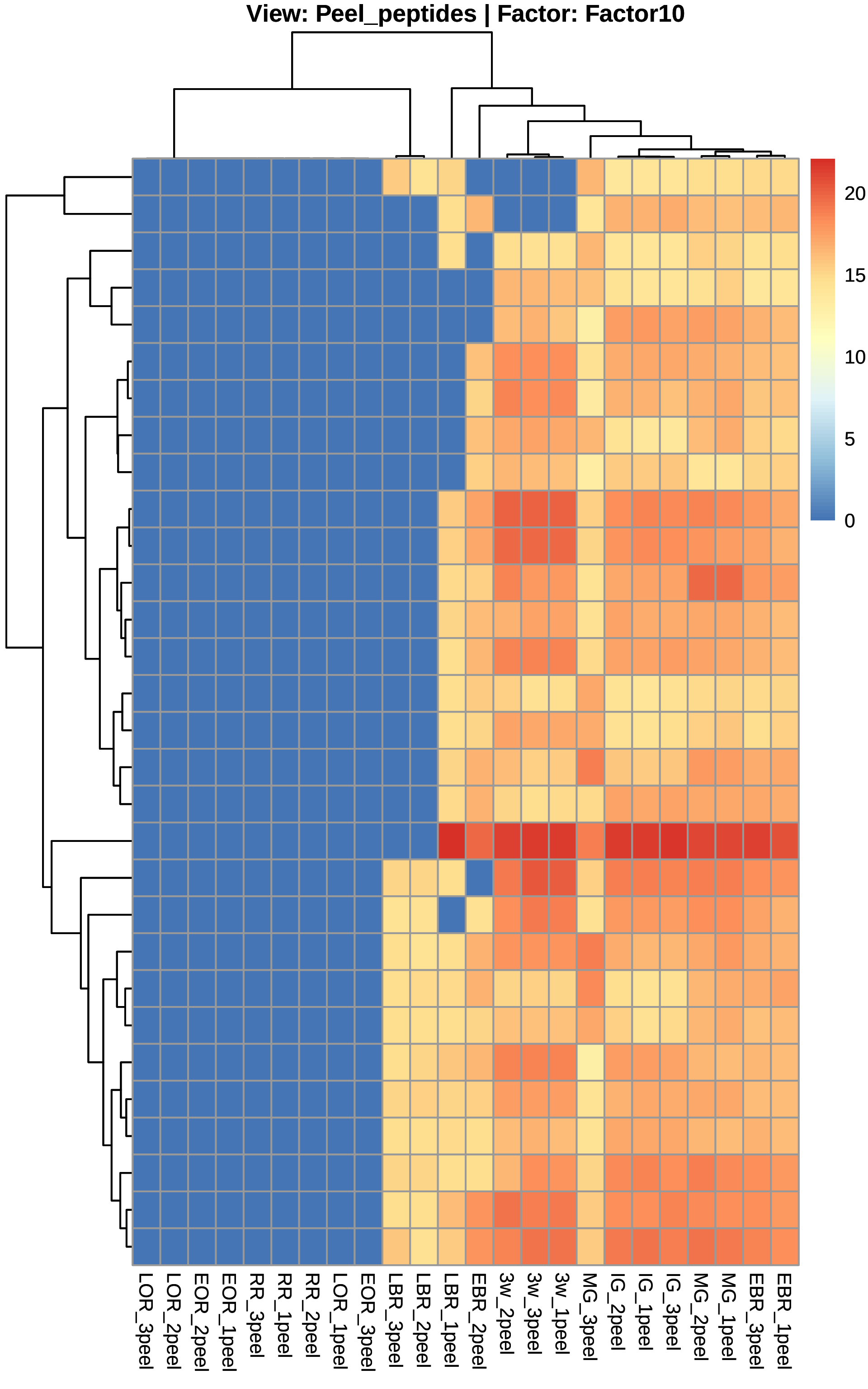
View: Peel_peptides | Factor: Factor10
20
15
10
5
0
LOR_3peel
LOR_2peel
EOR_2peel
EOR_1peel
RR_3peel
RR_1peel
RR_2peel
LOR_1peel
EOR_3peel
LBR_3peel
LBR_2peel
LBR_1peel
EBR_2peel
3w_2peel
3w_3peel
3w_1peel
MG_3peel
IG_2peel
IG_1peel
IG_3peel
MG_2peel
MG_1peel
EBR_3peel
EBR_1peel

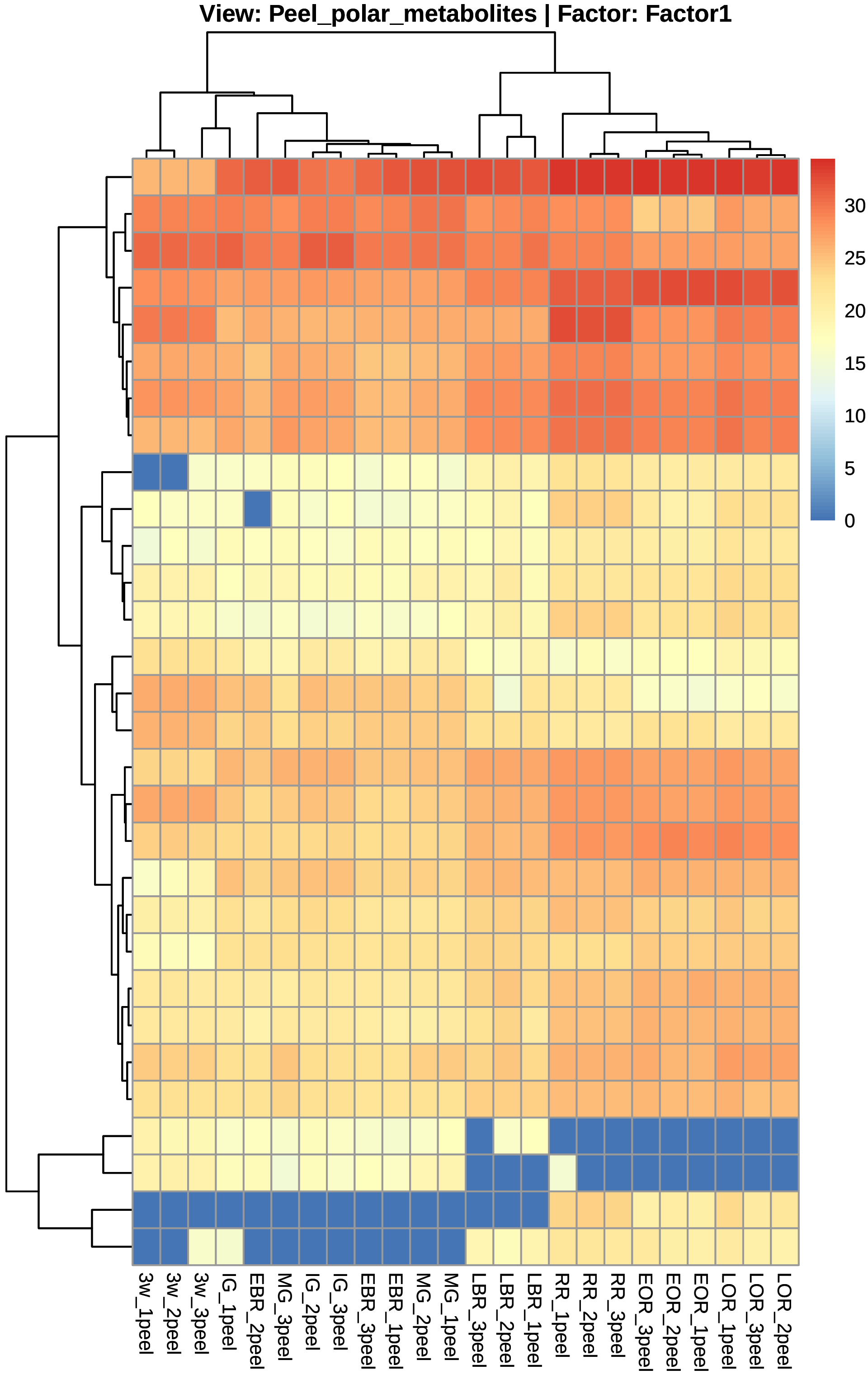
View: Peel_polar_metabolites | Factor: Factor1
30
25
20
15
10
5
0
3w_1peel
3w_2peel
3w_3peel
IG_1peel
EBR_2peel
MG_3peel
IG_2peel
IG_3peel
EBR_3peel
EBR_1peel
MG_2peel
MG_1peel
LBR_3peel
LBR_2peel
LBR_1peel
RR_1peel
RR_2peel
RR_3peel
EOR_3peel
EOR_2peel
EOR_1peel
LOR_1peel
LOR_3peel
LOR_2peel

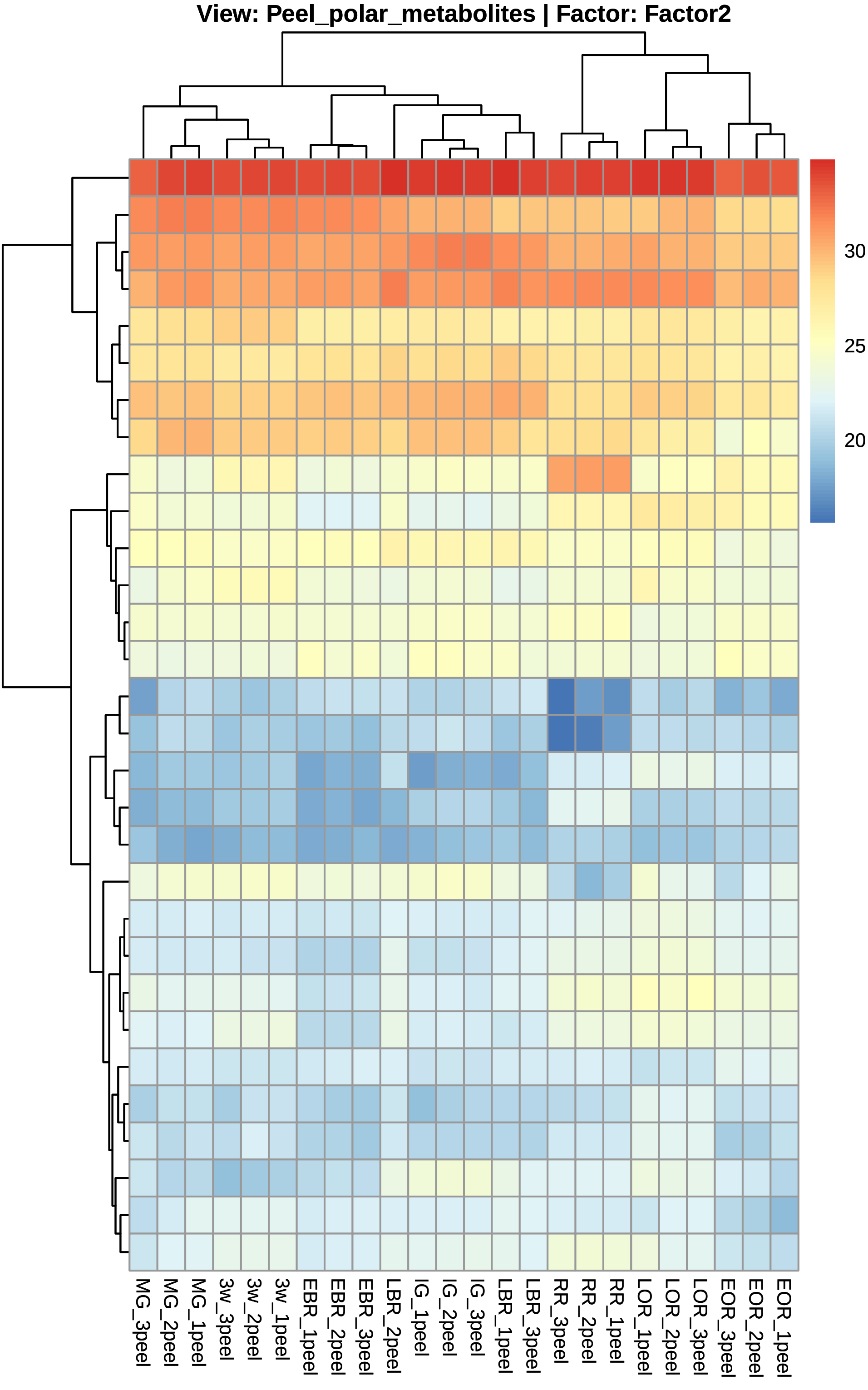

View: Peel_polar_metabolites | Factor: Factor2
30
25
20
MG_3peel
MG_2peel
MG_1peel
3w_3peel
3w_2peel
3w_1peel
EBR_1peel
EBR_2peel
EBR_3peel
LBR_2peel
IG_1peel
IG_2peel
IG_3peel
LBR_1peel
LBR_3peel
RR_3peel
RR_2peel
RR_1peel
LOR_1peel
LOR_2peel
LOR_3peel
EOR_3peel
EOR_2peel
EOR_1peel

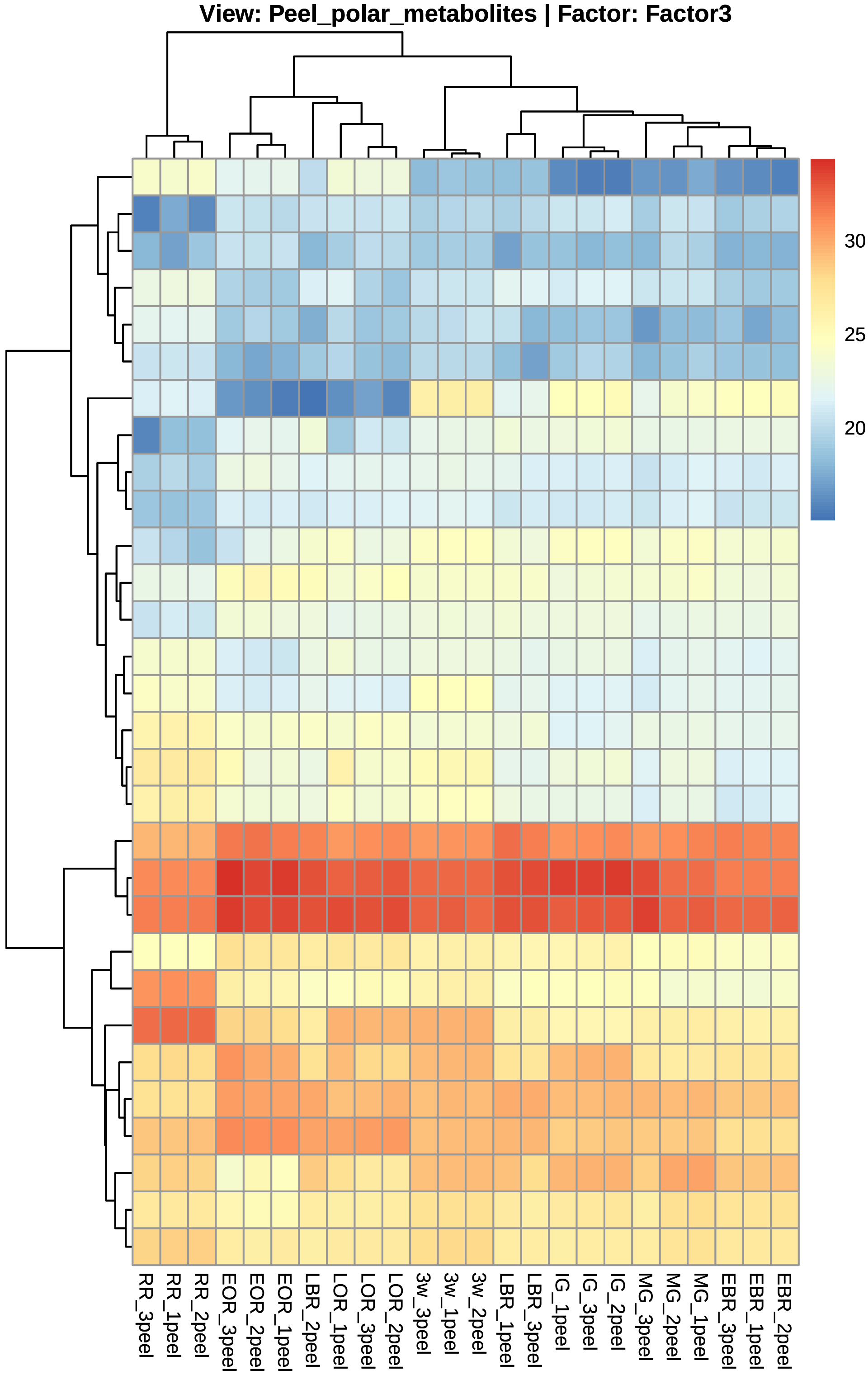
View: Peel_polar_metabolites | Factor: Factor3
30
25
20
RR_3peel
RR_1peel
RR_2peel
EOR_3peel
EOR_2peel
EOR_1peel
LBR_2peel
LOR_1peel
LOR_3peel
LOR_2peel
3w_3peel
3w_1peel
3w_2peel
LBR_1peel
LBR_3peel
IG_1peel
IG_3peel
IG_2peel
MG_3peel
MG_2peel
MG_1peel
EBR_3peel
EBR_1peel
EBR_2peel

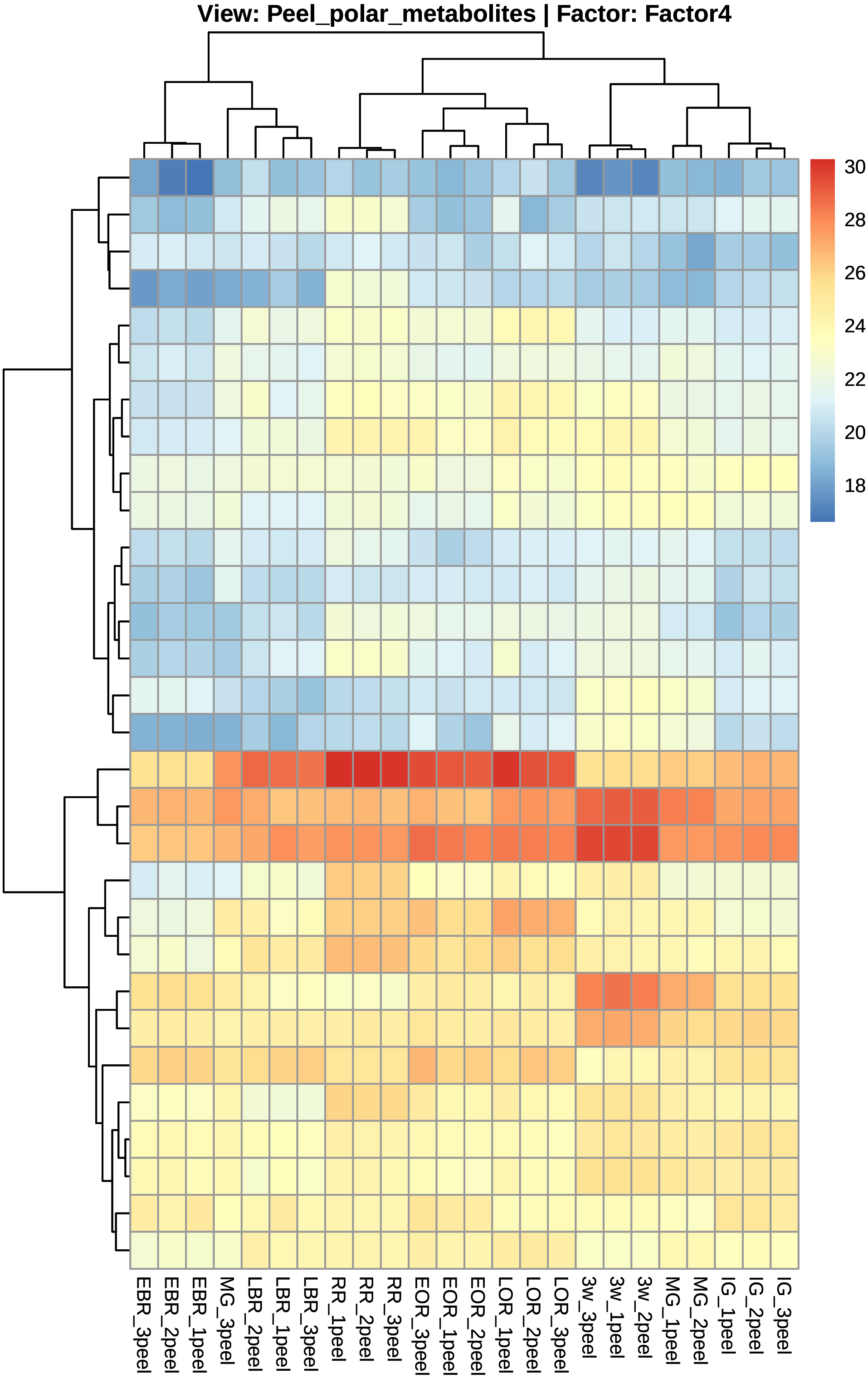

View: Peel_polar_metabolites | Factor: Factor4
30
28
26
24
22
20
18
EBR_3peel
EBR_2peel
EBR_1peel
MG_3peel
LBR_2peel
LBR_1peel
LBR_3peel
RR_1peel
RR_2peel
RR_3peel
EOR_3peel
EOR_1peel
EOR_2peel
LOR_1peel
LOR_2peel
LOR_3peel
3w_3peel
3w_1peel
3w_2peel
MG_1peel
MG_2peel
IG_1peel
IG_2peel
IG_3peel

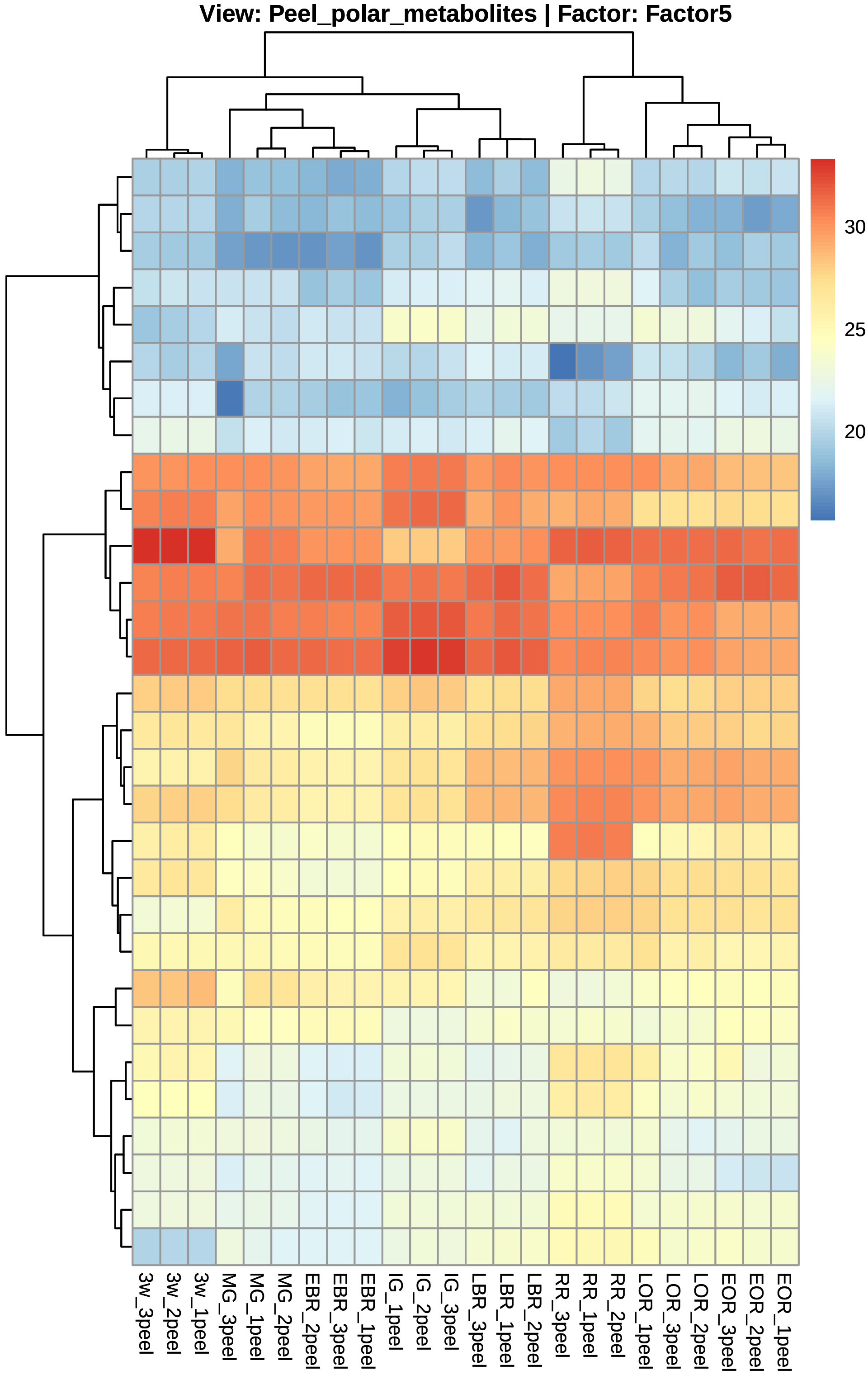
View: Peel_polar_metabolites | Factor: Factor5
30
25
20
3w_3peel
3w_2peel
3w_1peel
MG_3peel
MG_1peel
MG_2peel
EBR_2peel
EBR_3peel
EBR_1peel
IG_1peel
IG_2peel
IG_3peel
LBR_3peel
LBR_1peel
LBR_2peel
RR_3peel
RR_1peel
RR_2peel
LOR_1peel
LOR_3peel
LOR_2peel
EOR_3peel
EOR_2peel
EOR_1peel

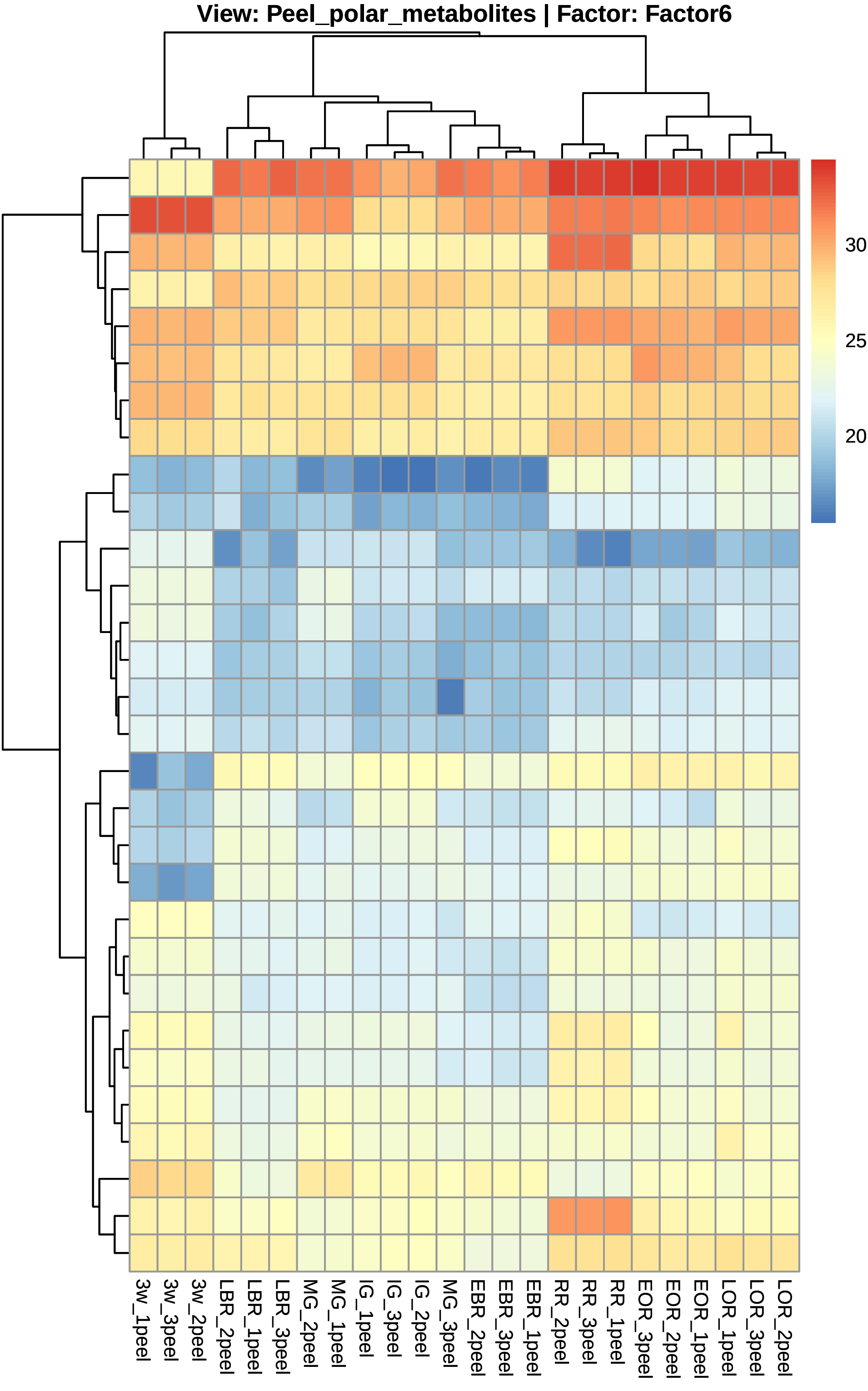
View: Peel_polar_metabolites | Factor: Factor6
30
25
20
3w_1peel
3w_3peel
3w_2peel
LBR_2peel
LBR_1peel
LBR_3peel
MG_2peel
MG_1peel
IG_1peel
IG_3peel
IG_2peel
MG_3peel
EBR_2peel
EBR_3peel
EBR_1peel
RR_2peel
RR_3peel
RR_1peel
EOR_3peel
EOR_2peel
EOR_1peel
LOR_1peel
LOR_3peel
LOR_2peel

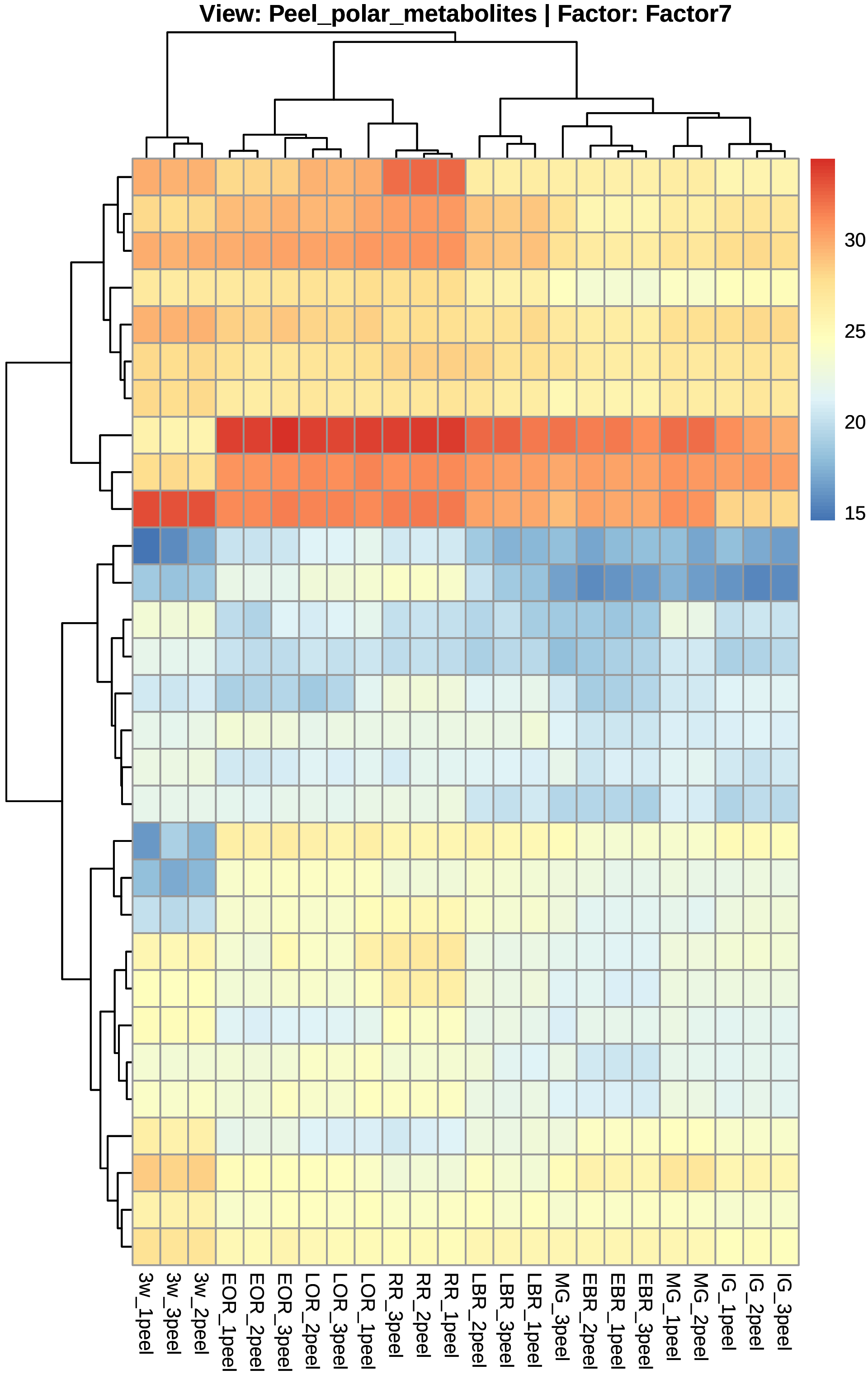

View: Peel_polar_metabolites | Factor: Factor7
30
25
20
15
3w_1peel
3w_3peel
3w_2peel
EOR_1peel
EOR_2peel
EOR_3peel
LOR_2peel
LOR_3peel
LOR_1peel
RR_3peel
RR_2peel
RR_1peel
LBR_2peel
LBR_3peel
LBR_1peel
MG_3peel
EBR_2peel
EBR_1peel
EBR_3peel
MG_1peel
MG_2peel
IG_1peel
IG_2peel
IG_3peel

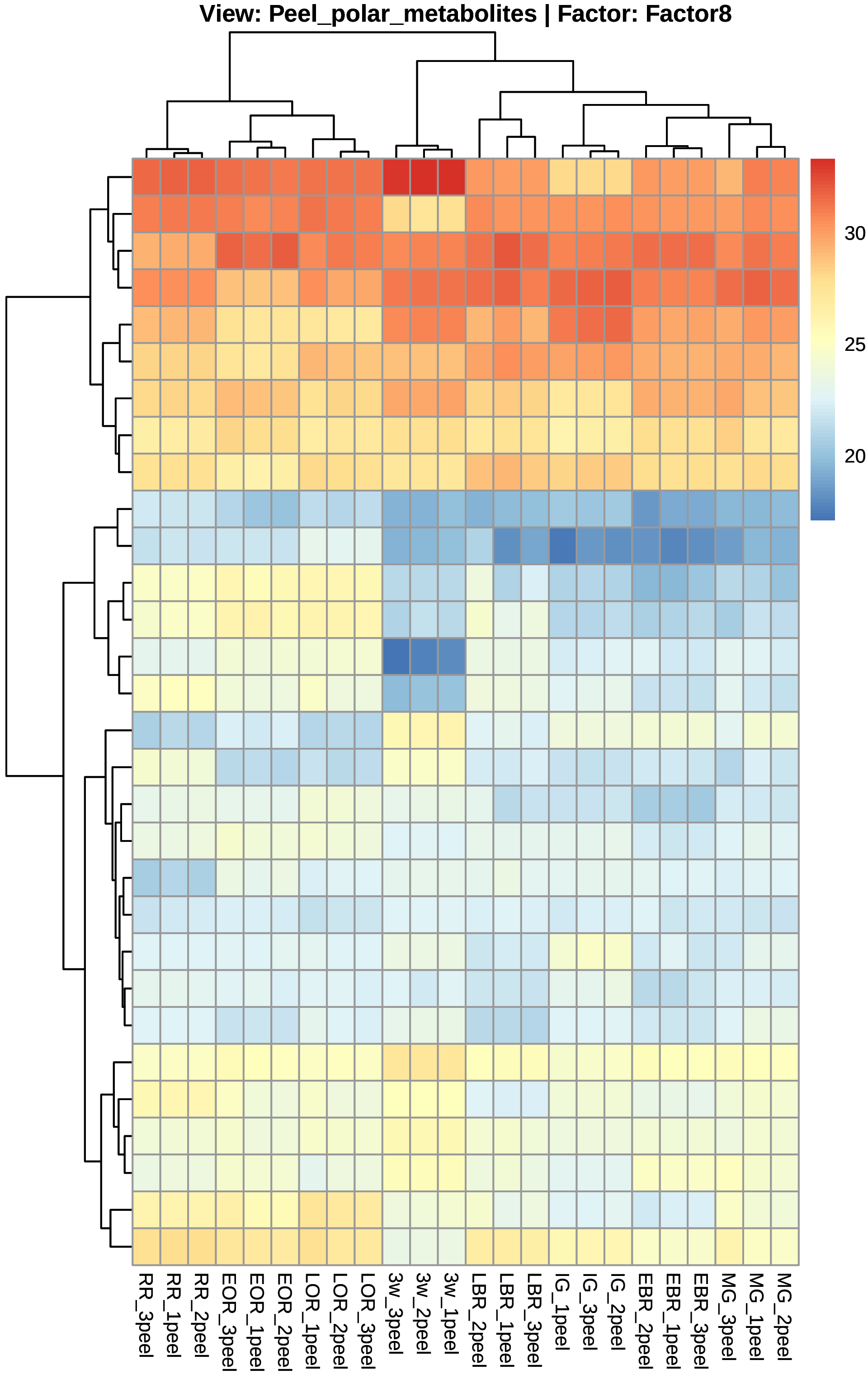
View: Peel_polar_metabolites | Factor: Factor8
30
25
20
RR_3peel
RR_1peel
RR_2peel
EOR_3peel
EOR_1peel
EOR_2peel
LOR_1peel
LOR_2peel
LOR_3peel
3w_3peel
3w_2peel
3w_1peel
LBR_2peel
LBR_1peel
LBR_3peel
IG_1peel
IG_3peel
IG_2peel
EBR_2peel
EBR_1peel
EBR_3peel
MG_3peel
MG_1peel
MG_2peel

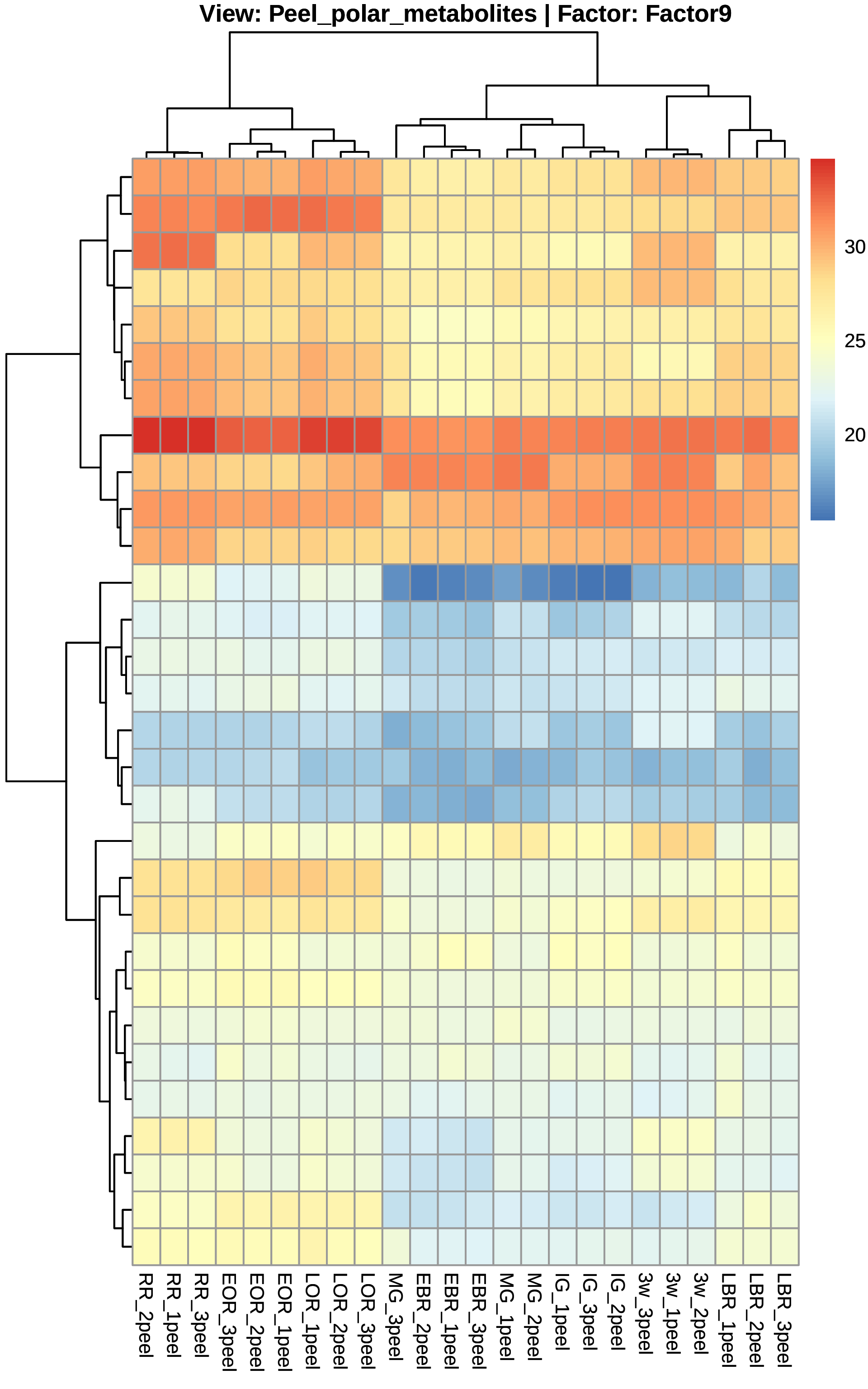

View: Peel_polar_metabolites | Factor: Factor9
30
25
20
RR_2peel
RR_1peel
RR_3peel
EOR_3peel
EOR_2peel
EOR_1peel
LOR_1peel
LOR_2peel
LOR_3peel
MG_3peel
EBR_2peel
EBR_1peel
EBR_3peel
MG_1peel
MG_2peel
IG_1peel
IG_3peel
IG_2peel
3w_3peel
3w_1peel
3w_2peel
LBR_1peel
LBR_2peel
LBR_3peel

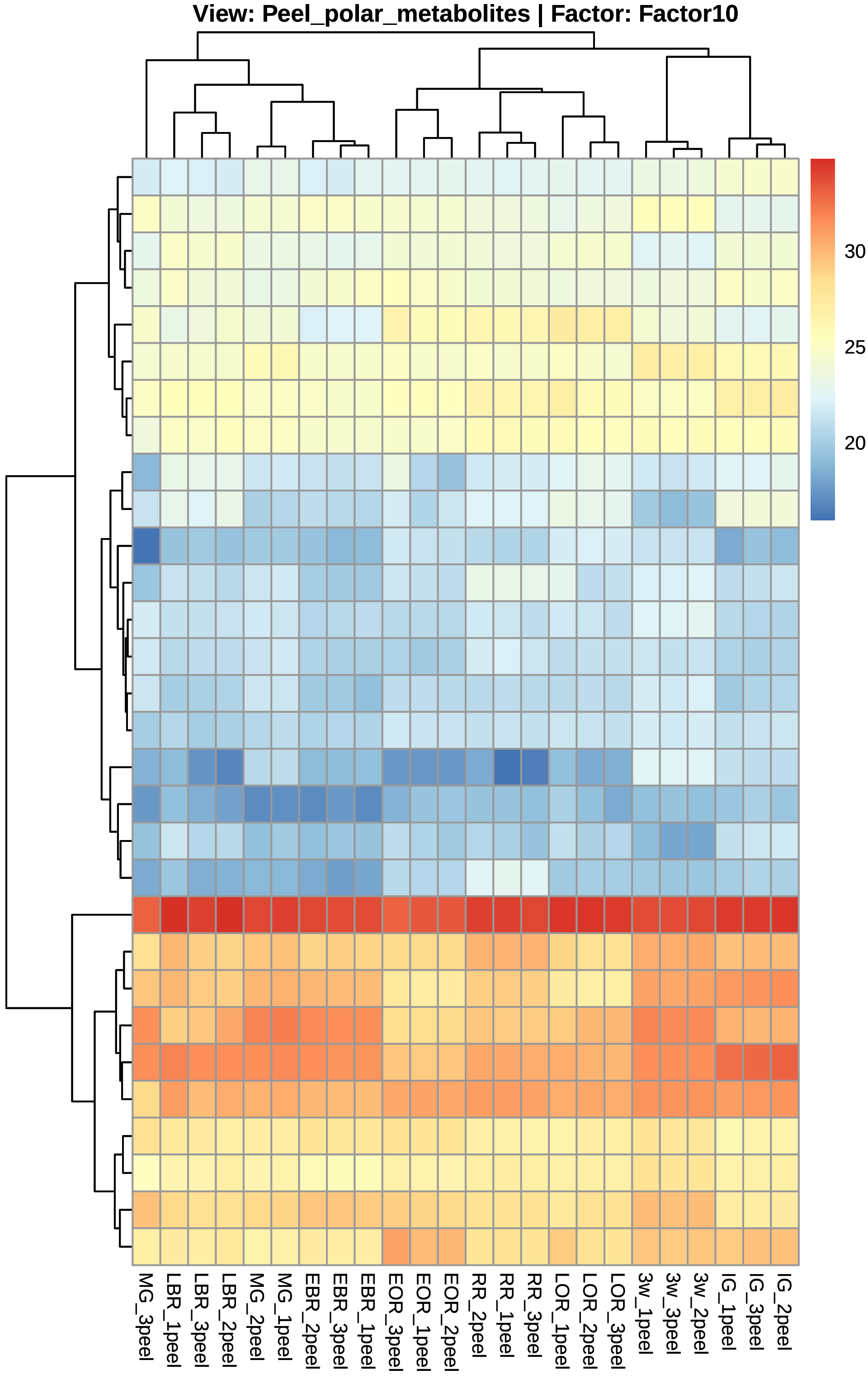
View: Peel_polar_metabolites | Factor: Factor10
30
25
20
MG_3peel
LBR_1peel
LBR_3peel
LBR_2peel
MG_2peel
MG_1peel
EBR_2peel
EBR_3peel
EBR_1peel
EOR_3peel
EOR_1peel
EOR_2peel
RR_2peel
RR_1peel
RR_3peel
LOR_1peel
LOR_2peel
LOR_3peel
3w_1peel
3w_3peel
3w_2peel
IG_1peel
IG_3peel
IG_2peel

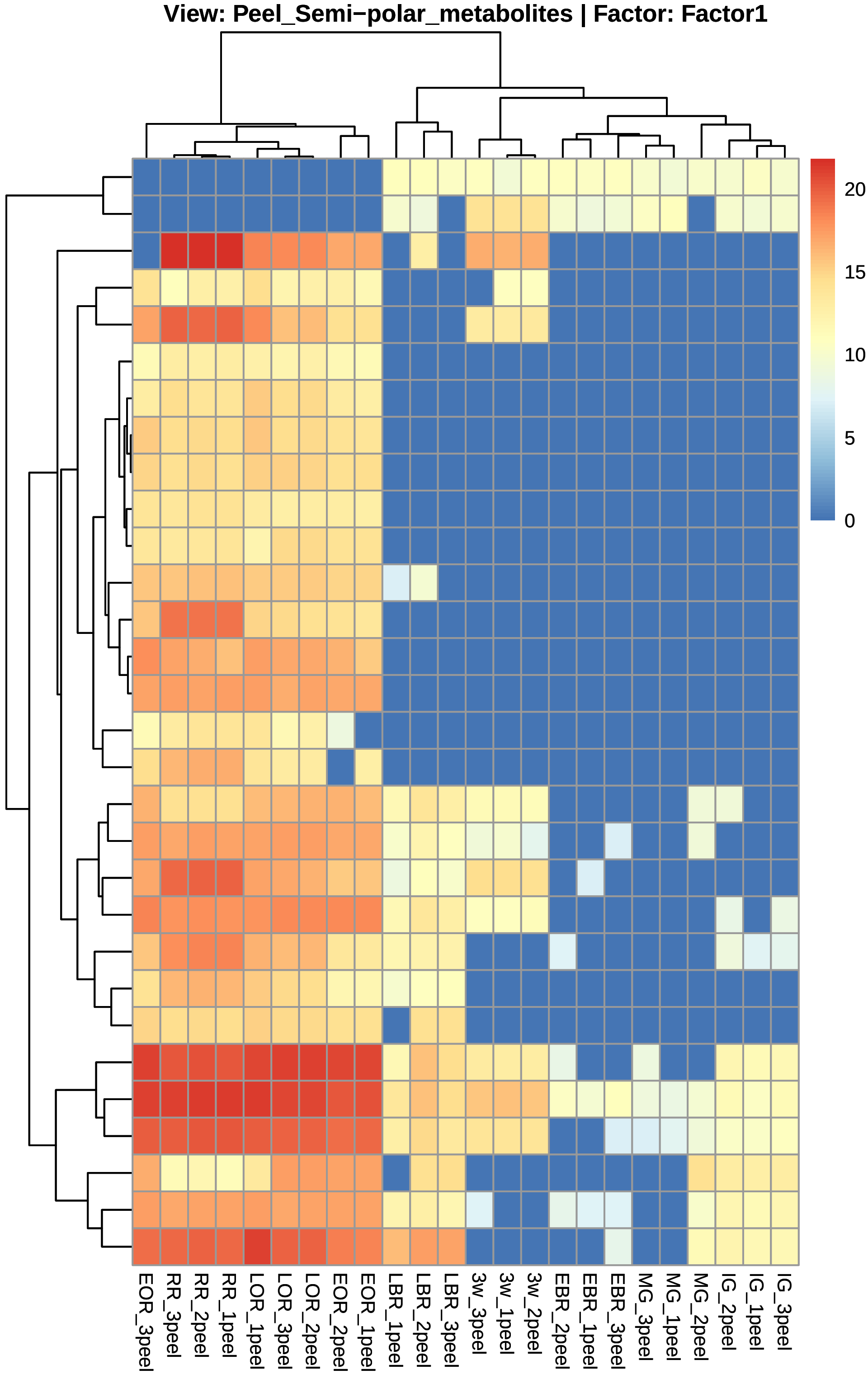

View: Peel_Semi−polar_metabolites | Factor: Factor1
EOR_3peel
RR_3peel
RR_2peel
RR_1peel
LOR_1peel
LOR_3peel
LOR_2peel
EOR_2peel
EOR_1peel
LBR_1peel
LBR_2peel
LBR_3peel
3w_3peel
3w_1peel
3w_2peel
EBR_2peel
EBR_1peel
EBR_3peel
MG_3peel
MG_1peel
MG_2peel
IG_2peel
IG_1peel
IG_3peel
20
15
10
5
0

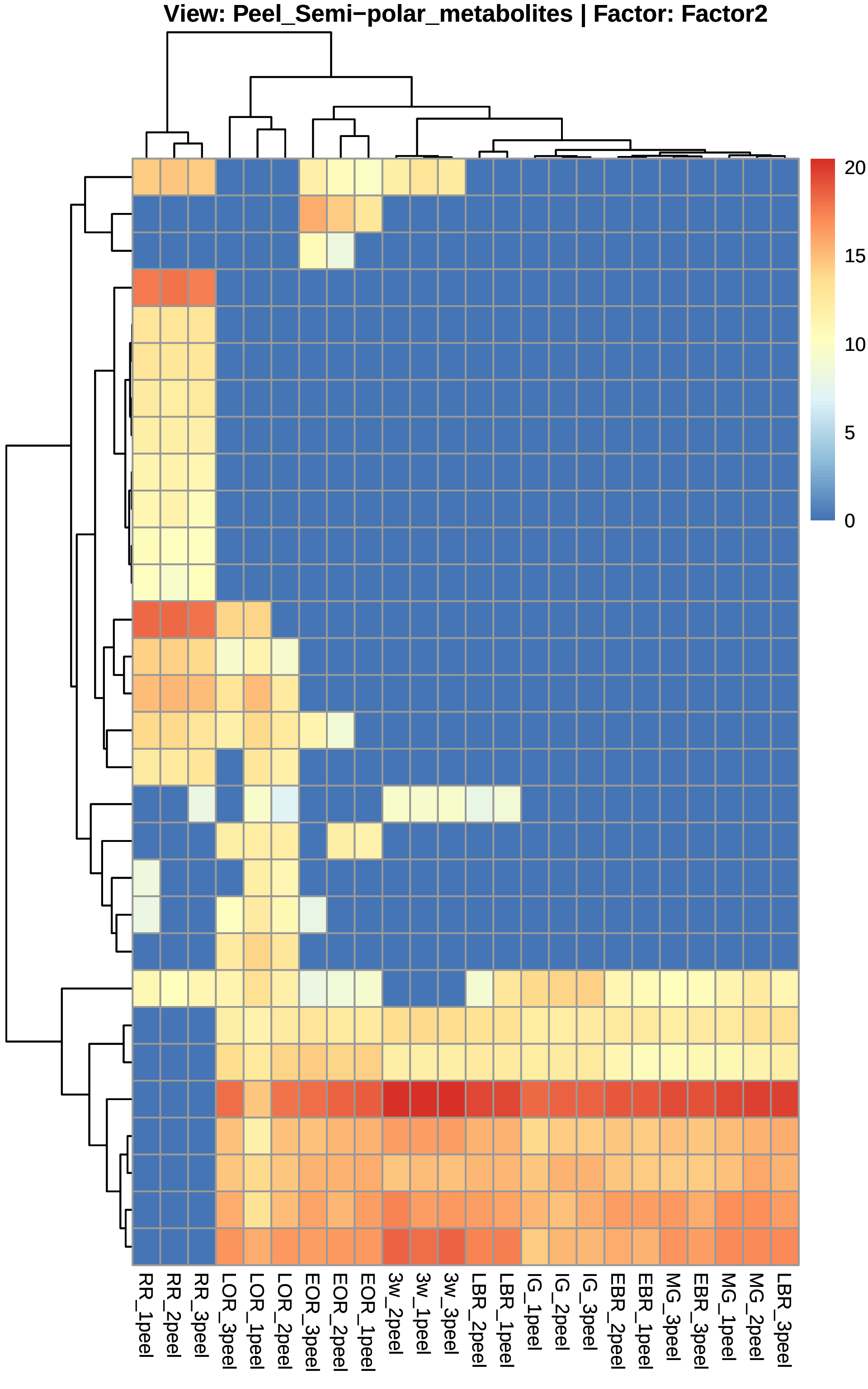

View: Peel_Semi−polar_metabolites | Factor: Factor2
20
15
10
5
0
RR_1peel
RR_2peel
RR_3peel
LOR_3peel
LOR_1peel
LOR_2peel
EOR_3peel
EOR_2peel
EOR_1peel
3w_2peel
3w_1peel
3w_3peel
LBR_2peel
LBR_1peel
IG_1peel
IG_2peel
IG_3peel
EBR_2peel
EBR_1peel
MG_3peel
EBR_3peel
MG_1peel
MG_2peel
LBR_3peel

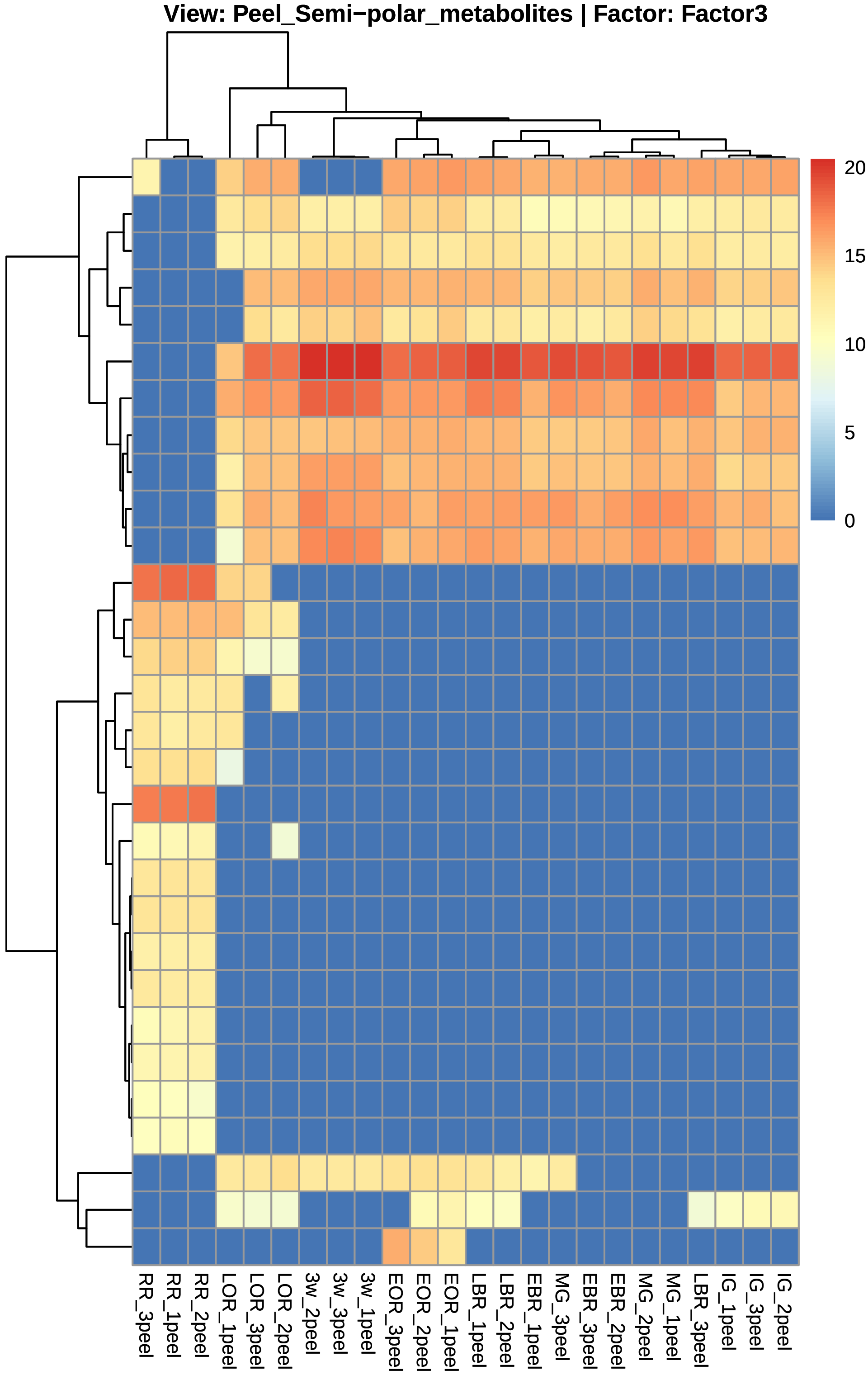
View: Peel_Semi−polar_metabolites | Factor: Factor3
20
15
10
5
0
RR_3peel
RR_1peel
RR_2peel
LOR_1peel
LOR_3peel
LOR_2peel
3w_2peel
3w_3peel
3w_1peel
EOR_3peel
EOR_2peel
EOR_1peel
LBR_1peel
LBR_2peel
EBR_1peel
MG_3peel
EBR_3peel
EBR_2peel
MG_2peel
MG_1peel
LBR_3peel
IG_1peel
IG_3peel
IG_2peel

View: Peel_Semi−polar_metabolites | Factor: Factor4

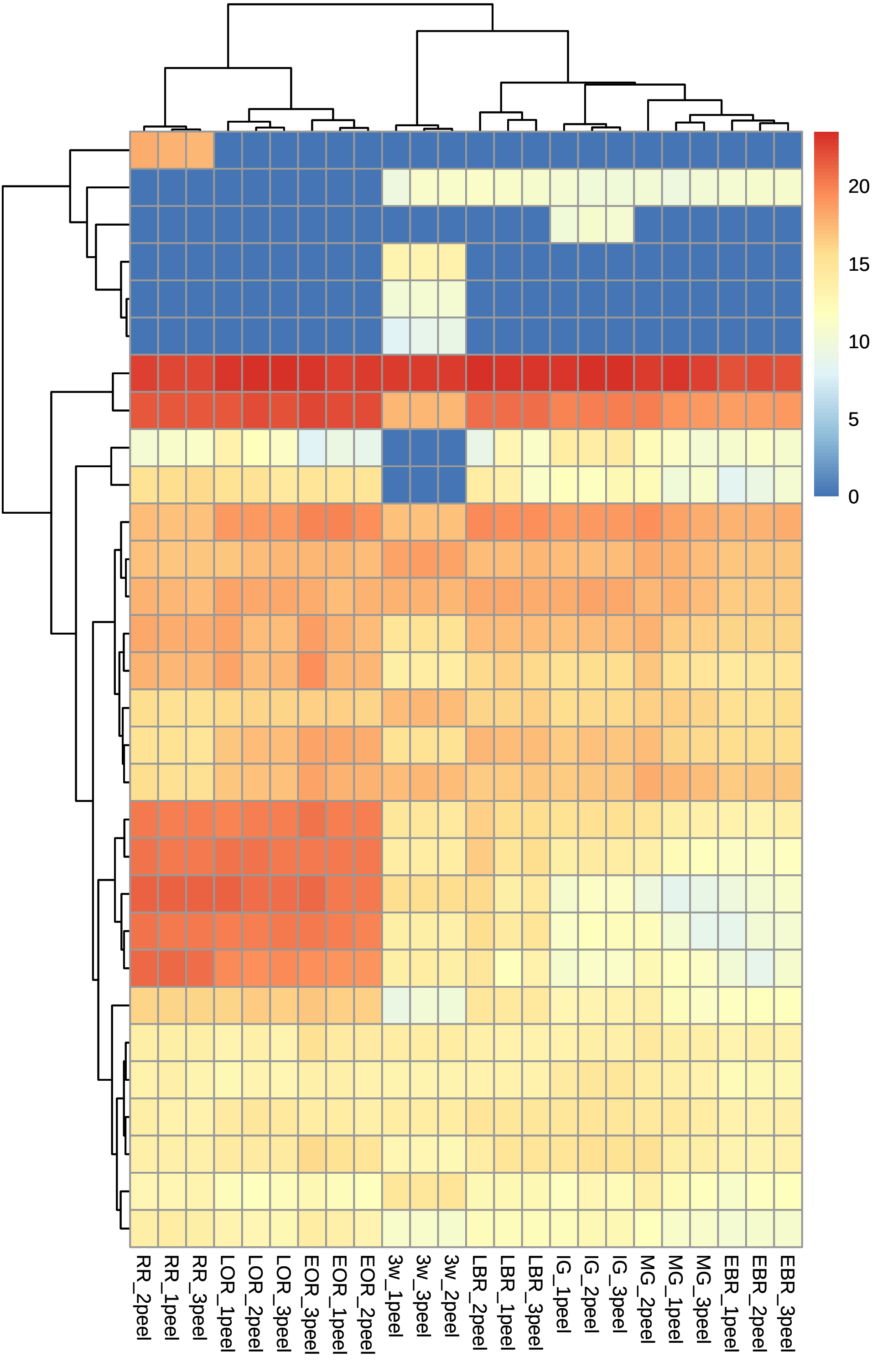

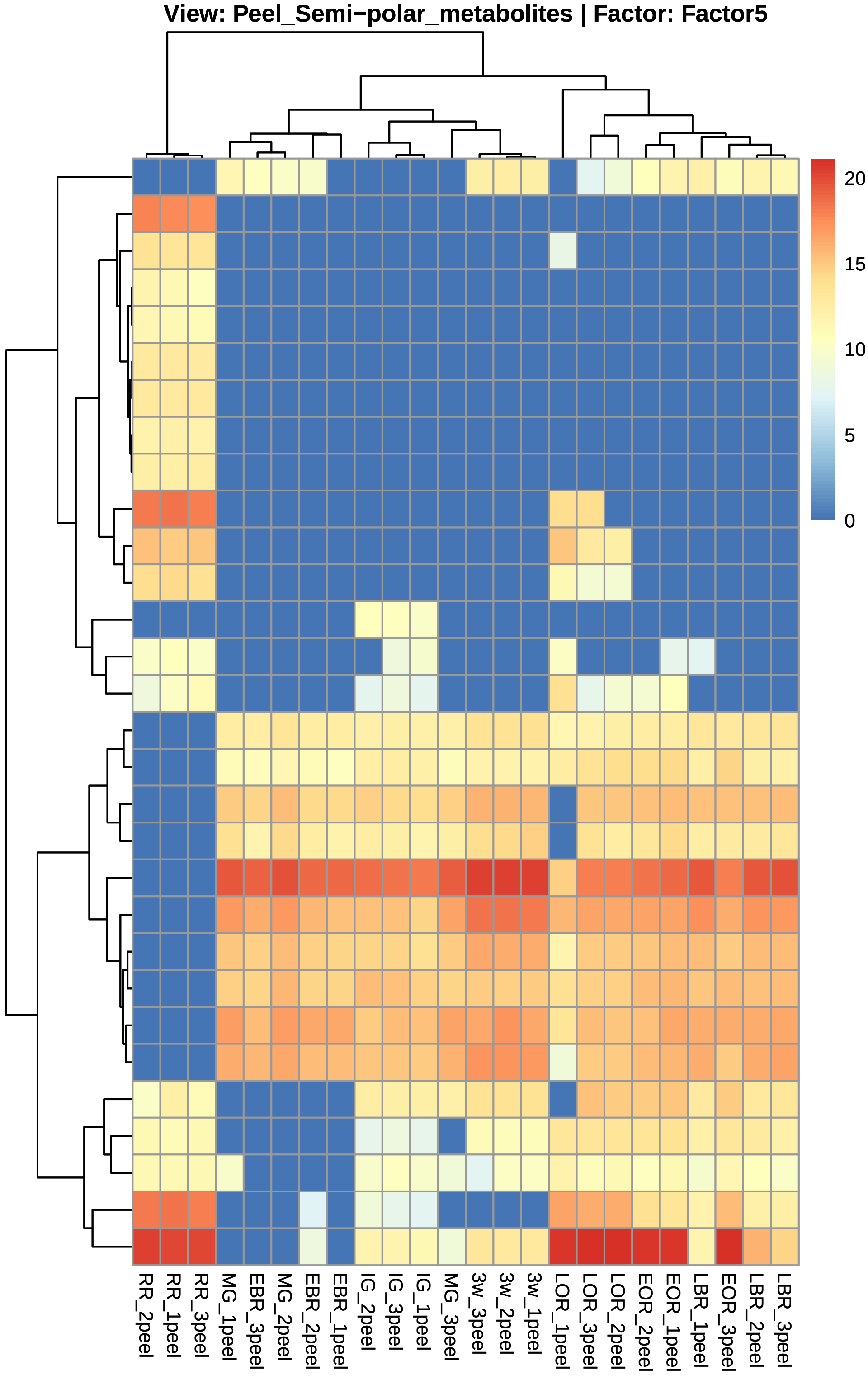
View: Peel_Semi−polar_metabolites | Factor: Factor5
20
15
10
5
0
RR_2peel
RR_1peel
RR_3peel
MG_1peel
EBR_3peel
MG_2peel
EBR_2peel
EBR_1peel
IG_2peel
IG_3peel
IG_1peel
MG_3peel
3w_3peel
3w_2peel
3w_1peel
LOR_1peel
LOR_3peel
LOR_2peel
EOR_2peel
EOR_1peel
LBR_1peel
EOR_3peel
LBR_2peel
LBR_3peel

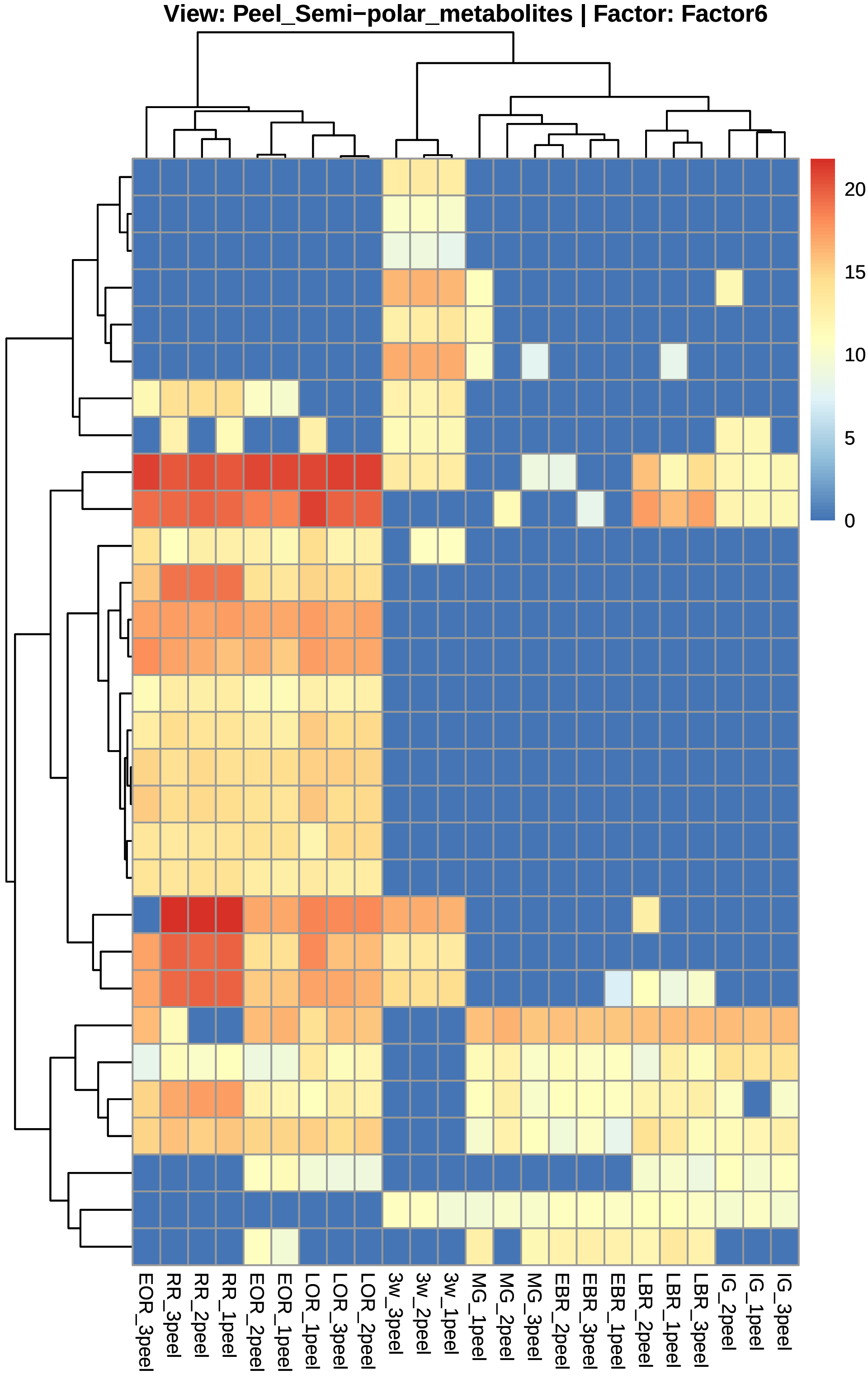

View: Peel_Semi−polar_metabolites | Factor: Factor6
20
15
10
5
0
EOR_3peel
RR_3peel
RR_2peel
RR_1peel
EOR_2peel
EOR_1peel
LOR_1peel
LOR_3peel
LOR_2peel
3w_3peel
3w_2peel
3w_1peel
MG_1peel
MG_2peel
MG_3peel
EBR_2peel
EBR_3peel
EBR_1peel
LBR_2peel
LBR_1peel
LBR_3peel
IG_2peel
IG_1peel
IG_3peel

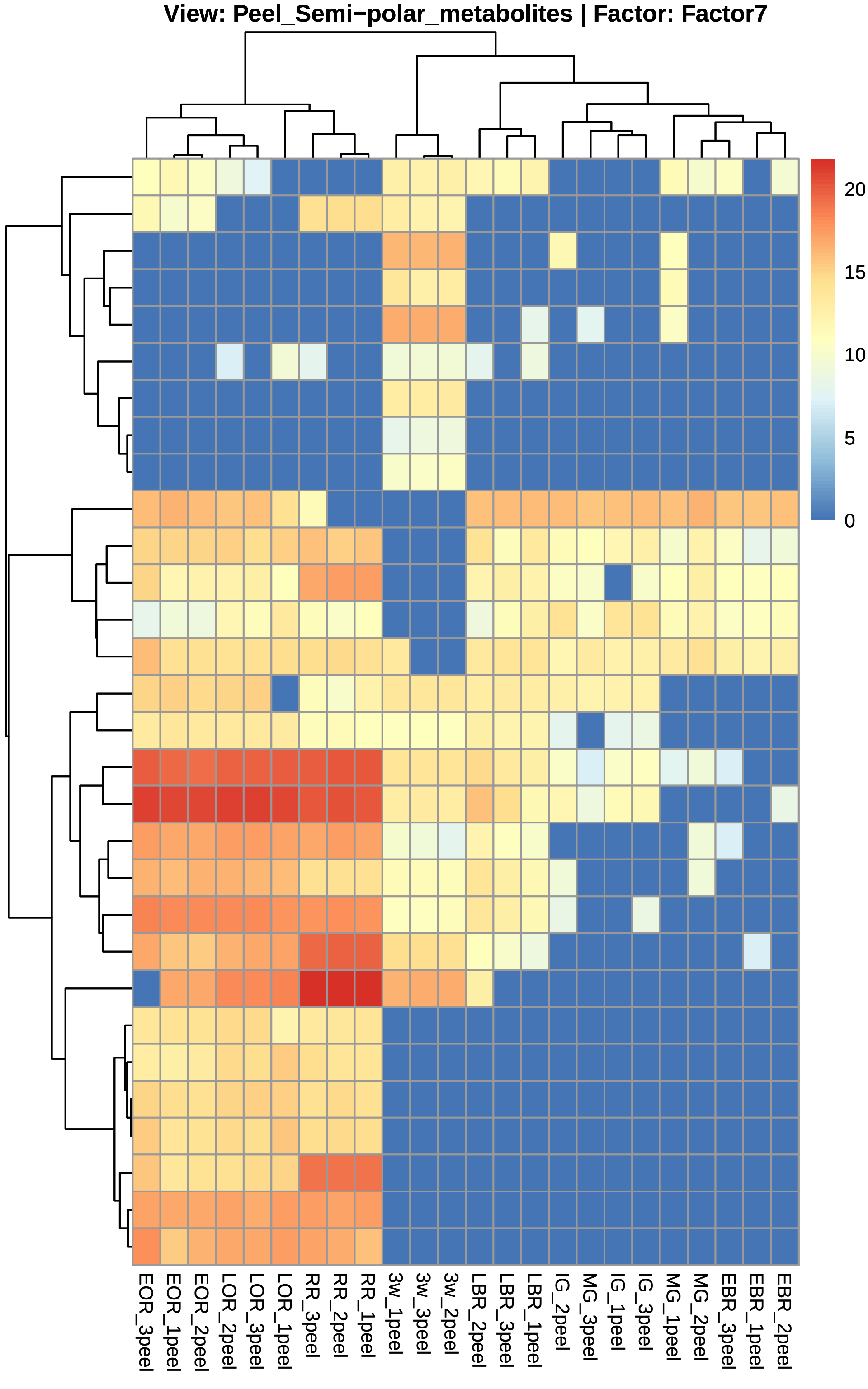
View: Peel_Semi−polar_metabolites | Factor: Factor7
20
15
10
5
0
EOR_3peel
EOR_1peel
EOR_2peel
LOR_2peel
LOR_3peel
LOR_1peel
RR_3peel
RR_2peel
RR_1peel
3w_1peel
3w_3peel
3w_2peel
LBR_2peel
LBR_3peel
LBR_1peel
IG_2peel
MG_3peel
IG_1peel
IG_3peel
MG_1peel
MG_2peel
EBR_3peel
EBR_1peel
EBR_2peel

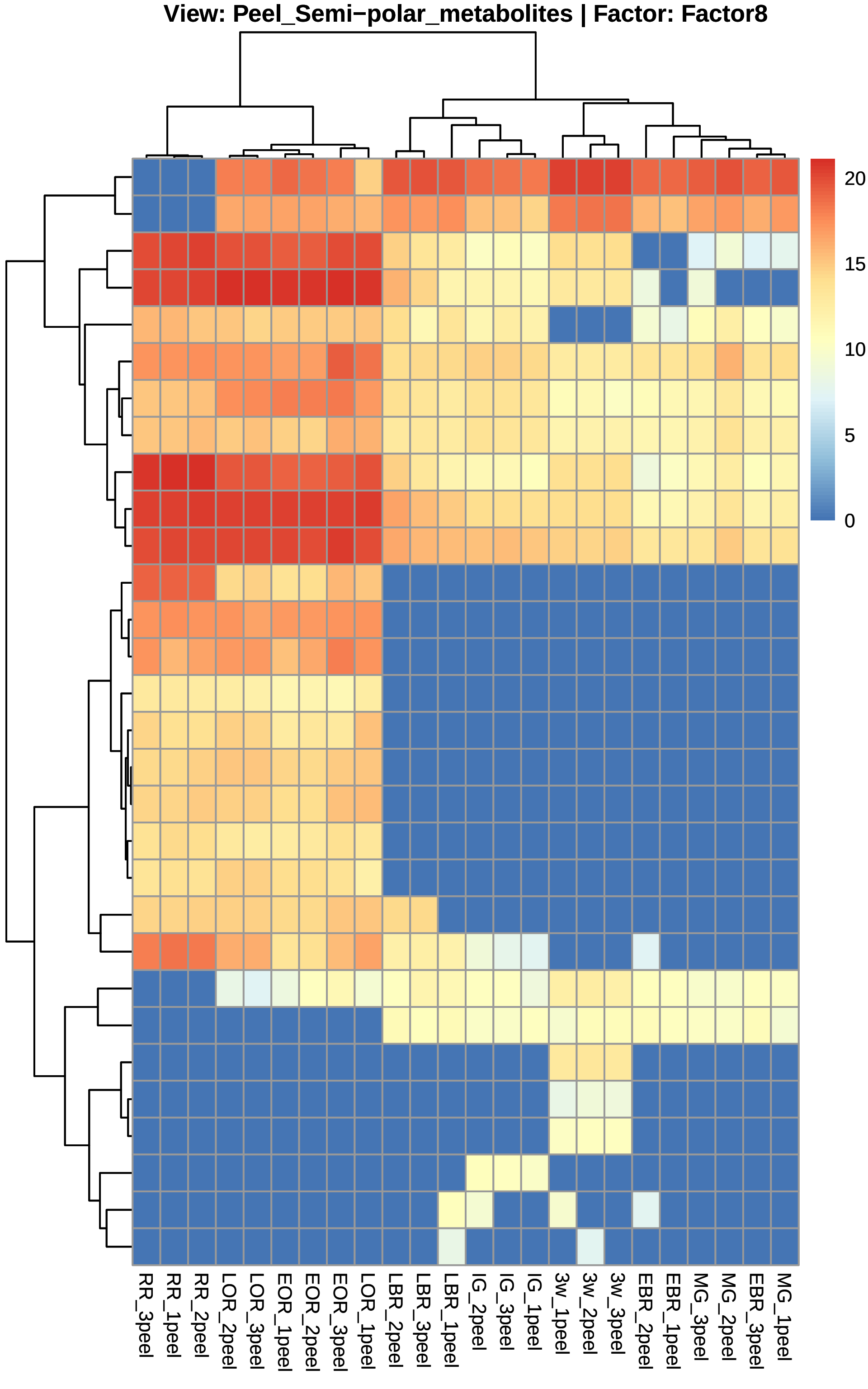
View: Peel_Semi−polar_metabolites | Factor: Factor8
20
15
10
5
0
RR_3peel
RR_1peel
RR_2peel
LOR_2peel
LOR_3peel
EOR_1peel
EOR_2peel
EOR_3peel
LOR_1peel
LBR_2peel
LBR_3peel
LBR_1peel
IG_2peel
IG_3peel
IG_1peel
3w_1peel
3w_2peel
3w_3peel
EBR_2peel
EBR_1peel
MG_3peel
MG_2peel
EBR_3peel
MG_1peel

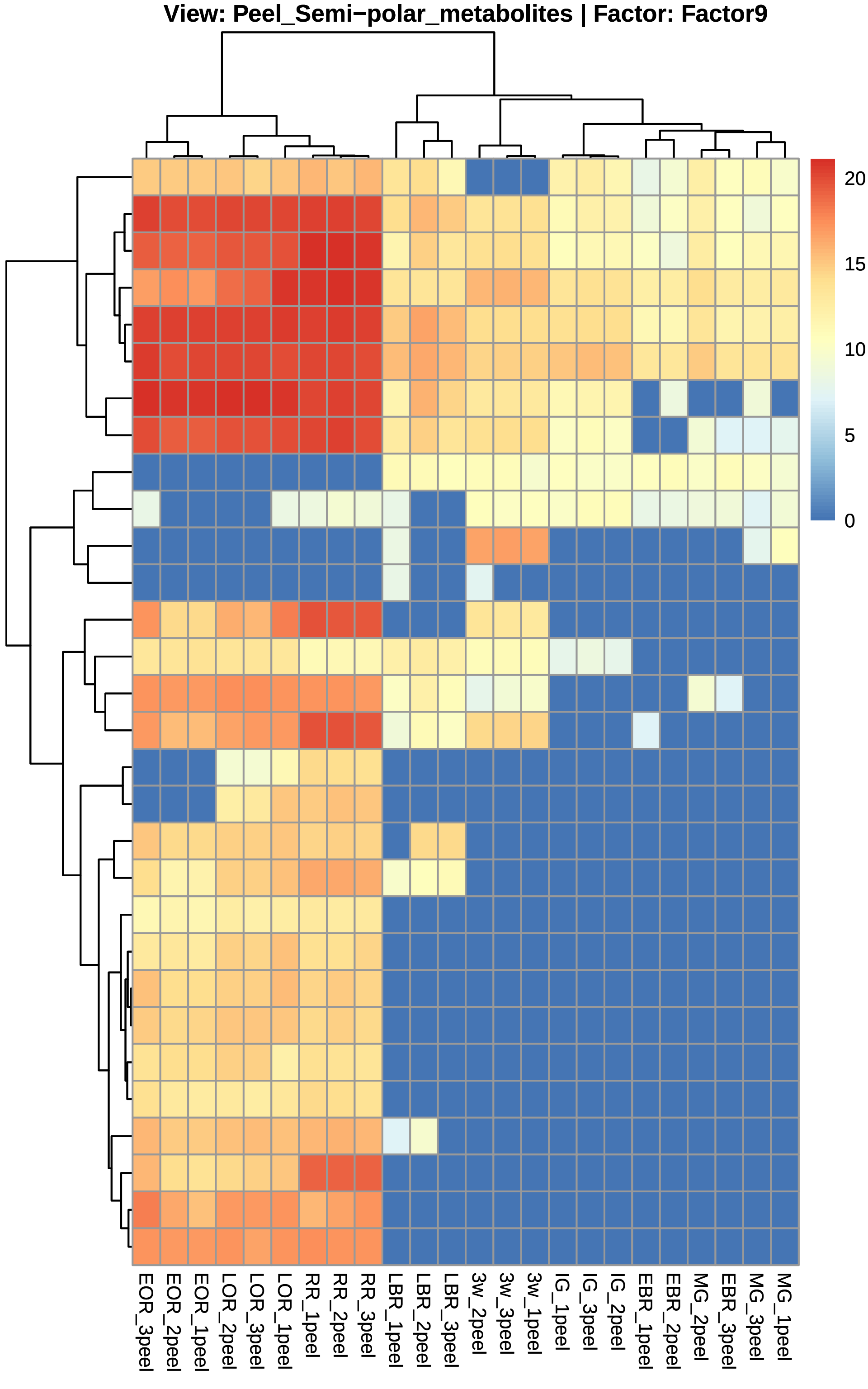

View: Peel_Semi−polar_metabolites | Factor: Factor9
20
15
10
5
0
EOR_3peel
EOR_2peel
EOR_1peel
LOR_2peel
LOR_3peel
LOR_1peel
RR_1peel
RR_2peel
RR_3peel
LBR_1peel
LBR_2peel
LBR_3peel
3w_2peel
3w_3peel
3w_1peel
IG_1peel
IG_3peel
IG_2peel
EBR_1peel
EBR_2peel
MG_2peel
EBR_3peel
MG_3peel
MG_1peel

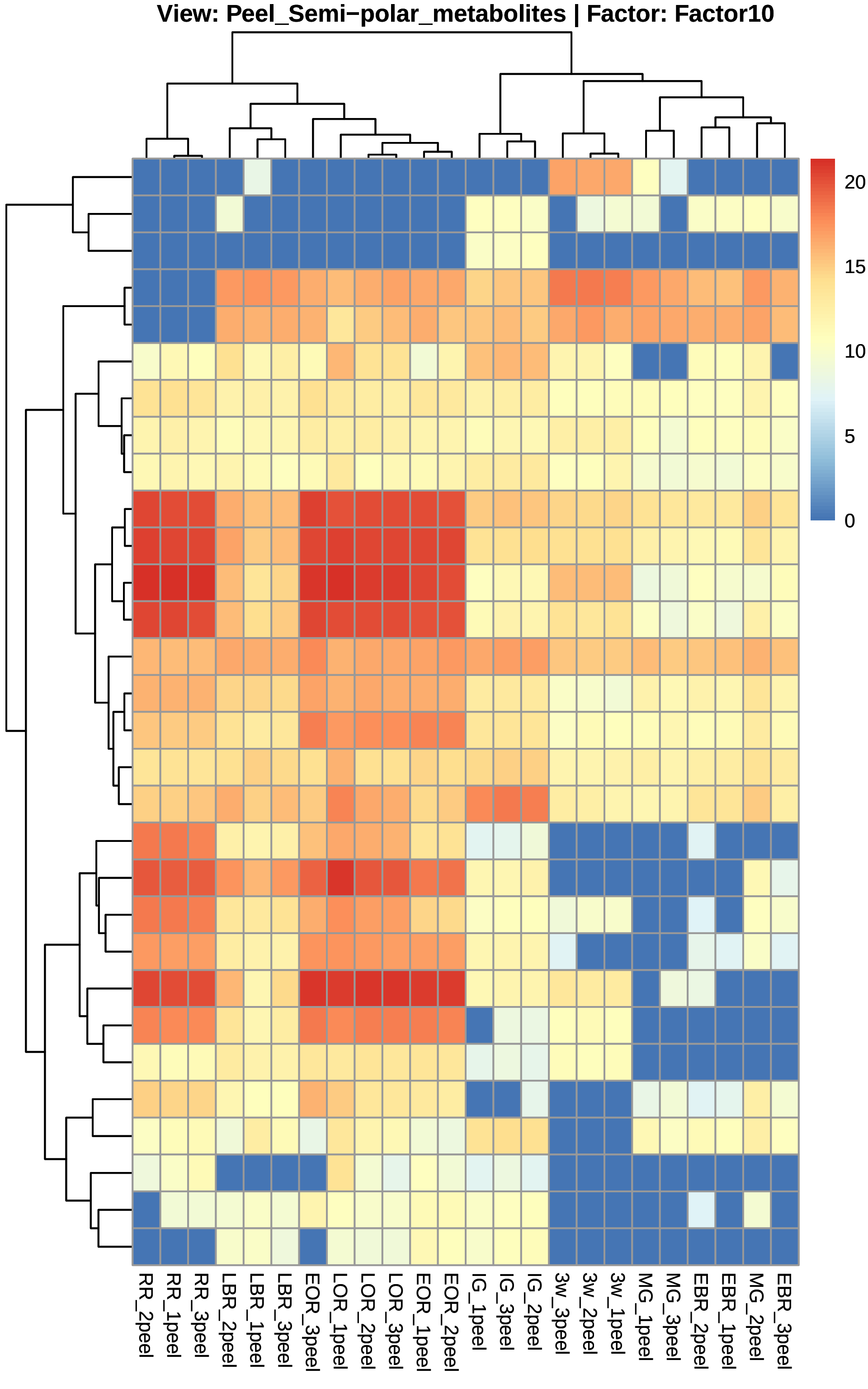

View: Peel_Semi−polar_metabolites | Factor: Factor10
20
15
10
5
0
RR_2peel
RR_1peel
RR_3peel
LBR_2peel
LBR_1peel
LBR_3peel
EOR_3peel
LOR_1peel
LOR_2peel
LOR_3peel
EOR_1peel
EOR_2peel
IG_1peel
IG_3peel
IG_2peel
3w_3peel
3w_2peel
3w_1peel
MG_1peel
MG_3peel
EBR_2peel
EBR_1peel
MG_2peel
EBR_3peel

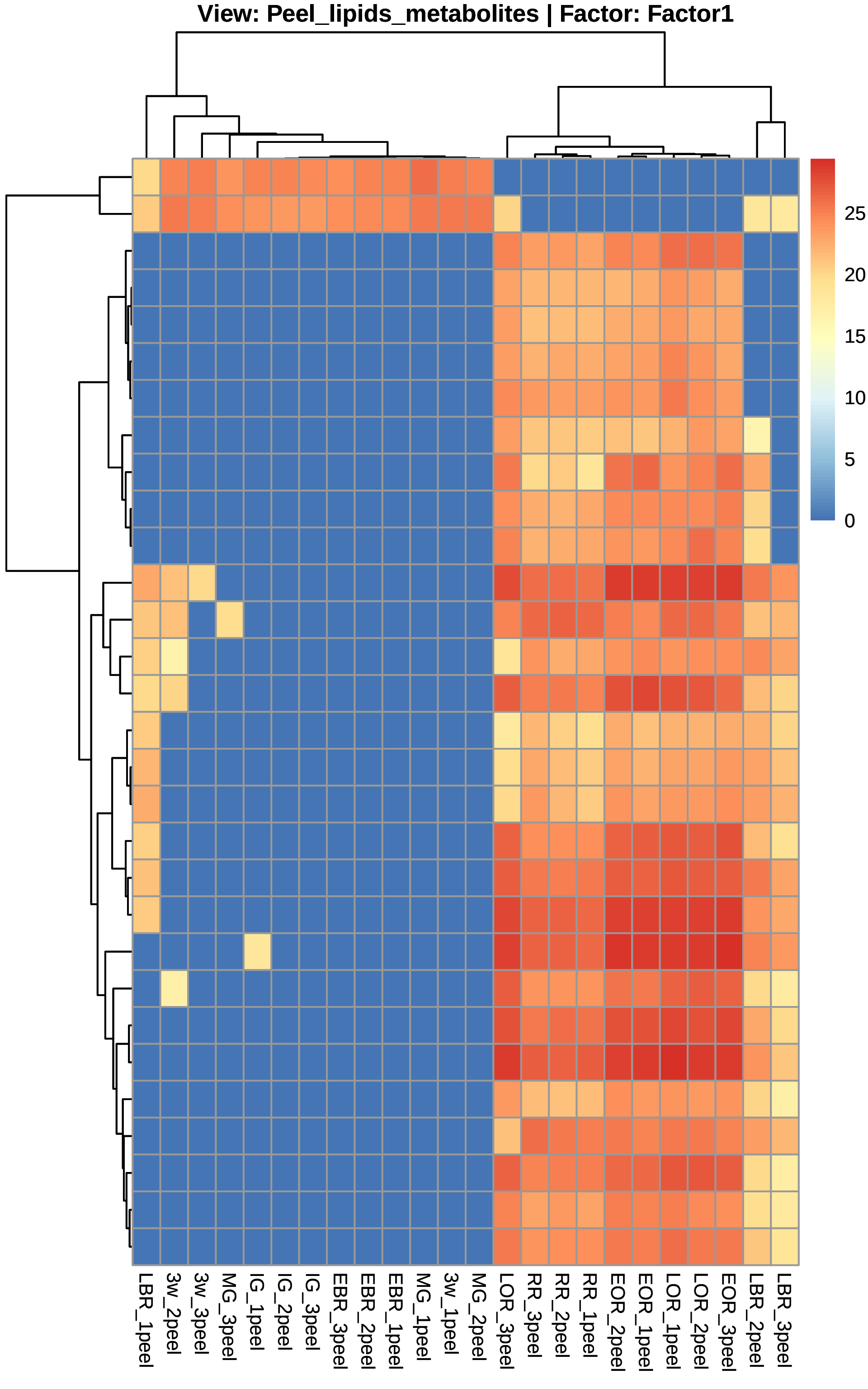
View: Peel_lipids_metabolites | Factor: Factor1
25
20
15
10
5
0
LBR_1peel
3w_2peel
3w_3peel
MG_3peel
IG_1peel
IG_2peel
IG_3peel
EBR_3peel
EBR_2peel
EBR_1peel
MG_1peel
3w_1peel
MG_2peel
LOR_3peel
RR_3peel
RR_2peel
RR_1peel
EOR_2peel
EOR_1peel
LOR_1peel
LOR_2peel
EOR_3peel
LBR_2peel
LBR_3peel

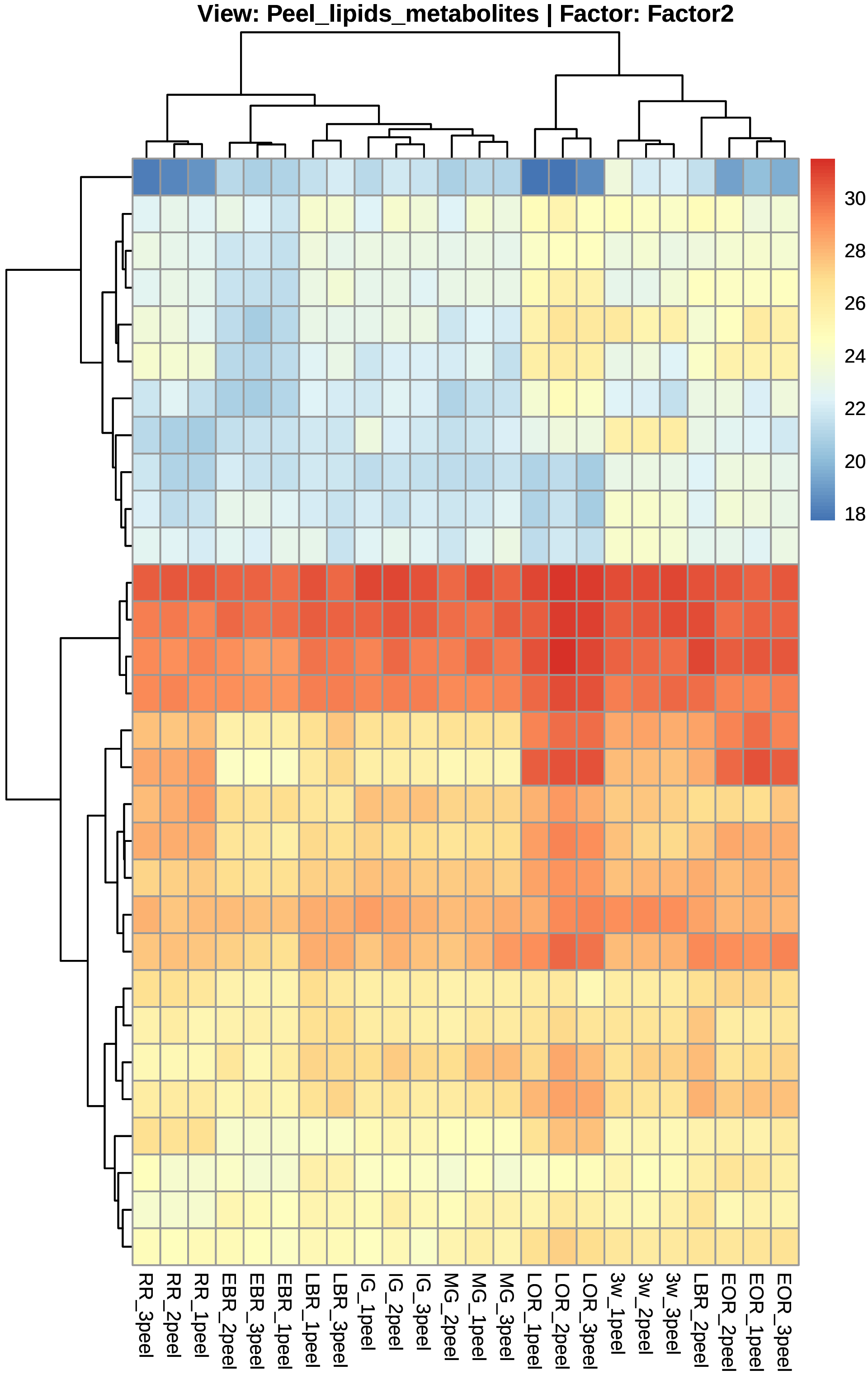
View: Peel_lipids_metabolites | Factor: Factor2
30
28
26
24
22
20
18
RR_3peel
RR_2peel
RR_1peel
EBR_2peel
EBR_3peel
EBR_1peel
LBR_1peel
LBR_3peel
IG_1peel
IG_2peel
IG_3peel
MG_2peel
MG_1peel
MG_3peel
LOR_1peel
LOR_2peel
LOR_3peel
3w_1peel
3w_2peel
3w_3peel
LBR_2peel
EOR_2peel
EOR_1peel
EOR_3peel

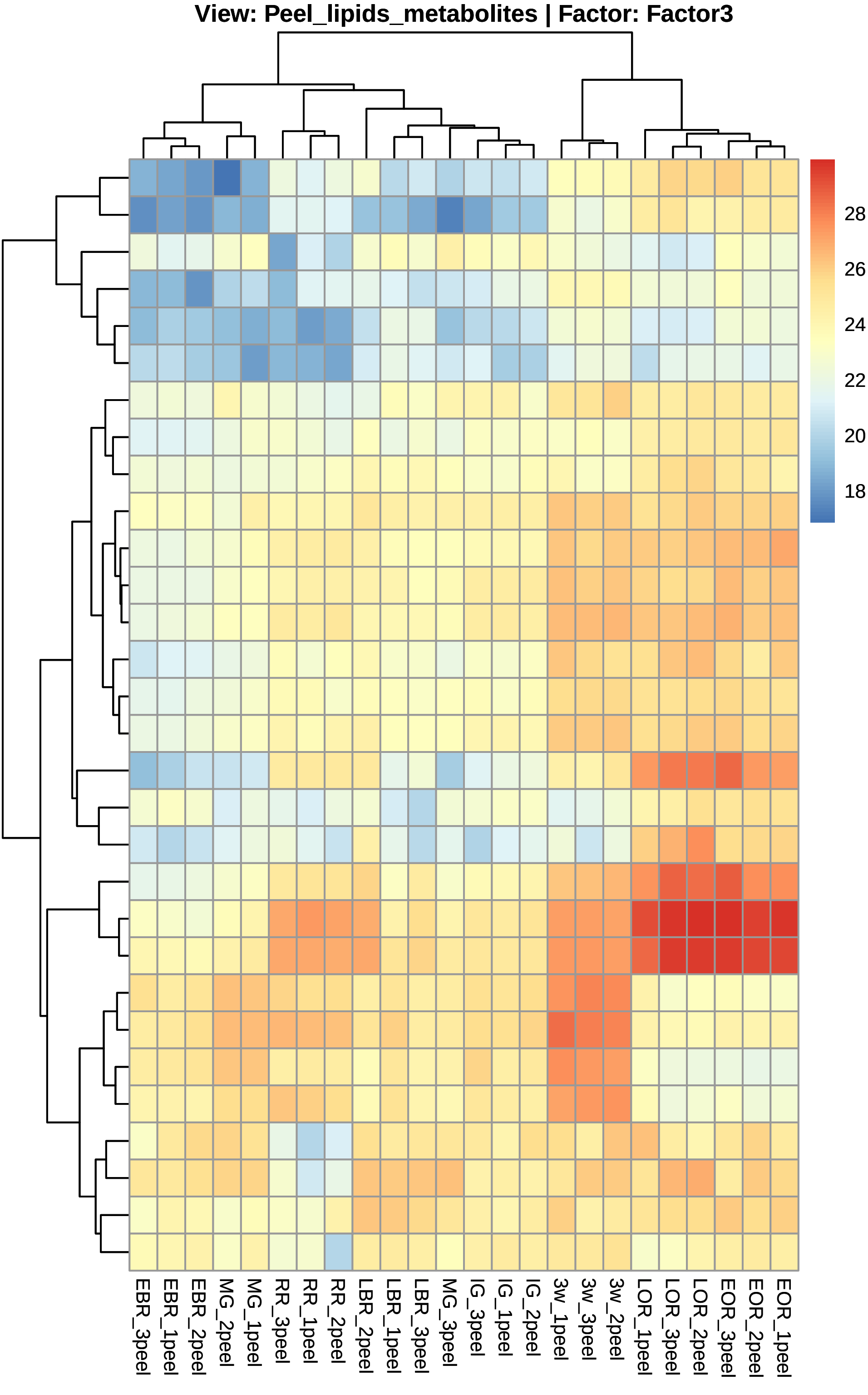
View: Peel_lipids_metabolites | Factor: Factor3
28
26
24
22
20
18
EBR_3peel
EBR_1peel
EBR_2peel
MG_2peel
MG_1peel
RR_3peel
RR_1peel
RR_2peel
LBR_2peel
LBR_1peel
LBR_3peel
MG_3peel
IG_3peel
IG_1peel
IG_2peel
3w_1peel
3w_3peel
3w_2peel
LOR_1peel
LOR_3peel
LOR_2peel
EOR_3peel
EOR_2peel
EOR_1peel

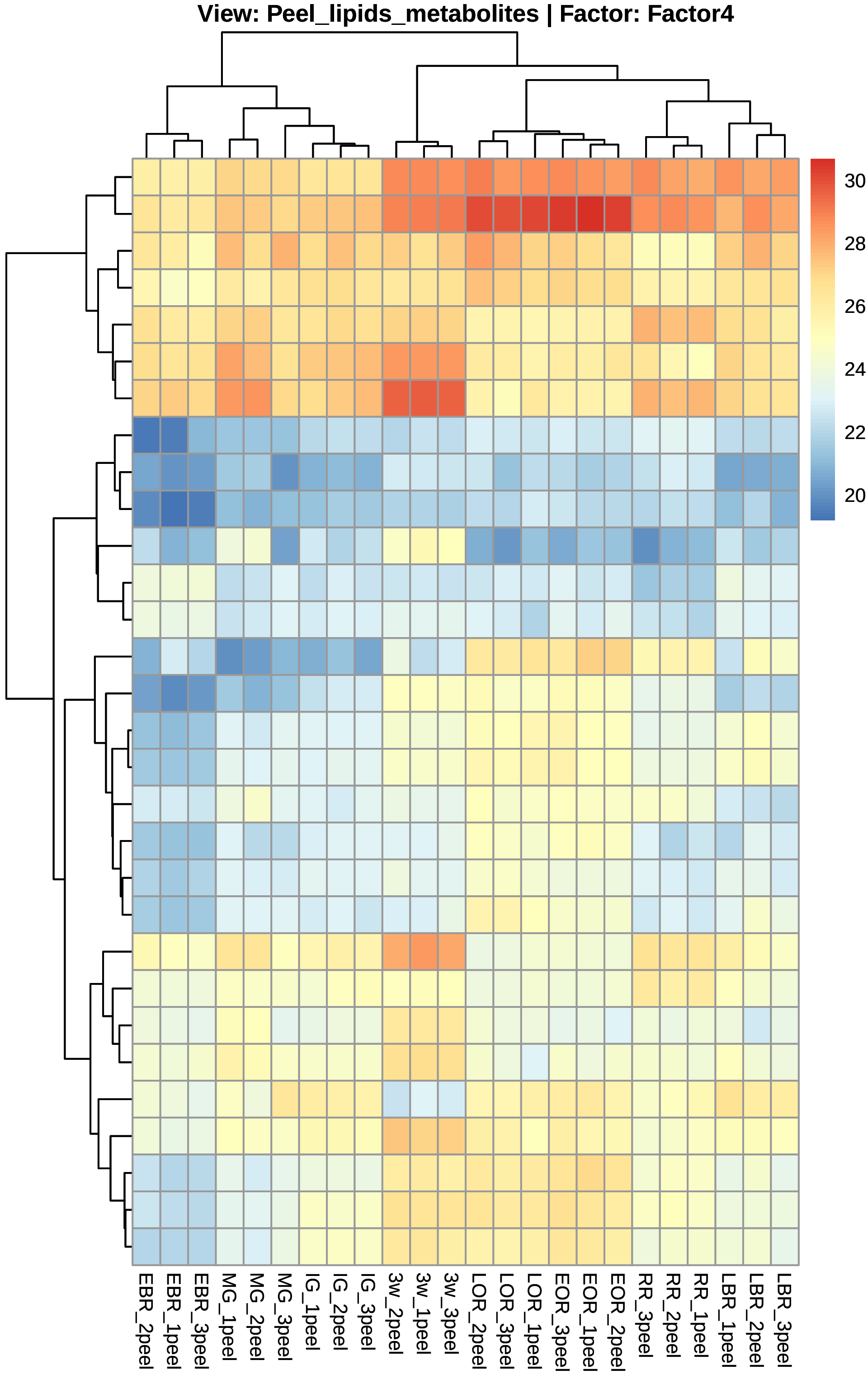
View: Peel_lipids_metabolites | Factor: Factor4
30
28
26
24
22
20
EBR_2peel
EBR_1peel
EBR_3peel
MG_1peel
MG_2peel
MG_3peel
IG_1peel
IG_2peel
IG_3peel
3w_2peel
3w_1peel
3w_3peel
LOR_2peel
LOR_3peel
LOR_1peel
EOR_3peel
EOR_1peel
EOR_2peel
RR_3peel
RR_2peel
RR_1peel
LBR_1peel
LBR_2peel
LBR_3peel

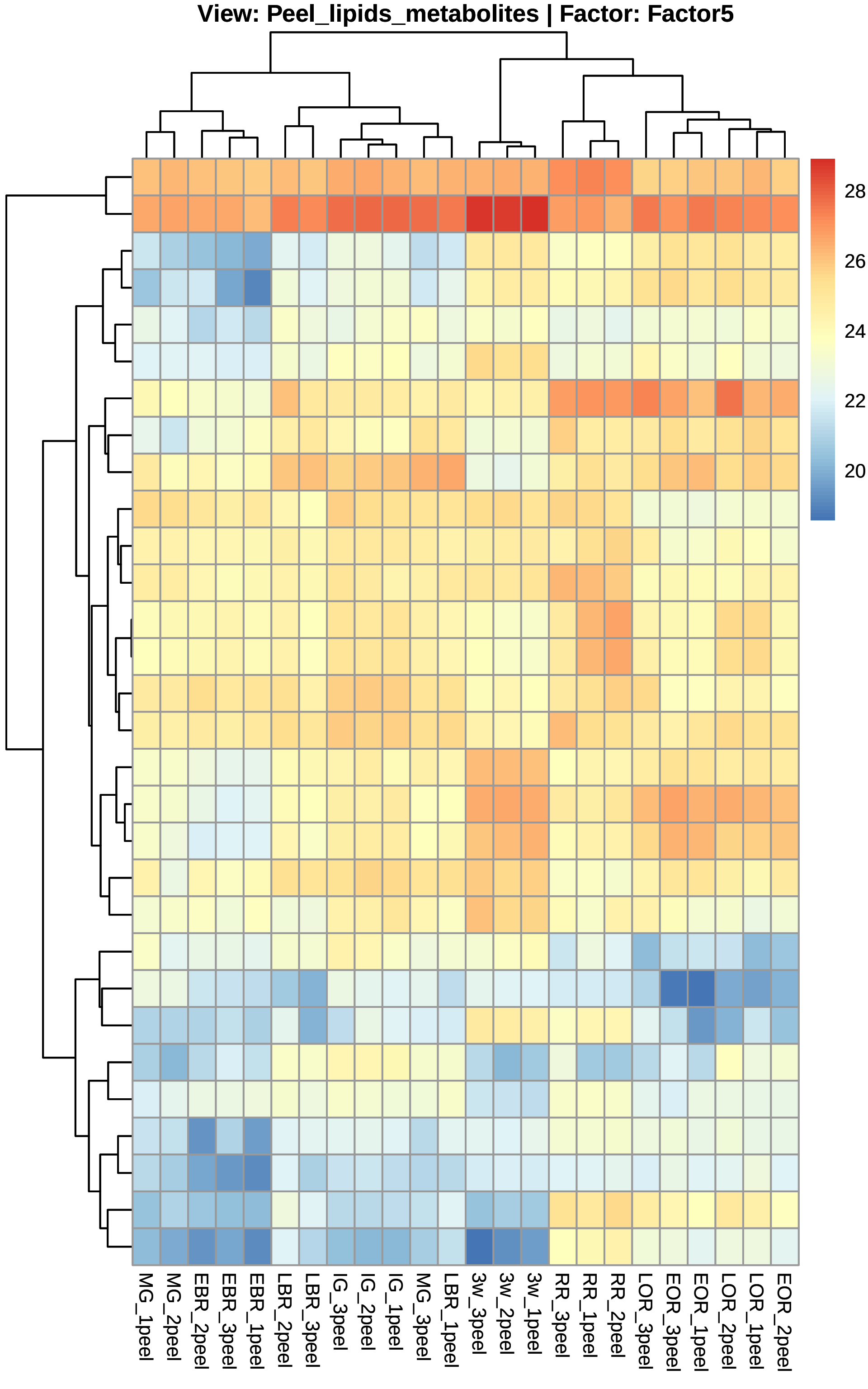
View: Peel_lipids_metabolites | Factor: Factor5
28
26
24
22
20
MG_1peel
MG_2peel
EBR_2peel
EBR_3peel
EBR_1peel
LBR_2peel
LBR_3peel
IG_3peel
IG_2peel
IG_1peel
MG_3peel
LBR_1peel
3w_3peel
3w_2peel
3w_1peel
RR_3peel
RR_1peel
RR_2peel
LOR_3peel
EOR_3peel
EOR_1peel
LOR_2peel
LOR_1peel
EOR_2peel

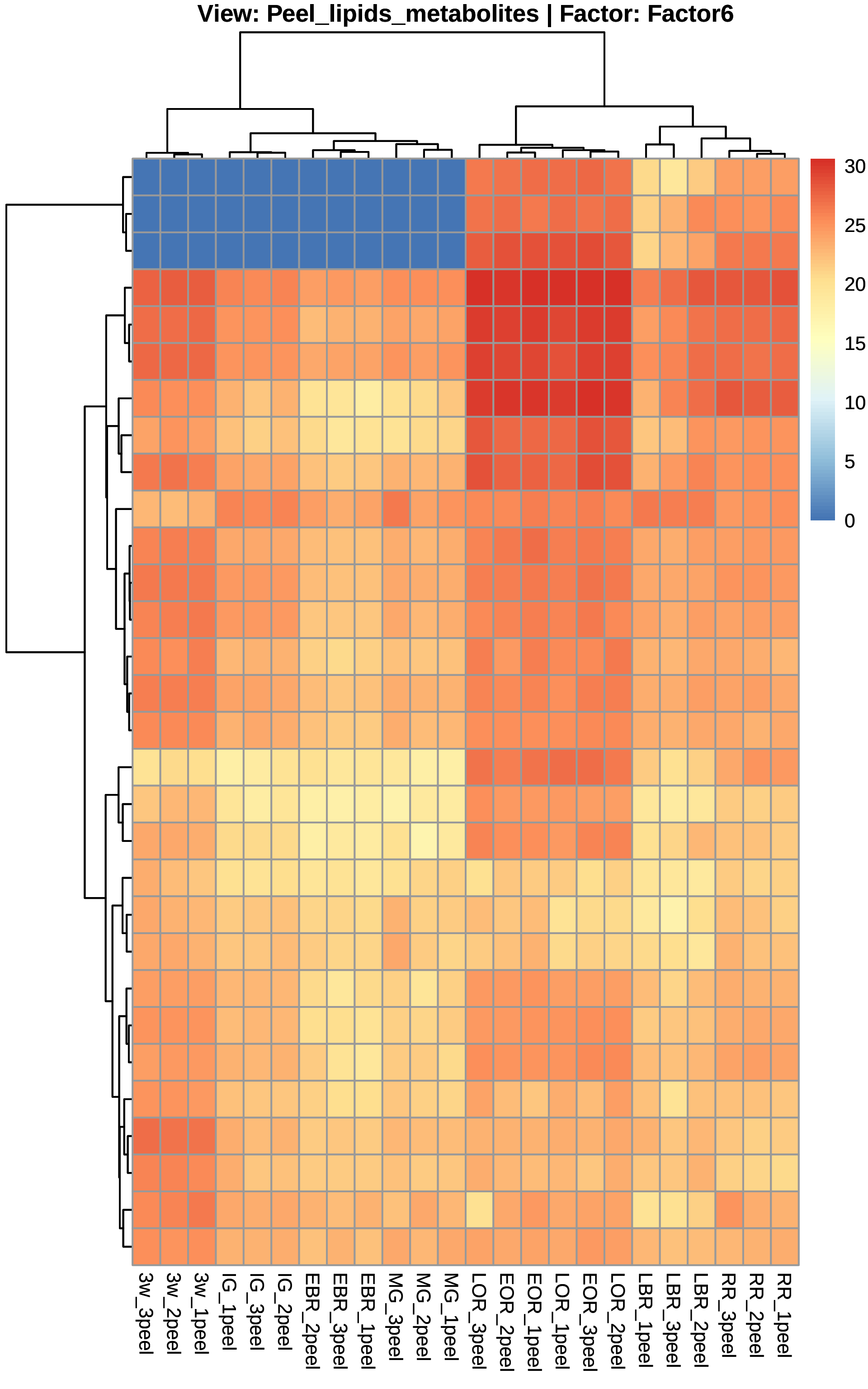
View: Peel_lipids_metabolites | Factor: Factor6
3w_3peel
3w_2peel
3w_1peel
IG_1peel
IG_3peel
IG_2peel
EBR_2peel
EBR_3peel
EBR_1peel
MG_3peel
MG_2peel
MG_1peel
LOR_3peel
EOR_2peel
EOR_1peel
LOR_1peel
EOR_3peel
LOR_2peel
LBR_1peel
LBR_3peel
LBR_2peel
RR_3peel
RR_2peel
RR_1peel
30
25
20
15
10
5
0

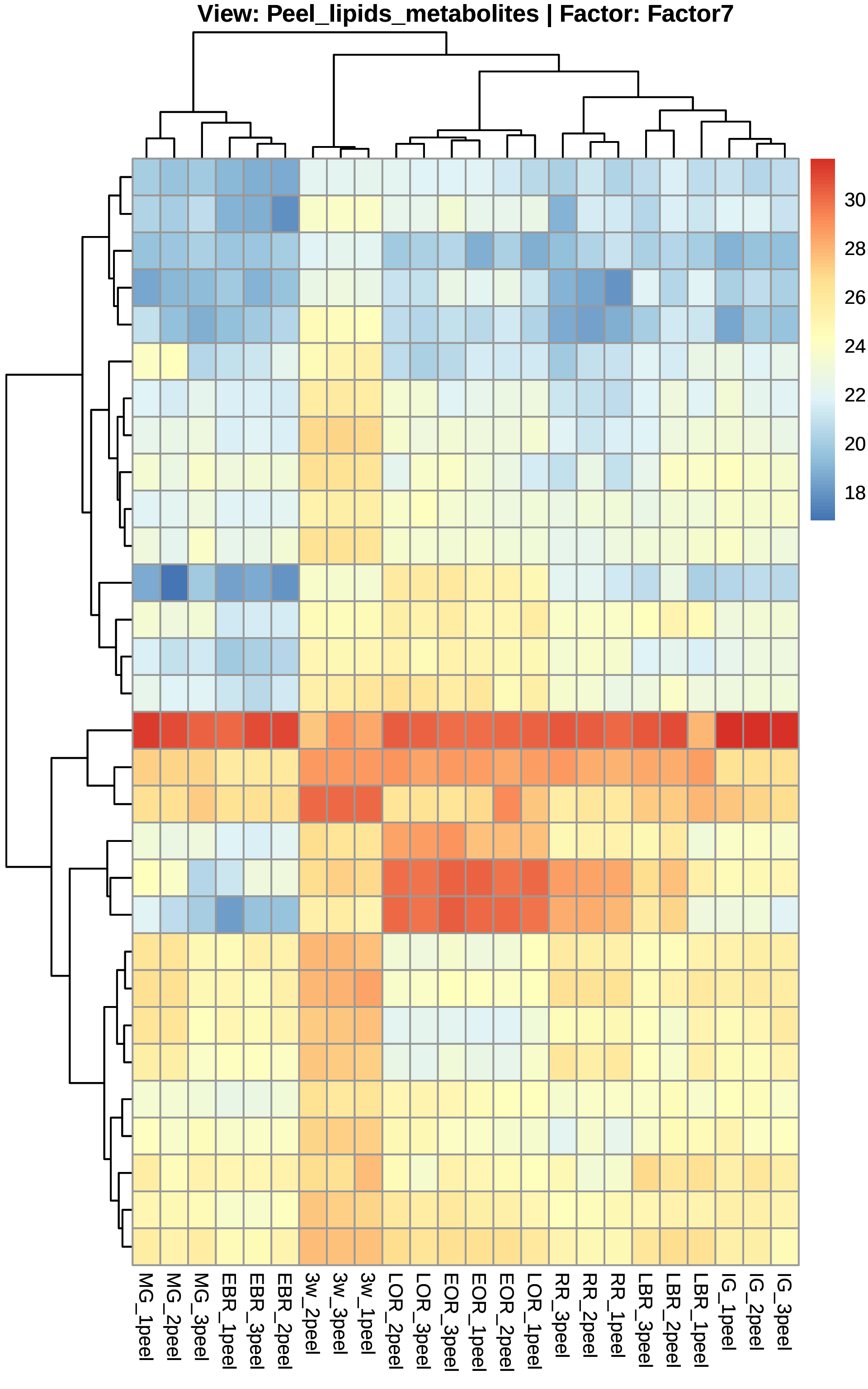
View: Peel_lipids_metabolites | Factor: Factor7
30
28
26
24
22
20
18
MG_1peel
MG_2peel
MG_3peel
EBR_1peel
EBR_3peel
EBR_2peel
3w_2peel
3w_3peel
3w_1peel
LOR_2peel
LOR_3peel
EOR_3peel
EOR_1peel
EOR_2peel
LOR_1peel
RR_3peel
RR_2peel
RR_1peel
LBR_3peel
LBR_2peel
LBR_1peel
IG_1peel
IG_2peel
IG_3peel

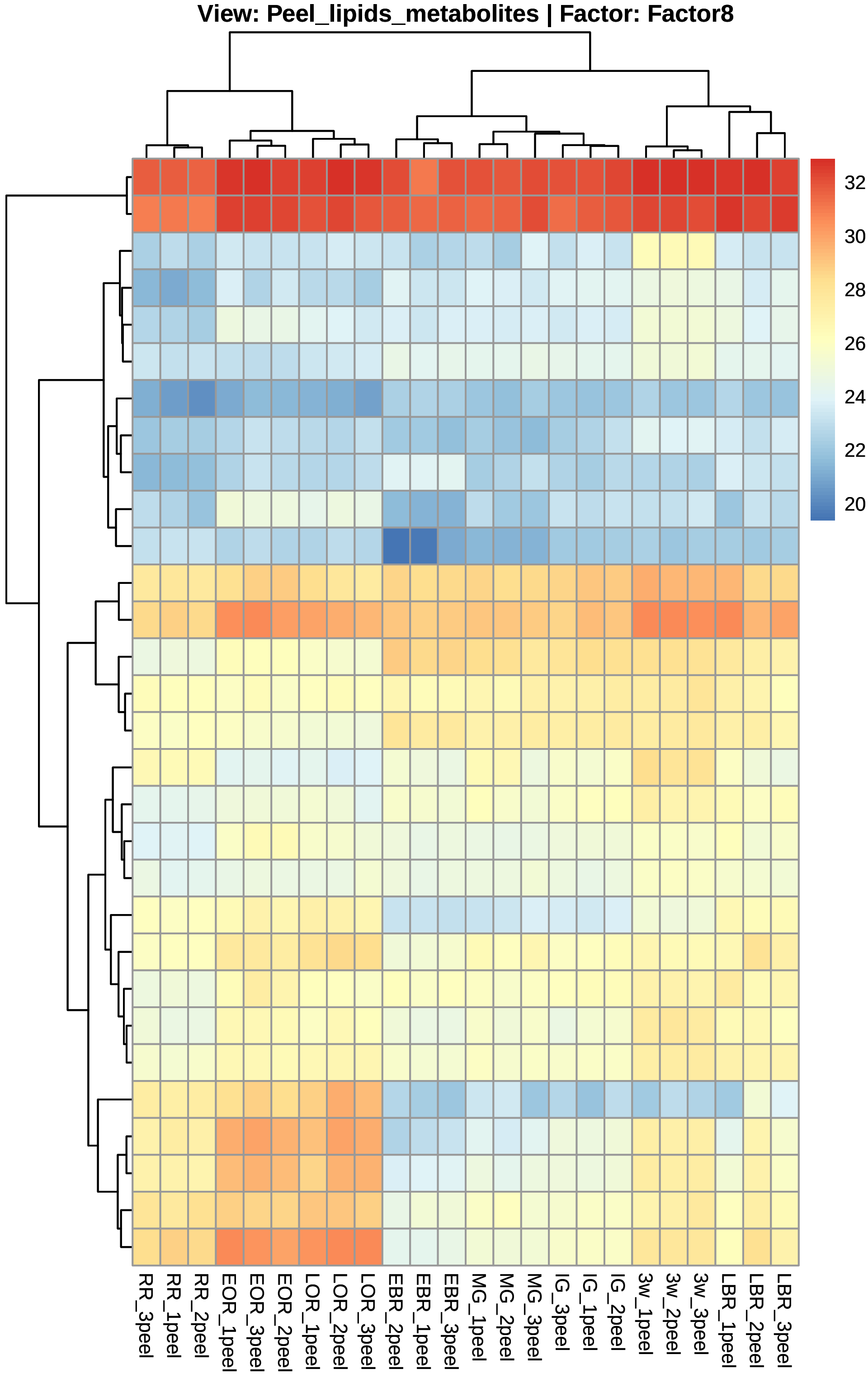

View: Peel_lipids_metabolites | Factor: Factor8
32
30
28
26
24
22
20
RR_3peel
RR_1peel
RR_2peel
EOR_1peel
EOR_3peel
EOR_2peel
LOR_1peel
LOR_2peel
LOR_3peel
EBR_2peel
EBR_1peel
EBR_3peel
MG_1peel
MG_2peel
MG_3peel
IG_3peel
IG_1peel
IG_2peel
3w_1peel
3w_2peel
3w_3peel
LBR_1peel
LBR_2peel
LBR_3peel

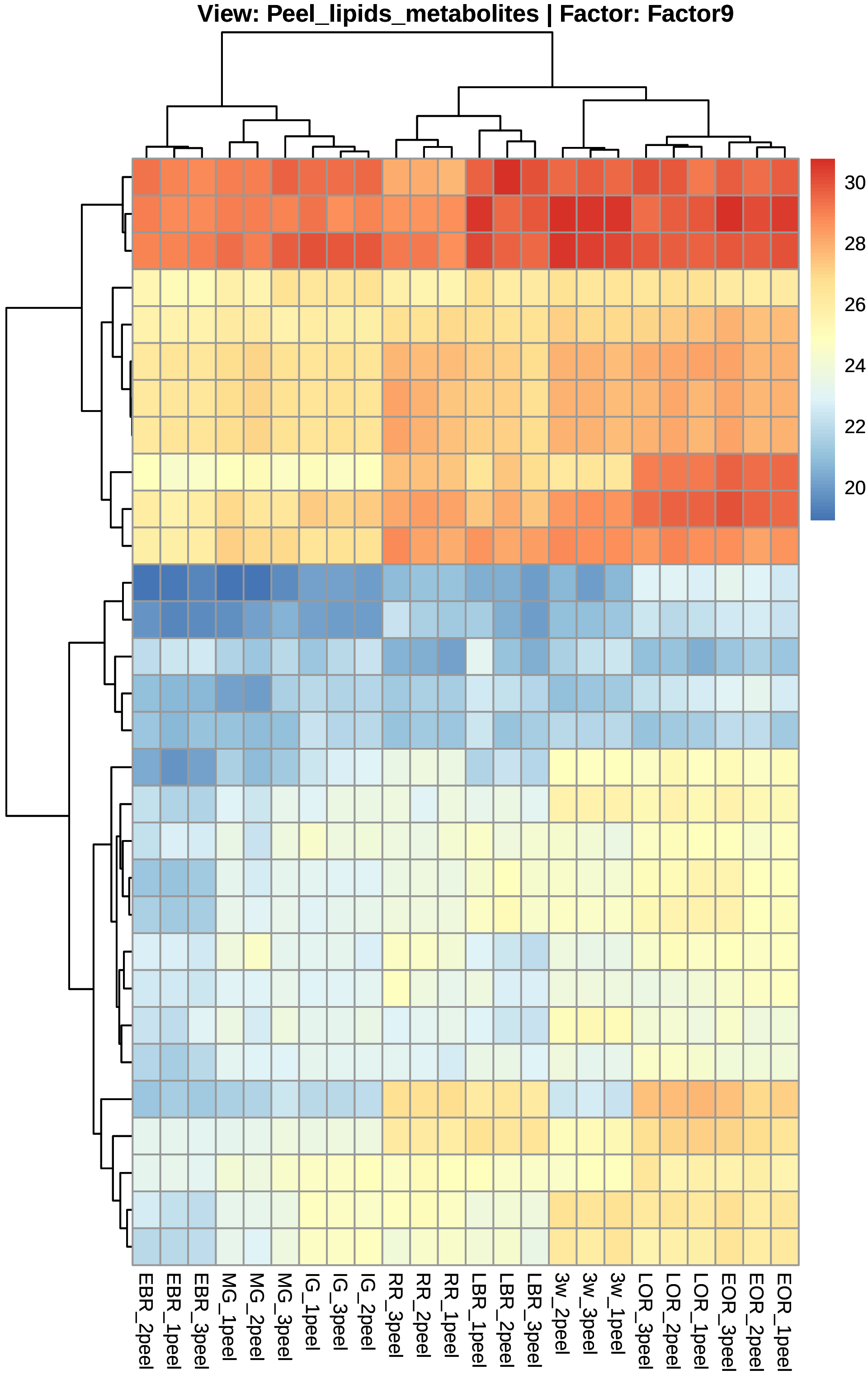

View: Peel_lipids_metabolites | Factor: Factor9
30
28
26
24
22
20
EBR_2peel
EBR_1peel
EBR_3peel
MG_1peel
MG_2peel
MG_3peel
IG_1peel
IG_3peel
IG_2peel
RR_3peel
RR_2peel
RR_1peel
LBR_1peel
LBR_2peel
LBR_3peel
3w_2peel
3w_3peel
3w_1peel
LOR_3peel
LOR_2peel
LOR_1peel
EOR_3peel
EOR_2peel
EOR_1peel

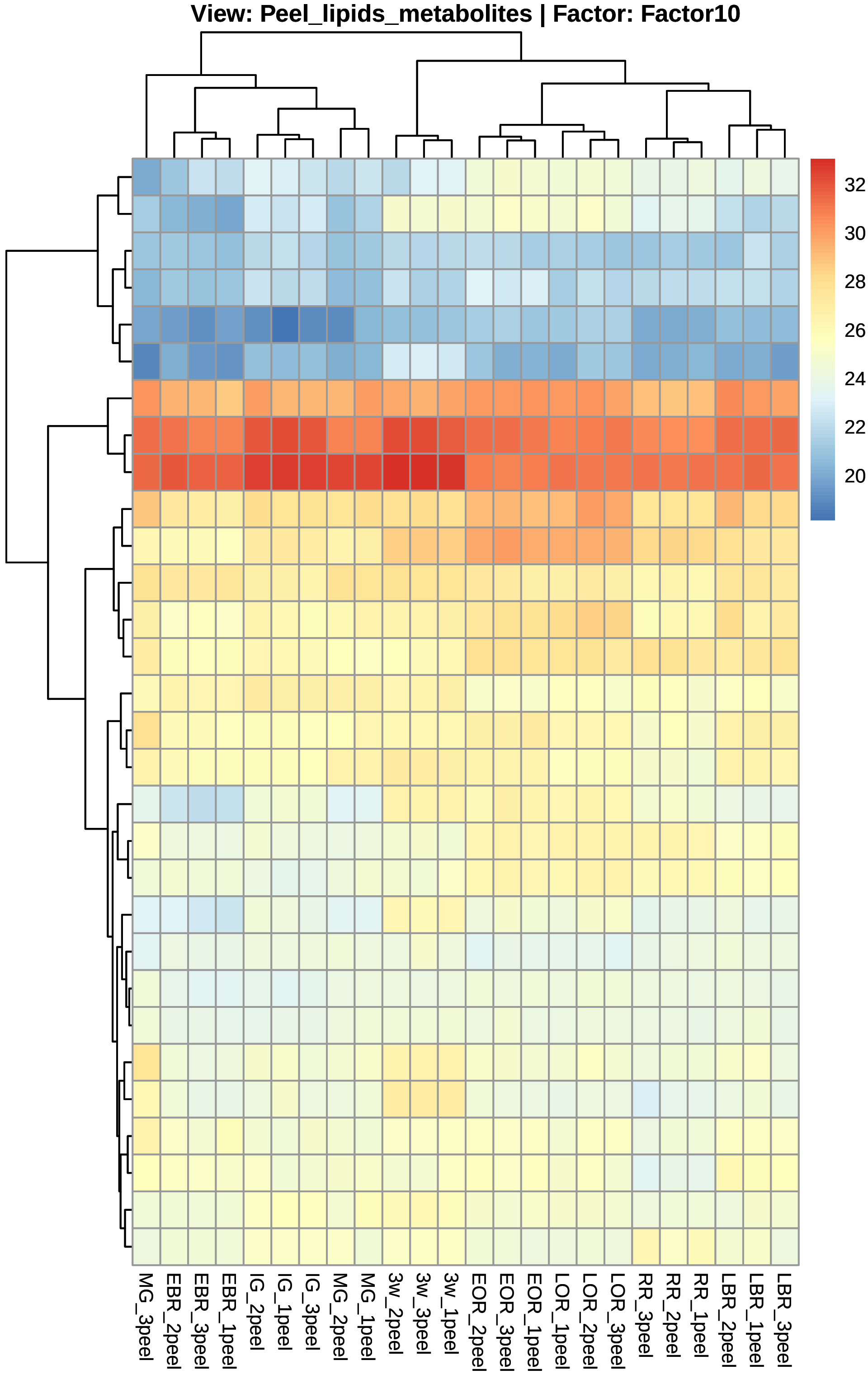
View: Peel_lipids_metabolites | Factor: Factor10
32
30
28
26
24
22
20
MG_3peel
EBR_2peel
EBR_3peel
EBR_1peel
IG_2peel
IG_1peel
IG_3peel
MG_2peel
MG_1peel
3w_2peel
3w_3peel
3w_1peel
EOR_2peel
EOR_3peel
EOR_1peel
LOR_1peel
LOR_2peel
LOR_3peel
RR_3peel
RR_2peel
RR_1peel
LBR_2peel
LBR_1peel
LBR_3peel

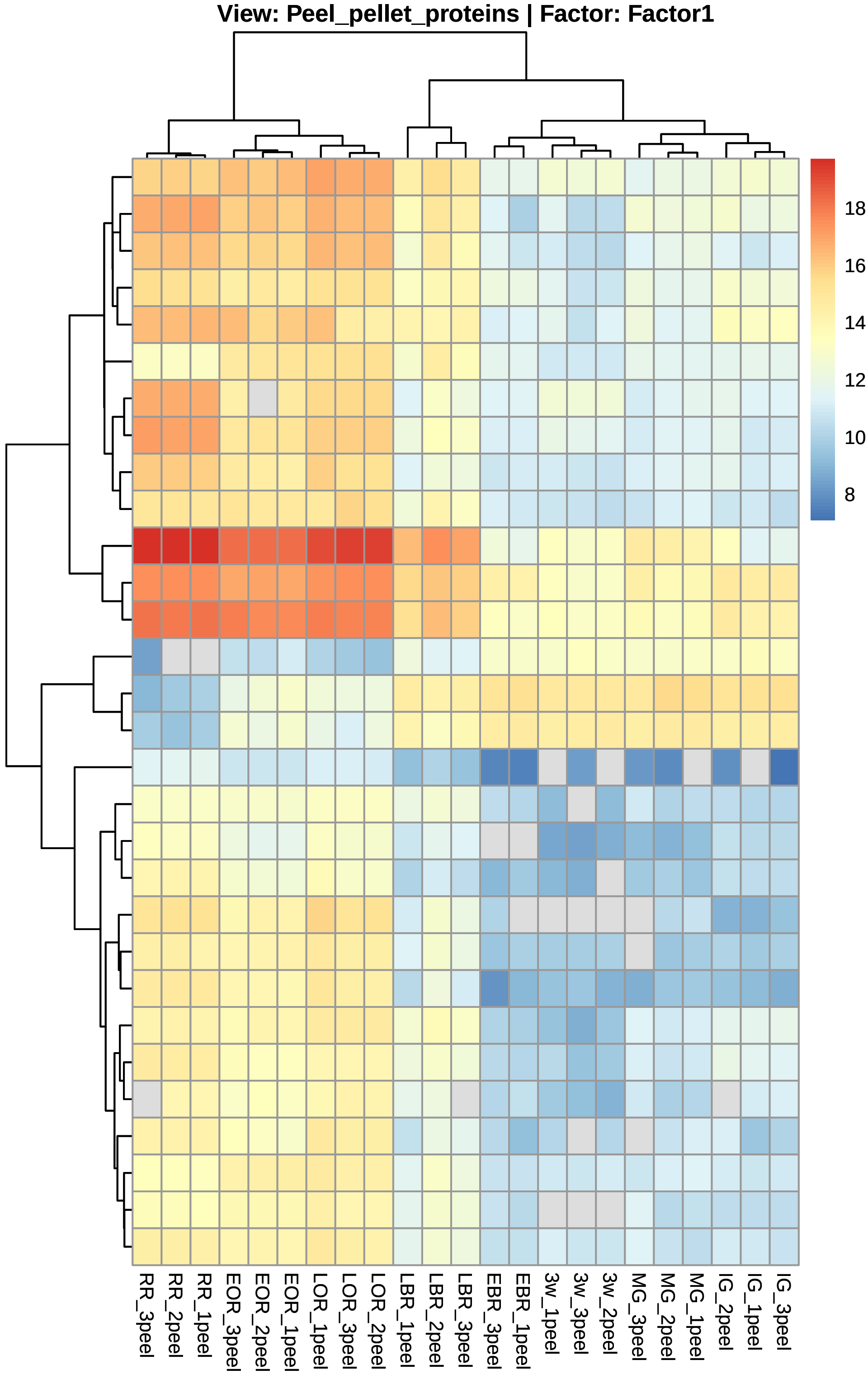
View: Peel_pellet_proteins | Factor: Factor1
18
16
14
12
10
8
RR_3peel
RR_2peel
RR_1peel
EOR_3peel
EOR_2peel
EOR_1peel
LOR_1peel
LOR_3peel
LOR_2peel
LBR_1peel
LBR_2peel
LBR_3peel
EBR_3peel
EBR_1peel
3w_1peel
3w_3peel
3w_2peel
MG_3peel
MG_2peel
MG_1peel
IG_2peel
IG_1peel
IG_3peel

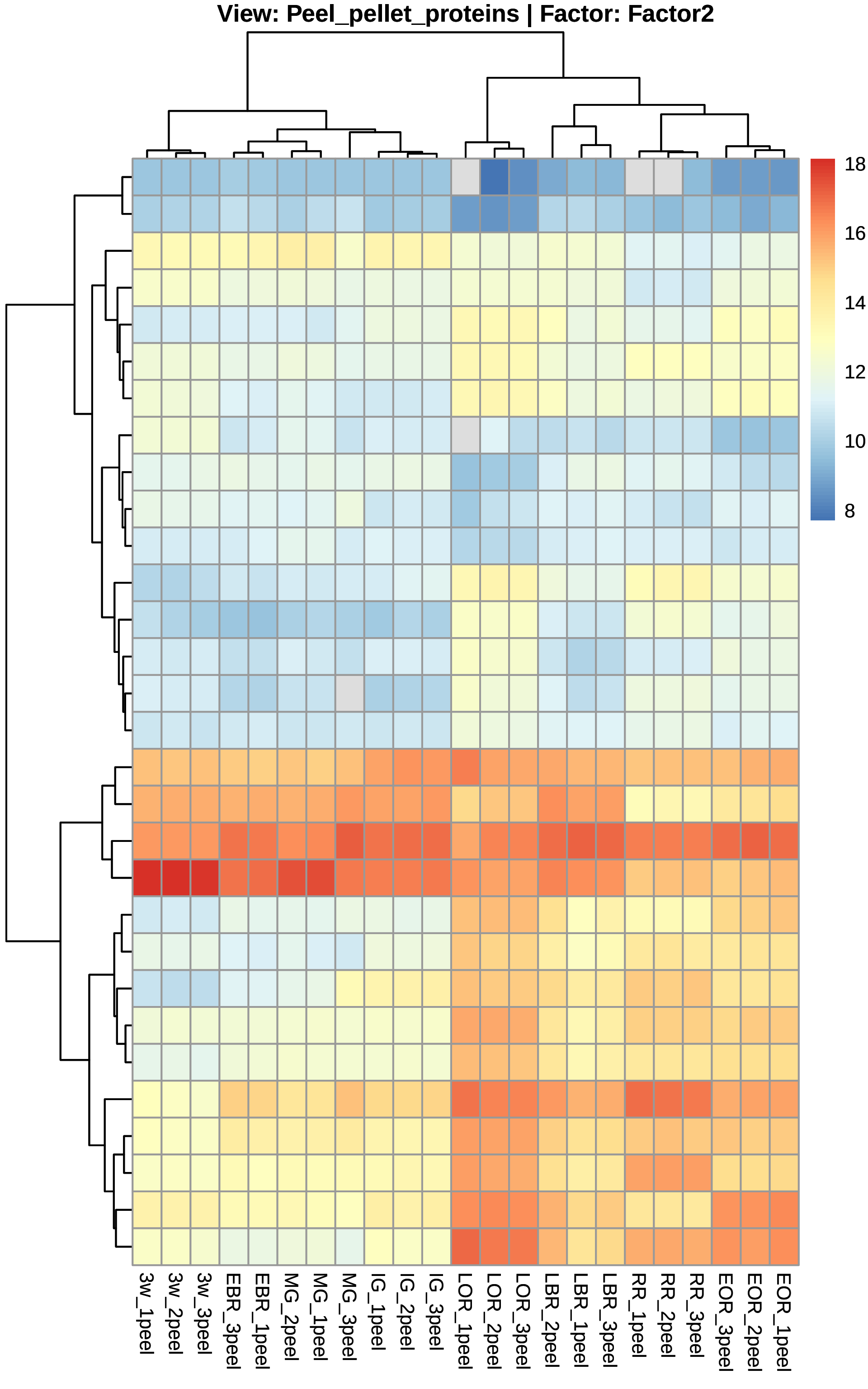

View: Peel_pellet_proteins | Factor: Factor2
18
16
14
12
10
8
3w_1peel
3w_2peel
3w_3peel
EBR_3peel
EBR_1peel
MG_2peel
MG_1peel
MG_3peel
IG_1peel
IG_2peel
IG_3peel
LOR_1peel
LOR_2peel
LOR_3peel
LBR_2peel
LBR_1peel
LBR_3peel
RR_1peel
RR_2peel
RR_3peel
EOR_3peel
EOR_2peel
EOR_1peel

View: Peel_pellet_proteins | Factor: Factor3

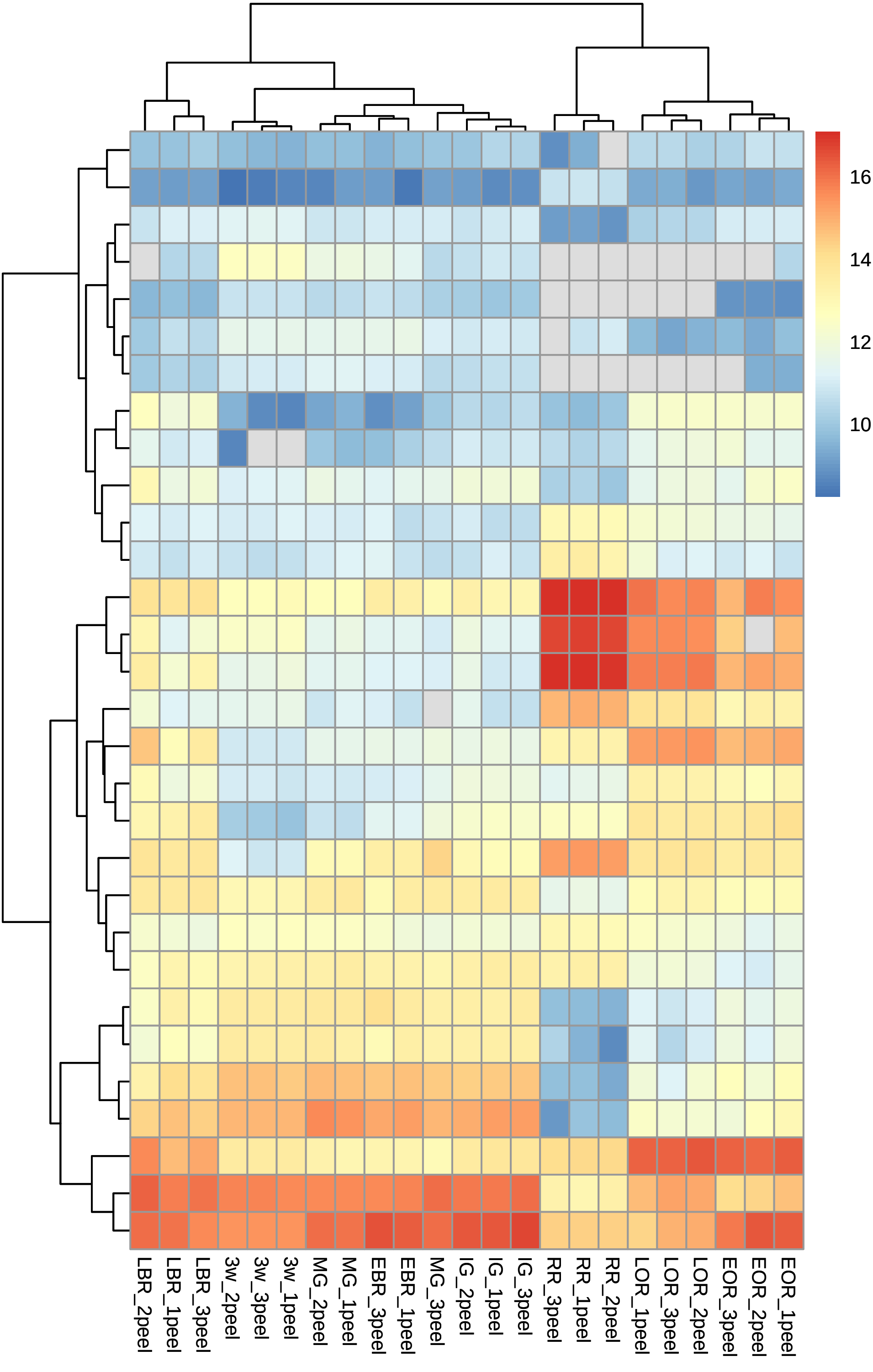

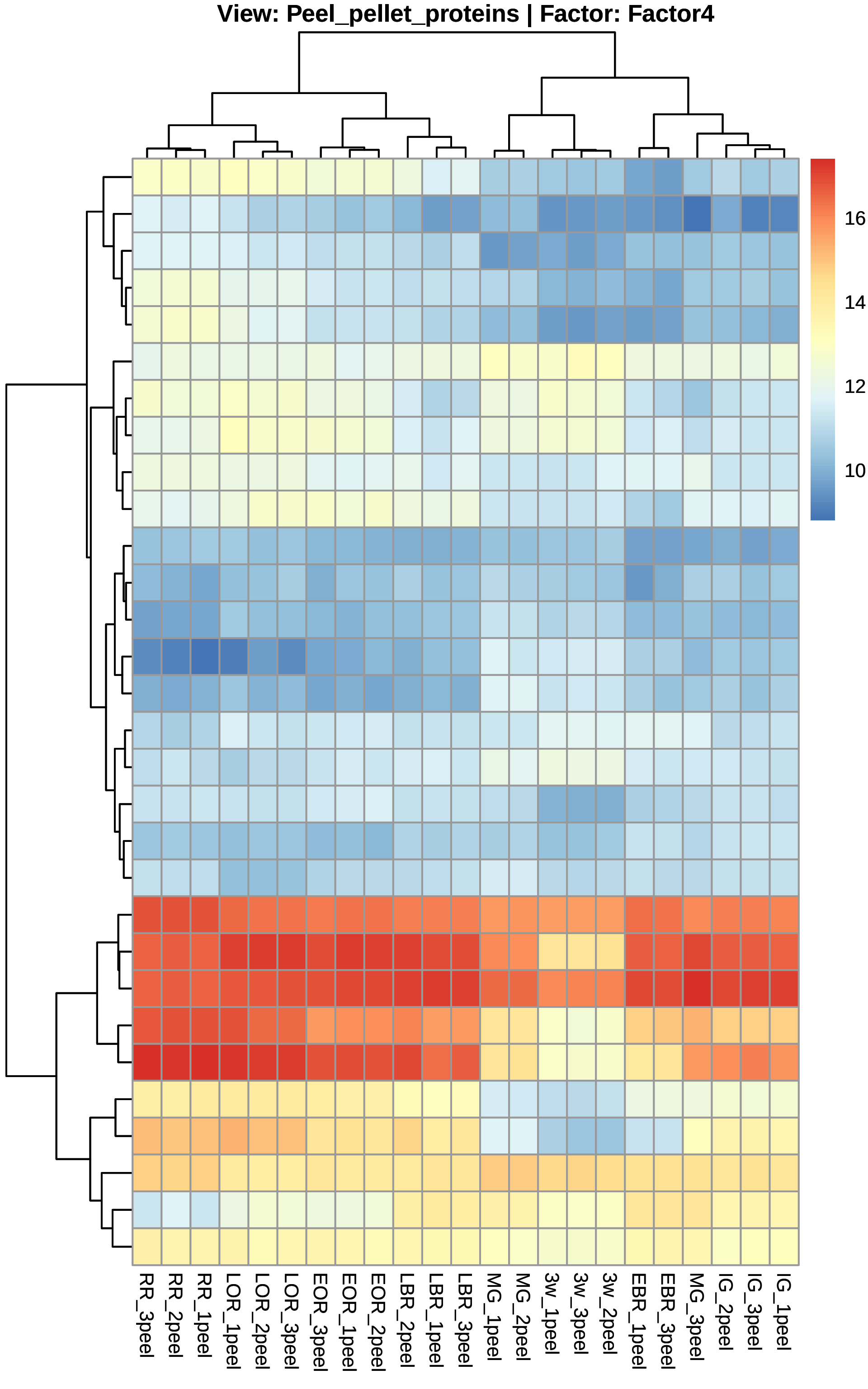
View: Peel_pellet_proteins | Factor: Factor4
16
14
12
10
RR_3peel
RR_2peel
RR_1peel
LOR_1peel
LOR_2peel
LOR_3peel
EOR_3peel
EOR_1peel
EOR_2peel
LBR_2peel
LBR_1peel
LBR_3peel
MG_1peel
MG_2peel
3w_1peel
3w_3peel
3w_2peel
EBR_1peel
EBR_3peel
MG_3peel
IG_2peel
IG_3peel
IG_1peel

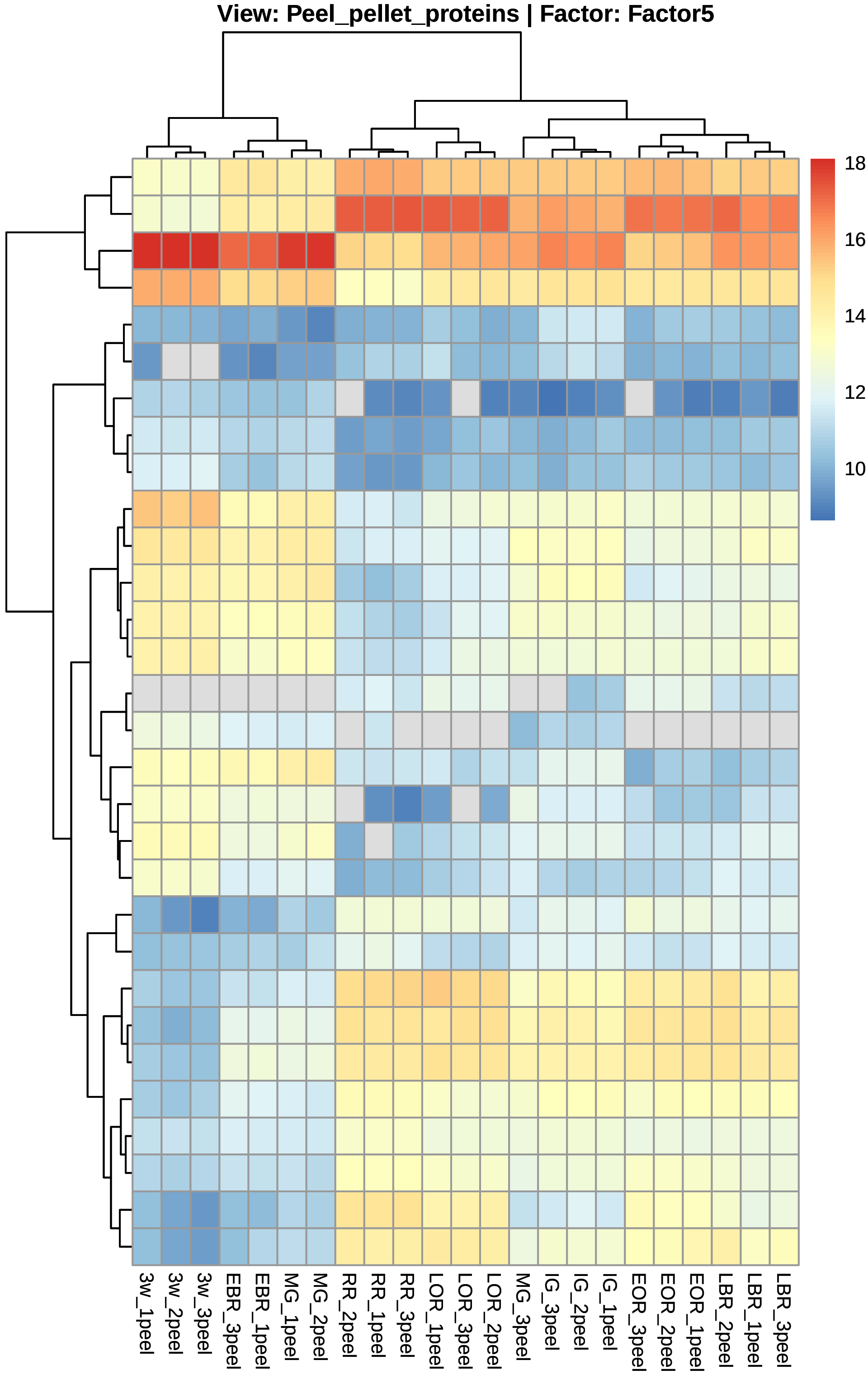

View: Peel_pellet_proteins | Factor: Factor5
18
16
14
12
10
3w_1peel
3w_2peel
3w_3peel
EBR_3peel
EBR_1peel
MG_1peel
MG_2peel
RR_2peel
RR_1peel
RR_3peel
LOR_1peel
LOR_3peel
LOR_2peel
MG_3peel
IG_3peel
IG_2peel
IG_1peel
EOR_3peel
EOR_2peel
EOR_1peel
LBR_2peel
LBR_1peel
LBR_3peel

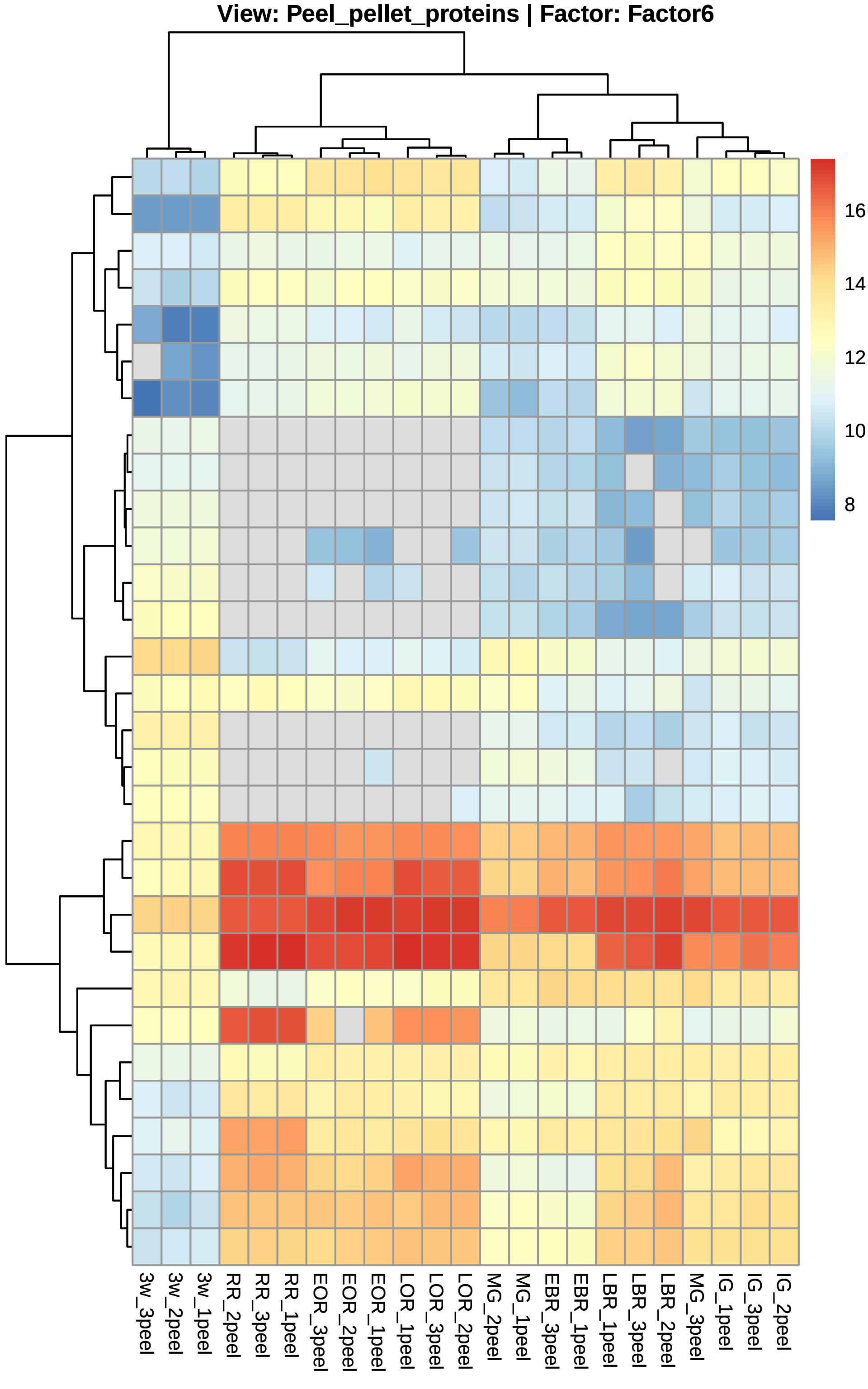
View: Peel_pellet_proteins | Factor: Factor6
16
14
12
10
8
3w_3peel
3w_2peel
3w_1peel
RR_2peel
RR_3peel
RR_1peel
EOR_3peel
EOR_2peel
EOR_1peel
LOR_1peel
LOR_3peel
LOR_2peel
MG_2peel
MG_1peel
EBR_3peel
EBR_1peel
LBR_1peel
LBR_3peel
LBR_2peel
MG_3peel
IG_1peel
IG_3peel
IG_2peel

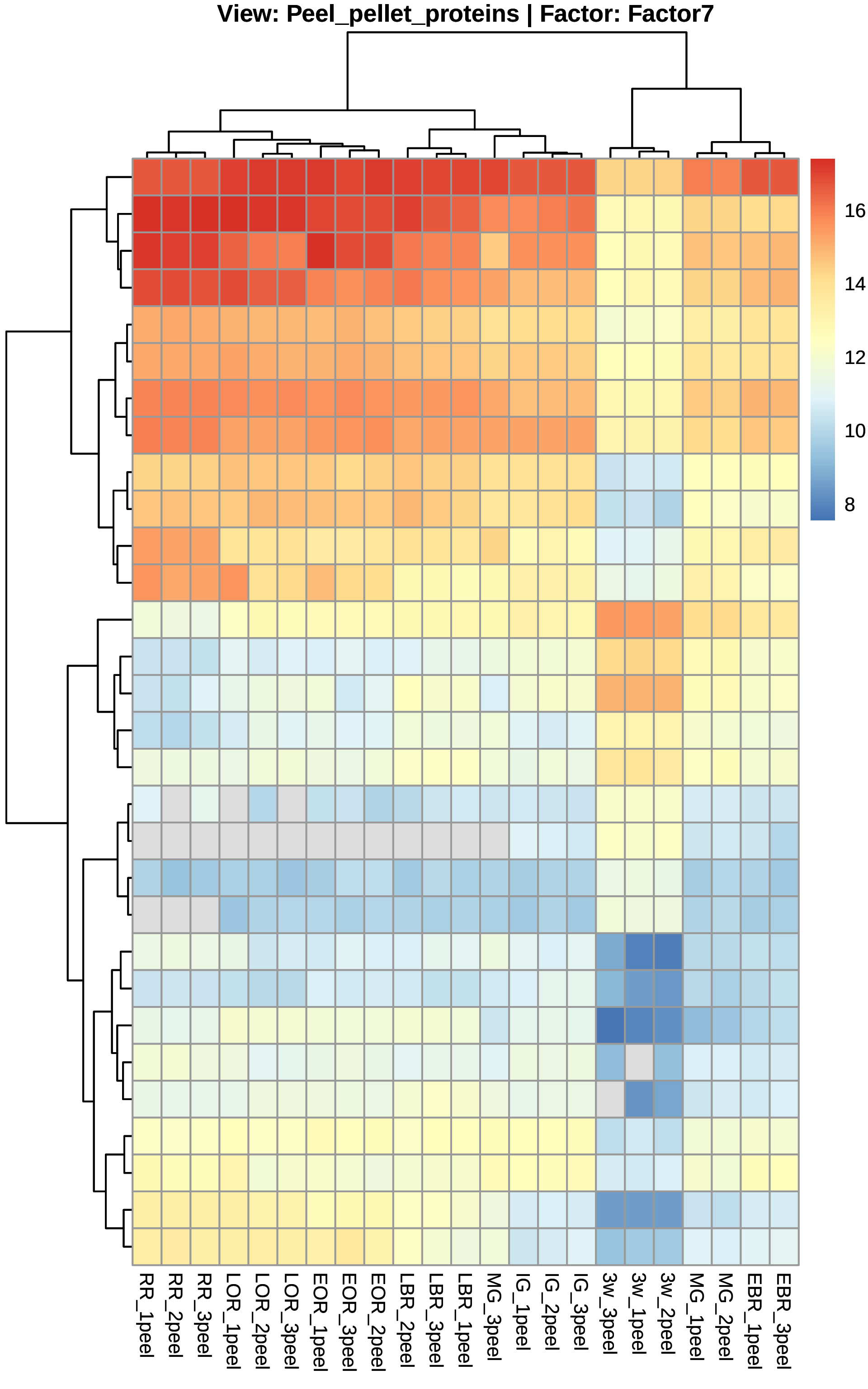

View: Peel_pellet_proteins | Factor: Factor7
16
14
12
10
8
RR_1peel
RR_2peel
RR_3peel
LOR_1peel
LOR_2peel
LOR_3peel
EOR_1peel
EOR_3peel
EOR_2peel
LBR_2peel
LBR_3peel
LBR_1peel
MG_3peel
IG_1peel
IG_2peel
IG_3peel
3w_3peel
3w_1peel
3w_2peel
MG_1peel
MG_2peel
EBR_1peel
EBR_3peel

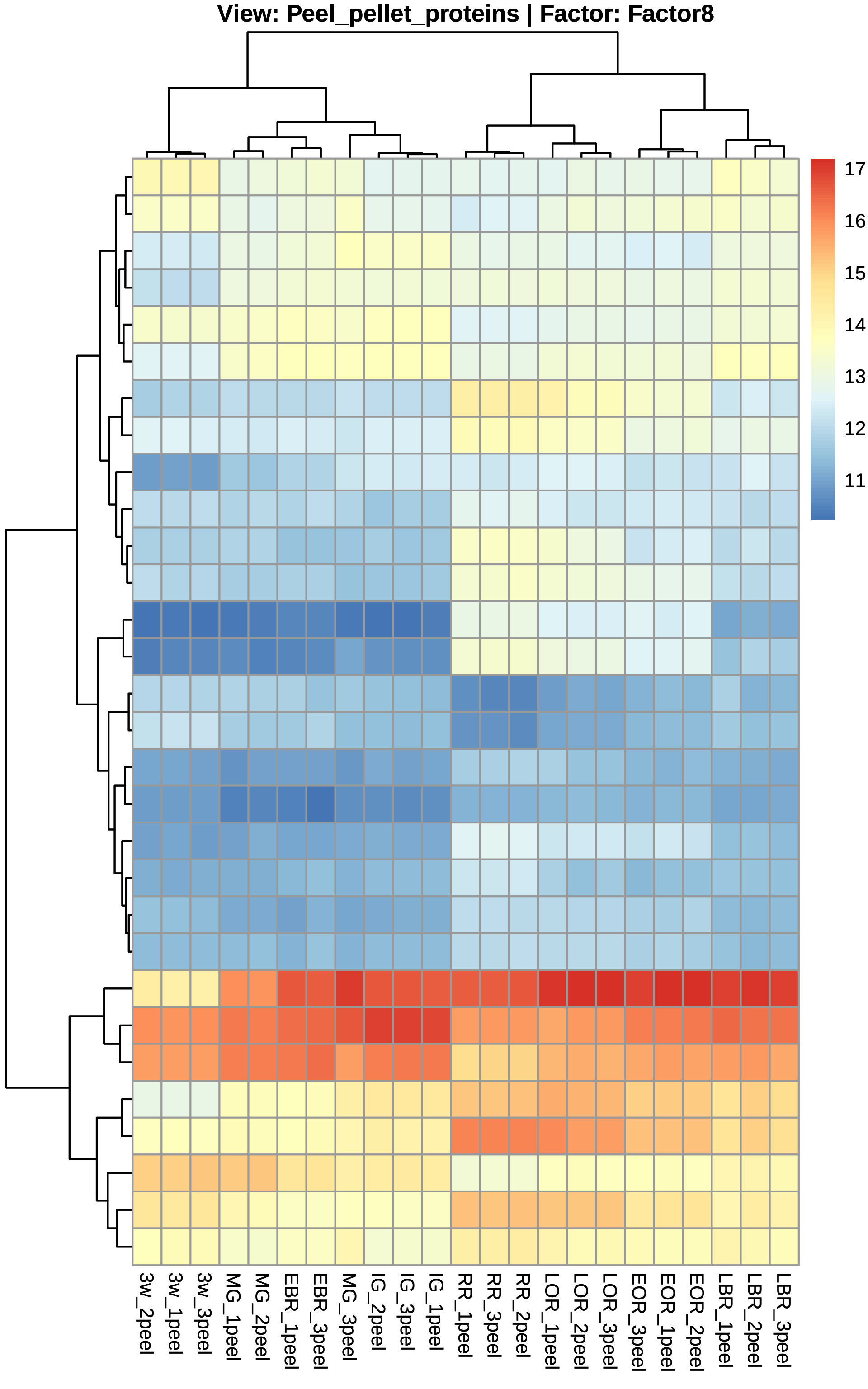

View: Peel_pellet_proteins | Factor: Factor8
17
16
15
14
13
12
11
3w_2peel
3w_1peel
3w_3peel
MG_1peel
MG_2peel
EBR_1peel
EBR_3peel
MG_3peel
IG_2peel
IG_3peel
IG_1peel
RR_1peel
RR_3peel
RR_2peel
LOR_1peel
LOR_2peel
LOR_3peel
EOR_3peel
EOR_1peel
EOR_2peel
LBR_1peel
LBR_2peel
LBR_3peel

View: Peel_pellet_proteins | Factor: Factor9

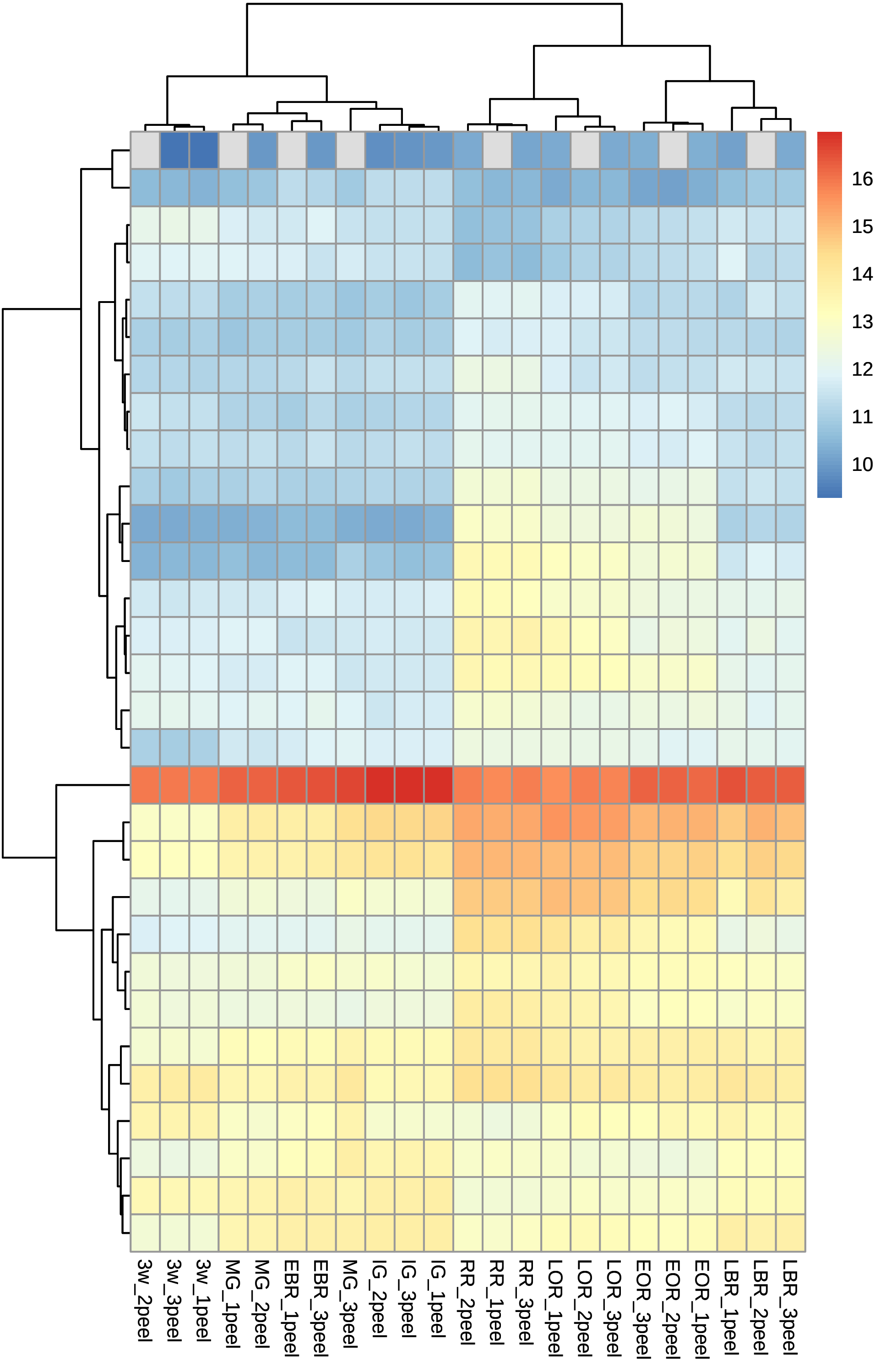

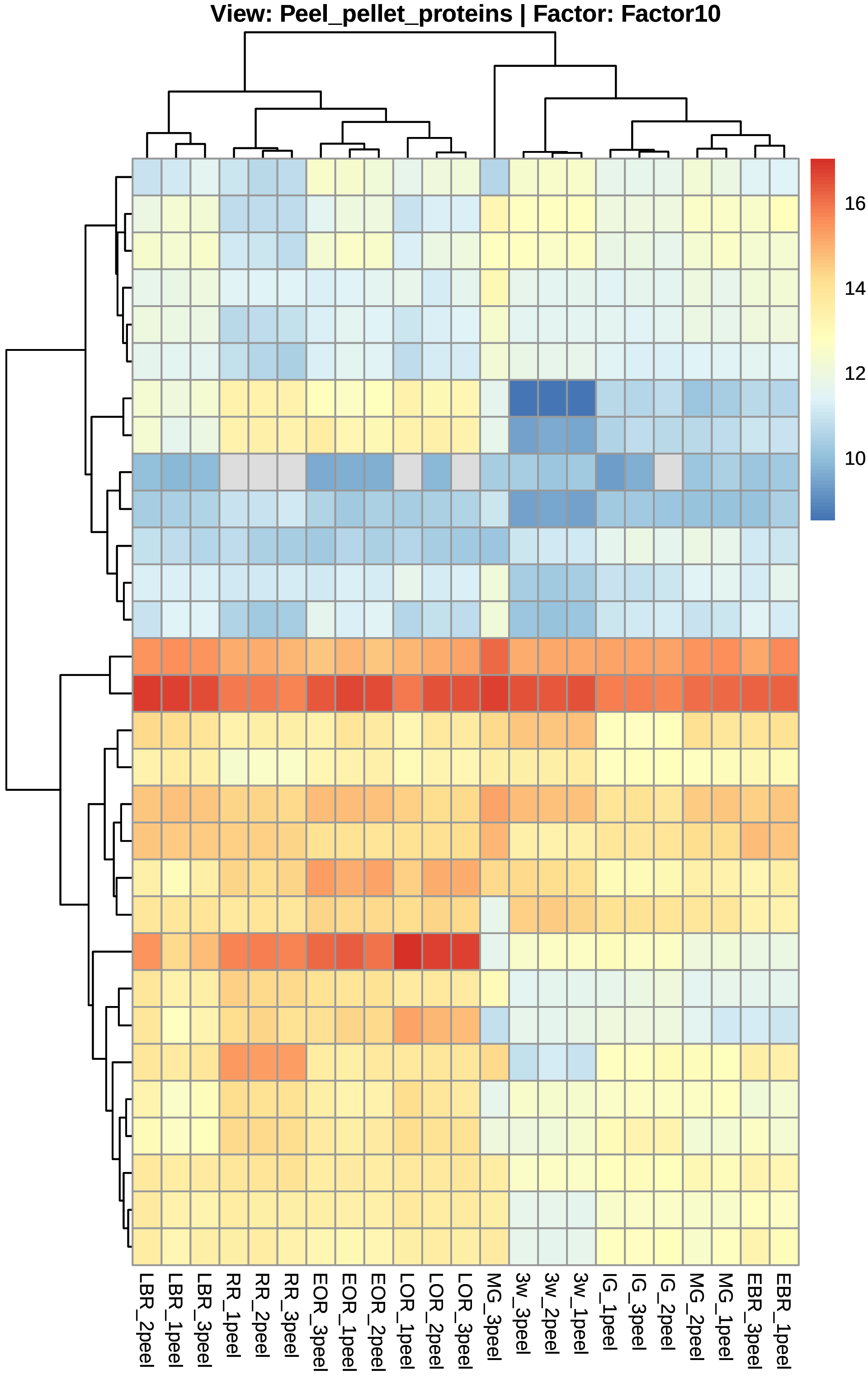
View: Peel_pellet_proteins | Factor: Factor10
16
14
12
10
LBR_2peel
LBR_1peel
LBR_3peel
RR_1peel
RR_2peel
RR_3peel
EOR_3peel
EOR_1peel
EOR_2peel
LOR_1peel
LOR_2peel
LOR_3peel
MG_3peel
3w_3peel
3w_2peel
3w_1peel
IG_1peel
IG_3peel
IG_2peel
MG_2peel
MG_1peel
EBR_3peel
EBR_1peel
